# Iridium-Catalyzed Stereocontrolled C(sp^3^)–C(sp^3^) Cross-Coupling of Boronic Esters and Allylic Carbonates Enabled by Boron-to-Zinc Transmetalation

**DOI:** 10.1021/jacs.4c17931

**Published:** 2025-02-06

**Authors:** Hong-Cheng Shen, Varinder K. Aggarwal

**Affiliations:** School of Chemistry, University of Bristol, Cantock’s Close, Bristol BS8 1TS, U.K.

## Abstract

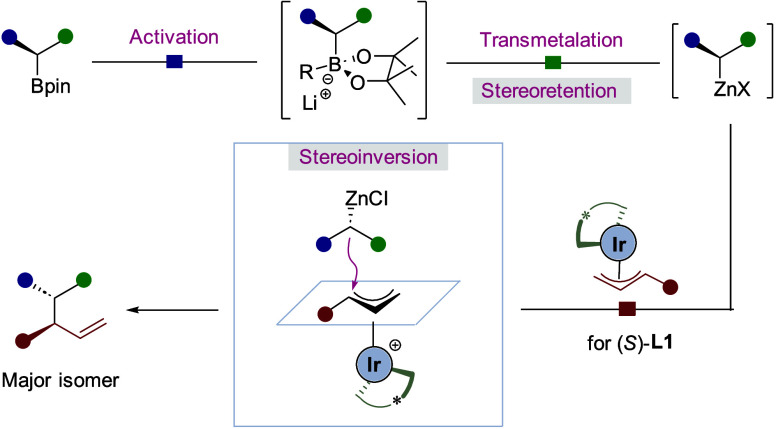

The stereocontrolled C(sp^3^)–C(sp^3^) cross-coupling represents a considerable challenge of great contemporary interest. While this has been achieved through the reactions of boronate complexes with π-allyl iridium complexes, such reactions suffered from a limited substrate scope. We now report that following transmetalation from boronate complexes to organozinc reagents enables previously unreactive substrates to engage in stereocontrolled C(sp^3^)–C(sp^3^) cross-coupling. The broader substrate scope has enabled their application to the synthesis of biologically active molecules. The organozinc reagents react through a stereoinvertive coupling pathway with π-allyl iridium complexes, in contrast to reactions with other electrophiles that occur with retention of stereochemistry. The reaction uniquely combines the enantiospecific reactivity of an enantioenriched organometallic nucleophile with the enantioselective engagement of a racemic electrophile, enabling access to all stereoisomers.

Fuelled by the greater clinical success with an increasing proportion of sp^3^-rich fragments,^1^ there has been increased interest in cross-coupling reactions that form C(sp^3^)–C(sp^3^) bonds, particularly with stereocontrol.^2^ Transition metal-catalyzed stereocontrolled cross-coupling reactions between organometallic nucleophiles and electrophiles are one of the most powerful tools for constructing enantioenriched C(sp^3^)-rich building blocks.^3^ On the basis of the stereochemical outcome, these reactions can broadly be classified as enantiospecific cross-coupling (Scheme 1a, top) and enantioselective cross-coupling (Scheme 1a, bottom), distinguished by whether the chirality originates from the starting material or is induced by the catalyst. These two classes of stereocontrolled cross-coupling reactions typically yield products with only one stereogenic center. However, stereocontrolled synthesis of vicinal stereogenic centers, enabled by a single chiral transition metal catalyst is still challenging.^4^

**Scheme 1:**
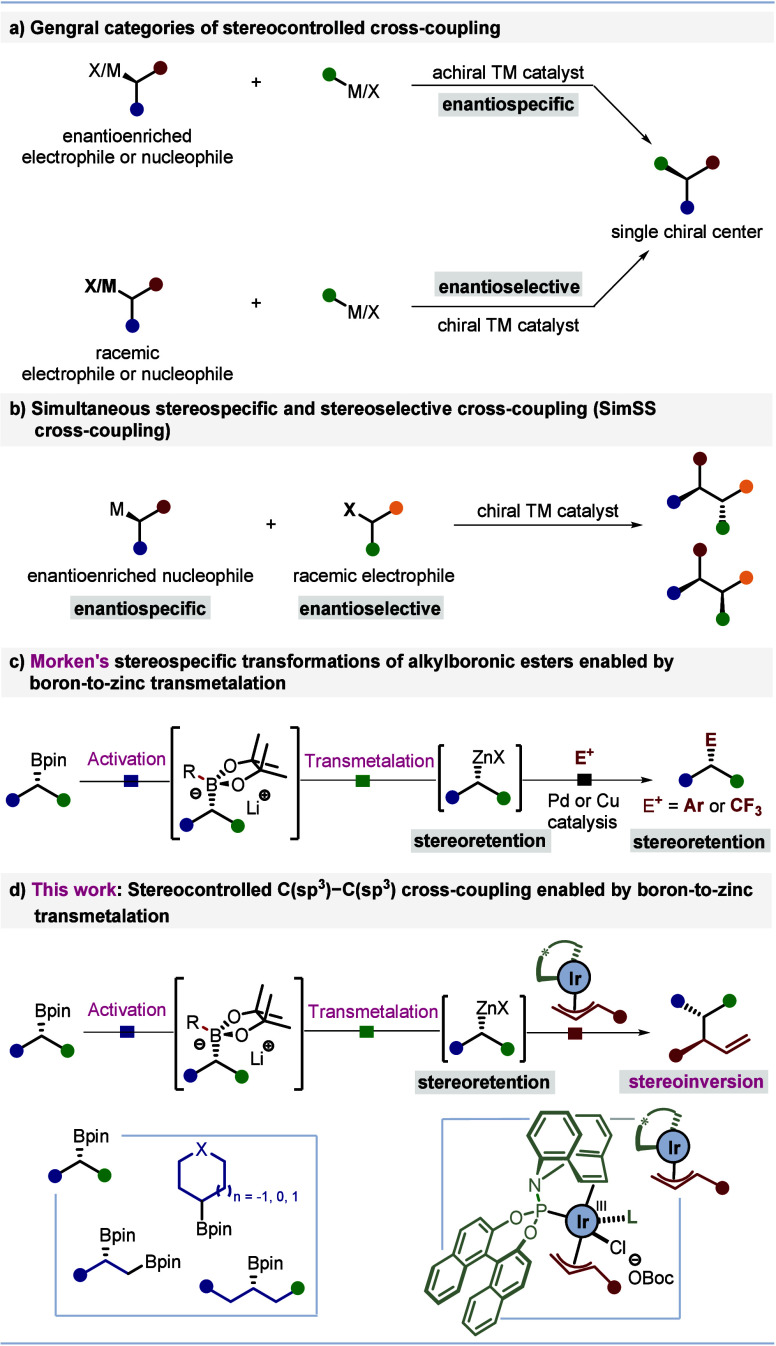
Stereocontrolled Cross-Coupling and Our Reaction Design

To address this challenge, we recently reported an approach that merged the broad features of enantiospecific and enantioselective cross-couplings into a single process (SimSS cross-coupling, Scheme 1b): the simultaneous enantiospecific reaction of an enantioenriched organometallic nucleophile^5^ with the enantioselective reaction of a racemic electrophile.^6,7^ In this work we exploited the enantioselective reaction of electrophilic π-allyl iridium complexes^8,9^ with the enantiospecific reaction of nucleophilic boronate complexes.^10,11^ However, attempts to extend the generality of this methodology to less reactive substrates, such as small and medium-sized cyclic boronic esters, 1,2-bis(boronic) esters, and nonbenzylic secondary boronic esters, were unsuccessful, as the boronate complexes were not sufficiently reactive. To increase the reactivity, we considered the transmetalation of a boronate complex to a more reactive organometallic nucleophile. Recently, Morken demonstrated that enantioenriched secondary boronate complexes could undergo efficient transmetalation with zinc salts that could be trapped by a range of electrophiles with retention of configuration (Scheme 1c).^12^ Additionally, Carreira, Cho, and Li and Zhan have shown that functional primary zinc reagents,^13^ (diborylmethyl)zinc,^14^ and racemic α-boryl benzyliczinc reagents^15^ serve as efficient nucleophiles in reactions with π-allyl iridium complexes.

We now report that the boron-to-zinc transmetalation process enables previously unreactive substrates to participate in SimSS cross-coupling (Scheme 1d). However, unlike Morken’s findings^12,16^ and those of Knochel,^17^ Campos,^18^ Gawley,^19^ Lautens,^20^ and Marek,^21^ where reactions of organozincates with electrophiles occurred with retention of configuration, we have found that they react with inversion with π-allyl iridium complexes. The divergent behavior was analyzed and rationalized.

The reaction of N-Boc-piperidine-4-boronic ester **1** with racemic allylic carbonate **2a** was initially explored. Full optimization of the process is provided in the Supporting Information, but key findings are discussed below (Table 1). Treatment of the boronic ester with ^*t*^BuLi, followed by ZnCl_2_ and subsequent addition of carbonate **2a**, along with the iridium catalyst and chiral ligand (*S*)-**L1**, furnished the cross-coupling product **4** in 30% yield with excellent enantioselectivity (98:2 er, entry 1). A systematic evaluation of different solvent systems revealed that using a 1:1 mixture of THF and hexane provided improved results compared to THF alone or other solvent mixtures (entries 2–4), delivering a yield of 57% while retaining excellent enantioselectivity (entry 4). Alternative allylic carbonates **2b** and **2c** with different leaving groups showed reduced yields or enantioselectivity (entries 5 and 6). An increase in reaction temperature led to a marginal increase in yield (61%) while preserving excellent enantioselectivity (>99:1 er, entries 7 and 8). Notably, the reaction failed to deliver the product when the boron-to-zinc transmetalation process was omitted (entry 9). Finally, while other zinc salts were tested, ZnCl_2_ remained optimum (entries 10–13). Interestingly, when Zn(OAc)_2_ and Zn(OPiv)_2_ were used, we found that significant amounts of side products were observed arising from acetate and pivalate anions attacking the π-allyl iridium complex (see Figure S5 in the Supporting Information).

**Table 1:**
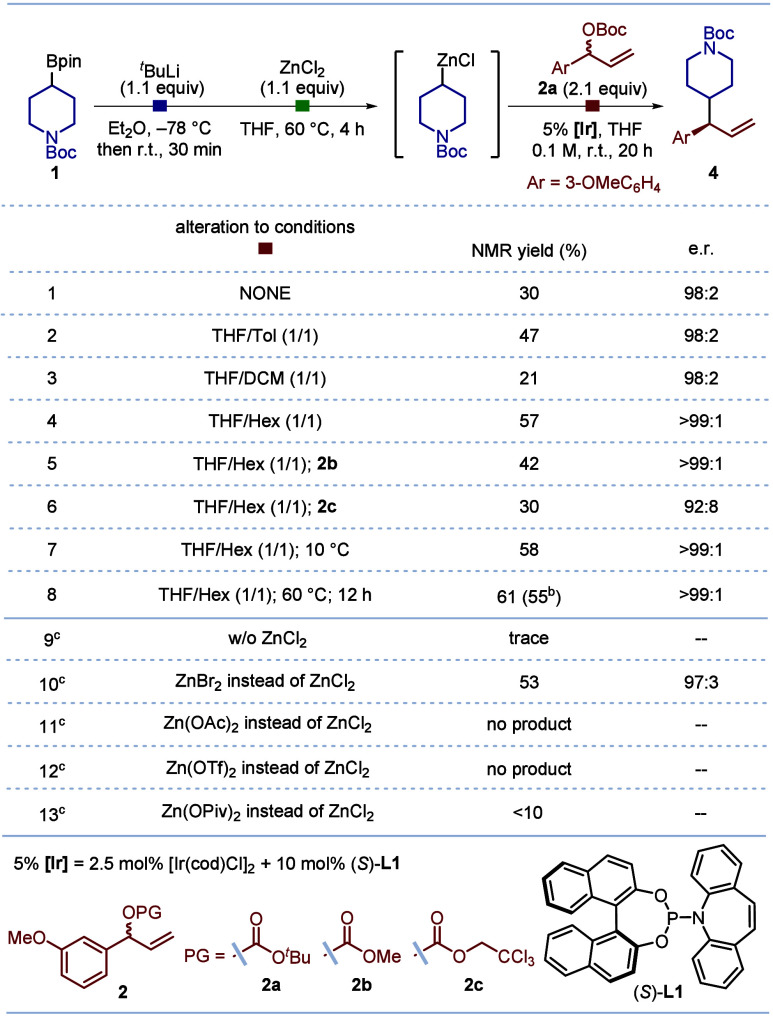
Reaction Optimization^a^

a Reactions were conducted on 0.2 mmol scale as shown in scheme; er was determined by HPLC analysis.

b Isolated yield.

c THF/Hex (1/1); 60 °C; 12 h.

Having established suitable conditions, we embarked on evaluating the generality of this stereocontrolled cross-coupling (Table 2, top). We first investigated the scope with respect to racemic allylic carbonate. The reaction accommodated various substituents on the phenyl group, including both electron-donating and electron-withdrawing groups at the *meta*-, *para*-, and *ortho*- positions, giving products **4**, **7**–**15** with yields ranging from 34%–67%, and excellent enantioselectivity (98:2 to >99:1 er). Allylic carbonates derived from other aromatic rings, including naphthalene, 2-chloroquinoline, and thiophene, were also suitable partners, leading to the formation of the corresponding products **16**–**18** in good yields with excellent selectivity. Notably, alkenyl- and alkynyl-substituted allylic carbonates were readily converted to coupling products, **19** and **20**, again with excellent enantioselectivity. However, attempts to extend the reaction to aliphatic allyl electrophiles were unsuccessful; no product was obtained. The absolute configuration of enantioenriched product **11** was determined by X-ray crystallographic analysis.

**Table 2:**
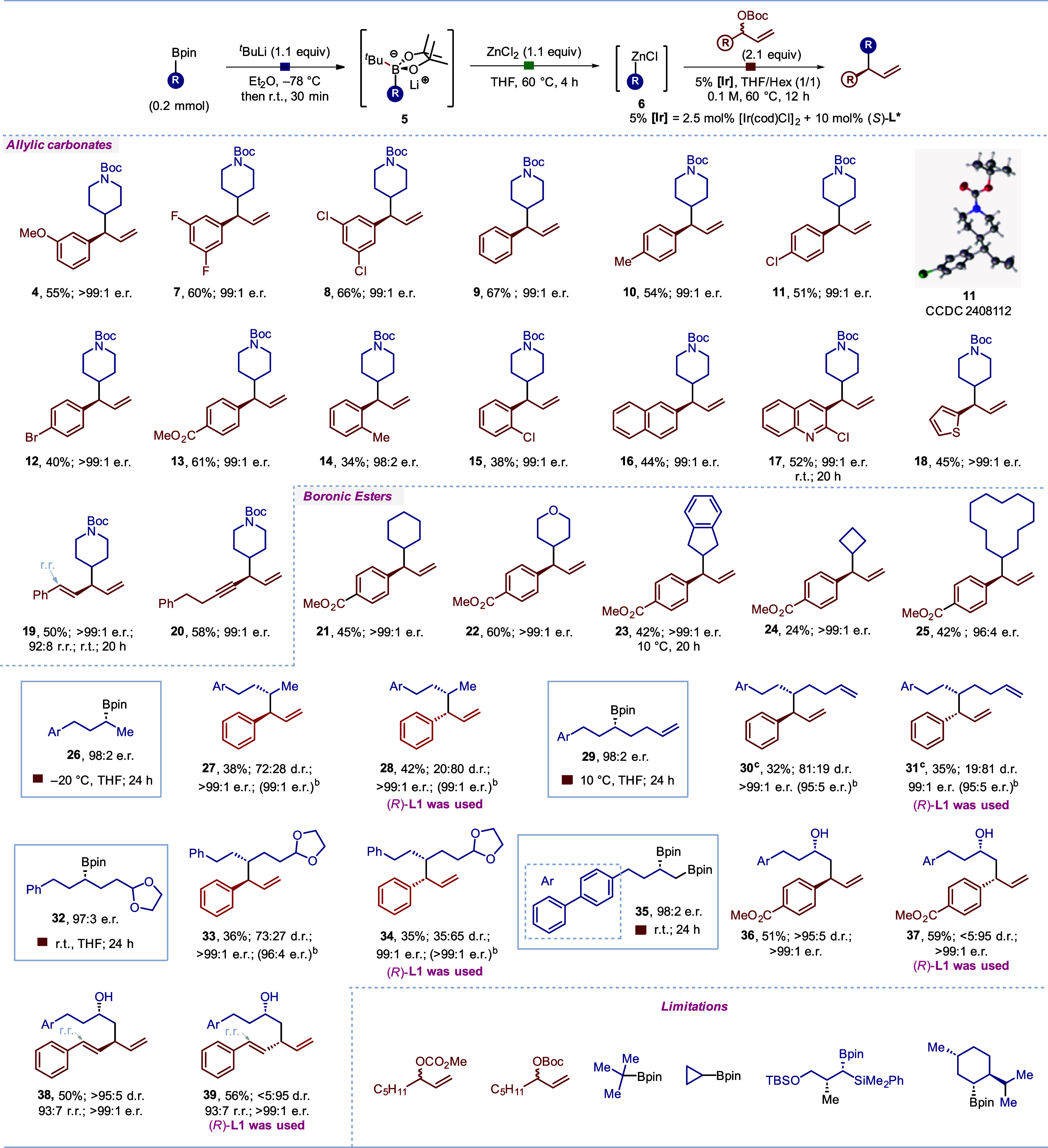
Substrate Scope^a^

a Reactions were conducted on 0.2 mmol scale as shown in scheme; yields are of isolated products; e.r. was determined by HPLC analysis. Transmetaltion process of enantioenriched boronic esters was performed at r.t. for 2 h. The d.r. was determined by GCMS (**27** and **28**, **36** and **37**) or ^1^H NMR (**30** and **31**, **33** and **34**, **38** and **39**) analysis.

b e.r. of minor diastereomer.

c [Ir(cod)Cl]_2_ (5 mol %)/**L1** (20 mol %) was used.

The scope of the boronic esters was subsequently examined (Table 2, bottom). Various cyclic boronic esters, such as cyclohexyl (**21**), tetrahydropyran-4-yl (**22**), 2,3-dihydro-1H-inden-2-yl (**23**), cyclobutyl (**24**), and cyclododecyl (**25**), delivered moderate to good yields with excellent enantioselectivity. However, the conformationally rigid cyclopropyl boronic ester was found to be unreactive, presumably because of the increased barrier due to ring strain in the invertive pathway. For enantioenriched secondary boronic esters (**26**, **29**, and **32**), our process produced the corresponding products (**27**, **30**, and **33**) in moderate yields, with good diastereoselectivity and excellent enantioselectivity for both diastereomers. Using the enantiomeric ligand (*R*)-**L1** afforded the opposite diastereomers (**28**, **31**, and **34**) with comparable reactivity and selectivity, indicating the absence of significant matched/mismatched effects. Finally, the enantioenriched 1,2-bis(boronic) esters **35** was investigated. Stereocontrolled coupling occurred exclusively at the terminal position, via a β-boryl-alkylzinc intermediate,^22^ affording the products **36**–**39** in good yields and with high selectivities.

We conducted a series of experiments to investigate the mechanism of the coupling reaction between the boronic ester and allylic carbonate (Scheme 2). To determine whether the racemic carbonate **2a** underwent kinetic resolution, we carried out allylation with boronic ester **1** and isolated both the coupling product **4** and the unreacted carbonate **2a** (Scheme 2a). The recovered carbonate, (*R*)-**2a**, was obtained in 99:1 er, indicating that an efficient kinetic resolution had occurred.

**Scheme 2:**
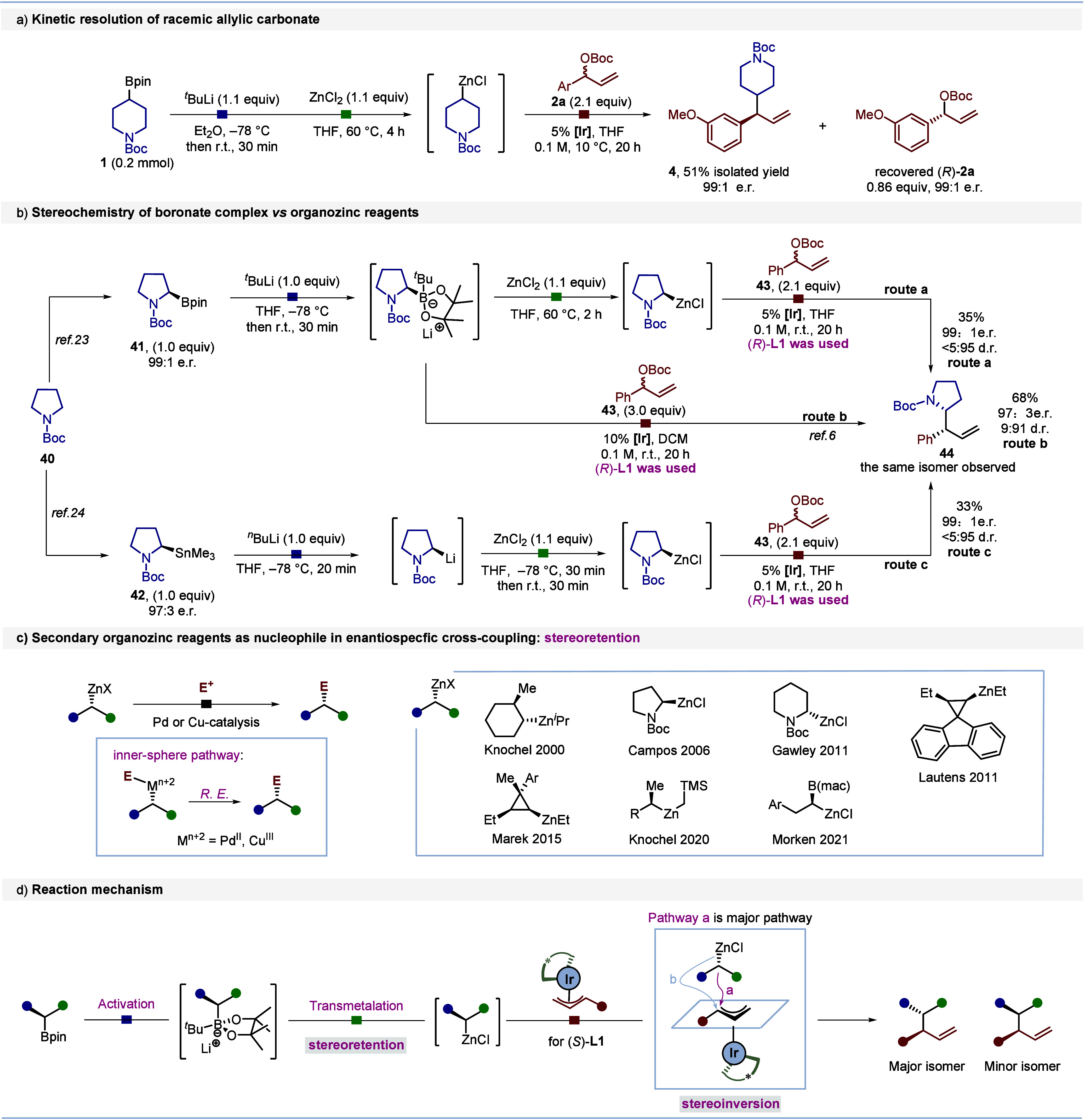
Mechanistic Studies^a^ All reactions were conducted on a 0.2 mmol scale, yields are of isolated products; e.r. was determined by HPLC analysis. The d.r. was determined by ^1^H NMR partial erosion in stereospecificity of the reaction of the organozinc reagent had occurred.

We wanted to compare the stereochemical outcome of the reactions of boronate complexes and zincates with the π-allyl iridium complexes. The boronate complexes were known to react with inversion^6,10^ of configuration and zincates were known to react with retention^12,16−21^ of configuration with other electrophiles. Thus, boronic ester **41**(23) was employed as a starting material for coupling with carbonate **43** (Scheme 2b) using either the boron-to-zinc transmetalation process (**route a**) or directly via the boronate complex (**route b**).^6^ Surprisingly, both routes **a** and **b** gave the same major isomer **44**, as confirmed by HPLC analysis (see Figure S2 in the Supporting Information). Since the boron-to-zinc transmetalation process occurs with retention of configuration, this indicates that both the boronate complex and the organozincate react with inversion of configuration with the π-allyl-Ir complex.

Further proof of the stereochemical outcome was achieved by subjecting the organozinc reagent derived from the known organotin compound **42**(24) to the same reaction conditions (**route c**), which once again yielded the same major product **44**. Since the stereochemistry of the different steps along **route c** has been proven in Negishi cross-couplings,^18,24^ and **route a** and **c** give the same product, this proves that the boron-to-zinc transmetalation (**route a**) occurs with retention of configuration, in keeping with Morken’s findings.^12^ For substrates where lower diastereoselectivity was observed (**30** and **31**), we determined, by careful analysis of the HPLC (see Figure S3 in the Supporting Information), that erosion had taken place at what had been the α-boryl carbon center rather than the carbonate center. Since the boron-to-zinc transmetalation process is stereospecific, this indicates that the divergent behavior of organozincates with electrophiles can be accounted for by whether they react through an inner or outer sphere mechanism. In cases where they react under Pd or Cu catalysis,^12,16−21^ they react through an inner sphere mechanism and the reactions occur with retention (Scheme 2c); however, in reactions with π-ally iridium complexes, they react through an outer sphere mechanism and the reactions occur with inversion. Of note, these reactions are distinct from stereoselective cross couplings of organozinc reagents under Ni catalysis, which, being radical in nature, are stereoconvergent.^4,25^

These observations support the proposed reaction mechanism depicted in Scheme 2d.^9b,26^ The boronic ester is activated by *tert*-butyllithium to form a boronate complex, which undergoes stereoretentive transmetalation to generate an enantioenriched organozinc reagent. The organozinc reagent attacks the π-allyl iridium complex through a stereoinvertive pathway (pathway a), leading to the corresponding major product. However, the observed erosion in the stereospecificity of the organozinc stereocenter could arise from a competing (minor) stereoretentive pathway (pathway b).

To underscore the synthetic potential of this stereocontrolled cross-coupling reaction, we investigated its application in both gram-scale transformations and the synthesis of bioactive motifs, as illustrated in Scheme 3. The reaction was successfully scaled up (Scheme 3a), producing piperidine **7** in 58% yield (1.37 g) while maintaining excellent enantioselectivity, a testament to the robustness and scalability of the method. Piperidine **7** was then subjected to a three-step synthetic sequence, culminating in the formation of piperidine **46**. This compound serves as a key intermediate in the synthesis of AZD5672, a drug candidate for the treatment of rheumatoid arthritis.^27^ In a similar vein, the cross-coupling reaction was applied to the synthesis of enantioenriched quinuclidine derivatives, specifically quinuclidine **49**, as depicted in Scheme 3b. Following ozonolysis and reduction of **4**, alcohol **47** was converted to bromide **48**. Subsequent Boc deprotection followed by cyclization gave quinuclidine **49**. These quinuclidine derivatives are of significant pharmacological interest due to their role as ligands for nonselective nicotinic acetylcholine receptors (nAChRs).^28^

**Scheme 3:**
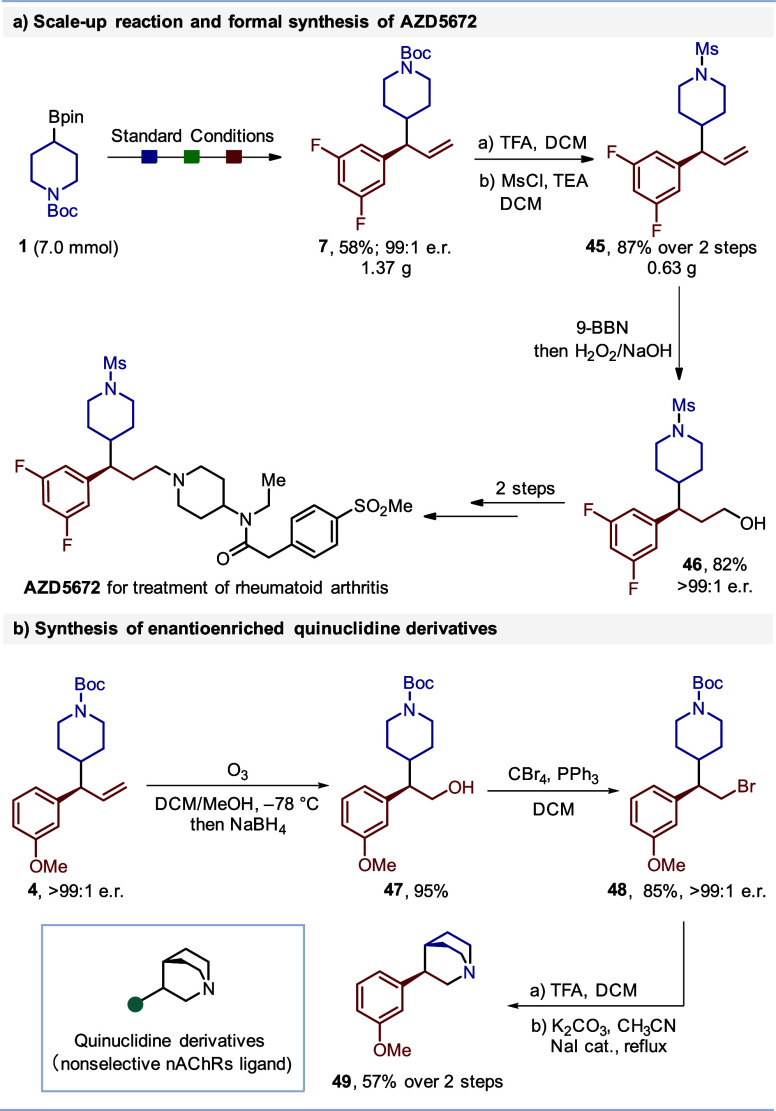
Scale Up and Synthesis of Bioactive Moleculars See the Supporting Information for reaction conditions.

In conclusion, we have successfully developed an iridium-catalyzed, stereocontrolled cross-coupling reaction between boronic esters and racemic allylic carbonates enabled by a boron-to-zinc transmetalation strategy. This method demonstrates a broad scope, including small and medium-sized cyclic boronic esters, 1,2-bis(boronic) esters, and nonbenzylic secondary boronic esters, affording enantioenriched sp^3^-rich building blocks equipped with an alkenyl handle for further derivation. The synthetic utility of this method has been exemplified by efficient transformations of the products into bioactive motifs. Importantly, our mechanistic investigations indicate that the organozinc reagents react with inversion of configuration with π-allyl-iridium complexes, in contrast to other electrophiles where they react with retention.

## Supporting Information Available

Experimental procedures and characterization data for new compounds (PDF)
