# Supplementary material for: Iridium-Catalyzed Stereocontrolled C(sp3)–C(sp3) Cross-Coupling of Boronic Esters and Allylic Carbonates Enabled by Boron-to-Zinc Transmetalation
Source: J Am Chem Soc. 2025 Feb 6;147(7):5583–9. doi: 10.1021/jacs.4c17931 (PMC11848825; doi:10.1021/jacs.4c17931)
Supplement: Supplementary file 1 — ja4c17931_si_001.pdf [file ja4c17931_si_001.pdf]

Supplementary Information for

Iridium-Catalyzed Stereocontrolled C(sp^3)–C(sp^3) Cross-Coupling of Boronic Esters and Allylic Carbonates Enabled by Boron-to-Zinc Transmetalation

Hong-Cheng Shen and Varinder K. Aggarwal*

School of Chemistry, University of Bristol, Cantock's Close, Bristol, BS8 1TS, U.K.

Correspondence to: v.aggarwal@bristol.ac.uk

Contents

1. MATERIALS AND GENERAL METHODS **3**

1.1. Glassware, Solvents and Reagents 3

1.2. Instrumentation 3

1.3. Naming of Compounds 4

2. EXPERIMENTAL DATA **5**

2.1. Reaction Optimization 5

2.2. HPLC Analysis for Reaction Mechanism 8

2.3. General Procedures 11

2.3.1. General Procedure A for product **4**, **7-25**. 11

2.3.2. General Procedure B for product **27-28**. 12

2.3.3. General Procedure C for product 3**0-31**, **33-34**. 13

2.3.4. General Procedure D for product **36-39** 14

2.3.5. Procedures for stereochemistry analysis of brornate complex vs organozinc reagents. 15

2.4. Synthesis of Starting Materials 17

2.4.1. Synthesis of racemic allylic carbonates 17

2.4.2. Characterization data for boronic esters 17

2.4.3. Characterization data for **42** 19

2.5. Procedures for Transformation of Products 21

2.6. Crystallography 25

2.7. Characterization Data for Products. 27

3. REFERENCE 62

4. NMR SPECTRA 63

1. MATERIALS AND GENERAL METHODS

1.1. Glassware, Solvents and Reagents

All manipulations were performed with oven-dried (130 °C for a minimum of 12 h) or flame-dried glassware using standard Schlenk techniques under an atmosphere of nitrogen, unless otherwise stated.

All anhydrous solvents were commercially supplied or dried using an Anhydrous Engineering alumina column drying system (dichloromethane, toluene, diethyl ether, and tetrahydrofuran). Reagents were purchased from commercial sources and used as received. All organolithium reagents were titrated against *N*-benzylbenzamide.[1]

Activation of $ZnCl_2$: Commercial $ZnCl_2$ (purchased from Sigma Aldrich) was placed in a flask and heated under vacuum until the solid fully melted to form a liquid. After cooling to room temperature, the flask was flushed with nitrogen to create an inert atmosphere and subsequently transferred to a glovebox for storage under Argon.

1.2. Instrumentation

Thin layer chromatography (TLC) was performed using Merck Kieselgel 60 F254 fluorescent treated silica, which was visualised under UV light, or by staining with aqueous basic potassium permanganate followed by heating, or Hanessian's stain (CAM stain) followed by heating, or *p*-anisaldehyde solution followed by heating, as stated.

Preparative Thin layer chromatography (PLC) was performed using Merck Z513032-1PAK TLC plates, Silica gel, which was visualised under UV light.

Flash column chromatography (FCC) was carried out using Sigma-Aldrich silica gel (60 Å, 230-400 mesh, 40-63 μm) or Thermo Scientific aluminum oxide (neutral for flash chromatography).

NMR spectra were recorded at various field strengths, as indicated, using Bruker 400 MHz,

Varian VNMR 400 MHz, Bruker Cryo 500 MHz or Bruker Cryo 600 MHz for ^{1}H, ^{11}B, and ^{13}C acquisitions. All NMR spectra were recorder at 25 °C unless otherwise stated. Chemical shifts (δ) are reported in parts per million (ppm) and referenced to $CDCl_3$ (^{1}H: 5.32 ppm; ^{13}C: 77.16 ppm). Coupling constants (J) are given in Hertz (Hz) and refer to apparent multiplicities (s = singlet, d = doublet, t = triplet, q = quartet, quin = quintet, hex = hextet, h = heptet, m = multiplet, brs = broad signal, dd = doublet of doublets, etc.). The ^{1}H NMR spectra are reported as follows: chemical shift (multiplicity, coupling constants, number of protons).

HPLC analyses were performed on Agilent 1100 system with Daicel Chiralpak columns.

High resolution mass spectra (HRMS) were recorded on a Bruker Daltonics MicrOTOF II by Electrospray Ionisation (ESI); a Thermo Scientific QExactive by Electron Ionisation (EI); a Thermo Scientific Orbitrap Elite by ESI or Atmospheric Pressure Chemical Ionisation (APCI); or a Bruker UltrafleXtreme by Matrix-assisted Laser Desorption/Ionisation (MALDI).

IR spectra were recorded neat as a thin film on a Perkin Elmer Spectrum One FT-IR. Selected absorption maxima (ν_{max}) are reported in wavenumbers (cm^{-1}).

Gas chromatography–mass spectrometry (**GC-MS**) was recorded on an Agilent 6890 Series GC and 5973 detectors using a HP-5MS UI column (15 m × 0.25 mm × 0.25 μm).

1.3. Naming of Compounds

Compound names are those generated by ChemDraw Professional 20.0 software (PerkinElmer), following the IUPAC nomenclature.

2. EXPERIMENTAL DATA

2.1. Reaction Optimization

Table S1: Reaction optimization of dynamic kinetic resolution (DKR) process.

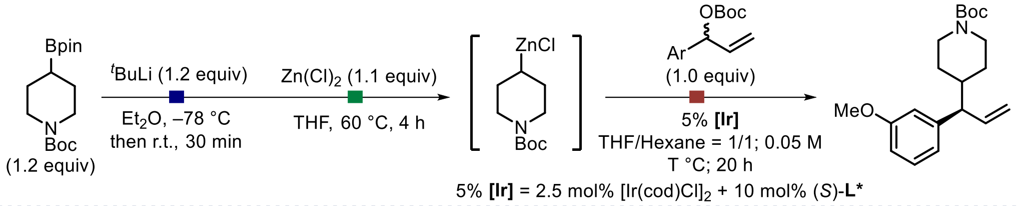

	alteration to conditions	yield[a]	er
1	room temperature	69%	85:15
2	60 °C	64%	93:7
3	80 °C	56%	94:6
4	110 °C	50%	96:4

a: NMR yield

Conclusion of DKR process (after > 50 reactions): 1) The use of a weaker nucleophile is crucial for DKR process, while secondary zinc reagents seem highly reactive nucleophile. 2) A higher reaction temperature is essential for the internal conversion of π-ally-Ir. However, a higher reaction temperature negatively impacts the stability of secondary zinc reagents.

Table S2: Reaction optimization.

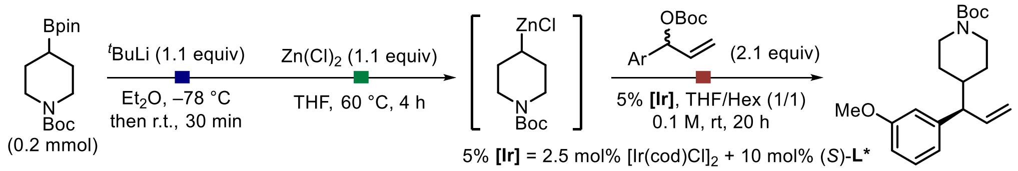

	alteration to conditions (green)	alteration to conditions (red)	yield[a]	er
1			57%	99:1
2[b]		THF/Hex (1/1); 10 °C	61%	99:1
3[b]	rt, 14 h	THF/Hex (1/1); 10 °C	59%	99:1
4[b]		THF/Hex (1/1); 10 °C; 1.5 equiv **a**	62% (54%)	98:2
5[b]	$Zn(Br)_2$ instead of $Zn(Cl)_2$	THF/Hex (1/1); 10 °C; 1.5 equiv **a**	46%	91:9
6[b]	$Zn(OAc)_2$ instead of $Zn(Cl)_2$	THF/Hex (1/1); 10 °C; 1.5 equiv **a**	trace	--
7[b]	$Zn(OTf)_2$ instead of $Zn(Cl)_2$	THF/Hex (1/1); 10 °C; 1.5 equiv **a**	no product	--

a: NMR yield; b: 0.4 mmol scale;

Figure S1: Boron-to-zinc transmetalation processes monitored by ^{11}B-NMR.

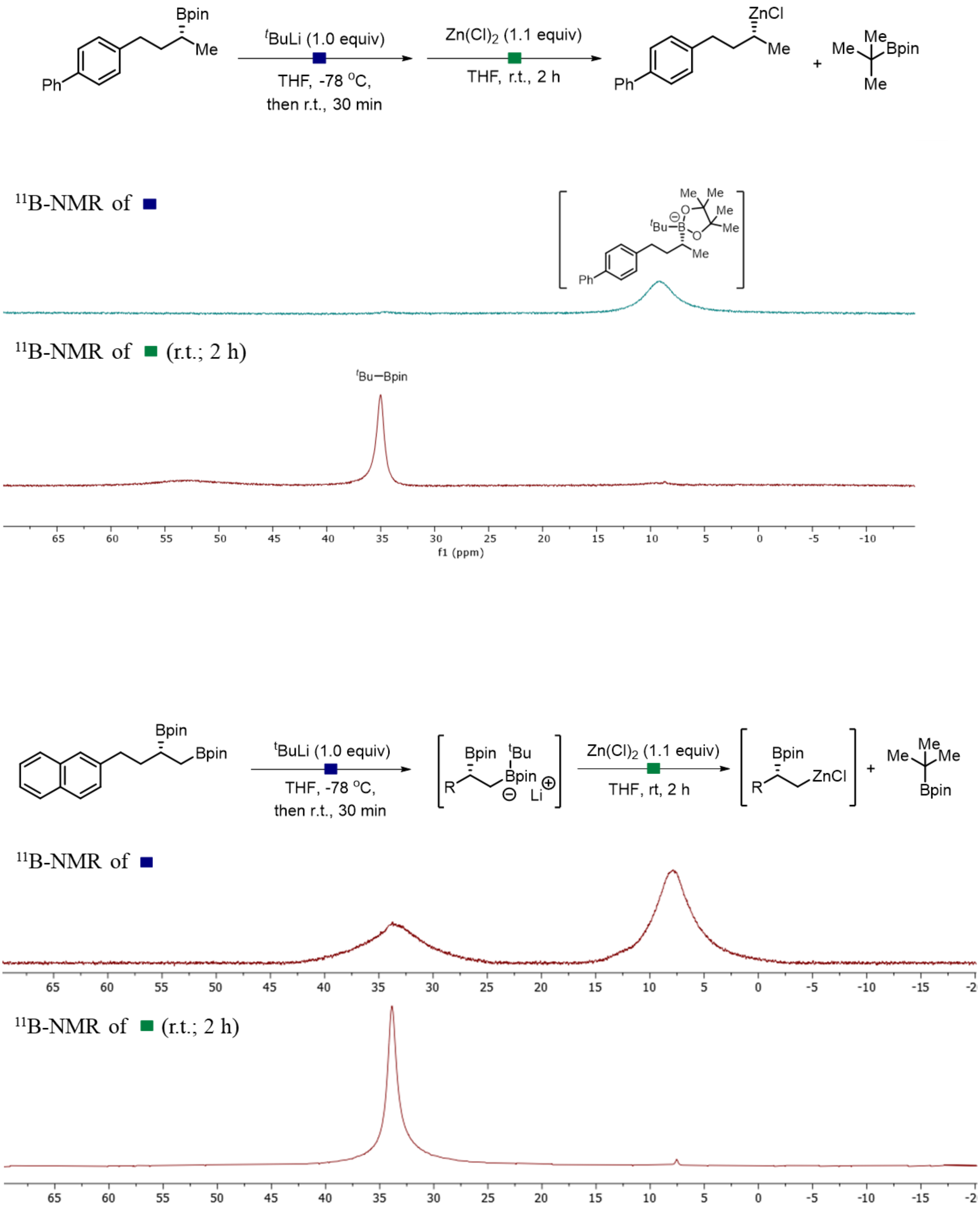

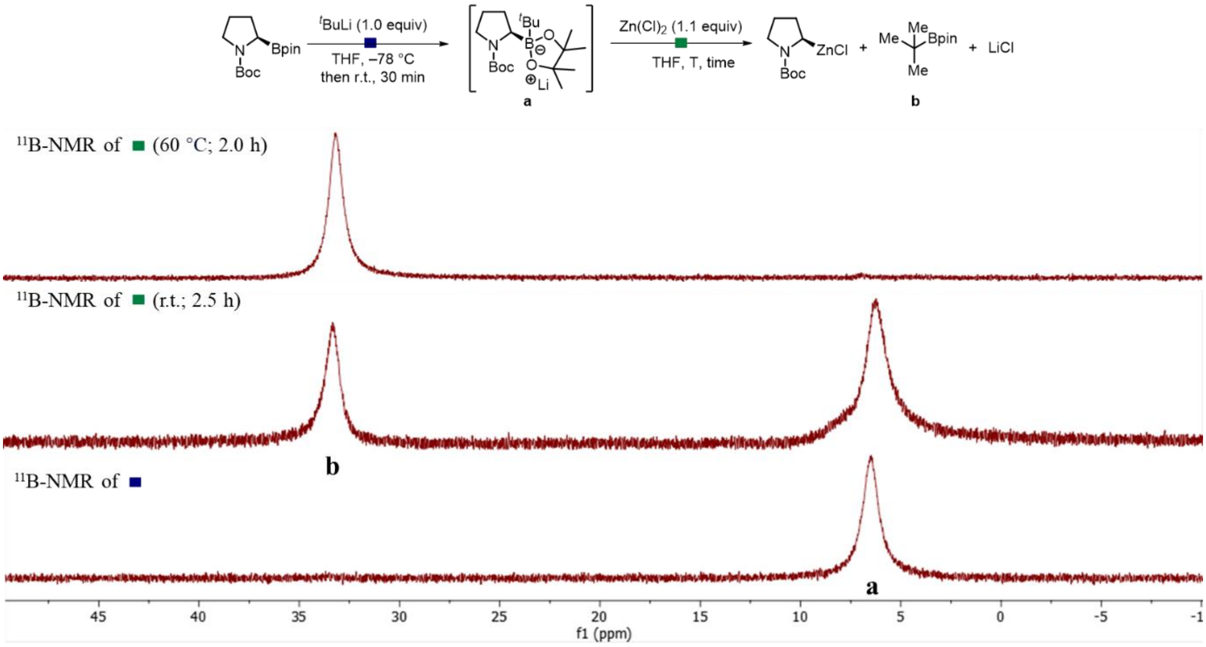

Using boron nuclear magnetic resonance (^{11}B-NMR) to observe the reaction mixture, we verified the successful transformation of the boronate complex to the organozinc reagent.

2.2. HPLC Analysis for Reaction Mechanism

Figure S2: Stereochemistry of product **44** from different routes.

The enantiomeric rate (e.r.) was determined after hydroboration/oxidation to alcohol. HPLC conditions: Chiral column IB, hexane: isopropanol = 97:3, flow rate = 1.0 mL/min, wavelength = 210 nm.

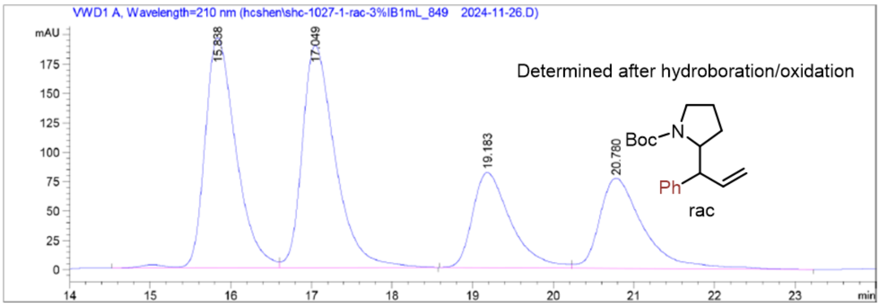

>> *rac*-product

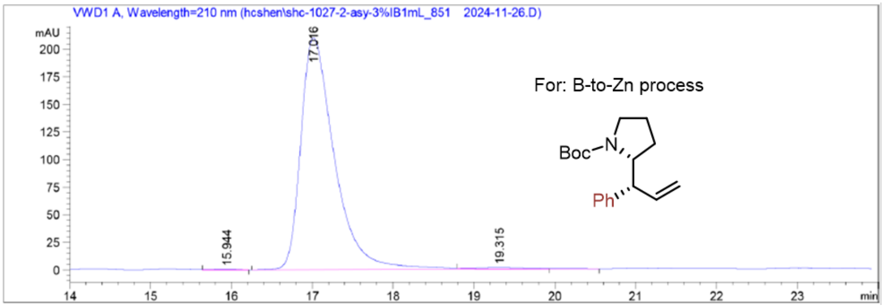

>> **44** from route a

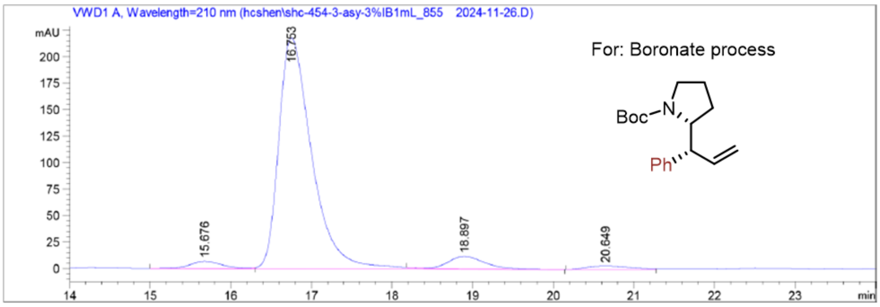

>> **44** from route b

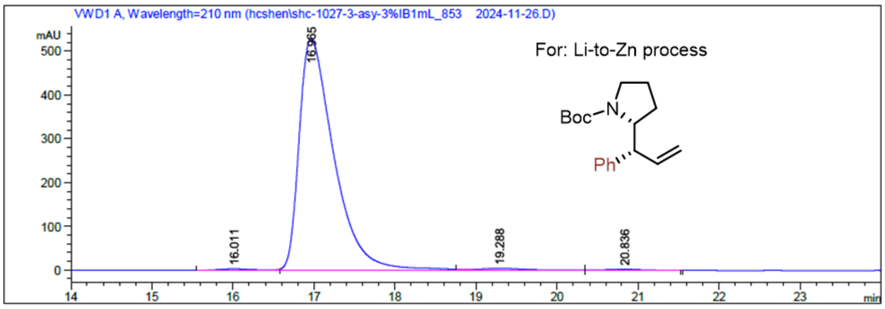

>> **44** from route c

All three routes depicted in Scheme 2b yield product **44** with the same conformation.

Figure S3: The erosion of stereospecificity in the reaction of enantioenriched organozinc reagents for product **30** and **31**.

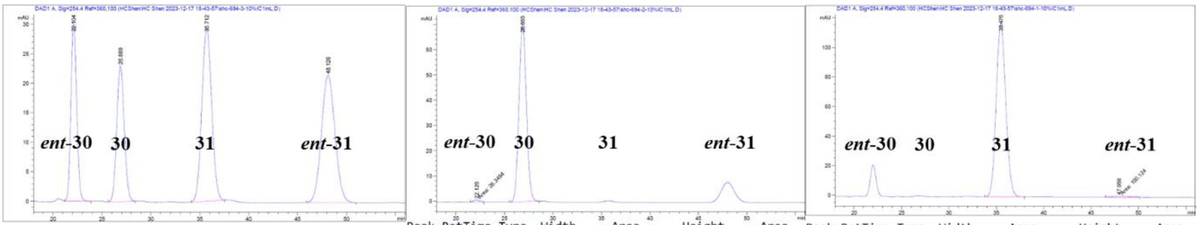

Peak #	RetTime [min]	Type	Width [min]	Area [mAU*s]	Height [mAU]	Area %
1	22.104	BB	0.6443	1246.16833	30.14376	18.8052
2	26.889	BB	0.8256	1229.31494	22.99871	18.5508
3	35.712	BB	1.0896	2065.84351	29.33322	31.1744
4	48.126	BB	1.4459	2085.40747	21.60919	31.4696

Peak #	RetTime [min]	Type	Width [min]	Area [mAU*s]	Height [mAU]	Area %
1	22.126	MM	0.6131	26.34935	7.16258e-1	0.6976
2	26.865	BB	0.8300	3751.01025	70.12582	99.3024

Peak #	RetTime [min]	Type	Width [min]	Area [mAU*s]	Height [mAU]	Area %
1	35.476	BB	1.1012	8275.91602	117.28199	98.8046
2	47.956	MM	2.0778	100.12354	8.03127e-1	1.1954

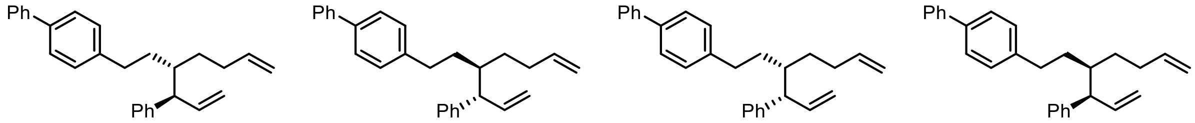

30 **_ent_-30** **31** **_ent_-31**

The enantiomeric rate (e.r.) was determined after hydroboration/oxidation to alcohol. The HPLC analysis of *rac*-**30** + *rac*-**31**, **30**, and **31** is presented here. Compound **30** was observed alongside ***ent*-31** as the minor diastereomer, while **31** was observed with ***ent*-30** as the minor diastereomer. This indicates that the lower diastereoselectivities obtained for **30** and **31** result from partial erosion of the stereospecificity at the organozinc stereocenter, with inversion efficiency falling below 100%.

Figure S4: HPLC analysis of recovered (*R*)-**2a**.

HPLC conditions: Chiral column IC, hexane: isopropanol = 99:1, flow rate = 0.8 mL/min, wavelength = 210 nm.

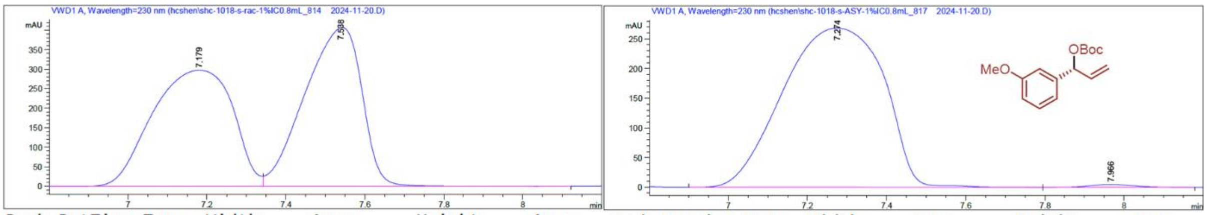

Peak #	RetTime [min]	Type	Width [min]	Area [mAU*s]	Height [mAU]	Area %
1	7.179	BV	0.2408	4211.65869	297.63480	50.6875
2	7.538	VV R	0.1673	4097.40332	405.89096	49.3125

Peak #	RetTime [min]	Type	Width [min]	Area [mAU*s]	Height [mAU]	Area %
1	7.274	BV	0.2973	4766.70996	268.60510	99.2009
2	7.966	VB	0.1208	38.39575	4.88684	0.7991

Figure S5: GC-MS analysis of model reaction mixture that employing Zn(OAc)$_2$ and Zn(OPiv)$_2$ as zinc salts.

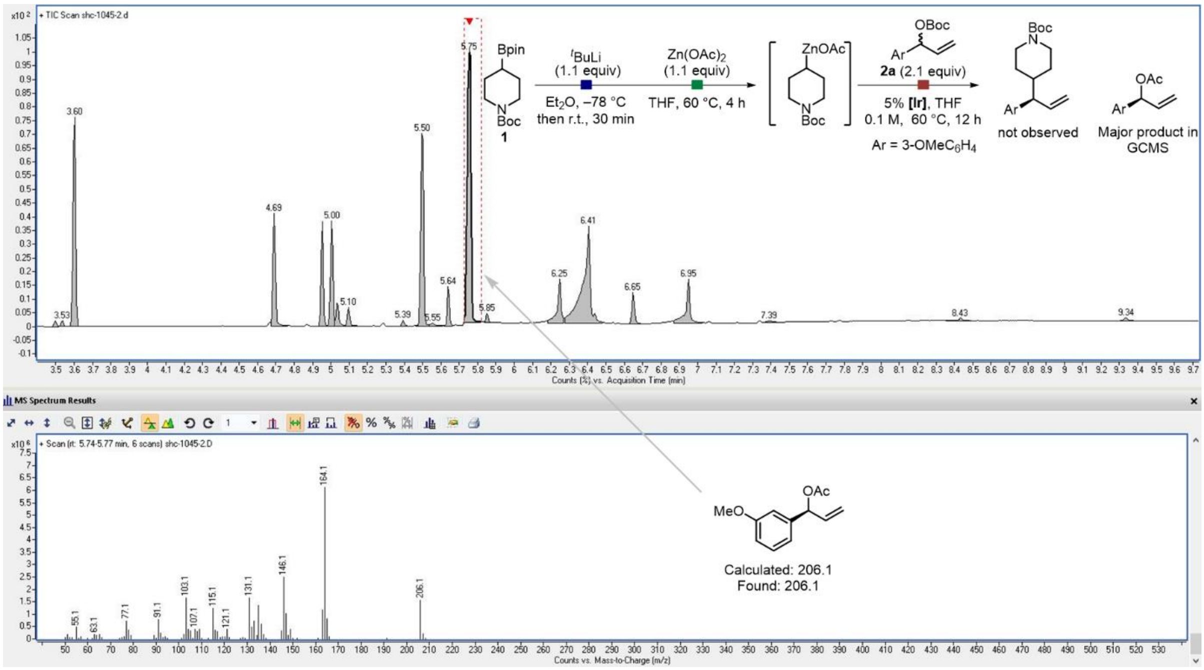

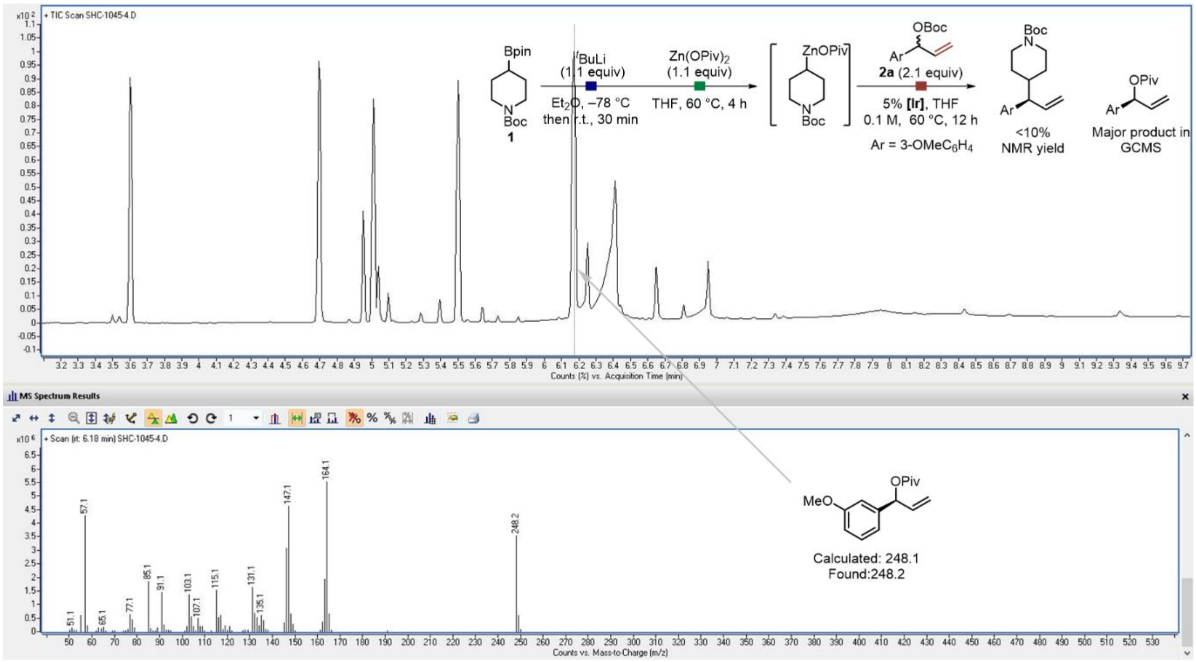

2.3. General Procedures

2.3.1. General Procedure A for product 4, 7-25.

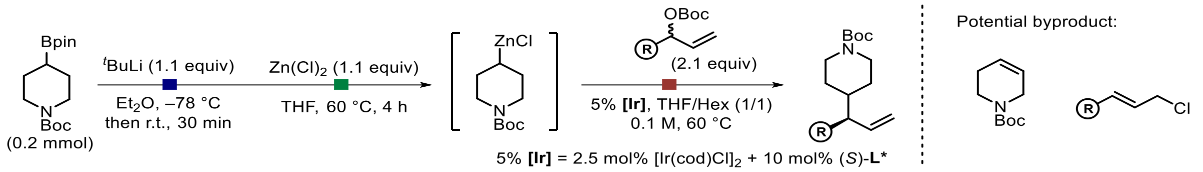

Step 1 boronate complex generation: An oven-dried 25 mL Schlenk tube equipped with a magnetic stir bar was purged with nitrogen (N_2) three times. Boronic ester (0.2 mmol, 1.0 equiv) and anhydrous diethyl ether (Et_2O, 2 mL) were added under nitrogen. The solution was cooled to −78 °C using a dry ice/acetone bath. A solution of ${}^tBuLi$ (0.22 mmol, 1.1 equiv) was added dropwise while maintaining the temperature at −78 °C. The mixture was stirred for 5 minutes at this temperature and then warmed to ambient temperature, where it was stirred for an additional 30 minutes to ensure complete formation of the boronate complex. The solvent was removed under high vacuum, and the reaction vessel was refilled with nitrogen. **Step 2 boron-to-zinc transmetalation:** The reaction tube was transferred to glovebox, where $ZnCl_2$ (28.6 mg, 0.22 mmol, 1.1 equiv) and anhydrous THF (0.5 mL) were added to the reaction. After removal from the glovebox, the reaction mixture was stirred at 60 °C for 4 hours to complete the transmetalation process. And then cool to ambient temperature. **Step 3 asymmetric allylation:** Inside the glovebox, an oven-dried 7.0 mL vial equipped with a magnetic stir bar was charged with $[Ir(COD)Cl]_2$ (0.005 mmol, 2.5 mol%, 3.4 mg) and (*S*)-**L1** (0.02 mmol, 10 mol%, 10.1 mg). The vial was sealed with a subar seal, removed from the glovebox, and anhydrous THF (0.3 mL) was added under nitrogen. The mixture was stirred at ambient temperature for 15–30 minutes to allow the formation of the active catalyst complex. Allylic carbonate (0.42 mmol, 2.1 equiv) was added to the mixture, forming a π-allyl iridium complex. The π-allyl iridium solution was added to the organozinc reagent prepared in Step 2. The reaction vial was rinsed with 0.2 mL anhydrous THF and 1.0 mL hexane, and the rinsings were added to the reaction mixture in the Schlenk tube. The reaction was stirred at ambient temperature or 60 °C for 12 hours. The crude reaction mixture was filtered through a silica gel pad, wash with Et_2O (3 times/10 mL) and the filtrate was concentrated under reduced pressure.

The resulting crude material was purified by flash column chromatography or preparative thin-layer chromatography (PLC) to isolate the desired product. **Note**: 1) After **Step 3**, 1,3,5-trimethoxybenzene was added as an internal standard, and the NMR yield was determined. 2) Two byproducts were observed in certain cases, which may be challenging to separate from the product. Preparative HPLC may be required for final purification.

2.3.2. General Procedure B for product 27-28.

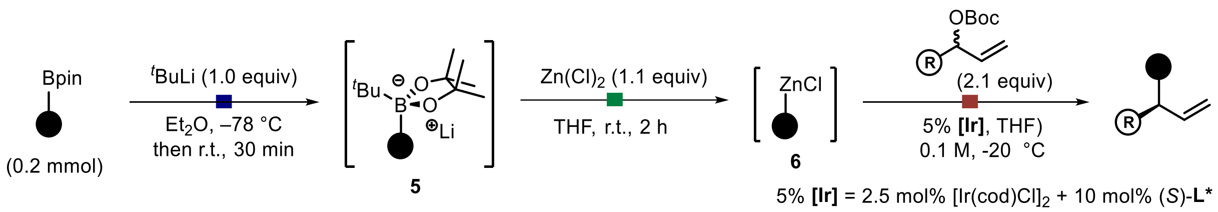

Step 1 boronate complex generation: An oven-dried 25 mL Schlenk tube equipped with a magnetic stir bar was purged with nitrogen (N_2) three times. Boronic ester **26** (0.2 mmol, 1.0 equiv) and anhydrous diethyl ether (Et_2O, 2 mL) were added under nitrogen. The solution was cooled to −78 °C using a dry ice/acetone bath. A solution of <u>^{t}BuLi (0.20 mmol, 1.0 equiv)</u> was added dropwise while maintaining the temperature at −78 °C. The mixture was stirred for 5 minutes at this temperature and then warmed to ambient temperature, where it was stirred for an additional 30 minutes to ensure complete formation of the boronate complex. The solvent was removed under high vacuum, and the reaction vessel was refilled with nitrogen. **Step 2 boron-to-zinc transmetalation**: The reaction mixture was transferred to glovebox, where $ZnCl_2$ (28.6 mg, 0.22 mmol, 1.1 equiv) and anhydrous <u>THF (1 mL)</u> were added to the reaction mixture. After removal from the glovebox, the reaction mixture was stirred at <u>ambient temperature for 2 hours</u> to complete the transmetalation process. **Step 3 asymmetric allylation**: Inside the glovebox, an oven-dried 7.0 mL vial equipped with a magnetic stir bar was charged with $[Ir(COD)Cl]_2$ (0.005 mmol, 2.5 mol%, 3.4 mg) and (*S*)-**L1** (0.02 mmol, 10 mol%, 10.1 mg). The vial was sealed with a subar seal, removed from the glovebox, and anhydrous <u>THF (0.5 mL)</u> was added under nitrogen. The mixture was stirred at ambient temperature for 15–30 minutes to allow the formation of the active catalyst complex. Allylic carbonate (0.42 mmol, 2.1 equiv) was added to the mixture, forming a π-allyl iridium complex. The π-allyl iridium

solution was added to the organozinc reagent prepared in Step 2. The reaction vial was rinsed with 0.5 mL anhydrous THF, and the rinsings were added to the reaction mixture in the Schlenk tube. The reaction was stirred at -20 °C for 24 hours. The crude reaction mixture was filtered through a silica gel pad, wash with Et_2O (3 times/10 mL) and the filtrate was concentrated under reduced pressure. The resulting crude material was purified by flash column chromatography to isolate the desired product.

2.3.3. General Procedure C for product 30-31, 33-34.

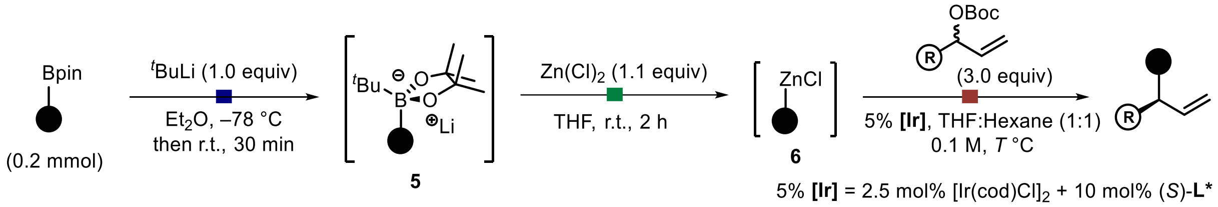

Step 1 boronate complex generation: An oven-dried 25 mL Schlenk tube equipped with a magnetic stir bar was purged with nitrogen (N_2) three times. Boronic ester **29** or **32** (0.2 mmol, 1.0 equiv) and anhydrous diethyl ether (Et_2O, 2 mL) were added under nitrogen. The solution was cooled to −78 °C using a dry ice/acetone bath. A solution of ^{t}BuLi (0.20 mmol, 1.0 equiv) was added dropwise while maintaining the temperature at −78 °C. The mixture was stirred for 5 minutes at this temperature and then warmed to ambient temperature, where it was stirred for an additional 30 minutes to ensure complete formation of the boronate complex. The solvent was removed under high vacuum, and the reaction vessel was refilled with nitrogen. **Step 2 boron-to-zinc transmetalation**: The reaction mixture was transferred to glovebox, where $ZnCl_2$ (28.6 mg, 0.22 mmol, 1.1 equiv) and anhydrous THF (1 mL) were added to the reaction mixture. After removal from the glovebox, the reaction mixture was stirred at ambient temperature for 2 hours (For boronic ester **32**, 60 °C for 7 hours) to complete the transmetalation process. **Step 3 asymmetric allylation**: Inside the glovebox, an oven-dried 7.0 mL vial equipped with a magnetic stir bar was charged with $[Ir(COD)Cl]_2$ (0.01 mmol, 5 mol%, 6.8 mg) and (*S*)-**L1** (0.04 mmol, 20 mol%, 20.2 mg). The vial was sealed with a subar seal, removed from the glovebox, and anhydrous THF (0.5 mL) was added under nitrogen. The mixture was stirred at ambient temperature for 15–30 minutes to allow the formation of the

active catalyst complex. Allylic carbonate (0.60 mmol, 3.0 equiv) was added to the mixture, forming a π-allyl iridium complex. The π-allyl iridium solution was added to the organozinc reagent prepared in Step 2. The reaction vial was rinsed with 0.5 mL anhydrous THF, and the rinsings were added to the reaction mixture in the Schlenk tube. The reaction was stirred at 10 °C or r.t. for 24 hours (For boronic ester **32**, r.t. for 24 hours). The crude reaction mixture was filtered through a silica gel pad, wash with Et_2O (3 times/10 mL) and the filtrate was concentrated under reduced pressure. The resulting crude material was purified by flash column chromatography to isolate the desired product.

2.3.4. General Procedure D for product 36-39

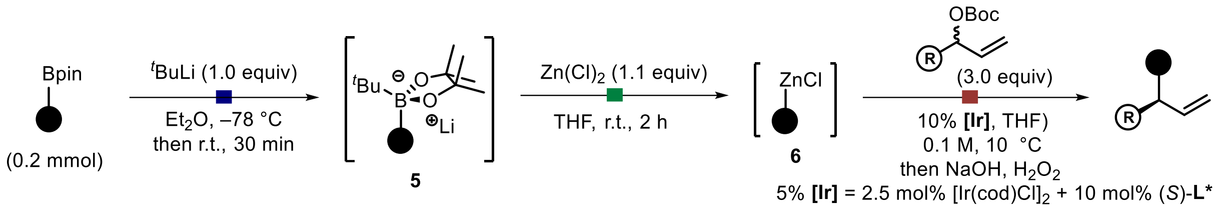

Step 1 boronate complex generation: An oven-dried 25 mL Schlenk tube equipped with a magnetic stir bar was purged with nitrogen (N_2) three times. Boronic ester **35** (0.2 mmol, 1.0 equiv) and anhydrous diethyl ether (Et_2O, 2 mL) were added under nitrogen. The solution was cooled to −78 °C using a dry ice/acetone bath. A solution of ^{t}BuLi (0.20 mmol, 1.0 equiv) was added dropwise while maintaining the temperature at −78 °C. The mixture was stirred for 5 minutes at this temperature and then warmed to ambient temperature, where it was stirred for an additional 30 minutes to ensure complete formation of the boronate complex. The solvent was removed under high vacuum, and the reaction vessel was refilled with nitrogen. **Step 2 boron-to-zinc transmetalation**: The reaction mixture was transferred to glovebox, where $ZnCl_2$ (28.6 mg, 0.22 mmol, 1.1 equiv) and anhydrous THF (0.5 mL) were added to the residue. After removal from the glovebox, the reaction mixture was stirred at ambient temperature for 2 hours to complete the transmetalation process. **Step 3 asymmetric allylation**: Inside the glovebox, an oven-dried 7.0 mL vial equipped with a magnetic stir bar was charged with $[Ir(COD)Cl]_2$ (0.01 mmol, 5 mol%, 6.8 mg) and (*S*)-**L1** (0.04 mmol, 20 mol%, 20.2 mg). The vial was sealed with a septum, removed from the glovebox, and anhydrous THF (0.3 mL) was

added under nitrogen. The mixture was stirred at ambient temperature for 15–30 minutes to allow the formation of the active catalyst complex. Allylic carbonate (0.60 mmol, 3.0 equiv) was added to the mixture, forming a π-allyl iridium complex. The π-allyl iridium solution was added to the organozinc reagent prepared in **Step 2**. The reaction vial was rinsed with 0.2 mL anhydrous THF and 1 mL anhydrous hexane, and the rinsings were added to the reaction mixture in the Schlenk tube. The reaction was stirred at ambient temperature for 24 hours. The crude reaction mixture was filtered through a silica gel pad, wash with Et_2O (3 times/10 mL) and the filtrate was concentrated under reduced pressure. The resulting crude material was dissolved in 2 mL THF. The reaction was cooled to 0 °C, then 3 N aqueous NaOH (1 mL) and 30% aqueous H_2O_2 (0.6 mL) were added. The reaction was allowed to warm to room temperature and stirring continued for 1 h before quenching with saturated $Na_2S_2O_3$ (2 mL) at 0 °C and extracting with Et_2O (5 mL) three times. The organic layer was dried over $MgSO_4$, filtered, and concentrated under reduced pressure. The crude mixture was purified by flash column chromatography to isolate the desired product.

2.3.5. Procedures for stereochemistry analysis of brornate complex vs organozinc reagents.

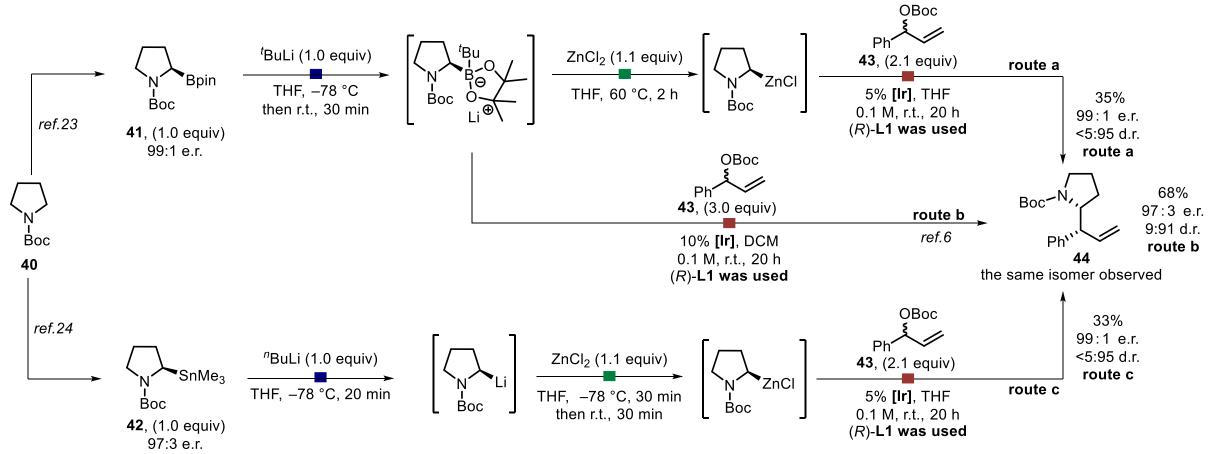

Route a: Following **General Procedure A**, **Step 2** was conducted at 60 °C for 2 hours, and **Step 3** was carried out at room temperature for 20 hours. The resulting crude material was preliminarily isolated via flash column chromatography (ethyl acetate/hexane, 20:1) for NMR analysis to assess the diastereoselectivity. Subsequently, the desired product **44** was isolated

using preparative thin-layer chromatography (PLC) with a solvent system of toluene/ethyl acetate (10:1), yielding 20.1 mg (35% yield, 99:1 e.r., <5:95 d.r.).

Route b: This is results of our previous published work.[2]

Route c: **Step 1 organolithium generation**: An oven-dried 25 mL Schlenk tube equipped with a magnetic stir bar was purged with nitrogen (N_2) three times. Organotin compound **42** (60 μL, 0.2 mmol, 1.0 equiv) and anhydrous THF (0.78 mL) were added under nitrogen. The solution was cooled to −78 °C using a dry ice/acetone bath. A solution of ^{n}BuLi (0.20 mmol, 1.0 equiv) was added dropwise while maintaining the temperature at −78 °C. The mixture was stirred for 20 minutes at this temperature. **Step 2 lithium-to-zinc transmetalation**: The solution of $ZnCl_2$ (0.22 mL, 1M in anhydrous THF) were added dropwise to the residue. The reaction mixture was stirred at this temperature for 30 minutes, then ambient temperature 30 minutes to complete the transmetalation process. **Step 3 asymmetric allylation**: Inside the glovebox, an oven-dried 7.0 mL vial equipped with a magnetic stir bar was charged with $[Ir(COD)Cl]_2$ (0.01 mmol, 2.5 mol%, 3.4 mg) and (*S*)-**L1** (0.02 mmol, 10 mol%, 10.1 mg). The vial was sealed with a subar seal, removed from the glovebox, and anhydrous THF (0.5 mL) was added under nitrogen. The mixture was stirred at ambient temperature for 15–30 minutes to allow the formation of the active catalyst complex. Allylic carbonate (0.42 mmol, 2.1 equiv) was added to the mixture, forming a π-allyl iridium complex. The π-allyl iridium solution was added to the organozinc reagent prepared in Step 2. The reaction vial was rinsed with 0.5 mL anhydrous THF, and the rinsings were added to the reaction mixture in the Schlenk tube. The reaction was stirred at ambient temperature for 24 hours. The crude reaction mixture was filtered through a silica gel pad, wash with Et_2O (3 times/10 mL) and the filtrate was concentrated under reduced pressure. The resulting crude material was preliminarily isolated via flash column chromatography (ethyl acetate/hexane, 20:1) for NMR analysis to assess the diastereoselectivity. Subsequently, the desired product **44** was isolated using preparative thin-layer chromatography (PLC) with a solvent system of toluene/ethyl acetate (10:1), yielding 19.2 mg (33% yield, 99:1 e.r., <5:95 d.r.).

2.4. Synthesis of Starting Materials

2.4.1. Synthesis of racemic allylic carbonates

All racemic allylic carbonates were synthesized according to relevant literatures.[3]

2.4.2. Characterization data for boronic esters

(*R*)-2-(4-([1,1'-biphenyl]-4-yl)butan-2-yl)-4,4,5,5-tetramethyl-1,3,2-dioxaborolane (26)

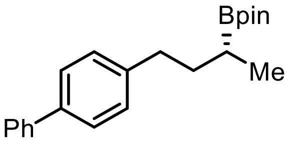

Prepared according to the referenced literature procedure.[4] ^{1}**H NMR** (400 MHz, $CDCl_3$) δ 7.61 – 7.55 (m, 2H), 7.54 – 7.46 (m, 2H), 7.47 – 7.37 (m, 2H), 7.36 – 7.27 (m, 1H), 7.28 – 7.25 (m, 2H), 2.61 – 2.72 (m, 2H), 1.78 – 1.87 (m, 1H), 1.58-1.67 (m, 1H), 1.26 (s, 12H), 1.07 – 1.14 (m, 1H), 1.03 – 1.05 (m, 3H). ^{13}**C NMR** (126 MHz, $CDCl_3$) δ 142.4, 141.4, 138.7, 129.0, 128.8, 127.1(4), 127.1(3), 127.0(5), 83.1, 35.4, 35.1, 25.0, 24.9, 15.6. **Specific rotation** $[\alpha]_D^{21}$ = −14 (c = 0.29, CH_2Cl_2). **HRMS** (EI) m/z calculated for $C_{20}H_{25}BO_2$ $[M]^+$, 309.2020; found, 309.2031. **IR (neat)** 3005, 1459, 1276, 764 cm^{-1}. The enantiomeric excess (ee) was determined after oxidation to alcohol. **HPLC conditions**: Chiral column IB, hexane: isopropanol = 95:5, flow rate = 1.0 mL/min, wavelength = 254 nm, t_R = 22.1 min for major isomer, t_R = 21.5 min for minor isomer.

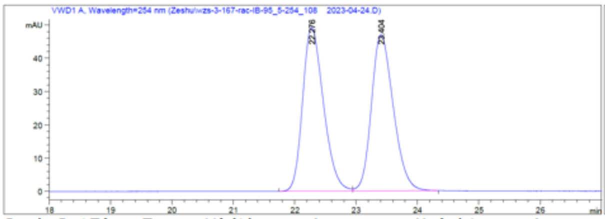

Peak #	RetTime [min]	Type	Width [min]	Area [mAU*s]	Height [mAU]	Area %
1	22.276	VV R	0.3432	1159.80981	49.17299	49.9585
2	23.404	VB	0.3549	1161.73755	46.90362	50.0415

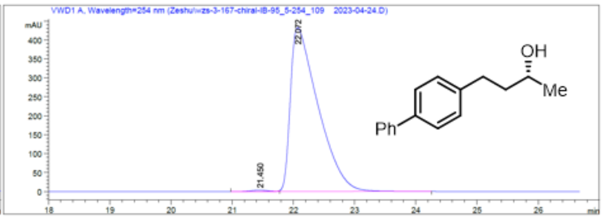

Peak #	RetTime [min]	Type	Width [min]	Area [mAU*s]	Height [mAU]	Area %
1	21.450	BV E	0.2357	69.15667	3.60074	0.5068
2	22.072	VV R	0.4314	1.35768e4	432.50787	99.4932

(*R*)-2-(1-([1,1'-biphenyl]-4-yl)hept-6-en-3-yl)-4,4,5,5-tetramethyl-1,3,2-dioxaborolane (29)

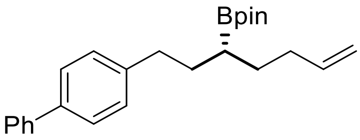

The compound **29** was synthesized according to Morken's asymmetric diboration followed by allylation chemistry.[4] **^{1}H NMR** (400 MHz, $CDCl_3$) δ 7.59-7.56 (m, 2H), 7.52 – 7.49 (m, 2H), 7.47 – 7.40 (m, 2H), 7.34 – 7.30 (m, 1H), 7.26 (d, J = 7.9 Hz, 2H), 5.87 – 5.77 (m, 1H), 5.03 – 4.91 (m, 2H), 2.73 – 2.56 (m, 2H), 2.17 – 1.99 (m, 2H), 1.86 – 1.65 (m, 2H), 1.64 – 1.46 (m, 2H), 1.28 (s, 12H), 1.16 – 1.06 (m, 1H) ppm. **^{13}C NMR** (126 MHz, $CDCl_3$) δ 142.3, 141.4, 139.3, 138.7, 129.0, 128.8, 127.1(6), 127.1(5), 127.1, 114.5, 83.2, 35.3, 33.5(4), 33.4(6), 30.7, 25.0(3), 25.0(0) ppm. **Specific rotation** $[\alpha]_D^{22}$ = −10 (c = 0.83, CH_2Cl_2). **HRMS** (ESI) m/z calculated for $C_{25}H_{33}BO_2$ $[M+H]^+$, 377.2646, found: 377.2642. **IR (neat)** 2976, 2924, 2855, 1640, 1486, 1379, 1315, 1234, 1142, 966, 909, 697 cm^{-1}. The enantiomeric excess (ee) was determined after oxidation to alcohol. **HPLC conditions**: Chiral column IB, hexane: isopropanol = 90:10, flow rate = 1.0 mL/min, wavelength = 254 nm, t_R = 19.9 min for major isomer, t_R = 19.2 min for minor isomer.

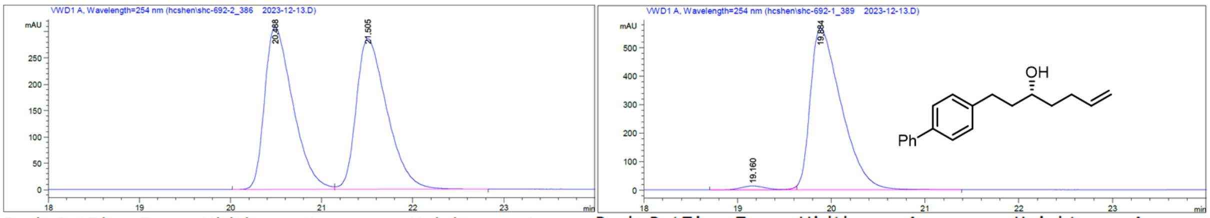

Peak #	RetTime [min]	Type	Width [min]	Area [mAU*s]	Height [mAU]	Area %
1	20.488	BV	0.2880	6632.18701	307.56192	49.7124
2	21.505	VV R	0.2970	6708.92969	285.94476	50.2876

Peak #	RetTime [min]	Type	Width [min]	Area [mAU*s]	Height [mAU]	Area %
1	19.160	BV E	0.2399	248.12032	13.50112	1.8706
2	19.884	VV R	0.3046	1.30164e4	567.03967	98.1294

(*R*)-2,2'-(4-([1,1'-biphenyl]-4-yl)butane-1,2-diyl)bis(4,4,5,5-tetramethyl-1,3,2-dioxaborolane) (35)

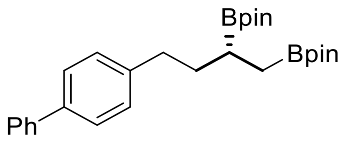

The compound **35** was synthesized according to Morken's asymmetric diboration chemistry.[4] **^{1}H NMR** (500 MHz, $CDCl_3$) δ 7.58-7.56 (m, 2H), 7.51 – 7.47 (m, 2H), 7.44 – 7.39 (m, 2H), 7.34 – 7.29 (m, 1H), 7.26 (d, *J* = 7.9 Hz, 2H), 2.66 (t, *J* = 8.2 Hz, 2H), 1.88 – 1.78 (m, 1H), 1.72 – 1.62 (m, 1H), 1.28 – 1.19 (m, 25H), 0.99 – 0.87 (m, 2H) ppm. **^{13}C NMR** (126 MHz, $CDCl_3$) δ 142.6, 141.5, 138.6, 129.0, 128.8, 127.2, 127.1, 127.0, 83.0(6), 83.0(5), 36.0, 35.1, 25.0(8), 25.0(5), 25.0, 24.9 ppm. **Specific rotation** $[\alpha]_D^{22}$ = −13 (c = 1.7, CH_2Cl_2). **HRMS** (ESI) m/z calculated for $C_{28}H_{40}B_2O_4$ $[M+H]^+$, 463.3185, found: 463.3175. **IR (neat)** 2977, 2926, 1486, 1370, 1312, 1214, 1141, 968, 845, 698 cm^{-1}. The enantiomeric excess (ee) was determined after protodeboronation/oxidation to alcohol (**26**).

2.4.3. Characterization data for 42

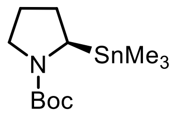

The compound **42** was synthesized using (+)-Sparteine according to the referenced literature procedure.[5] **^{1}H NMR** (400 MHz, $CDCl_3$) δ 3.63 – 3.54 (m, 0.3 H), 3.47 – 3.17 (m, 2.7H), 2.29 – 1.65 (m, 4H), 1.44 (s, 2.7H), 1.44 (s, 6.3H), 0.09 (s, 2.7H), 0.07 (s, 6.3H). ^{1}H NMR data in accordance with the literature.[5] The e.r. of **42** was determined by the following procedure: An oven-dried 25 mL Schlenk tube equipped with a magnetic stir bar was purged with nitrogen (N_2) three times. Organotin compound **42** (60 μL, 0.2 mmol, 1.0 equiv) and anhydrous THF (1 mL) were added under nitrogen. The solution was cooled to −78 °C using a dry ice/acetone bath. A solution of ^{n}BuLi (0.20 mmol, 1.0 equiv) was added dropwise while maintaining the temperature at −78 °C. The mixture was stirred for 20 minutes at this temperature. Inside the glovebox, an oven-dried 7.0 mL vial equipped with a magnetic stir bar was charged with Benzophenone (0.24 mmol, 43.7 mg, 1.2 equiv), and anhydrous THF (0.5 mL) was added under

nitrogen. The Benzophenone solution was added to the organolithium reagent prepared at −78 °C. The reaction vial was rinsed with 0.5 mL anhydrous THF, and the rinsings were added to the reaction mixture in the Schlenk tube. The reaction was stirred at −78 °C for 30 minutes then at ambient temperature overnight. The crude reaction mixture was filtered through a silica gel pad, wash with Et_2O (3 times/10 mL) and the filtrate was concentrated under reduced pressure. The resulting crude material was preliminarily isolated via flash column chromatography (ethyl acetate/hexane/DCM, 5/1/1) to give **S1** (51.6 mg, 92%) for HPLC analysis.

(*S*)-1,1-diphenyltetrahydro-1H,3H-pyrrolo[1,2-c]oxazol-3-one (S1)

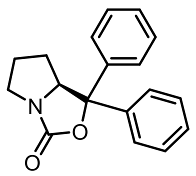

^{1}H NMR (500 MHz, $CDCl_3$) δ 7.67 – 7.61 (m, 2H), 7.53 – 7.38 (m, 8H), 4.67 (dd, J = 10.5, 5.5 Hz, 1H), 3.85 (dt, J = 11.4, 8.1 Hz, 1H), 3.37 (ddd, J = 11.4, 9.6, 3.7 Hz, 1H), 2.15 – 2.04 (m, 1H), 1.98 (dddt, J = 13.1, 11.1, 9.5, 7.4 Hz, 1H), 1.90 – 1.80 (m, 1H), 1.25 (dtd, J = 12.6, 10.8, 8.7 Hz, 1H) ppm. **^{13}C NMR** (126 MHz, $CDCl_3$) δ 160.5, 143.4, 140.4, 128.7, 128.42, 128.40, 127.8, 126.1, 125.6, 86.0, 69.3, 46.1, 29.1, 25.0 ppm. **Specific rotation** $[\alpha]_D^{24}$ = −172 (c = 1.1, CH_2Cl_2). **HRMS** (EI) m/z calculated for $C_{18}H_{17}O_2N$ $[M]^+$, 279.1254, found: 279.1249. **IR (neat)** 2974, 1751, 1449, 1227, 1003, 763, 701 cm^{-1}. **HPLC conditions**: Chiral column IB, hexane: isopropanol = 90:10, flow rate = 1.0 mL/min, wavelength = 230 nm, t_R = 16.0 min for major isomer, t_R = 22.5 min for minor isomer.

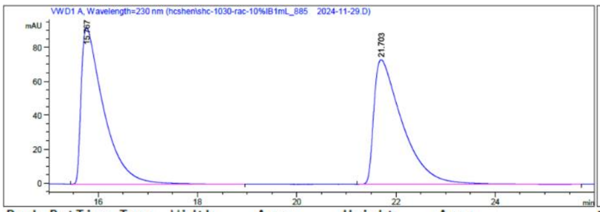

Peak #	RetTime [min]	Type	Width [min]	Area [mAU*s]	Height [mAU]	Area %
1	15.767	VB	0.4586	2906.58350	92.06866	49.7861
2	21.703	BB	0.5855	2931.55566	73.36939	50.2139

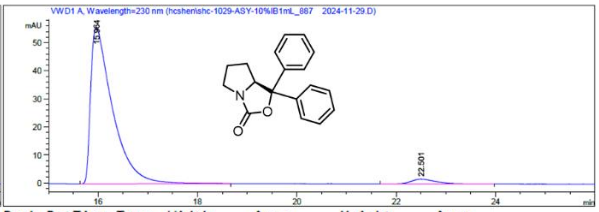

Peak #	RetTime [min]	Type	Width [min]	Area [mAU*s]	Height [mAU]	Area %
1	15.964	BB	0.4442	1702.77380	55.67255	96.6695
2	22.501	BB	0.5193	58.66486	1.67494	3.3305

2.5. Procedures for Transformation of Products

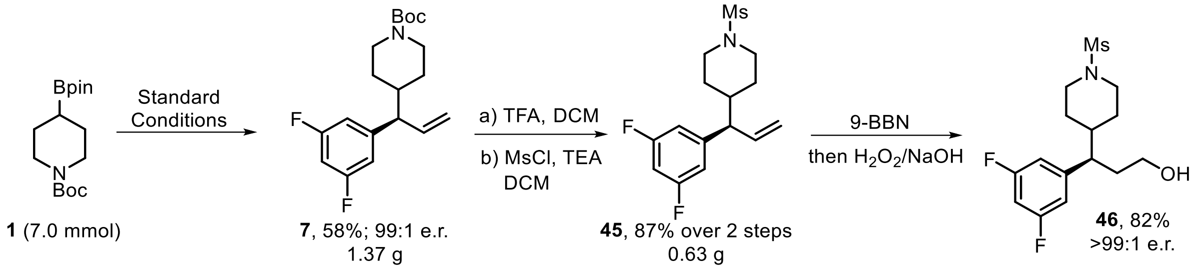

Scare-up: **Step 1 boronate complex generation:** An oven-dried 250 mL Schlenk tube equipped with a magnetic stir bar was purged with nitrogen (N_2) three times. Boronic ester (2.18 g, 7.0 mmol, 1.0 equiv) and anhydrous diethyl ether (Et_2O, 70 mL) were added under nitrogen. The solution was cooled to −78 °C using a dry ice/acetone bath. A solution of ^{t}BuLi (4.5 mL, 7.7 mmol, 1.1 equiv) was added dropwise while maintaining the temperature at −78 °C. The mixture was stirred for 5 minutes at this temperature and then warmed to ambient temperature, where it was stirred for an additional 30 minutes to ensure complete formation of the boronate complex. The solvent was removed under high vacuum, and the reaction mixture was refilled with nitrogen. **Step 2 boron-to-zinc transmetalation:** The reaction mixture was transferred to glovebox, where $ZnCl_2$ (1.05 g, 7.7 mmol, 1.1 equiv) and anhydrous THF (20 mL) were added to the residue. After removal from the glovebox, the reaction mixture was stirred at 60 °C for 4 hours to complete the transmetalation process. And then cool to ambient temperature. **Step 3 asymmetric allylation:** Inside the glovebox, an oven-dried 25 mL vial equipped with a magnetic stir bar was charged with $[Ir(COD)Cl]_2$ (0.175 mmol, 2.5 mol%, 119 mg) and (*S*)-**L1** (0.7 mmol, 10 mol%, 353.5 mg). The vial was sealed with a seal, removed from the glovebox, and anhydrous THF (10 mL) was added under nitrogen. The mixture was stirred at ambient temperature for 15 minutes to allow the formation of the active catalyst complex. Allylic carbonate (3.97g, 14.7 mmol, 2.1 equiv) was added to the mixture, forming a π-allyl iridium complex. The π-allyl iridium solution was added to the organozinc reagent prepared in Step 2. The reaction vial was rinsed with 5 mL anhydrous THF and 35 mL hexane, and the rinsings were added to the reaction mixture in the Schlenk tube. The reaction was stirred at ambient temperature or 60 °C for 12 hours. The crude reaction mixture was filtered through a silica gel pad, wash with Et_2O (3 times/10 mL) and the filtrate was concentrated under

reduced pressure. The resulting crude material was purified by flash column chromatography (Hexane/Ethyl acetate/DCM = 10/1/1), yielding **7** (1.37g, 58%, 99:1 e.r.).

Procedure for the synthesis of 45: An oven-dried 25 mL flask equipped with a magnetic stir bar was purged with nitrogen (N_2) three times. Compound **7** (0.78 g, 2.3 mmol, 1.0 equiv) and anhydrous DCM (5 mL) were added under nitrogen. The solution was cooled to 0 °C using an ice bath. Trifluoroacetic acid (2 mL) was added dropwise while maintaining the temperature at 0 °C. The mixture was stirred for 2 hours at ambient temperature. The reaction mixture was evaporated under reduced pressure, diluted with DCM (30 mL), cooled to 0 °C, pH adjusted to 8.0 with saturated $NaHCO_3$ solution and extracted with DCM (3 x 30 mL). The combined organic layer was dried over sodium sulfate, filtered and evaporated under reduced pressure to obtain the compound for next step without further purification. To a 100 mL flask with deprotected compound from last step and a magnetic stir bar, anhydrous DCM (10 mL) was added. The solution was cooled to 0 °C using an ice bath. Triethylamine (0.95 mL, 6.9 mmol, 3.0 equiv) was added to the solution. Methanesulfonyl chloride (0.26 mL, 3.8 mmol, 1.7 equiv) was added dropwise at 0 °C. After stirring at room temperature overnight, the reaction mixture was diluted with ethyl acetate (50 mL) and washed with water (20 mL) and brine (10 mL). The organic layer was dried over anhydrous sodium sulfate, filtered and concentrated under reduced pressure. The resulting crude material was purified by flash column chromatography (Hexane/Ethyl acetate = 5/1), yielding **45** (0.63g, 87%).

Procedure for the synthesis of 46: 9-BBN (0.6 mL, 0.5 M in THF, 1.5 equiv) was added to a solution of **37** (0.2 mmol, 63.0 mg, 1.0 equiv) in THF (1.4 mL) at 0 °C. The mixture was stirred for 15 min at 0 °C and 3 hours at ambient temperature. The reaction was cooled to 0 °C, then 3 N aqueous NaOH (1 mL) and 30% aqueous H_2O_2 (0.6 mL) were added. The reaction was allowed to warm to room temperature and stirring continued for 3 h before quenching with saturated $Na_2S_2O_3$ (2 mL) at 0 °C and extracting with Et_2O (3 x 10 mL) three times. The organic layer was dried over $MgSO_4$, filtered, and concentrated under reduced pressure. The crude mixture was purified by flash column chromatography with hexanes/EtOAc (1/2) as eluent to give the corresponding product **46** (54.6 mg, 82%, >99:1 e.r.).

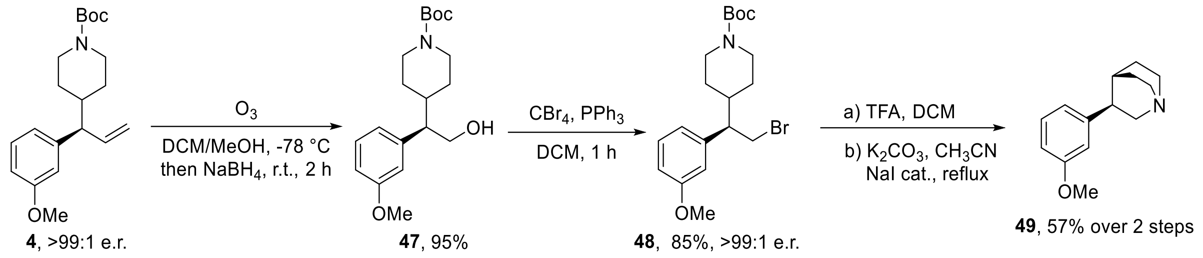

Procedure for the synthesis of 47: To the solution of the alkene **4** (66.2 mg, 0.2 mmol, 1.0 equiv,) in CH_2Cl_2/MeOH (4 mL, 3:1) and cooled to –78 °C before a stream of O_3–$O_{2(g)}$ was gently bubbled through the solution, until the solution appears blue for 1~2 minutes. The reaction was then purged by bubbling a gentle stream of $N_{2(g)}$ through the solution for 20 min, which was followed by the portion wise addition of $NaBH_4$ (35.6 mg, 1.0 mmol, 5.0 equiv). The mixture was stirred at –78 °C for 15 min followed by 2 h at room temperature before quenching with brine (5 mL) and extracting with EtOAc (3 × 5 mL). The organic phases were combined, dried over $MgSO_4$, filtered, and concentrated under reduced pressure to give a crude material that was then purified by column chromatography (EtOAc/hexane = 1/1) to give alcohol **47** (63.9 mg, 95%).

Procedure for the synthesis of 48: **47** (112 mg, 0.34 mmol, 1.0 equiv) and Ph_3P (116 mg, 0.44 mmol, 1.3 equiv) were dissolved in **anhydrous** DCM (4 mL) and CBr_4 (147 mg, 0.44 mmol, 1.3 equiv) was added in portions at 0 °C. The reaction was stirred for 5 min at 0 °C, then it was warmed to room temperature and stirred for 1 h. After completion (monitored by TLC, EtOAc /Hexane = 1/1), the solvent was evaporated under reduced pressure. The product was isolated by chromatography on silica gel (EtOAc/Hexane = 1/5) to yield **48** (112.1 mg, 85%).

Procedure for the synthesis of 49: An oven-dried 25 mL flask equipped with a magnetic stir bar was purged with nitrogen (N_2) three times. Compound **48** (112.1 g, 0.28 mmol, 1.0 equiv) and anhydrous DCM (3 mL) were added under nitrogen. The solution was cooled to 0 °C using an ice bath. Trifluoroacetic acid (1 mL) was added dropwise while maintaining the temperature at 0 °C. The mixture was stirred for 1 hours at ambient temperature. The reaction mixture was evaporated under reduced pressure, diluted with DCM (10 mL), cooled to 0 °C, pH adjusted to 8.0 with saturated $NaHCO_3$ solution and extracted with DCM (3 x 10 mL). The combined organic layer was dried over sodium sulfate, filtered and evaporated under reduced pressure to

obtain the compound for next step without further purification. Under N_2, to a solution of deprotected compound in 4 mL of CH_3CN were added K_2CO_3 (77.4 mg, 2.0 equiv.) and NaI (4 mg, cat). The mixture was heated to reflux for 4 h and the solvent was evaporated under vacuum. 10 mL of water were added, and the aqueous phase extracted by 4x10 mL of DCM. The combined organic phases were dried over Na_2SO_4, filtered, and evaporated under vacuum. The crude was purified by chromatography on silica (DCM/MeOH/TEA: 90/9/1) to give **49** (34.6 mg, 57%).

2.6. Crystallography

An X-ray diffraction experiment for compound **11** was carried out at 200(2) K on a Bruker D8 Venture diffractometer using Mo K_{α} radiation (λ = 0.71073 Å). Intensities were integrated in SAINT[6] and an absorption correction based on equivalent reflections was applied using SADABS.[7] The structure was solved using ShelXT[8] and refined by full matrix least squares against F^2 in ShelXL[9-10] using Olex2.[11] All of the non-hydrogen atoms were refined anisotropically whilst all of the hydrogen atoms were located geometrically and refined using a riding model. Disorder in the tertiary butyl and vinyl groups has been modelled by applying constraints to the displacement parameters of each pair of disordered atoms and restraining the bond lengths to be equivalent for each pair of disordered bonds. The distances between the backbone carbon and the terminal CH_2 carbon of the disordered vinyl group were also constrained to be equivalent. Absolute structure has been determined by refinement of the Flack parameter (−0.002(19)). Crystal structure and refinement data are given in Table X. Crystallographic data have been deposited with the Cambridge Crystallographic Data Centre as supplementary publication CCDC 2408112. Copies of the data can be obtained free of charge on application to CCDC, 12 Union Road, Cambridge CB2 1EZ, UK (fax +44 1223 336033, email: deposit@ccdc.cam.ac.uk).

Table S3: Crystal data and structure refinement for **11**

Empirical formula	$C_{19}H_{26}ClNO_2$
Formula weight	335.86
Temperature/K	200(2)
Crystal system	Orthorhombic
Space group	$P2_12_12_1$
a/Å	7.9496(2)
b/Å	11.9132(3)
c/Å	20.0313(6)
Volume/Å^3	1897.06(9)
Z	4
σ_{calc}/g cm^{-3}	1.176
μ/mm−1	0.210
F(000)	720
Crystal size/mm^3	0.51×0.35×0.21
Radiation	Mo K_α
2θ range for data collection	3.98-55.88
Index ranges	$-10\leq h\leq 10$<br>$-15\leq k\leq 15$<br>$-26\leq l\leq 26$
Reflections collected	47766
Independent reflections	4536 [R_{int} = 0.0541, R_{sigma} = 0.0279]
Data/restraints/parameters	4536/6/231
Goodness of fit on F^2	1.044
Final R indices [I>=2σ(I)]	R_1 = 0.0408<br>wR_2 = 0.0934<br>(3637 reflections)
Final R indices [all data]	R_1 = 0.0568<br>wR_2 =0.1029
Largest diff. peak/hole/e Å−3	−0.160/0.258
Flack parameter	−0.002(19)

2.7. Characterization Data for Products.

tert-butyl (*R*)-4-(1-(3-methoxyphenyl)allyl)piperidine-1-carboxylate (4)

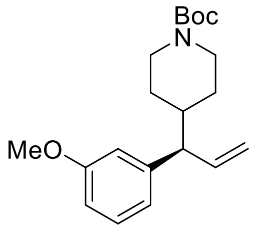

Prepared following **General Procedure A** using (*S*)-**L1**. Purification by flash column chromatography (Hexane/Ethyl acetate/DCM = 10/1/1) gave the title compound (36.1 mg, 55%, >99:1 e.r.).

^{1}H NMR (500 MHz, $CDCl_3$) δ 7.22 (t, J = 7.9 Hz, 1H), 6.74 (dd, J = 8.2, 2.9 Hz, 2H), 6.69 (t, J = 2.1 Hz, 1H), 5.98 – 5.89 (m, 1H), 5.05 (s, 1H), 5.05 – 5.01 (m, 1H), 4.00 – 4.12 (m, 2H), 3.80 (s, 3H), 2.89 (t, J = 9.3 Hz, 1H), 2.58 – 2.67 (m, 1H), 1.83 – 1.85 (m, 1H), 1.66 – 1.72 (m, 1H), 1.44 (s, 9H), 1.33 – 1.36 (m, 1H), 1.08 – 1.15 (m, 1H), 0.97-1.04 (m, 1H) ppm. **^{13}C NMR** (151 MHz, $CDCl_3$) δ 159.9, 154.9, 144.9, 140.2, 129.7, 120.3, 115.9, 114.1, 111.3, 79.4, 57.1, 55.3, 44.1, 40.7, 30.6, 28.6 ppm. **Specific rotation** $[\alpha]_D^{22}$ = +14 (c = 0.7, CH_2Cl_2). **HRMS** (ESI) m/z calculated for $C_{20}H_{29}NO_3Na$ $[M+Na]^+$, 354.2040, found: 354.2042. **IR (neat)** 2975, 1690, 1599, 1422, 1165, 768 cm^{-1}. **HPLC conditions**: Chiral column IA, hexane: isopropanol = 99:1, flow rate = 0.8 mL/min, wavelength = 254 nm, t_R = 16.6 min for major isomer.

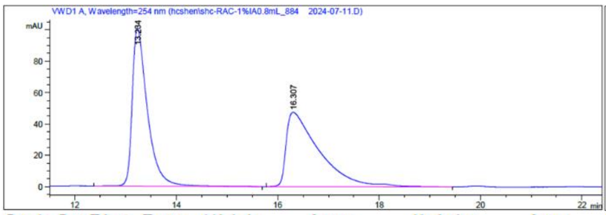

Peak #	RetTime [min]	Type	Width [min]	Area [mAU*s]	Height [mAU]	Area %
1	13.234	BB	0.3079	2107.55396	100.84420	49.5232
2	16.307	BV R	0.6361	2148.13452	47.43737	50.4768

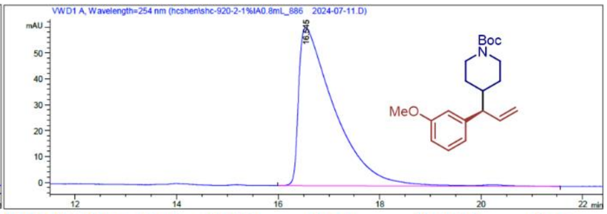

Peak #	RetTime [min]	Type	Width [min]	Area [mAU*s]	Height [mAU]	Area %
1	16.545	BV R	0.6891	3007.06226	60.97688	100.0000

tert-butyl (*R*)-4-(1-(3,5-difluorophenyl)allyl)piperidine-1-carboxylate (7)

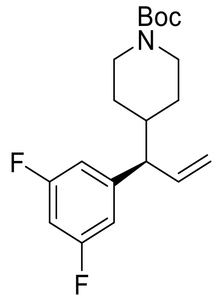

Prepared following **General Procedure A** using (*S*)-**L1**. Purification by flash column chromatography (Hexane/Ethyl acetate/DCM = 10/1/1) gave the title compound (40.3 mg, 60%, 99:1 e.r.).

^{1}H NMR (500 MHz, $CDCl_3$) δ 6.72 – 6.60 (m, 3H), 5.86 (ddd, J = 16.9, 10.1, 9.2 Hz, 1H), 5.10 (dd, J = 10.2, 1.5 Hz, 1H), 5.06 (ddd, J = 16.9, 1.5, 0.8 Hz, 1H), 4.14 – 4.01 (m, 2H), 2.92 (t, J = 9.2 Hz, 1H), 2.69 – 2.56 (m, 2H), 1.86 – 1.77 (m, 1H), 1.72 – 1.60 (m, 1H), 1.44 (s, 9H), 1.37 – 1.29 (m, 1H), H1.16 – 0.96 (m, 2H) ppm. **^{13}C NMR** (126 MHz, $CDCl_3$) δ 164.2 (d, J = 13.0 Hz), 162.3 (d, J = 12.9 Hz), 154.88, 147.3 (t, J = 8.5 Hz), 138.9, 117.0, 110.9 – 110.5 (m), 101.9 (t, J = 25.3 Hz), 79.5, 56.8, 44.0, 40.6, 30.4, 28.6 ppm. **Specific rotation** $[\alpha]_D^{24}$ = +11 (c = 2.3, CH_2Cl_2). **HRMS** (ESI) m/z calculated for $C_{19}H_{26}F_2NO_2$ $[M+H]^+$, 338.1926, found: 338.1933. **IR (neat)** 3080, 2938, 1687, 1597, 1421, 1116, 985, 870 cm^{-1}. **HPLC conditions**: Chiral column IC, hexane: isopropanol = 99.5:0.5, flow rate = 0.7 mL/min, wavelength = 254 nm, t_R = 20.1 min for major isomer.

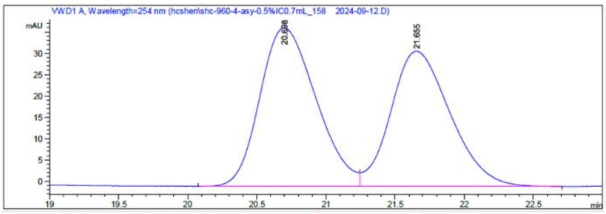

Peak #	RetTime [min]	Type	Width [min]	Area [mAU*s]	Height [mAU]	Area %
1	20.698	BV	0.4400	1049.24219	37.17022	52.5773
2	21.655	VB	0.4648	946.37494	31.70135	47.4227

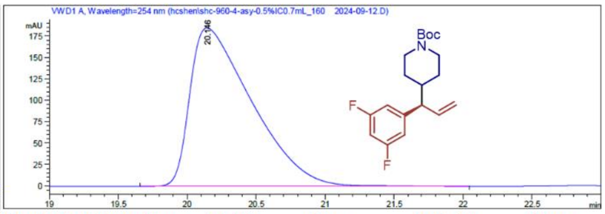

Peak #	RetTime [min]	Type	Width [min]	Area [mAU*s]	Height [mAU]	Area %
1	20.146	BB	0.4686	5763.79248	184.84151	100.0000

tert-butyl (*R*)-4-(1-(3,5-dichlorophenyl)allyl)piperidine-1-carboxylate (8)

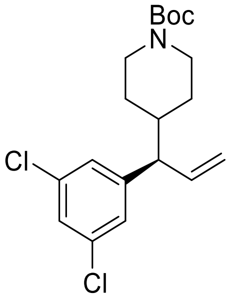

Prepared following **General Procedure A** using (*S*)-**L1**. Purification by flash column chromatography (Hexane/Ethyl acetate/DCM = 10/1/1) gave the title compound (48.5 mg, 66%, 99:1 e.r.).

^{1}H NMR (500 MHz, $CDCl_3$) δ 7.36 (d, J = 8.2 Hz, 1H), 7.23 (d, J = 2.1 Hz, 1H), 6.98 (dd, J = 8.3, 2.0 Hz, 1H), 5.87 (ddd, J = 16.9, 10.1, 9.1 Hz, 1H), 5.09 (dd, J = 10.2, 1.5 Hz, 1H), 5.04 (ddd, J = 16.9, 1.5, 0.8 Hz, 1H), 4.14-4.01 (m, 2H), 2.90 (t, J = 9.2 Hz, 1H), 2.69-2.55 (m, 2H), 1.86 – 1.76 (m, 1H), 1.70-1.62 (m, 1H), 1.44 (s, 9H), 1.34-1.29 (m, 1H), 1.16 – 1.04 (m, 1H), 1.05 – 0.93 (m, 1H) ppm. **^{13}C NMR** (126 MHz, $CDCl_3$) δ 154.9, 143.6, 139.1, 132.7, 130.6, 130.4, 129.9, 127.4, 116.8, 79.5, 56.1, 44.0, 40.6, 30.5, 28.6 ppm. **Specific rotation** $[\alpha]_D^{24}$ = +12 (c = 0.7, CH_2Cl_2). **HRMS** (ESI) m/z calculated for $C_{19}H_{26}Cl_2NO_2$ $[M+H]^+$, 370.1335, found: 370.1351. **IR (neat)** 3070, 2936, 1686, 1422, 1166, 919 cm^{-1}. **HPLC conditions**: Chiral column IC, hexane: isopropanol = 99:1, flow rate = 0.7 mL/min, wavelength = 230 nm, t_R = 23.5 min for major isomer.

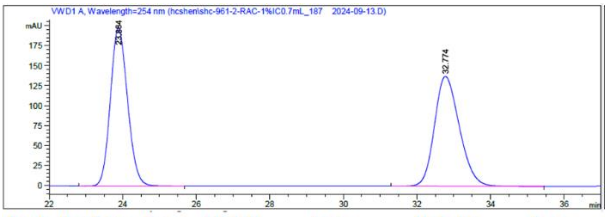

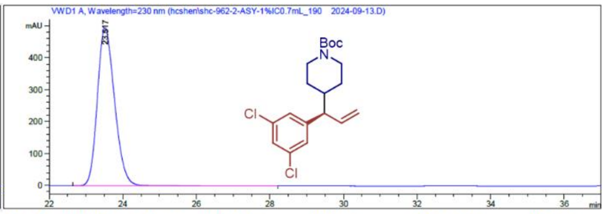

Peak #	RetTime [min]	Type	Width [min]	Area [mAU*s]	Height [mAU]	Area %
1	23.884	BB	0.5070	6420.04248	197.67513	49.8078
2	32.774	BB	0.7296	6469.60059	137.47655	50.1922

Peak #	RetTime [min]	Type	Width [min]	Area [mAU*s]	Height [mAU]	Area %
1	23.517	BB	0.5050	1.61216e4	495.23914	100.0000

tert-butyl (*R*)-4-(1-phenylallyl)piperidine-1-carboxylate (9)

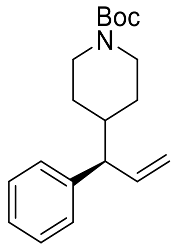

Prepared following **General Procedure A** using (*S*)-**L1**. Purification by flash column chromatography (Hexane/Ethyl acetate/DCM = 10/1/1) gave the title compound (40.3 mg, 67%, 99:1 e.r.).

^{1}H NMR (500 MHz, $CDCl_3$) δ 7.33 – 7.26 (m, 2H), 7.23 – 7.16 (m, 1H), 7.17 – 7.11 (m, 2H), 5.96 (ddd, J = 16.2, 10.8, 9.2 Hz, 1H), 5.06 (d, J = 0.7 Hz, 1H), 5.03 (ddd, J = 7.8, 1.7, 0.7 Hz, 1H), 4.13 – 3.99 (m, 2H), 2.93 (t, J = 9.3 Hz, 1H), 2.67 (td, J = 12.9, 2.8 Hz, 1H), 2.57 (td, J = 12.9, 2.9 Hz, 1H), 1.90 – 1.81 (m, 1H), 1.70 (tdd, J = 11.5, 8.2, 5.8 Hz, 1H), 1.44 (s, 9H), 1.37 – 1.28 (m, 1H), 1.20 – 1.07 (m, 1H), 1.05 – 0.94 (m, 1H) ppm. **^{13}C NMR** (126 MHz, $CDCl_3$) δ 155.0, 143.3, 140.3, 128.7, 128.0, 126.4, 115.8, 79.4, 57.1, 44.1, 40.7, 30.6, 28.6 ppm. **Specific rotation** $[\alpha]_D^{24}$ = +19 (c = 0.3, CH_2Cl_2). **HRMS** (ESI) m/z calculated for $C_{19}H_{28}NO_2$ $[M+H]^+$, 302.2115, found: 302.2126. **IR (neat)** 2975, 1691, 1422, 1170, 702 cm^{-1}. **HPLC conditions**: Chiral column IA, hexane: isopropanol = 99:1, flow rate = 1.0 mL/min, wavelength = 230 nm, t_R = 6.5 min for major isomer.

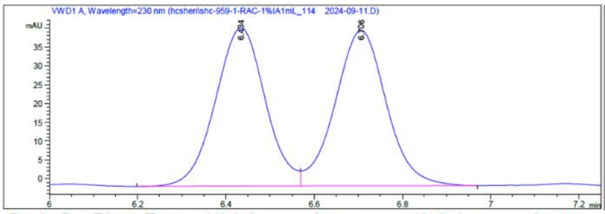

Peak #	RetTime [min]	Type	Width [min]	Area [mAU*s]	Height [mAU]	Area %
1	6.434	BV	0.1240	331.09509	42.01676	49.7879
2	6.706	VB	0.1264	333.91626	41.26125	50.2121

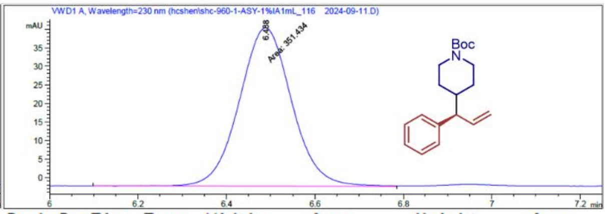

Peak #	RetTime [min]	Type	Width [min]	Area [mAU*s]	Height [mAU]	Area %
1	6.488	MM	0.1371	351.43381	42.72858	100.0000

tert-butyl (*R*)-4-(1-(p-tolyl)allyl)piperidine-1-carboxylate (10)

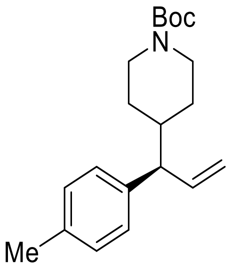

Prepared following **General Procedure A** using (*S*)-**L1**. Purification by flash column chromatography (Hexane/Ethyl acetate/DCM = 10/1/1) gave the title compound (33.7 mg, 54%, 99:1 e.r.).

^{1}H NMR (500 MHz, $CDCl_3$) δ 7.14 – 7.08 (m, 2H), 7.06 – 7.01 (m, 2H), 6.00 – 5.89 (m, 1H), 5.04 (d, J = 1.0 Hz, 1H), 5.03 – 4.98 (m, 1H), 3.98 – 4.13 (m, 2H), 2.89 (t, J = 9.3 Hz, 1H), 2.67 (td, J = 12.9, 2.8 Hz, 1H), 2.57 (td, J = 12.9, 2.8 Hz, 1H), 2.31 (s, 3H), 1.88 – 1.79 (m, 1H), 1.74 – 1.62 (m, 1H), 1.44 (s, 9H), 1.37 – 1.32 (m, 1H), 1.17 – 1.05 (m, 1H), 1.05 – 0.93 (m, 1H) ppm. **^{13}C NMR** (126 MHz, $CDCl_3$) δ 155.0, 140.6, 140.2, 136.0, 129.4, 127.8, 115.6, 79.3, 56.6, 44.1, 40.7, 30.6, 28.6, 21.1 ppm. **Specific rotation** $[\alpha]_D^{24}$ = +15 (c = 0.6, CH_2Cl_2). **HRMS** (ESI) m/z calculated for $C_{20}H_{30}NO_2$ $[M+H]^+$, 316.2271, found: 316.2282. **IR (neat)** 2975, 1690, 1421, 1167, 807 cm^{-1}. **HPLC conditions**: Chiral column IA, hexane: isopropanol = 99:1, flow rate = 1.0 mL/min, wavelength = 230 nm, t_R = 5.6 min for major isomer.

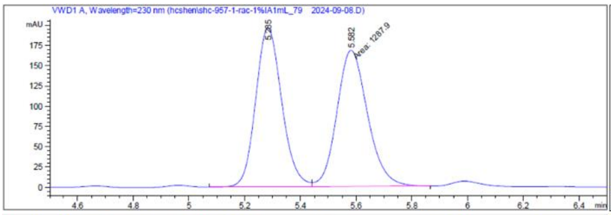

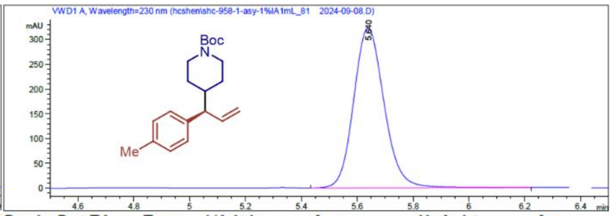

Peak #	RetTime [min]	Type	Width [min]	Area [mAU*s]	Height [mAU]	Area %
1	5.285	BV	0.1024	1280.46594	196.06329	49.8552
2	5.582	MM	0.1276	1287.90186	168.26599	50.1448

Peak #	RetTime [min]	Type	Width [min]	Area [mAU*s]	Height [mAU]	Area %
1	5.640	BB	0.1176	2442.51660	322.16824	100.0000

tert-butyl (*R*)-4-(1-(4-chlorophenyl)allyl)piperidine-1-carboxylate (11)

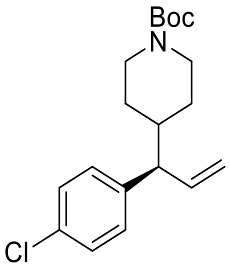

Prepared following **General Procedure A** using (*S*)-**L1**. Purification by flash column chromatography (Hexane/Ethyl acetate/DCM = 10/1/1) gave the title compound (33.8 mg, 51%, 99:1 e.r.).

^{1}H NMR (500 MHz, $CDCl_3$) δ 7.30 – 7.23 (m, 2H), 7.11 – 7.03 (m, 2H), 5.90 (ddd, J = 16.8, 10.1, 9.1 Hz, 1H), 5.11 – 4.99 (m, 2H), 4.13-3.99 (m, 2H), 2.91 (t, J = 9.2 Hz, 1H), 2.71 – 2.61 (m, 1H), 2.61 – 2.50 (m, 1H), 1.85 – 1.80 (m, 1H), 1.72 – 1.60 (m, 1H), 1.43 (s, 9H), 1.35 – 1.27 (m, 1H), 1.17 – 1.05 (m, 1H), 1.03 – 0.91 (m, 1H) ppm. **^{13}C NMR** (126 MHz, $CDCl_3$) δ 154.9, 141.7, 139.8, 132.1, 129.3, 128.8, 116.2, 79.4, 56.3, 44.0, 40.7, 30.5, 28.6 ppm. **Specific rotation** $[\alpha]_D^{23}$ = +22 (c = 0.5, CH_2Cl_2). **HRMS** (ESI) m/z calculated for $C_{19}H_{27}ClNO_2$ $[M+H]^+$, 336.1725, found: 336.1728. **IR (neat)** 2937, 1689, 1491, 1422, 1167, 811 cm^{-1}. **HPLC conditions**: Chiral column IA, hexane: isopropanol = 99:1, flow rate = 1.0 mL/min, wavelength = 230 nm, t_R = 7.1 min for major isomer.

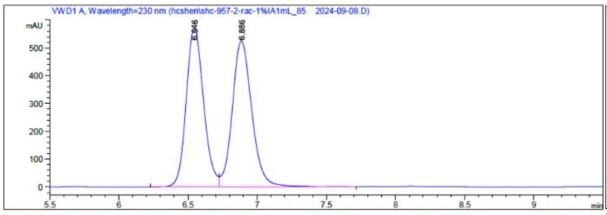

Peak #	RetTime [min]	Type	Width [min]	Area [mAU*s]	Height [mAU]	Area %
1	6.546	BV	0.1369	4983.77686	570.30664	49.1749
2	6.886	VV R	0.1525	5151.02246	523.33618	50.8251

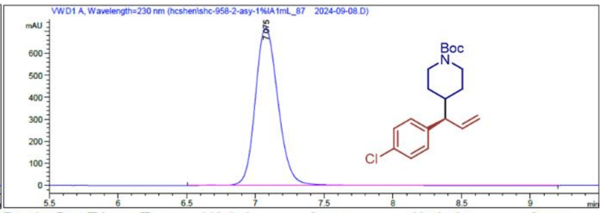

Peak #	RetTime [min]	Type	Width [min]	Area [mAU*s]	Height [mAU]	Area %
1	7.075	VB R	0.1808	8259.77832	716.93799	100.0000

tert-butyl (*R*)-4-(1-(4-bromophenyl)allyl)piperidine-1-carboxylate (12)

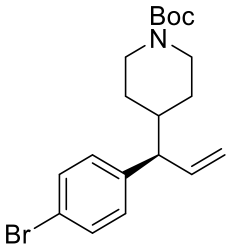

Prepared following **General Procedure A** using (*S*)-**L1**. Purification by flash column chromatography (Hexane/Ethyl acetate/DCM = 10/1/1) gave the title compound (30.3 mg, 40%, >99:1 e.r.).

^{1}H NMR (500 MHz, $CDCl_3$) δ 7.45 – 7.38 (m, 2H), 7.06 – 6.99 (m, 2H), 5.90 (ddd, J = 16.8, 10.1, 9.1 Hz, 1H), 5.10 – 4.94 (m, 2H), 4.18 – 3.93 (m, 2H), 2.90 (t, J = 9.2 Hz, 1H), 2.68 – 2.63 (m, 1H), 2.59 – 2.54 (m, 1H), 1.85 – 1.80 (m, 1H), 1.70 – 1.60 (m, 1H), 1.43 (s, 9H), 1.34 – 1.29 (m, 1H), 1.16 – 1.04 (m, 1H), 1.02 – 0.94 (m, 1H) ppm. **^{13}C NMR** (126 MHz, $CDCl_3$) δ 154.9, 142.2, 139.7, 131.8, 129.7, 120.2, 116.3, 79.4, 56.4, 44.0, 40.7, 30.5, 28.6 ppm. **Specific rotation** $[\alpha]_D^{24}$ = +14 (c = 0.7, CH_2Cl_2). **HRMS** (ESI) m/z calculated for $C_{19}H_{27}BrNO_2$ $[M+H]^+$, 380.1220, found: 380.1232. **IR (neat)** 2975, 1689, 1422, 1167, 1010 cm^{-1}. **HPLC conditions**: Chiral column IA, hexane: isopropanol = 99:1, flow rate = 1.0 mL/min, wavelength = 230 nm, t_R = 7.3 min for major isomer, t_R = 6.8 min for minor isomer.

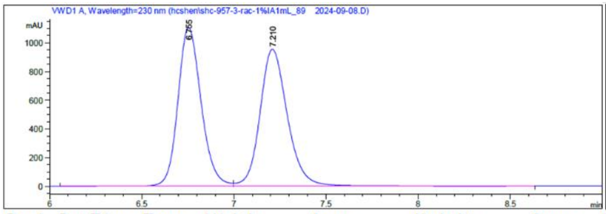

Peak #	RetTime [min]	Type	Width [min]	Area [mAU*s]	Height [mAU]	Area %
1	6.755	BV	0.1373	9695.81738	1105.26147	49.4480
2	7.210	VB	0.1620	9912.29883	953.99976	50.5520

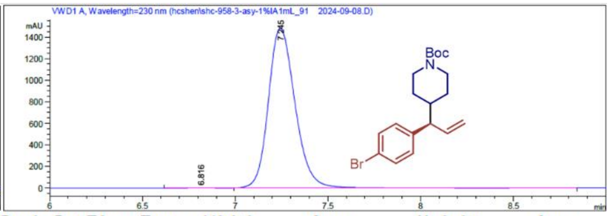

Peak #	RetTime [min]	Type	Width [min]	Area [mAU*s]	Height [mAU]	Area %
1	6.816	BV E	0.1293	25.10327	3.00977	0.1682
2	7.245	VV R	0.1550	1.48987e4	1481.28711	99.8318

tert-butyl (*R*)-4-(1-(4-(methoxycarbonyl)phenyl)allyl)piperidine-1-carboxylate (13)

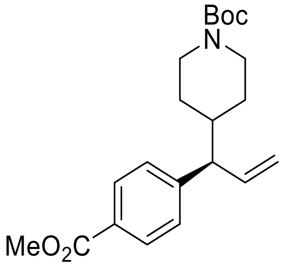

Prepared following **General Procedure A** using (*S*)-**L1**. Purification by flash column chromatography (Hexane/Ethyl acetate/DCM = 10/1/1) gave the title compound (43.6 mg, 61%, 99:1 e.r.).

^{1}H NMR (500 MHz, $CDCl_3$) δ 8.00 – 7.87 (m, 2H), 7.24 – 7.16 (m, 2H), 5.93 (ddd, J = 16.9, 10.2, 9.2 Hz, 1H), 5.11 – 5.00 (m, 2H), 4.12 (d, J = 13.3 Hz, 1H), 3.99 (d, J = 13.5 Hz, 1hH), 3.89 (s, 3H), 2.99 (t, J = 9.3 Hz, 1H), 2.69 – 2.63 (m, 1H), 2.59 – 2.53 (m, 1H), 1.88 – 1.79 (m, 1H), 1.78 – 1.66 (m, 1H), 1.43 (s, 9H), 1.32 – 1.23 (m, 1H), 1.18 – 1.06 (m, 1H), 1.04 – 0.94 (m, 1H) ppm. **^{13}C NMR** (126 MHz, $CDCl_3$) δ 167.1, 154.9, 148.7, 139.4, 130.0, 128.4, 128.0, 116.6, 79.4, 57.0, 52.1, 44.0, 40.7, 30.5, 28.6 ppm. **Specific rotation** $[\alpha]_D^{24}$ = +17 (c = 0.8, CH_2Cl_2). **HRMS** (ESI) m/z calculated for $C_{21}H_{29}NO_4Na$ $[M+Na]^+$, 382.1989, found: 382.1991. **IR (neat)** 2976, 1721, 1689, 1609, 1422, 1277, 1168, 771 cm^{-1}. **HPLC conditions**: Chiral column IA, hexane: isopropanol = 99:1, flow rate = 1.0 mL/min, wavelength = 230 nm, t_R = 22.8 min for major isomer.

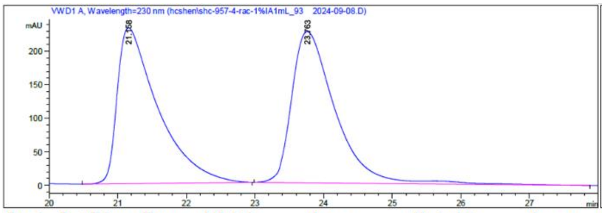

Peak #	RetTime [min]	Type	Width [min]	Area [mAU*s]	Height [mAU]	Area %
1	21.158	BB	0.5943	9572.91797	232.31905	49.0230
2	23.763	BV R	0.6511	9954.50098	225.34340	50.9770

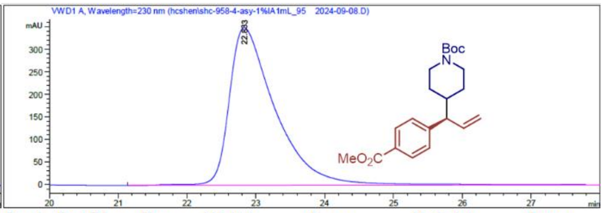

Peak #	RetTime [min]	Type	Width [min]	Area [mAU*s]	Height [mAU]	Area %
1	22.833	BV R	0.6841	1.65334e4	350.03168	100.0000

tert-butyl (*R*)-4-(1-(o-tolyl)allyl)piperidine-1-carboxylate (14)

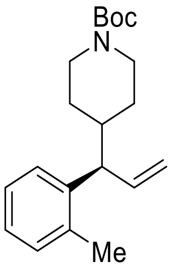

Prepared following **General Procedure A** using (*S*)-**L1**. Purification by flash column chromatography (Hexane/Ethyl acetate/DCM = 10/1/1) gave the title compound (21.5 mg, 34%, 98:2 e.r.).

^{1}H NMR (500 MHz, $CDCl_3$) δ 7.22 – 7.11 (m, 3H), 7.08 (ddd, J = 7.6, 6.2, 2.2 Hz, 1H), 5.91 – 5.80 (m, 1H), 5.04 – 4.97 (m, 2H), 4.15 – 4.13 (M, 1H), 4.01 – 3.98 (m, 1H), 3.23 (t, J = 9.5 Hz, 1H), 2.71 – 2.66 (m, 1H), 2.60 – 2.54 (m, 1H), 2.31 (s, 3H), 1.96 – 1.87 (m, 1H), 1.80 – 1.72 (m, 1H), 1.44 (s, 9H), 1.42 – 1.33 (m, 1H), 1.22 – 1.10 (m, 1H), 1.04 – 0.92 (m, 1H) ppm. **^{13}C NMR** (126 MHz, $CDCl_3$) δ 155.0, 141.3, 140.4, 136.0, 130.7, 126.6, 126.4, 126.0, 115.6, 79.4, 51.7, 44.2, 40.5, 30.9, 30.3, 28.6, 20.1 ppm. **Specific rotation** $[\alpha]_D^{24}$ = +21 (c = 0.3, CH_2Cl_2). **HRMS** (ESI) m/z calculated for $C_{20}H_{29}NO_2Na$ $[M+Na]^+$, 338.2091, found: 338.2106. **IR (neat)** 2975, 1691, 1421, 1169, 755 cm^{-1}. **HPLC conditions**: Chiral column IC, hexane: isopropanol = 99:1, flow rate = 1.0 mL/min, wavelength = 230 nm, t_R = 17.1 min for major isomer, t_R = 16.4 min for minor isomer.

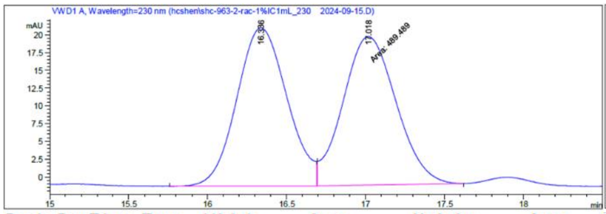

Peak #	RetTime [min]	Type	Width [min]	Area [mAU*s]	Height [mAU]	Area %
1	16.336	BV	0.3487	493.15561	22.26238	50.1865
2	17.018	MM	0.3908	489.48941	20.87475	49.8135

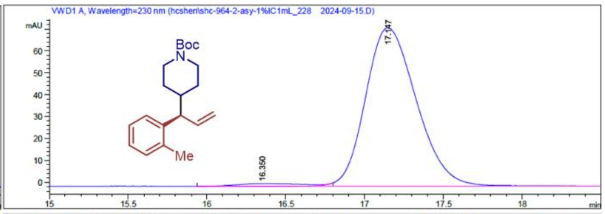

Peak #	RetTime [min]	Type	Width [min]	Area [mAU*s]	Height [mAU]	Area %
1	16.350	BV E	0.3330	31.89776	1.13744	1.9318
2	17.147	VB R	0.3474	1619.25647	72.24888	98.0682

tert-butyl (*R*)-4-(1-(2-chlorophenyl)allyl)piperidine-1-carboxylate (15)

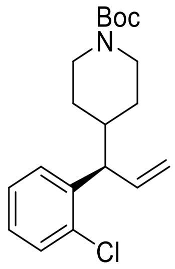

Prepared following **General Procedure A** using (*S*)-**L1**. Purification by flash column chromatography (Hexane/Ethyl acetate/DCM = 10/1/1) gave the title compound (25.1 mg, 38%, 99:1 e.r.).

^{1}H NMR (500 MHz, $CDCl_3$) δ 7.38 – 7.32 (m, 1H), 7.26 – 7.18 (m, 2H), 7.12 (ddd, J = 8.0, 6.3, 2.7 Hz, 1H), 5.91 (ddd, J = 16.9, 10.1, 9.1 Hz, 1H), 5.15 – 5.03 (m, 2H), 4.14-3.99 (m, 2H), 3.64 (t, J = 9.3 Hz, 1H), 2.72 – 2.63 (m, 1H), 2.62 – 2.56 (m, 1H), 1.90 – 1.81 (m, 1H), 1.81 – 1.76 (m, 1H), 1.44 (s, 9H), 1.35 – 1.30 (m, 1H), 1.24 – 1.05 (m, 2H) ppm. **^{13}C NMR** (126 MHz, $CDCl_3$) δ 154.9, 140.6, 138.9, 134.1, 130.0, 128.7, 127.4, 127.1, 116.9, 79.4, 51.7, 44.0, 40.3, 30.4, 30.0, 28.6 ppm. **Specific rotation** $[\alpha]_D^{24}$ = +8 (c = 0.5, CH_2Cl_2). **HRMS** (ESI) m/z calculated for $C_{19}H_{26}ClNO_2Na$ $[M+Na]^+$, 358.1544, found: 358.1559. **IR (neat)** 2984, 1691, 1428, 1034, 754 cm^{-1}. **HPLC conditions**: Chiral column IC, hexane: isopropanol = 99:1, flow rate = 1.0 mL/min, wavelength = 254 nm, t_R = 10.6 min for major isomer.

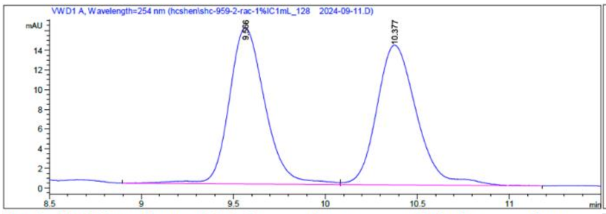

Peak #	RetTime [min]	Type	Width [min]	Area [mAU*s]	Height [mAU]	Area %
1	9.566	BV	0.2085	216.18439	15.86623	50.1681
2	10.377	VB	0.2318	214.73604	14.19881	49.8319

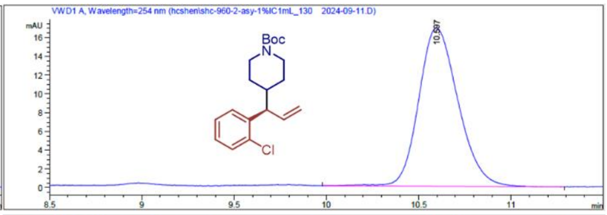

Peak #	RetTime [min]	Type	Width [min]	Area [mAU*s]	Height [mAU]	Area %
1	10.597	BB	0.2318	251.25696	16.88968	100.0000

tert-butyl (R)-4-(1-(naphthalen-2-yl)allyl)piperidine-1-carboxylate (16)

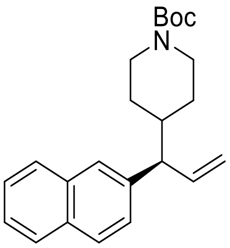

Prepared following **General Procedure A** using (*S*)-**L1**. Purification by flash column chromatography (Hexane/Ethyl acetate/DCM = 10/1/1) gave the title compound (43.9 mg, 44%, 99:1 e.r.).

^{1}H NMR (500 MHz, $CDCl_3$) δ 7.85 – 7.74 (m, 3H), 7.61 – 7.56 (m, 1H), 7.51 – 7.39 (m, 2H), 7.31 (dd, J = 8.5, 1.7 Hz, 1H), 6.15 – 6.00 (m, 1H), 5.13 – 5.10 (m, 1H), 5.08 (s, 1H), 4.16 – 3.97 (m, 2H), 3.11 (t, J = 9.2 Hz, 1H), 2.73 – 2.67 (m, 1H), 2.61 – 2.55 (m, 1H), 1.95 – 1.86 (m, 1H), 1.87 – 1.79 (m, 1H), 1.44 (s, 9H), 1.37 – 1.31 (m, 1H), 1.25 – 1.13 (m, 1H), 1.11 – 0.99 (m, 1H) ppm. **^{13}C NMR** (126 MHz, $CDCl_3$) δ 154.9, 140.7, 140.2, 133.7, 132.4, 128.4, 127.8, 127.7, 126.5, 126.2, 126.2, 125.6, 116.1, 79.4, 77.4, 57.1, 44.1, 40.7, 30.7, 28.6 ppm. **Specific rotation** $[\alpha]_D^{24}$ = +12 (c = 0.5, CH_2Cl_2). **HRMS** (ESI) m/z calculated for $C_{23}H_{29}NO_2Na$ $[M+Na]^+$, 374.2091, found: 374.2092. **IR (neat)** 2974, 1690, 1423, 1277, 1169, 748 cm^{-1}. **HPLC conditions**: Chiral column IA, hexane: isopropanol = 99:1, flow rate = 0.7 mL/min, wavelength = 254 nm, t_R = 14.2 min for major isomer.

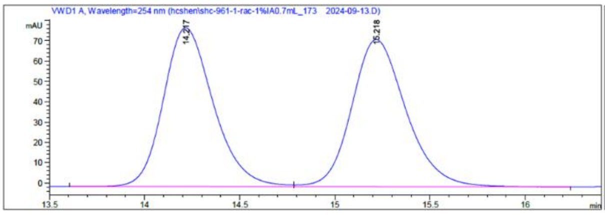

Peak #	RetTime [min]	Type	Width [min]	Area [mAU*s]	Height [mAU]	Area %
1	14.217	BV	0.2805	1428.55066	78.07279	49.8355
2	15.218	VB	0.3047	1437.97888	72.35264	50.1645

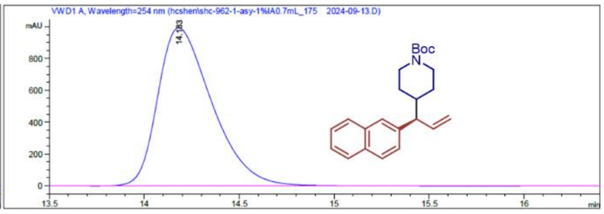

Peak #	RetTime [min]	Type	Width [min]	Area [mAU*s]	Height [mAU]	Area %
1	14.183	VV R	0.3090	1.99745e4	995.07190	100.0000

***tert*-butyl (*R*)-4-(1-(2-chloroquinolin-3-yl)allyl)piperidine-1-carboxylate (17)**

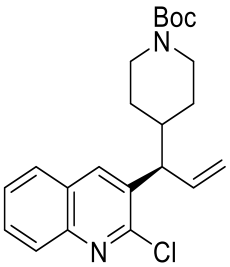

Prepared following **General Procedure A** using (*S*)-**L1**. Purification by flash column chromatography (Hexane/Ethyl acetate = 5/1) gave the title compound (39.9 mg, 52%, 99:1 e.r.).

^{1}H NMR (500 MHz, $CDCl_3$) δ 7.99 (dt, J = 8.6, 0.9 Hz, 1H), 7.97 (s, 1H), 7.79 (dd, J = 8.3, 1.4 Hz, 1H), 7.69 (ddd, J = 8.4, 6.9, 1.5 Hz, 1H), 7.55 (ddd, J = 8.1, 6.9, 1.2 Hz, 1H), 6.00 (ddd, J = 16.9, 10.1, 9.1 Hz, 1H), 5.22 – 5.14 (m, 2H), 4.16 – 4.03 (m, 2H), 3.75 (t, J = 9.0 Hz, 1H), 2.74 – 2.58 (m, 2H), 1.93 (tdt, J = 11.9, 8.8, 3.5 Hz, 1H), 1.86 (dt, J = 13.3, 2.9 Hz, 1H), 1.44 (s, 9H), 1.30 – 1.15 (m, 2H) ppm. **^{13}C NMR** (126 MHz, $CDCl_3$) δ 154.9, 151.4, 146.4, 137.9, 136.8, 135.1, 130.2, 128.4, 127.5, 127.3, 127.3, 117.9, 79.5, 77.4, 77.2, 76.9, 51.8, 44.0, 40.5, 30.0, 28.6 ppm. **Specific rotation** $[\alpha]_D^{24}$ = +24 (c = 0.8, CH_2Cl_2). **HRMS** (ESI) m/z calculated for $C_{22}H_{28}ClN_2O_2$ $[M+H]^+$, 387.1834, found: 387.1816. **IR (neat)** 2976, 1690, 1424, 1169, 1033, 754 cm^{-1}. **HPLC conditions**: Chiral column IA, hexane: isopropanol = 90:10, flow rate = 1 mL/min, wavelength = 254 nm, t_R = 22.9 min for major isomer.

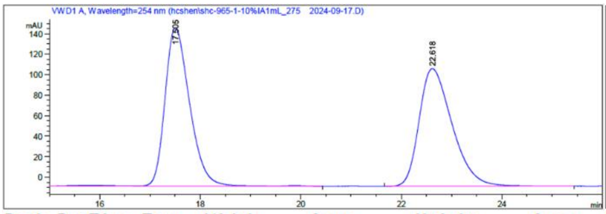

Peak #	RetTime [min]	Type	Width [min]	Area [mAU*s]	Height [mAU]	Area %
1	17.505	VV R	0.5215	5332.08057	154.65158	50.4606
2	22.618	BB	0.6923	5234.74902	114.77245	49.5394

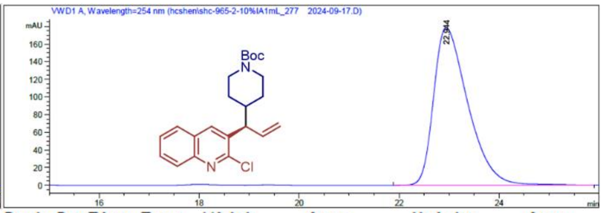

Peak #	RetTime [min]	Type	Width [min]	Area [mAU*s]	Height [mAU]	Area %
1	22.944	BB	0.7124	8462.07910	178.29118	100.0000

tert-butyl (_R_)-4-(1-(thiophen-2-yl)allyl)piperidine-1-carboxylate (18)

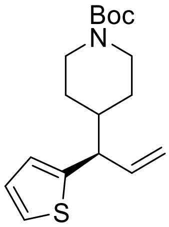

Prepared following **General Procedure A** using (*S*)-**L1**. Purification by flash column chromatography (Hexane/Ethyl acetate/DCM = 10/1/1) gave the title compound (27.6 mg, 45%, >99:1 e.r.).

^{1}H NMR (500 MHz, $CDCl_3$) δ 7.16 (dd, J = 5.2, 1.1 Hz, 1H), 6.94 (dd, J = 5.1, 3.4 Hz, 1H), 6.79 (dt, J = 3.5, 0.9 Hz, 1H), 5.91 (ddd, J = 16.6, 10.3, 9.1 Hz, 1H), 5.11 – 5.10 (m, 1H), 5.09 – 5.06 (m, 1H), 4.16 – 4.02 (m, 2H), 3.32 (t, J = 8.7 Hz, 1H), 2.64 (dtd, J = 15.7, 12.8, 2.9 Hz, 2H), 1.84 – 1.76 (m, 1H), 1.69 (tdt, J = 11.7, 8.3, 3.5 Hz, 1H), 1.55 – 1.47 (m, 1H), 1.44 (s, 9H), 1.18 – 1.02 (m, 2H) ppm. **^{13}C NMR** (126 MHz, $CDCl_3$) δ 154.9, 146.5, 139.4, 126.8, 124.1, 123.4, 116.3, 79.4, 51.7, 44.1, 42.1, 30.5, 30.1, 28.6 ppm. **Specific rotation** $[\alpha]_D^{24}$ = +33 (c = 0.4, CH_2Cl_2). **HRMS** (ESI) m/z calculated for $C_{17}H_{25}NO_2SNa$ $[M+Na]^+$, 330.1498, found: 330.1513. **IR (neat)** 2975, 1691, 1422, 1168, 694 cm^{-1}. **HPLC conditions**: Chiral column IC, hexane: isopropanol = 99:1, flow rate = 0.7 mL/min, wavelength = 230 nm, t_R = 22.3 min for major isomer, t_R = 20.6 min for minor isomer.

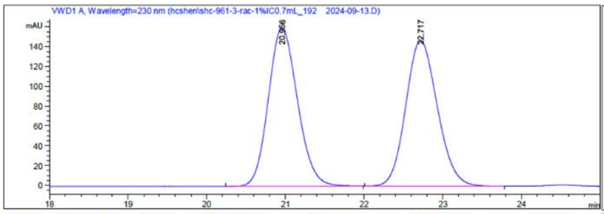

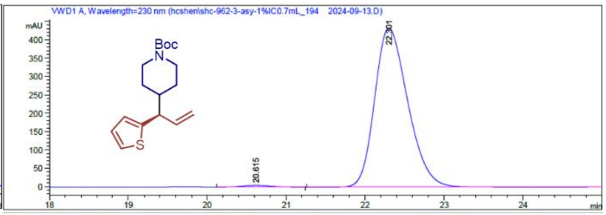

Peak #	RetTime [min]	Type	Width [min]	Area [mAU*s]	Height [mAU]	Area %
1	20.956	BB	0.3991	4113.30127	159.89137	50.1485
2	22.717	BB	0.4326	4088.94751	146.86115	49.8515

Peak #	RetTime [min]	Type	Width [min]	Area [mAU*s]	Height [mAU]	Area %
1	20.615	BB	0.3931	113.18118	4.44574	0.8890
2	22.301	BBA	0.4504	1.26178e4	433.25375	99.1110

***tert*-butyl (*R*, *E*)-4-(1-phenylpenta-1,4-dien-3-yl)piperidine-1-carboxylate (19)**

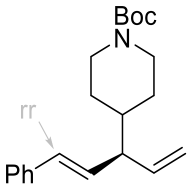

Prepared following **General Procedure A** using (*S*)-**L1**. Purification by flash column chromatography (Hexane/Ethyl acetate/DCM = 10/1/1) gave the title compound (34.2 mg, 50%, 92:8 r.r., >99:1 e.r.).

^{1}H NMR (500 MHz, $CDCl_3$) δ 7.38 – 7.32 (m, 2H), 7.30 (dd, J = 8.4, 6.8 Hz, 2H), 7.24 – 7.17 (m, 1H), 6.37 (d, J = 15.8 Hz, 1H), 6.12 (dd, J = 15.9, 8.5 Hz, 1H), 5.80 (ddd, J = 17.0, 10.3, 8.1 Hz, 1H), 5.12 – 5.03 (m, 2H), 4.11 (s, 2H), 2.78 – 2.60 (m, 3H), 1.76 – 1.68 (m, 2H), 1.52 (tdt, J = 11.3, 7.2, 3.5 Hz, 1H), 1.45 (s, 9H), 1.22 – 1.12 (m, 2H) ppm. **^{13}C NMR** (126 MHz, $CDCl_3$) δ 155.0, 139.2, 137.5, 131.0, 131.0, 128.6, 127.3, 126.2, 115.9, 79.4, 53.4, 44.2, 40.5, 30.0, 28.6 ppm. **Specific rotation** $[\alpha]_D^{24}$ = +20 (c = 0.5, CH_2Cl_2). **HRMS** (ESI) m/z calculated for $C_{21}H_{29}NO_2Na$ $[M+Na]^+$, 350.2091, found: 350.2102. **IR (neat)** 2974, 1689 1421, 1164, 750 cm^{-1}. **HPLC conditions**: Chiral column IC, hexane: isopropanol = 99:1, flow rate = 1 mL/min, wavelength = 230 nm, t_R = 13.6 min for major isomer, t_R = 15.5 min for minor isomer.

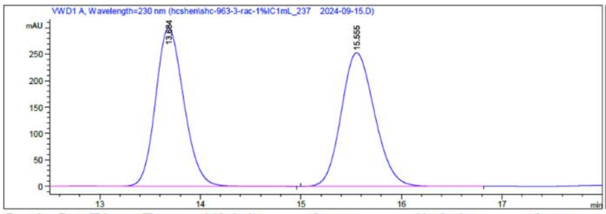

Peak #	RetTime [min]	Type	Width [min]	Area [mAU*s]	Height [mAU]	Area %
1	13.684	VB R	0.3029	5854.08301	300.05157	50.0787
2	15.555	BB	0.3564	5835.67773	253.06987	49.9213

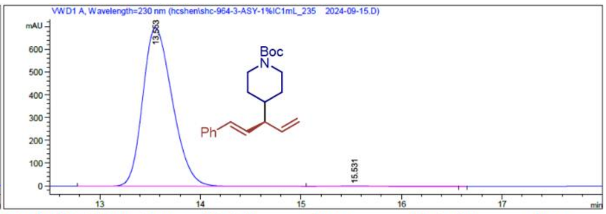

Peak #	RetTime [min]	Type	Width [min]	Area [mAU*s]	Height [mAU]	Area %
1	13.553	BV R	0.3181	1.42862e4	696.10797	99.6349
2	15.531	VB E	0.3437	52.34850	1.94137	0.3651

***tert*-butyl (*R*)-4-(7-phenylhept-1-en-4-yn-3-yl)piperidine-1-carboxylate (20)**

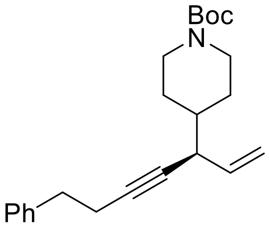

Prepared following **General Procedure A** using (*S*)-**L1**. Purification by flash column chromatography (Hexane/Ethyl acetate/DCM = 10/1/1) gave the title compound (40.7 mg, 58%, 99:1 e.r.).

¹H NMR (500 MHz, $CDCl_3$) δ 7.32 – 7.25 (m, 2H), 7.22-7.19 (m, 3H), 5.69 (ddd, J = 16.8, 10.0, 6.6 Hz, 1H), 5.22 (dt, J = 17.0, 1.6 Hz, 1H), 5.10 (dt, J = 10.0, 1.5 Hz, 1H), 4.10 (s, 2H), 2.91 (ddd, J = 6.4, 4.4, 2.2 Hz, 1H), 2.82 (t, J = 7.4 Hz, 2H), 2.61 (s, 2H), 2.51 (td, J = 7.4, 2.2 Hz, 2H), 1.68 – 1.57 (m, 2H), 1.46 (s, 9H), 1.49 – 1.42 (m, 1H), 1.30 – 1.14 (m, 2H) ppm. **¹³C NMR** (126 MHz, $CDCl_3$) δ 155.0, 140.9, 136.7, 128.6, 128.4, 126.3, 116.4, 84.2, 79.5, 79.4, 43.9, 41.5, 40.6, 35.5, 30.0, 28.6, 21.1 ppm. **Specific rotation** $[\alpha]_D^{24}$ = +25 (c = 1.2, CH_2Cl_2). **HRMS** (ESI) m/z calculated for $C_{23}H_{31}NO_2Na$ $[M+Na]^+$, 376.2247, found: 376.2252. **IR (neat)** 2955, 1693, 1422, 1277, 1033, 750 cm^{-1}. **HPLC conditions**: Chiral column IC, hexane: isopropanol = 99:1, flow rate = 1 mL/min, wavelength = 210 nm, t_R = 9.4 min for major isomer.

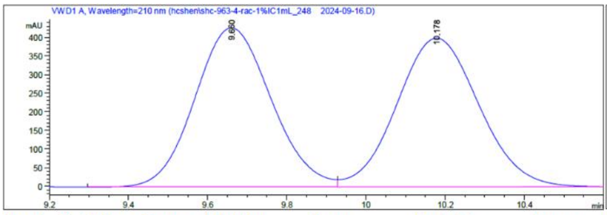

Peak #	RetTime [min]	Type	Width [min]	Area [mAU*s]	Height [mAU]	Area %
1	9.660	BV	0.2090	5734.09570	427.33804	49.8340
2	10.178	VB	0.2239	5772.29834	399.37860	50.1660

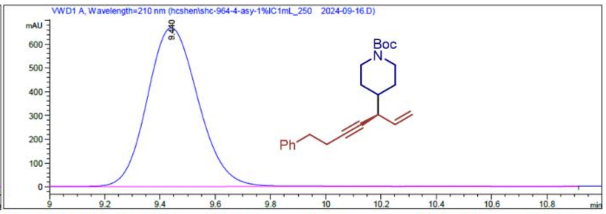

Peak #	RetTime [min]	Type	Width [min]	Area [mAU*s]	Height [mAU]	Area %
1	9.440	VV R	0.2016	8741.28418	666.87347	100.0000

methyl (*R*)-4-(1-cyclohexylallyl)benzoate (21)

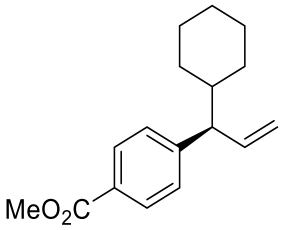

Prepared following **General Procedure A** using (*S*)-**L1**. Purification by flash column chromatography (Hexane/Ethyl acetate = 10/1) gave the title compound (27.6 mg, 45%, >99:1 e.r.).

^{1}H NMR (500 MHz, $CDCl_3$) δ 8.00 – 7.92 (m, 2H), 7.25 – 7.19 (m, 2H), 5.96 (ddd, J = 16.8, 10.2, 9.1 Hz, 1H), 5.07 – 4.98 (m, 2H), 3.90 (s, 3H), 2.99 (t, J = 9.1 Hz, 1H), 1.88 (dtd, J = 13.5, 4.1, 2.3 Hz, 1H), 1.74 (ddt, J = 12.9, 5.0, 2.4 Hz, 1H), 1.65 – 1.56 (m, 3H), 1.41 – 1.33 (m, 1H), 1.28 – 1.06 (m, 3H), 0.99 – 0.86 (m, 1H), 0.85 – 0.74 (m, 1H) ppm. **^{13}C NMR** (126 MHz, $CDCl_3$) δ 167.3, 149.8, 140.4, 129.9, 128.1, 128.1, 115.8, 57.7, 52.1, 42.3, 31.4, 31.3, 26.6, 26.4, 26.4 ppm. **Specific rotation** $[\alpha]_D^{24}$ = +20 (c = 0.9, CH_2Cl_2). **HRMS** (EI) m/z calculated for $C_{17}H_{22}O_2$ $[M]^+$, 258.1614, found: 258.1617. **IR (neat)** 2925, 1722, 1608, 1278, 1109, 770 cm^{-1}. **HPLC conditions**: Chiral column IC, hexane: isopropanol = 99:1, flow rate = 1 mL/min, wavelength = 254 nm, t_R = 7.6 min for major isomer, t_R = 8.5 min for minor isomer.

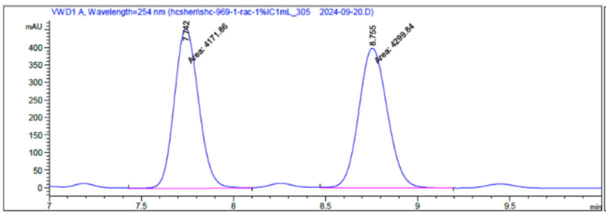

Peak #	RetTime [min]	Type	Width [min]	Area [mAU*s]	Height [mAU]	Area %
1	7.742	MM	0.1530	4171.86426	454.39688	49.2447
2	8.755	MM	0.1800	4299.83936	398.12634	50.7553

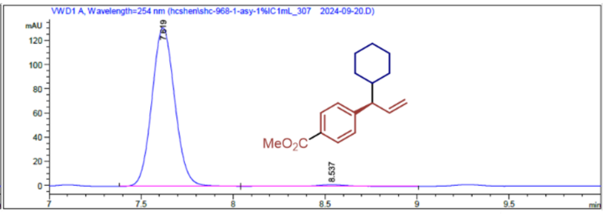

Peak #	RetTime [min]	Type	Width [min]	Area [mAU*s]	Height [mAU]	Area %
1	7.619	BV	0.1356	1130.20850	131.05887	98.7224
2	8.537	VB	0.1792	14.62675	1.20711	1.2776

methyl (*R*)-4-(1-(tetrahydro-2H-pyran-4-yl)allyl)benzoate (22)

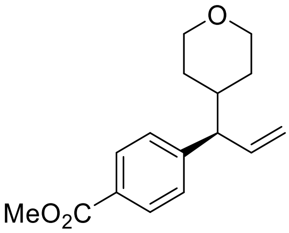

Prepared following **General Procedure A** using (*S*)-**L1**. Purification by flash column chromatography (Hexane/Ethyl acetate/DCM = 10/1/1) gave the title compound (31.3 mg, 60%, >99:1 e.r.).

^{1}H NMR (500 MHz, $CDCl_3$) δ 8.01 – 7.94 (m, 2H), 7.26 – 7.20 (m, 2H), 5.93 (ddd, J = 16.8, 10.2, 9.2 Hz, 1H), 5.11 – 5.03 (m, 2H), 4.03 – 3.96 (m, 1H), 3.90 (s, 3H), 3.88-3.84 (m, 1H), 3.41 – 3.32 (m, 1H), 3.31 – 3.22 (m, 1H), 3.00 (t, J = 9.3 Hz, 1H), 1.88 – 1.74 (m, 2H), 1.37 – 1.24 (m, 1H), 1.22 – 1.13 (m, 2H) ppm. **^{13}C NMR** (126 MHz, $CDCl_3$) δ 167.1, 148.6, 139.3, 130.0, 128.5, 128.0, 116.6, 68.1, 68.0, 57.4, 52.2, 39.6, 31.58, 31.46 ppm. **Specific rotation** $[\alpha]_D^{24}$ = +80 (c = 0.6, CH_2Cl_2). **HRMS** (EI) m/z calculated for $C_{16}H_{20}O_3$ $[M]^+$, 260.1407, found: 260.1404. **IR (neat)** 2948, 1720, 1608, 1435, 1278, 1112 771 cm^{-1}. **HPLC conditions**: Chiral column IC, hexane: isopropanol = 95:5, flow rate = 1 mL/min, wavelength = 254 nm, t_R = 24.5 min for major isomer, t_R = 27.3 min for minor isomer.

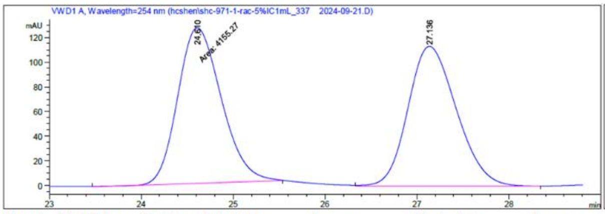

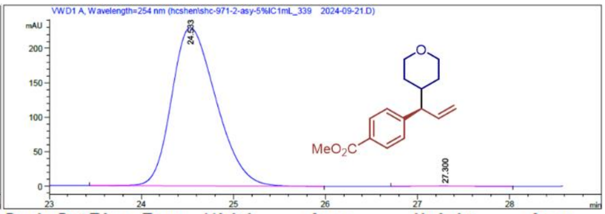

Peak #	RetTime [min]	Type	Width [min]	Area [mAU*s]	Height [mAU]	Area %
1	24.610	MM	0.5500	4155.27197	125.90963	50.0121
2	27.136	VV	0.5737	4153.25488	113.17857	49.9879

Peak #	RetTime [min]	Type	Width [min]	Area [mAU*s]	Height [mAU]	Area %
1	24.533	BB	0.5187	7699.10889	229.13124	99.7623
2	27.300	BB	0.4028	18.34344	5.39109e-1	0.2377

methyl (*R*)-4-(1-(2,3-dihydro-1H-inden-2-yl)allyl)benzoate (23)

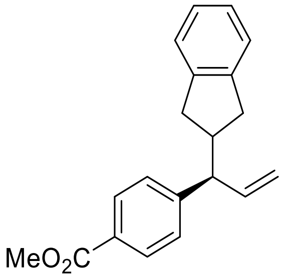

Prepared following **General Procedure A** using (*S*)-**L1**. Purification by flash column chromatography (Hexane/Ethyl acetate/DCM = 10/1/1) gave the title compound (24.5 mg, 42%, >99:1 e.r.).

^{1}H NMR (500 MHz, $CDCl_3$) δ 8.06 – 7.96 (m, 2H), 7.35 – 7.28 (m, 2H), 7.24 – 7.19 (m, 1H), 7.18 – 7.06 (m, 3H), 6.09 – 5.99 (m, 1H), 5.12 – 5.08 (m, 2H), 3.93 (s, 3H), 3.32 (dd, J = 10.1, 8.4 Hz, 1H), 3.13 (dd, J = 15.1, 7.1 Hz, 1H), 2.94 – 2.77 (m, 2H), 2.70 (dd, J = 15.9, 7.5 Hz, 1H), 2.58 – 2.45 (m, 1H) ppm. **^{13}C NMR** (126 MHz, $CDCl_3$) δ 167.2, 149.4, 143.0, 140.6, 130.1, 128.5, 128.0, 126.4, 124.5, 124.5, 115.8, 56.3, 52.2, 44.5, 38.1, 38.0 ppm. **Specific rotation** $[\alpha]_D^{24}$ = +32 (c = 0.9, CH_2Cl_2). **HRMS** (EI) m/z calculated for $C_{20}H_{20}O_2$ $[M]^+$, 292.1458, found: 292.1455. **IR (neat)** 2948, 1721, 1608, 1435, 1278, 1108, 745 cm^{-1}. **HPLC conditions**: Chiral column IA, hexane: isopropanol = 95:5, flow rate = 1 mL/min, wavelength = 254 nm, t_R = 6.7 min for major isomer.

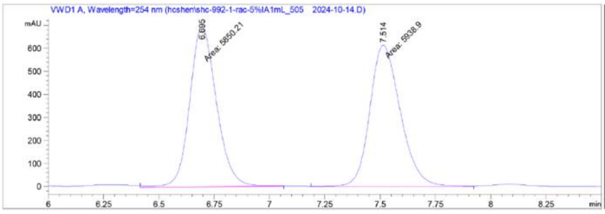

Peak #	RetTime [min]	Type	Width [min]	Area [mAU*s]	Height [mAU]	Area %
1	6.695	MM	0.1402	5850.20801	695.25214	49.6238
2	7.514	MM	0.1608	5938.90479	615.50806	50.3762

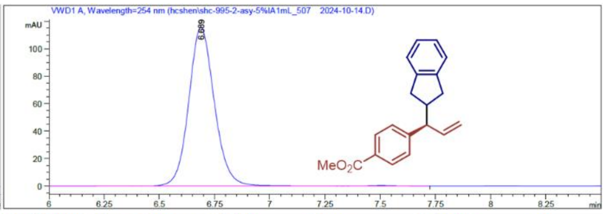

Peak #	RetTime [min]	Type	Width [min]	Area [mAU*s]	Height [mAU]	Area %
1	6.689	BV R	0.1273	940.88171	115.18623	100.0000

methyl (*R*)-4-(1-cyclobutylallyl)benzoate (24)

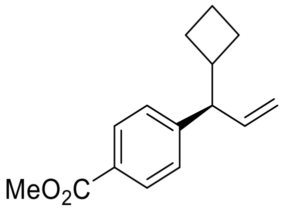

Prepared following **General Procedure A** using (*S*)-**L1**. Purification by flash column chromatography (Hexane/Ethyl acetate/DCM = 10/1/1) gave the title compound (11.1 mg, 24%, >99:1 e.r.).

^{1}H NMR (500 MHz, $CDCl_3$) δ 7.98 – 7.92 (m, 2H), 7.24 – 7.20 (m, 2H), 5.86 (ddd, J = 17.1, 10.3, 7.5 Hz, 1H), 5.11 – 4.93 (m, 2H), 3.90 (s, 3H), 3.29 – 3.19 (m, 1H), 2.70 – 2.57 (m, 1H), 2.12 (dqd, J = 7.6, 4.9, 1.7 Hz, 1H), 1.89 – 1.73 (m, 4H), 1.66 – 1.55 (m, 1H) ppm. **^{13}C NMR** (126 MHz, $CDCl_3$) δ 167.3, 148.5, 139.4, 129.8, 128.3, 128.0, 115.3, 56.9, 52.1, 39.8, 27.5, 26.9, 17.8 ppm. **Specific rotation** $[\alpha]_D^{24}$ = +8 (c = 0.3, CH_2Cl_2). **HRMS** (EI) m/z calculated for $C_{15}H_{18}O_2$ $[M]^+$, 260.1301, found: 230.1299. **IR (neat)** 2954, 1723, 1609, 1435, 1278, 1112, 710 cm^{-1}. **HPLC conditions**: Chiral column IC, hexane: isopropanol = 99:1, flow rate = 1 mL/min, wavelength = 254 nm, t_R = 8.8 min for major isomer, t_R = 7.8 min for minor isomer.

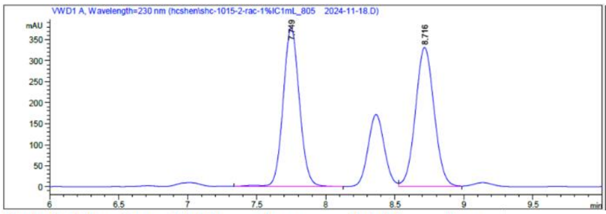

Peak #	RetTime [min]	Type	Width [min]	Area [mAU*s]	Height [mAU]	Area %
1	7.749	VB R	0.1273	3106.60669	377.19894	50.0689
2	8.716	VB	0.1474	3098.05542	329.95044	49.9311

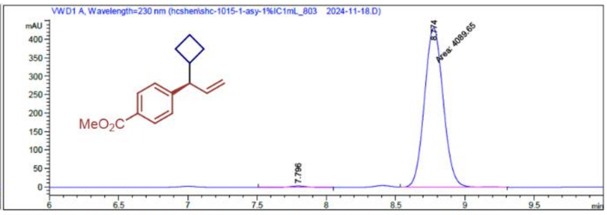

Peak #	RetTime [min]	Type	Width [min]	Area [mAU*s]	Height [mAU]	Area %
1	7.796	BB	0.1258	26.15874	3.25534	0.6356
2	8.774	MM	0.1575	4089.65381	432.83688	99.3644

methyl (*R*)-4-(1-cyclododecylallyl)benzoate (25)

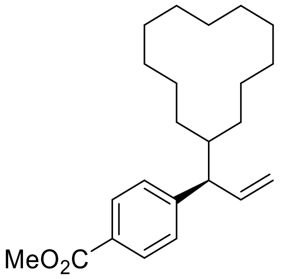

Prepared following **General Procedure A** using (*S*)-**L1**. Purification by flash column chromatography (Hexane/Ethyl acetate/DCM = 10/1/1) gave the title compound (38.7 mg, 42%, 96:4 e.r.).

^{1}H NMR (500 MHz, $CDCl_3$) δ 8.00 – 7.91 (m, 2H), 7.27 – 7.21 (m, 2H), 5.93 (ddd, J = 16.8, 10.3, 9.2 Hz, 1H), 5.07 – 5.00 (m, 2H), 3.90 (s, 3H), 3.15 (t, J = 9.4 Hz, 1H), 1.88-1.82 (m, 1H), 1.51 – 1.06 (m, 22H). ppm. **^{13}C NMR** (126 MHz, $CDCl_3$) δ 167.3, 150.3, 140.9, 129.9, 128.1, 128.0, 115.6, 54.6, 52.1, 39.9, 26.3, 26.2, 25.6, 25.5, 25.1, 23.2, 23.1, 23.1, 22.9, 21.3, 21.2 ppm. **Specific rotation** $[\alpha]_D^{24}$ = +67 (c = 0.5, CH_2Cl_2). **HRMS** (GC-EI) m/z calculated for $C_{23}H_{35}O_2$ $[M+H]^+$, 343.2632, found: 343.2625. **IR (neat)** 2933, 1723, 1608, 1277, 1109, 711 cm^{-1}. **HPLC conditions**: Chiral column IA, hexane: isopropanol = 98:2, flow rate = 1 mL/min, wavelength = 254 nm, t_R = 5.3 min for major isomer.

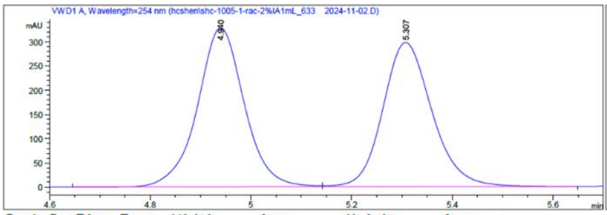

Peak #	RetTime [min]	Type	Width [min]	Area [mAU*s]	Height [mAU]	Area %
1	4.940	BV	0.0988	2125.24097	328.46307	51.1833
2	5.307	VB	0.1042	2026.97424	297.75458	48.8167

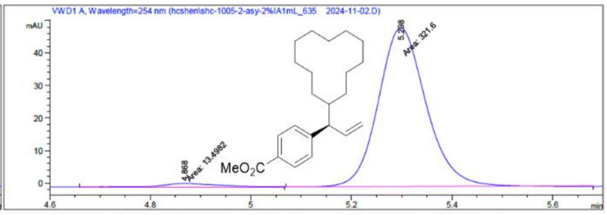

Peak #	RetTime [min]	Type	Width [min]	Area [mAU*s]	Height [mAU]	Area %
1	4.868	MM	0.1765	13.49816	1.27444	4.0281
2	5.298	MM	0.1097	321.60013	48.85316	95.9719

4-((3*S*,4*R*)-3-methyl-4-phenylhex-5-en-1-yl)-1,1'-biphenyl (27)

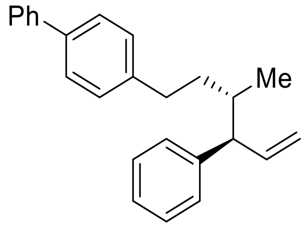

Prepared following **General Procedure B** using (*R*)-**26** and (*S*)-**L1**. Purification by flash column chromatography (20% toluene in *n*-hexane) gave the title compound (25.0 mg, 38%, >99:1 e.r. (99:1 e.r. for minor isomer), 72:28 d.r.).

^{1}H NMR (500 MHz, $CDCl_3$) δ 7.61 – 7.55 (m, 2H), 7.51 – 7.45 (m, 2H), 7.43 (dd, J = 8.4, 7.0 Hz, 2H), 7.36 – 7.26 (m, 3H), 7.23 – 7.16 (m, 1H), 7.17 – 7.13 (m, 4H), 6.05 – 5.94 (m, 1H), 5.11 – 5.06 (m, 1H), 5.06-5.05 (m, 1H), 3.13 (t, J = 8.7 Hz, 1H), 2.75 – 2.69 (m, 1H), 2.58 – 2.52 (m, 1H), 1.94 – 1.86 (m, 1H), 1.73 – 1.61 (m, 1H), 1.40 – 1.32 (m, 1H), 1.03 (d, J = 6.7 Hz, 3H) ppm. **^{13}C NMR** (126 MHz, $CDCl_3$) δ 144.2, 141.9, 141.3, 140.5, 138.7, 128.9, 128.8, 128.5, 128.1, 127.1, 126.2, 115.6, 56.6, 37.0, 36.4, 33.0, 17.2 ppm. **Specific rotation** $[\alpha]_D^{24}$ = +22 (c = 0.36, CH_2Cl_2). **HRMS** (EI) m/z calculated for $C_{28}H_{30}$ $[M]^+$, 326.2029, found: 326.2024. **IR (neat)** 3059, 3026, 2926, 1636, 1600, 1483, 911, 760, 698 cm^{-1}. The e.r. was determined after hydroboration/oxidation to alcohol. **HPLC conditions**: Chiral column IB, hexane: isopropanol = 90:10, flow rate = 0.5 mL/min, wavelength = 254 nm, t_R = 19.4 min for major isomer, t_R = 21.9 min for minor isomer.

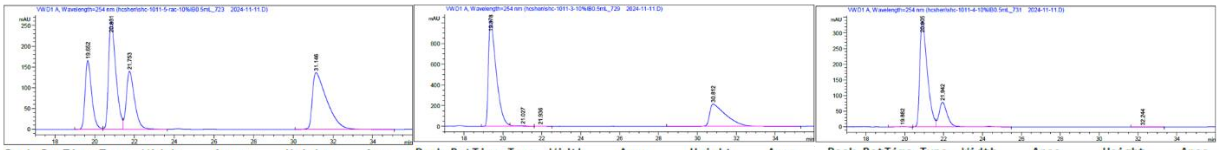

Peak #	RetTime [min]	Type	Width [min]	Area [mAU*s]	Height [mAU]	Area %
1	19.652	BV	0.3538	3842.71313	165.52148	17.8865
2	20.831	VV	0.3906	6715.61035	260.87286	31.2588
3	21.753	VB	0.4309	4020.09863	139.48280	18.7121
4	31.146	BB	0.7249	6905.51172	136.67038	32.1427

Peak #	RetTime [min]	Type	Width [min]	Area [mAU*s]	Height [mAU]	Area %
1	19.378	BV R	0.4141	2.87370e4	1025.99451	69.2750
2	21.027	VV E	0.4929	174.13321	4.97403	0.4198
3	21.936	VB E	0.3620	40.19152	1.37242	0.0969
4	30.812	VB R	0.8101	1.25312e4	214.96626	30.2084

Peak #	RetTime [min]	Type	Width [min]	Area [mAU*s]	Height [mAU]	Area %
1	19.882	BB	0.3428	26.02444	9.58914e-1	0.2268
2	20.905	BV	0.4074	9099.56250	337.85269	79.2953
3	21.942	VV R	0.4357	2340.69238	79.05348	20.3972
4	32.244	BB	0.4779	9.25857	2.31677e-1	0.0807

4-((*3S*,*4S*)-3-methyl-4-phenylhex-5-en-1-yl)-1,1'-biphenyl (28)

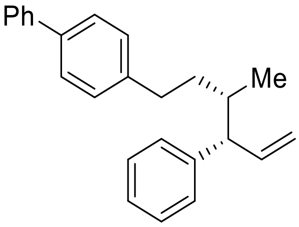

Prepared following **General Procedure B** using (*R*)-**26** and (*R*)-**L1**. Purification by flash column chromatography (20% toluene in *n*-hexane) gave the title compound (27.6 mg, 42%, >99:1 e.r. (99:1 e.r. for minor isomer), 20:80 d.r.).

^{1}H NMR (500 MHz, $CDCl_3$) δ 7.64 – 7.58 (m, 2H), 7.57 – 7.50 (m, 2H), 7.47 – 7.43 (m, 2H), 7.38 – 7.27 (m, 3H), 7.29 – 7.24 (m, 2H), 7.24 – 7.15 (m, 3H), 6.06 – 5.98 (m, 1H), 5.10 – 5.08 (m, 1H), 5.06 (d, J = 0.7 Hz, 1H), 3.09 (t, J = 8.8 Hz, 1H), 2.84 – 2.78 (m, 1H), 2.63 – 2.57 (m, 1H), 2.01 – 1.88 (m, 2H), 1.50 – 1.42 (m 1H), 0.87 (d, J = 6.7 Hz, 3H) ppm. **^{13}C NMR** (126 MHz, $CDCl_3$) 144.0, 142.1, 141.3, 141.2, 138.8, 128.9, 128.9, 128.8, 128.5, 128.2, 127.2, 127.1, 126.2, 115.4, 57.1, 37.2, 36.3, 33.0, 17.6 ppm. **Specific rotation** $[\alpha]_D^{24}$ = -42 (c = 0.42, CH_2Cl_2). **HRMS** (EI) m/z calculated for $C_{28}H_{30}$ $[M]^+$, 326.2029, found: 326.2024. **IR (neat)** 3059, 3026, 2925, 1636, 1600, 1487, 911, 760, 698 cm^{-1}. The e.r. was determined after hydroboration/oxidation to alcohol. **HPLC conditions**: Chiral column IB, hexane: isopropanol = 90:10, flow rate = 0.5 mL/min, wavelength = 254 nm, t_R = 20.9 min for major isomer, t_R = 32.2 min for minor isomer.

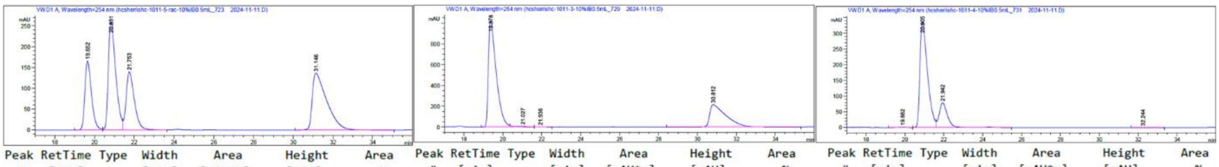

Peak #	RetTime [min]	Type	Width [min]	Area [mAU*s]	Height [mAU]	Area %
1	19.652	BV	0.3538	3842.71313	165.52148	17.8865
2	20.831	VV	0.3906	6715.61035	260.87286	31.2588
3	21.753	VB	0.4309	4020.09863	139.48280	18.7121
4	31.146	BB	0.7249	6905.51172	136.67038	32.1427

Peak #	RetTime [min]	Type	Width [min]	Area [mAU*s]	Height [mAU]	Area %
1	19.378	BV R	0.4141	2.87370e4	1025.99451	69.2750
2	21.027	VV E	0.4929	174.13321	4.97403	0.4198
3	21.936	VB E	0.3620	40.19152	1.37242	0.0969
4	30.812	VB R	0.8101	1.25312e4	214.96626	30.2084

Peak #	RetTime [min]	Type	Width [min]	Area [mAU*s]	Height [mAU]	Area %
1	19.882	BB	0.3428	26.02444	9.58914e-1	0.2268
2	20.905	BV	0.4074	9099.56250	337.85269	79.2953
3	21.942	VV R	0.4357	2340.69238	79.05348	20.3972
4	32.244	BB	0.4779	9.25857	2.31677e-1	0.0807

4-((*S*)-3-((*R*)-1-phenylallyl)hept-6-en-1-yl)-1,1'-biphenyl (30)

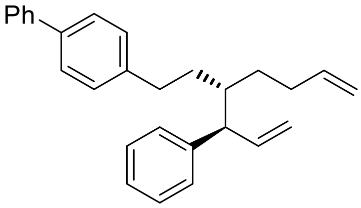

Prepared following **General Procedure C** using (*R*)-**43** and (*S*)-**L1**. Purification by flash column chromatography (20% toluene in *n*-hexane) gave the title compound (23.0 mg, 32%, >99:1 e.r. (95:5 e.r. for minor isomer), 81:19 d.r.).

^{1}H NMR (500 MHz, $CDCl_3$) δ 7.62 – 7.54 (m, 2H), 7.54 – 7.48 (m, 2H), 7.49 – 7.39 (m, 2H), 7.37 – 7.27 (m, 3H), 7.20 (ddd, J = 13.7, 8.0, 1.5 Hz, 5H), 6.02 (ddd, J = 16.8, 10.3, 9.3 Hz, 0H), 5.76 – 5.68 (m, 1H), 5.17 – 5.06 (m, 2H), 5.01 – 4.89 (m, 2H), 3.32 (t, J = 8.7 Hz, 1H), 2.71 (ddd, J = 13.6, 11.1, 5.2 Hz, 1H), 2.56 (ddd, J = 13.5, 11.0, 5.7 Hz, 1H), 2.12 – 1.98 (m, 2H), 1.92 – 1.77 (m, 2H), 1.72 – 1.59 (m, 1H), 1.48 – 1.30 (m, 2H) ppm. **^{13}C NMR** (126 MHz, $CDCl_3$) δ 144.0, 142.1, 141.3, 140.5, 139.0, 138.8, 128.9, 128.9, 128.6, 128.6, 128.1, 127.2, 127.1, 126.2, 115.8, 114.6, 53.8, 41.4, 32.3, 32.2, 30.9, 30.0 ppm. **Specific rotation** $[\alpha]_D^{24}$ = +10 (c = 0.8, CH_2Cl_2). **HRMS** (EI) m/z calculated for $C_{28}H_{30}$ $[M]^+$, 366.2342, found: 366.2336. **IR (neat)** 3060, 3027, 2925, 2857, 1639, 1600, 1487, 1451, 994, 910, 839, 698 cm^{-1}. The e.r. was determined after hydroboration/oxidation to alcohol. **HPLC conditions**: Chiral column IC, hexane: isopropanol = 90:10, flow rate = 1.0 mL/min, wavelength = 254 nm, t_R = 26.9 min for major isomer, t_R = 22.1 min for major isomer.

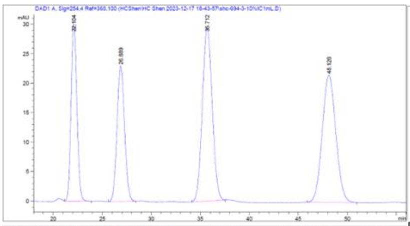

Peak #	RetTime [min]	Type	Width [min]	Area [mAU*s]	Height [mAU]	Area %
1	22.104	BB	0.6443	1246.16833	30.14376	18.8052
2	26.889	BB	0.8256	1229.31494	22.99871	18.5508
3	35.712	BB	1.0896	2065.84351	29.33322	31.1744
4	48.126	BB	1.4459	2085.40747	21.60919	31.4696

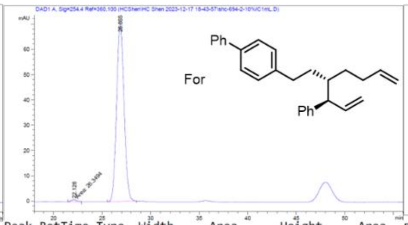

Peak #	RetTime [min]	Type	Width [min]	Area [mAU*s]	Height [mAU]	Area %
1	22.126	MM	0.6131	26.34935	7.16258e-1	0.6976
2	26.865	BB	0.8300	3751.01025	70.12582	99.3024

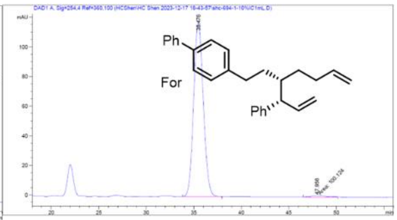

Peak #	RetTime [min]	Type	Width [min]	Area [mAU*s]	Height [mAU]	Area %
1	35.476	BB	1.1012	8275.91602	117.28199	98.8046
2	47.956	MM	2.0778	100.12354	8.03127e-1	1.1954

4-((*S*)-3-((*S*)-1-phenylallyl)hept-6-en-1-yl)-1,1'-biphenyl (31)

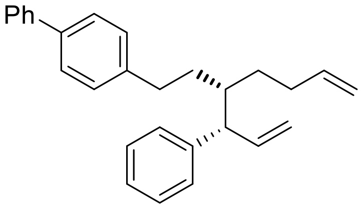

Prepared following **General Procedure G** using (*R*)-**43** and (*R*)-**L1**. Purification by flash column chromatography (20% toluene in *n*-hexane) gave the title compound (25.6 mg, 35%, >99:1 e.r. (95:5 e.r. for minor isomer), 19:81 d.r.).

^{1}H NMR (500 MHz, $CDCl_3$) δ 7.61 – 7.55 (m, 2H), 7.53 – 7.40 (m, 4H), 7.36 – 7.28 (m, 3H), 7.24 – 7.16 (m, 3.5H), 6.02 (ddd, *J* = 16.8, 10.3, 9.3 Hz, 1.5H), 5.78 – 5.66 (m, 1H), 5.15 – 5.06 (m, 2H), 5.01 – 4.89 (m, 2H), 3.32 (t, *J* = 8.7 Hz, 1H), 2.76 – 2.67 (m, 1H), 2.61 – 2.51 (m, 1H), 2.14 – 1.97 (m, 2H), 1.92 – 1.78 (m, 2H), 1.73 – 1.58 (m, 1H), 1.47 – 1.29 (m, 2H) ppm. **^{13}C NMR** (126 MHz, $CDCl_3$) δ 144.0, 142.1, 141.3, 140.5, 139.0, 138.8, 128.9(1), 128.8(6), 128.6, 128.1, 127.2, 127.1(4), 127.1(1), 126.2, 115.8, 114.6, 53.8, 41.4, 32.3, 32.2, 30.9, 30.0 ppm. **Specific rotation** $[\alpha]_D^{24}$ = −21 (c = 0.9, CH_2Cl_2). **HRMS** (MALDL) m/z calculated for $C_{28}H_{30}Na$ $[M+Na]^+$, 389.2240, found: 389.2244. **IR (neat)** 3060, 3026, 2924, 2856, 1639, 1600, 1487, 1451, 1144, 993, 910, 761, 698 cm^{-1}. The e.r. was determined after hydroboration/oxidation to alcohol. **HPLC conditions**: Chiral column IC, hexane: isopropanol = 90:10, flow rate = 1.0 mL/min, wavelength = 254 nm, t_R = 48.0 min for major isomer, t_R = 35.5 min for major isomer.

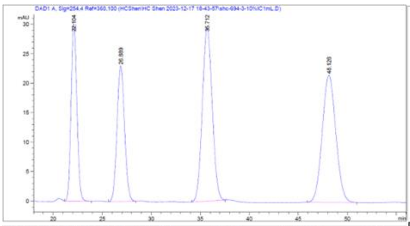

Peak #	RetTime [min]	Type	Width [min]	Area [mAU*s]	Height [mAU]	Area %
1	22.104	BB	0.6443	1246.16833	30.14376	18.8052
2	26.889	BB	0.8256	1229.31494	22.99871	18.5508
3	35.712	BB	1.0896	2065.84351	29.33322	31.1744
4	48.126	BB	1.4459	2085.40747	21.60919	31.4696

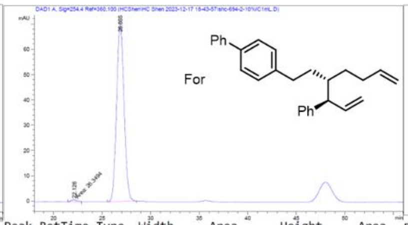

Peak #	RetTime [min]	Type	Width [min]	Area [mAU*s]	Height [mAU]	Area %
1	22.126	MM	0.6131	26.34935	7.16258e-1	0.6976
2	26.865	BB	0.8300	3751.01025	70.12582	99.3024

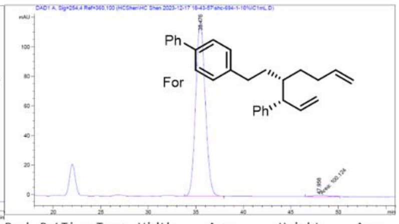

Peak #	RetTime [min]	Type	Width [min]	Area [mAU*s]	Height [mAU]	Area %
1	35.476	BB	1.1012	8275.91602	117.28199	98.8046
2	47.956	MM	2.0778	100.12354	8.03127e-1	1.1954

2-((*3R,4R*)-3-phenethyl-4-phenylhex-5-en-1-yl)-1,3-dioxolane (33)

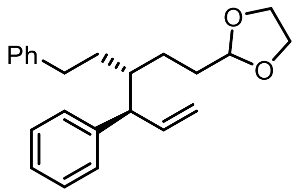

Prepared following **General Procedure C** using (*R*)-**32**[12] and (*S*)-**L1**. *Boron-to-zinc tramsformation at 60 ℃ for 7 hours.* Purification by flash column chromatography (20% ethyl acetate in *n*-hexane) gave the title compound (24.2 mg, 36%, >99:1 e.r. (96:4 e.r. for minor isomer), 73:27 d.r.).

^{1}H NMR (500 MHz, $CDCl_3$) δ 7.28 (t, J = 7.5 Hz, 2H), 7.25 – 7.15 (m, 3H), 7.18 – 7.12 (m, 3H), 7.05 – 6.99 (m, 2H), 5.98 (ddd, J = 16.9, 10.2, 9.3 Hz, 1H), 5.12 – 5.03 (m, 2H), 4.82 (t, J = 4.6 Hz, 1H), 4.01 – 3.93 (m, 2H), 3.89 – 3.82 (m, 2H), 3.25 (t, J = 9.0 Hz, 1H), 2.58 (ddd, J = 13.5, 10.8, 5.4 Hz, 1H), 2.47 (ddd, J = 13.5, 10.6, 6.0 Hz, 1H), 1.90 – 1.80 (m, 1H), 1.79 – 1.54 (m, 4H), 1.54 – 1.39 (m, 2H) ppm. **^{13}C NMR** (126 MHz, $CDCl_3$) δ 143.9, 142.7, 140.7, 128.6, 128.5, 128.4, 128.1, 126.3, 125.7, 115.7, 105.0, 65.0, 65.0, 54.1, 41.5, 32.6, 32.4, 30.8, 24.5 ppm. **Specific rotation** $[\alpha]_D^{24}$ = +47 (c = 0.6, CH_2Cl_2). **HRMS** (ESI) m/z calculated for $C_{23}H_{29}O_2$ $[M+H]^+$, 337.2162, found: 337.2167. **IR (neat)** 3025, 2929, 1601, 1453, 1141, 1032, 699 cm^{-1}. The e.r. was determined after hydroboration/oxidation to alcohol. **HPLC conditions**: Chiral column IC, hexane: isopropanol = 90:10, flow rate = 1.0 mL/min, wavelength = 210 nm, t_R = 17.2 min for major isomer, t_R = 11.8 min for major isomer.

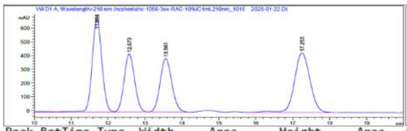

Peak #	RetTime [min]	Type	Width [min]	Area [mAU*s]	Height [mAU]	Area %
1	11.698	VV R	0.2873	1.25281e4	675.89313	29.8262
2	12.573	VB	0.3091	8338.11816	422.02371	19.8510
3	13.561	BB	0.3407	8547.63477	389.10098	20.3498
4	17.253	VB R	0.4444	1.25897e4	432.47147	29.9729

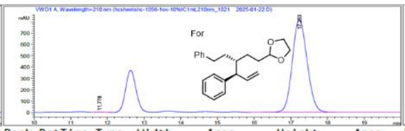

Peak #	RetTime [min]	Type	Width [min]	Area [mAU*s]	Height [mAU]	Area %
1	11.778	VB	0.2925	63.74561	3.29754	0.2756
2	17.233	VV R	0.4376	2.30638e4	820.32574	99.7244

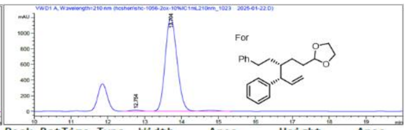

Peak #	RetTime [min]	Type	Width [min]	Area [mAU*s]	Height [mAU]	Area %
1	12.754	BB	0.3012	307.16187	16.31539	1.1257
2	13.704	BV R	0.3511	2.69792e4	1191.01941	98.8743

2-((*3R,4S*)-3-phenethyl-4-phenylhex-5-en-1-yl)-1,3-dioxolane (34)

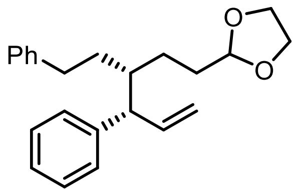

Prepared following **General Procedure C** using (*R*)-**32** and (*R*)-**L1**. *Boron-to-zinc tramsformation at 60 ℃ for 7 hours.* Purification by flash column chromatography (20% ethyl acetate in *n*-hexane) gave the title compound (23.8 mg, 35%, 99:1 e.r. (>99:1 e.r. for minor isomer), 35:65 d.r.).

^{1}H NMR (500 MHz, $CDCl_3$) δ 7.32 – 7.23 (m, 4H), 7.22 – 7.11 (m, 6H), 5.99 (ddd, J = 16.7, 10.5, 9.3 Hz, 1H), 5.12 – 5.05 (m, 2H), 4.72 (t, J = 4.8 Hz, 1H), 3.95 – 3.89 (m, 2H), 3.84 – 3.77 (m, 2H), 3.27 (t, J = 8.9 Hz, 1H), 2.68 (ddd, J = 13.4, 11.1, 5.1 Hz, 1H), 2.51 (ddd, J = 13.3, 11.0, 5.7 Hz, 1H), 1.91 – 1.74 (m, 2H), 1.71 – 1.55 (m, 3H), 1.50 – 1.40 (m, 1H), 1.39 – 1.31 (m, 1H) ppm. **^{13}C NMR** (126 MHz, $CDCl_3$) δ 143.9, 142.9, 140.4, 128.6, 128.5, 128.4, 128.0, 126.3, 125.8, 115.8, 105.0, 65.0, 64.9, 53.9, 41.7, 32.8, 32.3, 30.9, 25.0 ppm. **Specific rotation** $[\alpha]_D^{24}$ = -44 (c = 0.6, CH_2Cl_2). **HRMS** (EI) m/z calculated for $C_{23}H_{27}O_2$ $[M-H]^+$, 335.2006, found: 335.2001. **IR (neat)** 2948, 1603, 1453, 1141, 1033, 700 cm^{-1}. The e.r. was determined after hydroboration/oxidation to alcohol. **HPLC conditions**: Chiral column IC, hexane: isopropanol = 90:10, flow rate = 1.0 mL/min, wavelength = 210 nm, t_R = 13.7 min for major isomer, t_R = 12.8 min for major isomer.

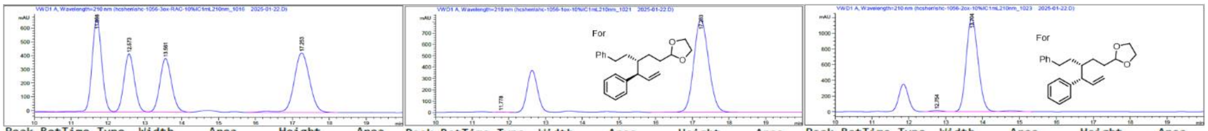

Peak #	RetTime [min]	Type	Width [min]	Area [mAU*s]	Height [mAU]	Area %
1	11.698	VV R	0.2873	1.25281e4	675.89313	29.8262
2	12.573	VB	0.3091	8338.11816	422.02371	19.8510
3	13.561	BB	0.3407	8547.63477	389.10098	20.3498
4	17.253	VB R	0.4444	1.25897e4	432.47147	29.9729

Peak #	RetTime [min]	Type	Width [min]	Area [mAU*s]	Height [mAU]	Area %
1	11.778	VB	0.2925	63.74561	3.29754	0.2756
2	17.233	VV R	0.4376	2.30638e4	820.32574	99.7244

Peak #	RetTime [min]	Type	Width [min]	Area [mAU*s]	Height [mAU]	Area %
1	12.754	BB	0.3012	307.16187	16.31539	1.1257
2	13.704	BV R	0.3511	2.69792e4	1191.01941	98.8743

methyl 4-((*3S*,*5S*)-7-([1,1'-biphenyl]-4-yl)-5-hydroxyhept-1-en-3-yl)benzoate (36)

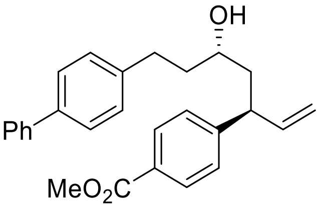

Prepared following **General Procedure D**, but **Step 2** at r.t. for 2 hours, using (*R*)-**35** and (*S*)-**L1**. Followed by oxidation. Purification by flash column chromatography (20% EtOAc in *n*-hexane) gave the title compound (41.1 mg, 51%, >95:5 d.r., >99:1 e.r.).

^{1}H NMR (500 MHz, $CDCl_3$) δ 8.00 – 7.94 (m, 2H), 7.62 – 7.56 (m, 2H), 7.55 – 7.49 (m, 2H), 7.47 – 7.40 (m, 2H), 7.37 – 7.30 (m, 1H), 7.31 – 7.23 (m, 4H), 5.92 (ddd, J = 17.0, 10.2, 8.2 Hz, 1H), 5.17 – 5.08 (m, 2H), 3.90 (s, 3H), 3.78 – 3.68 (m, 1H), 3.63 (td, J = 8.7, 5.9 Hz, 1H), 2.84 (ddd, J = 13.8, 9.6, 5.9 Hz, 1H), 2.71 (ddd, J = 13.8, 9.5, 6.7 Hz, 1H), 1.97 – 1.76 (m, 4H) ppm. **^{13}C NMR** (126 MHz, $CDCl_3$) δ 167.1, 149.9, 141.2, 141.1, 140.7, 139.0, 130.1, 128.9, 128.9, 128.4, 127.6, 127.3, 127.2, 127.1, 115.9, 69.2, 52.2, 46.6, 43.2, 39.5, 31.8 ppm. **Specific rotation** $[\alpha]_D^{24}$ = +25 (c = 0.6, CH_2Cl_2). **HRMS** (ESI) m/z calculated for $C_{27}H_{27}O_2$ [M-H_2O+H]$^+$, 383.2006, found: 383.2014. **IR (neat)** 3454, 2935, 1720, 1609, 1435, 1280, 1112, 763 cm^{-1}. **HPLC conditions**: Chiral column IC, hexane: isopropanol = 93:7, flow rate = 1 mL/min, wavelength = 254 nm, t_R = 22.9 min for major isomer.

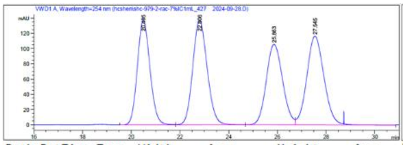

Peak #	RetTime [min]	Type	Width [min]	Area [mAU*s]	Height [mAU]	Area %
1	20.495	BV	0.5567	4957.38037	137.75595	23.3690
2	22.806	VB	0.6393	5646.88379	137.50941	26.6193
3	25.863	BV	0.7285	4909.62402	105.65298	23.1439
4	27.545	VB	0.7568	5699.59814	116.56354	26.8678

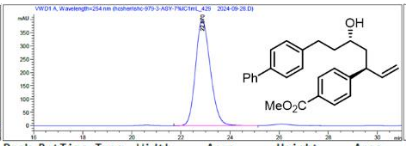

Peak #	RetTime [min]	Type	Width [min]	Area [mAU*s]	Height [mAU]	Area %
1	22.870	BV	0.6445	1.66837e4	399.48309	100.0000

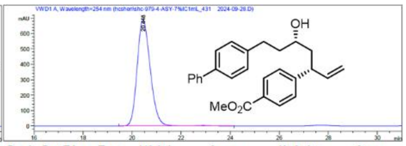

Peak #	RetTime [min]	Type	Width [min]	Area [mAU*s]	Height [mAU]	Area %
1	20.448	BV R	0.5744	2.56161e4	687.68042	100.0000

methyl 4-((*3R*,*5S*)-7-([1,1'-biphenyl]-4-yl)-5-hydroxyhept-1-en-3-yl)benzoate (35)

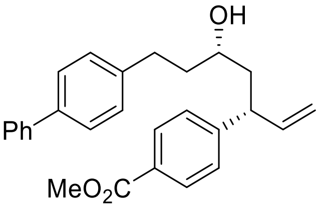

Prepared following **General Procedure D**, but **Step 2** at r.t. for 2 hours, using (*R*)-**35** and (*R*)-**L1**. Followed by oxidation. Purification by flash column chromatography (20% EtOAc in *n*-hexane) gave the title compound (47.5 mg, 59%, <95:5 d.r., >99:1 e.r.).

^{1}H NMR (500 MHz, $CDCl_3$) δ 8.03 – 7.96 (m, 2H), 7.60 – 7.54 (m, 2H), 7.52 – 7.46 (m, 2H), 7.47 – 7.39 (m, 2H), 7.37 – 7.30 (m, 1H), 7.32 – 7.26 (m, 2H), 7.23 – 7.17 (m, 2H), 6.08 – 5.98 (m, 1H), 5.10 – 5.08 (m, 1H), 5.07 – 5.06 (m, 1H), 3.90 (s, 3H), 3.72 – 3.63 (m, 1H), 3.50-3.44 (m, 1H), 2.78 – 2.72 (m, 1H), 2.67 – 2.61 (m, 1H), 1.99 – 1.86 (m, 2H), 1.84 – 1.76 (m, 2H) ppm. **^{13}C NMR** (126 MHz, $CDCl_3$) δ 167.1, 149.0, 141.8, 141.1, 141.1, 139.0, 130.1, 128.9, 128.6, 128.0, 127.3, 127.2, 127.1, 114.9, 69.4, 52.2, 46.7, 42.9, 39.8, 31.7 ppm. **Specific rotation** $[\alpha]_D^{24}$ = -26 (c = 0.7, CH_2Cl_2). **HRMS** (ESI) m/z calculated for $C_{27}H_{27}O_2$ [M-H_2O+H]$^+$, 383.2006, found: 383.2020. **IR (neat)** 3452, 2937, 1719, 1608, 1435, 1280, 1110, 762 cm^{-1}. **HPLC conditions**: Chiral column IC, hexane: isopropanol = 93:7, flow rate = 1 mL/min, wavelength = 254 nm, t_R = 20.5 min for major isomer.

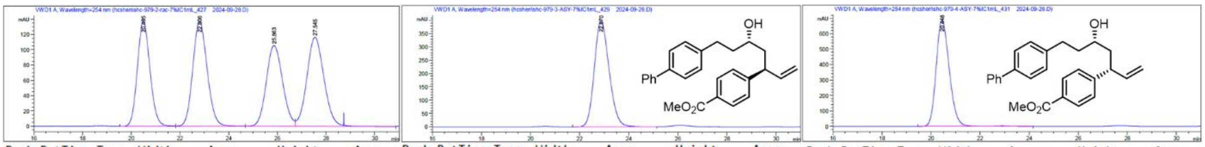

Peak #	RetTime [min]	Type	Width [min]	Area [mAU*s]	Height [mAU]	Area %
1	20.495	BV	0.5567	4957.38037	137.75595	23.3690
2	22.806	VB	0.6393	5646.88379	137.50941	26.6193
3	25.863	BV	0.7285	4909.62402	105.65298	23.1439
4	27.545	VB	0.7568	5699.59814	116.56354	26.8678

Peak #	RetTime [min]	Type	Width [min]	Area [mAU*s]	Height [mAU]	Area %
1	22.870	BV	0.6445	1.66837e4	399.48309	100.0000

Peak #	RetTime [min]	Type	Width [min]	Area [mAU*s]	Height [mAU]	Area %
1	20.448	BV R	0.5744	2.56161e4	687.68042	100.0000

(3*S*,5*S*,*E*)-1-([1,1'-biphenyl]-4-yl)-7-phenyl-5-vinylhept-6-en-3-ol (38)

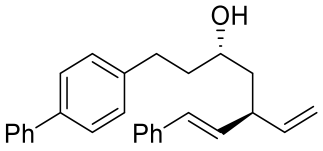

Prepared following **General Procedure D**, but **Step 2** at r.t. for 2 hours, using (*R*)-**35** and (*S*)-**L1**. Followed by oxidation. Purification by flash column chromatography (20% EtOAc in *n*-hexane) gave the title compound (36.9 mg, 50%, >95:5 d.r., 93:7 r.r., >99:1 e.r.).

^{1}H NMR (400 MHz, $CDCl_3$) δ 7.62 – 7.55 (m, 2H), 7.55 – 7.50 (m, 2H), 7.48 – 7.40 (m, 2H), 7.38 – 7.18 (m, 8H), 6.42 (d, *J* = 15.9 Hz, 1H), 6.18 (dd, *J* = 16.0, 7.6 Hz, 1H), 5.80 (ddd, *J* = 17.6, 10.3, 7.7 Hz, 1H), 5.20 – 5.07 (m, 2H), 3.85 – 3.73 (m, 1H), 3.23 – 3.10 (m, 1H), 2.92 – 2.80 (m, 1H), 2.80 – 2.68 (m, 1H), 1.90 – 1.81 (m, 2H), 1.76 – 1.69 (m, 2H), 1.63 – 1.57 (m, 1H) ppm. **^{13}C NMR** (126 MHz, $CDCl_3$) δ 141.6, 141.3, 141.2, 139.0, 137.4, 132.2, 130.6, 129.0, 128.8, 128.7, 127.4, 127.3, 127.2, 127.1, 126.3, 114.6, 69.6, 44.3, 42.5, 39.7, 31.8 ppm. **Specific rotation** $[\alpha]_D^{22}$ = +8 (c = 0.9, CH_2Cl_2). **HRMS** (ESI) m/z calculated for $C_{27}H_{29}O$ $[M+H]^+$, 369.2213, found: 369.2207. **IR (neat)** 3346, 3025, 2925, 2854, 1635, 1599, 1486, 1448, 1409, 1072, 965, 918, 747, 693 cm^{-1}. **HPLC conditions**: Chiral column IA, hexane: isopropanol = 90:10, flow rate = 1.0 mL/min, wavelength = 254 nm, t_R = 40.5 min for major isomer.

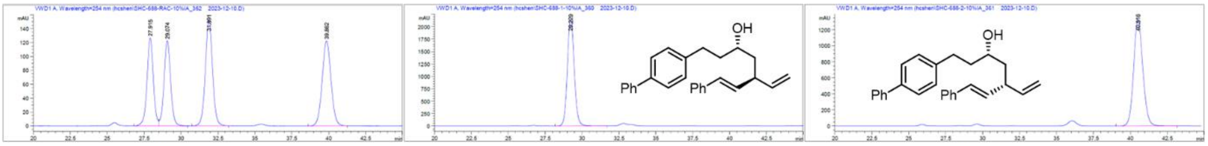

Peak #	RetTime [min]	Type	Width [min]	Area [mAU*s]	Height [mAU]	Area %
1	27.915	VV R	0.3857	3780.32227	126.52212	21.1656
2	29.074	VV R	0.3980	3789.78247	122.41310	21.2186
3	31.891	VV R	0.4184	5181.15332	152.57001	29.0088
4	39.862	VV R	0.5244	5109.39648	122.41987	28.6070

Peak #	RetTime [min]	Type	Width [min]	Area [mAU*s]	Height [mAU]	Area %
1	29.229	VV R	0.4023	7.00372e4	2145.35596	100.0000

Peak #	RetTime [min]	Type	Width [min]	Area [mAU*s]	Height [mAU]	Area %
1	40.516	VV R	0.5448	5.78601e4	1327.88098	100.0000

(3*S*,5*R*,*E*)-1-([1,1'-biphenyl]-4-yl)-7-phenyl-5-vinylhept-6-en-3-ol (39)

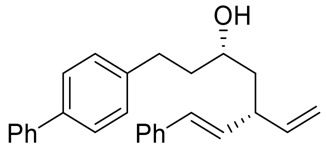

Prepared following **General Procedure D**, but **Step 2** at r.t. for 2 hours, using (*R*)-**35** and (*R*)-**L1**. Followed by oxidation. Purification by flash column chromatography (20% EtOAc in *n*-hexane) gave the title compound (41.2 mg, 56%, <5:95 d.r., 93:7 r.r., >99:1 e.r.).

^{1}H NMR (400 MHz, $CDCl_3$) δ 7.51 – 7.45 (m, 2H), 7.45 – 7.38 (m, 2H), 7.37 – 7.31 (m, 2H), 7.29 – 7.10 (m, 8H), 6.35 (d, *J* = 15.9 Hz, 1H), 6.00 (dd, *J* = 15.9, 8.2 Hz, 1H), 5.80 (ddd, *J* = 17.4, 10.2, 7.1 Hz, 1H), 5.03 (dt, *J* = 17.3, 1.4 Hz, 1H), 4.98 (dt, *J* = 10.3, 1.3 Hz, 1H), 3.75 – 3.63 (m, 1H), 3.15 – 3.03 (m, 1H), 2.81 – 2.69 (m, 1H), 2.69 – 2.56 (m, 1H), 1.79 – 1.70 (m, 2H), 1.66 – 1.56 (m, 2H), 1.54 – 1.49 (m, 1H) ppm. **^{13}C NMR** (126 MHz, $CDCl_3$) δ 141.3, 141.2, 140.6, 139.0, 137.5, 133.0, 129.9, 129.0, 128.9, 128.7, 127.4, 127.3, 127.2, 127.1, 126.3, 115.4, 69.5, 44.4, 42.6, 39.4, 31.8 ppm. **Specific rotation** $[\alpha]_D^{22}$ = −17 (c = 0.9, CH_2Cl_2). **HRMS** (ESI) m/z calculated for $C_{27}H_{29}O$ $[M+H]^+$, 369.2213, found: 369.2211. **IR (neat)** 3355, 3025, 2925, 2855, 1635, 1599, 1486, 1448, 1409, 1070, 966, 918, 841, 748, 694 cm^{-1}. **HPLC conditions**: Chiral column IA, hexane: isopropanol = 90:10, flow rate = 1.0 mL/min, wavelength = 254 nm, t_R = 29.229 min for major isomer.

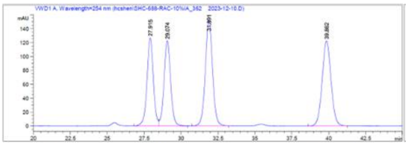

Peak #	RetTime [min]	Type	Width [min]	Area [mAU*s]	Height [mAU]	Area %
1	27.915	VV R	0.3857	3780.32227	126.52212	21.1656
2	29.074	VV R	0.3980	3789.78247	122.41310	21.2186
3	31.891	VV R	0.4184	5181.15332	152.57001	29.0088
4	39.862	VV R	0.5244	5109.39648	122.41987	28.6070

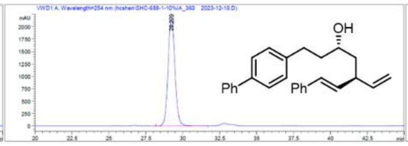

Peak #	RetTime [min]	Type	Width [min]	Area [mAU*s]	Height [mAU]	Area %
1	29.229	VV R	0.4023	7.00372e4	2145.35596	100.0000

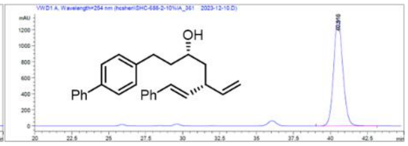

Peak #	RetTime [min]	Type	Width [min]	Area [mAU*s]	Height [mAU]	Area %
1	40.516	VV R	0.5448	5.78601e4	1327.88098	100.0000

(*R*)-4-(1-(3,5-difluorophenyl)allyl)-1-(methylsulfonyl)piperidine (45)

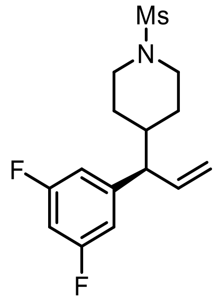

^{1}H NMR (500 MHz, $CDCl_3$) δ 6.72 – 6.62 (m, 3H), 5.85 (ddd, J = 16.9, 10.2, 9.2 Hz, 1H), 5.16 – 5.06 (m, 2H), 3.86 – 3.82 (m, 1H), 3.76 – 3.72 (m, 1H), 2.96 (t, J = 9.3 Hz, 1H), 2.76 (s, 3H), 2.65 – 2.53 (m, 2H), 1.98 – 1.93 (m, 1H), 1.69 – 1.57 (m, 1H), 1.52 – 1.43 (m, 1H), 1.36 – 1.1.19 (m, 2H) ppm. **^{13}C NMR** (126 MHz, $CDCl_3$) δ 164.3 (d, J = 13.0 Hz), 162.3 (d, J = 12.8 Hz), 146.8 (t, J = 8.5 Hz), 138.4, 117.4, 110.7 (d, J = 5.8 Hz), 110.6 (d, J = 5.7 Hz), 102.2 (t, J = 25.4 Hz), 56.5, 46.3, 46.2, 40.0, 34.8, 30.1, 30.0 ppm. **Specific rotation** $[\alpha]_D^{24}$ = +36 (c = 0.7, CH_2Cl_2). **HRMS** (ESI) m/z calculated for $C_{15}H_{20}F_2NO_2S$ $[M+H]^+$, 316.1177, found: 316.1187. **IR (neat)** 2943, 1621, 1596, 1456, 1329, 1155, 775, 518 cm^{-1}.

(*R*)-3-(3,5-difluorophenyl)-3-(1-(methylsulfonyl)piperidin-4-yl)propan-1-ol (46)

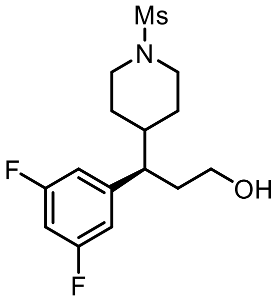

^{1}H NMR (500 MHz, $CDCl_3$) δ 6.71 – 6.62 (m, 3H), 3.84 (ddt, J = 11.8, 4.6, 2.5 Hz, 1H), 3.71 (ddt, J = 11.8, 4.6, 2.6 Hz, 1H), 3.50 (ddt, J = 8.7, 7.0, 4.3 Hz, 1H), 3.38 – 3.24 (m, 1H), 2.74 (s, 3H), 2.66 – 2.47 (m, 3H), 2.08 (dddd, J = 13.7, 8.4, 7.1, 3.8 Hz, 1H), 2.04 – 1.95 (m, 1H), 1.71 (dddd, J = 13.7, 11.6, 6.1, 4.3 Hz, 1H), 1.53 (tdt, J = 11.8, 8.4, 3.5 Hz, 1H), 1.49 – 1.41 (m, 1H), 1.35 (dtd, J = 13.1, 12.0, 4.3 Hz, 1H), 1.25 – 1.17 (m, 1H) ppm. **^{13}C NMR** (126 MHz, $CDCl_3$) δ 164.2 (d, J = 12.9 Hz), 162.2 (d, J = 12.9 Hz), 147.0 (t, J = 8.3 Hz), 111.2 (d, J = 5.7 Hz), 111.1 (d, J = 5.6 Hz), 102.3 (t, J = 25.3 Hz), 60.5, 47.5, 46.3, 41.0, 35.1, 34.7, 30.01, 29.96 ppm. **Specific rotation** $[\alpha]_D^{24}$ = +14 (c = 0.6, CH_2Cl_2). **HRMS** (ESI) m/z calculated for $C_{15}H_{22}F_2NO_3S$ $[M+H]^+$, 334.1283, found: 334.1296. **IR (neat)** 2943, 1623, 1594, 1456, 1327, 1154, 776, 517 cm^{-1}. **HPLC conditions**: Chiral column OD-H, hexane: isopropanol = 75:25, flow rate = 1.0 mL/min, wavelength = 210 nm, t_R = 14.6 min for major isomer.

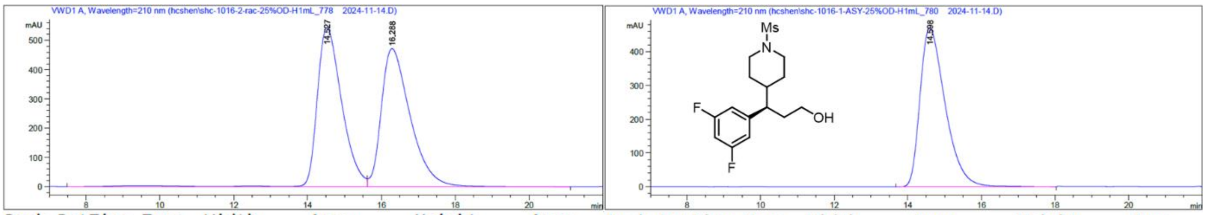

Peak #	RetTime [min]	Type	Width [min]	Area [mAU*s]	Height [mAU]	Area %
1	14.527	VV R	0.7057	2.54639e4	543.38562	49.7838
2	16.288	VV R	0.8247	2.56850e4	471.51401	50.2162

Peak #	RetTime [min]	Type	Width [min]	Area [mAU*s]	Height [mAU]	Area %
1	14.598	BB	0.7318	2.24828e4	470.81177	100.0000

tert-butyl (*R*)-4-(2-hydroxy-1-(3-methoxyphenyl)ethyl)piperidine-1-carboxylate (47)

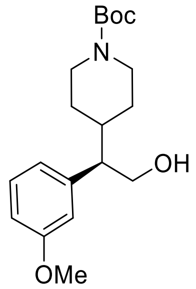

^{1}H NMR (500 MHz, $CDCl_3$) δ 7.26 – 7.23 (m, 1H), 6.79 – 6.76 (m, 2H), 6.73 – 6.72 (m, 1H), 4.13 – 3.96 (m, 2H), 3.90 (dd, J = 10.9, 4.8 Hz, 1H), 3.83 (dd, J = 10.9, 8.4 Hz, 1H), 3.80 (s, 3H), 2.75 – 2.63 (m, 1H), 2.53 (ddd, J = 13.4, 8.5, 4.1 Hz, 2H), 1.83 (dt, J = 13.0, 2.9 Hz, 1H), 1.74 (tdt, J = 12.2, 9.1, 3.5 Hz, 1H), 1.42 (s, 9H), 1.34 (dt, J = 13.1, 2.9 Hz, 1H), 1.28 – 1.16 (m, 1H), 1.00 (qd, J = 12.6, 4.4 Hz, 1H) ppm. **^{13}C NMR** (126 MHz, $CDCl_3$) δ 160.0, 154.9, 142.8, 129.8, 121.0, 114.9, 111.9, 79.4, 64.5, 55.3, 54.2, 44.0, 38.1, 30.5, 30.4, 28.6 ppm. **Specific rotation** $[\alpha]_D^{24}$ = -15 (c = 0.5, CH_2Cl_2). **HRMS** (ESI) m/z calculated for $C_{19}H_{29}NO_4Na$ $[M+Na]^+$, 358.1989, found: 358.1994. **IR (neat)** 3434, 2931, 1680, 1601, 1428, 1164, 706 cm^{-1}.

tert-butyl (*R*)-4-(2-bromo-1-(3-methoxyphenyl)ethyl)piperidine-1-carboxylate (48)

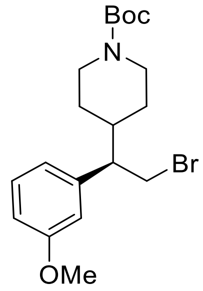

^{1}H NMR (500 MHz, $CDCl_3$) δ 7.27 – 7.21 (m, 1H), 6.80 (dd, J = 8.3, 2.5, 1H), 6.74 (d, J = 7.6, 1H), 6.71 – 6.70 (m, 1H), 4.15 – 4.00 (m, 2H), 3.81 (s, 3H), 3.74 (dd, J = 10.2, 4.8 Hz, 1H), 3.63 (dd, J = 10.2, 8.2 Hz, 1H), 2.77 – 2.65 (m, 2H), 2.58 (td, J = 12.9, 2.7 Hz, 1H), 1.90 – 1.80 (m, 2H), 1.44 – 1.38 (m, 1H), 1.42 (s, 9H), 1.24 – 1.12 (m, 1H), 1.03 (qd, J = 12.6, 4.4 Hz, 1H) ppm. **^{13}C NMR** (126 MHz, $CDCl_3$) δ 159.7, 154.8, 142.6, 129.5, 120.8, 114.7, 111.9, 79.5, 55.3, 53.1, 44.0, 39.7, 36.4, 30.5, 30.4, 29.8, 28.6 ppm. **Specific rotation** $[\alpha]_D^{22}$ = −20 (c = 1.12, CH_2Cl_2). **HRMS** (EI) m/z calculated for $C_{19}H_{29}BrNO_3$ $[M+H]^+$, 398.1325, found: 398.1327. **HPLC conditions**: Chiral column IA, hexane: isopropanol = 95:5, flow rate = 1.0 mL/min, wavelength = 254 nm, t_R = 7.6 min for major isomer.

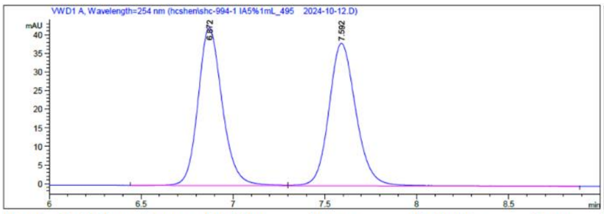

Peak #	RetTime [min]	Type	Width [min]	Area [mAU*s]	Height [mAU]	Area %
1	6.872	BV	0.1424	389.83484	42.34347	49.7326
2	7.592	VB	0.1581	394.02628	38.22464	50.2674

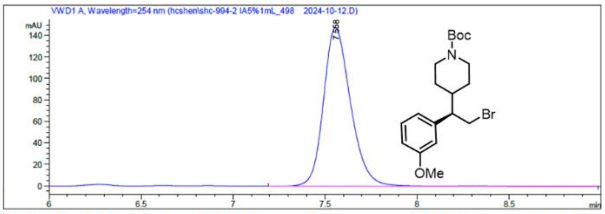

Peak #	RetTime [min]	Type	Width [min]	Area [mAU*s]	Height [mAU]	Area %
1	7.558	BV R	0.1571	1519.31616	147.74052	100.0000

(3*R*)-3-(3-methoxyphenyl)quinuclidine (49)

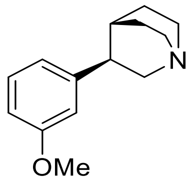

^{1}H NMR (500 MHz, $CDCl_3$) δ 7.20 (t, J = 7.9 Hz, 1H), 6.80 (d, J = 7.6 Hz, 1H), 6.74 (s, 1H), 6.70 (dd, J = 8.2, 2.5 Hz, 1H), 3.74 (s, 3H), 3.34 (ddd, J = 13.4, 10.4, 2.5 Hz, 1H), 3.10 (ddd, J = 13.7, 7.2, 2.1 Hz, 1H), 3.03 – 2.82 (m, 5H), 1.95 (d, J = 3.1 Hz, 1H), 1.81 – 1.58 (m, 3H), 1.37 (t, J = 12.0 Hz, 1H) ppm. **^{13}C NMR** (126 MHz, $CDCl_3$) δ 159.9, 145.1, 129.6, 119.9, 114.0, 111.2, 55.3, 53.3, 47.7, 47.2, 41.0, 27.7, 27.6, 20.7 ppm. **Specific rotation** $[\alpha]_D^{23}$ = −46 (c = 0.69, CH_2Cl_2). **HRMS** (EI) m/z calculated for $C_{14}H_{19}ON$ $[M]^+$, 217.1461, found: 217.1457. **IR (neat)** 3421, 2940, 2869, 1599, 1490, 1260, 1048, 781, 698 cm^{-1}.

3. REFERENCE

[1] A. F. Burchat, J. M. Chong, N. Nielsen, *J. Organomet. Chem.* **1997**, *542*, 281−283.

[2] H.-C. Shen, Z.-S. Wang, A. Noble, V. K. Aggarwal, *J. Am. Chem. Soc.* **2024**, *146*, 13719–13726.

[3] C. R. Davis, I. K. Luvaga, J. M. Ready, *J. Am. Chem. Soc.* **2021**, *143*, 4921−4927.

[4] H. Liang, J. P. Morken, *J. Am. Chem. Soc.* **2023**, *145*, 9976–9981.

[5] K. M. B. Gross, P. Beak, *J. Am. Chem. Soc.* **2001**, *123*, 315–321.

[6] SAINT v8.39.0, Bruker AXS Inc., Madison, Wisconsin, USA, **2018**.

[7] SADABS 2016/2, Bruker AXS Inc, Madison, Wisconsin, USA, **2016**.

[8] Sheldrick, G.M., *Acta Cryst.* **2014**, **C71**, 3.

[9] Sheldrick, G.M., *Acta Cryst.* **2008**, **A64**, 112.

[10] Sheldrick, G.M., *Acta Cryst.* **2015**, **A71**, 3.

[11] Dolomanov, O.V.; Bourhis, L.J.; Gildea, R.J.; Howard, J.A.K.; Puschmann, H., OLEX2: A complete structure solution, refinement and analysis program. *J. Appl. Cryst.*, **2009**, *42*, 339-341.

[12] Aichhorn, S.; Bigler, R.; Myers, E. L.; Aggarwal, V. K. *J. Am. Chem. Soc.* **2017**, *139*, 9519–9522.

Compound 26

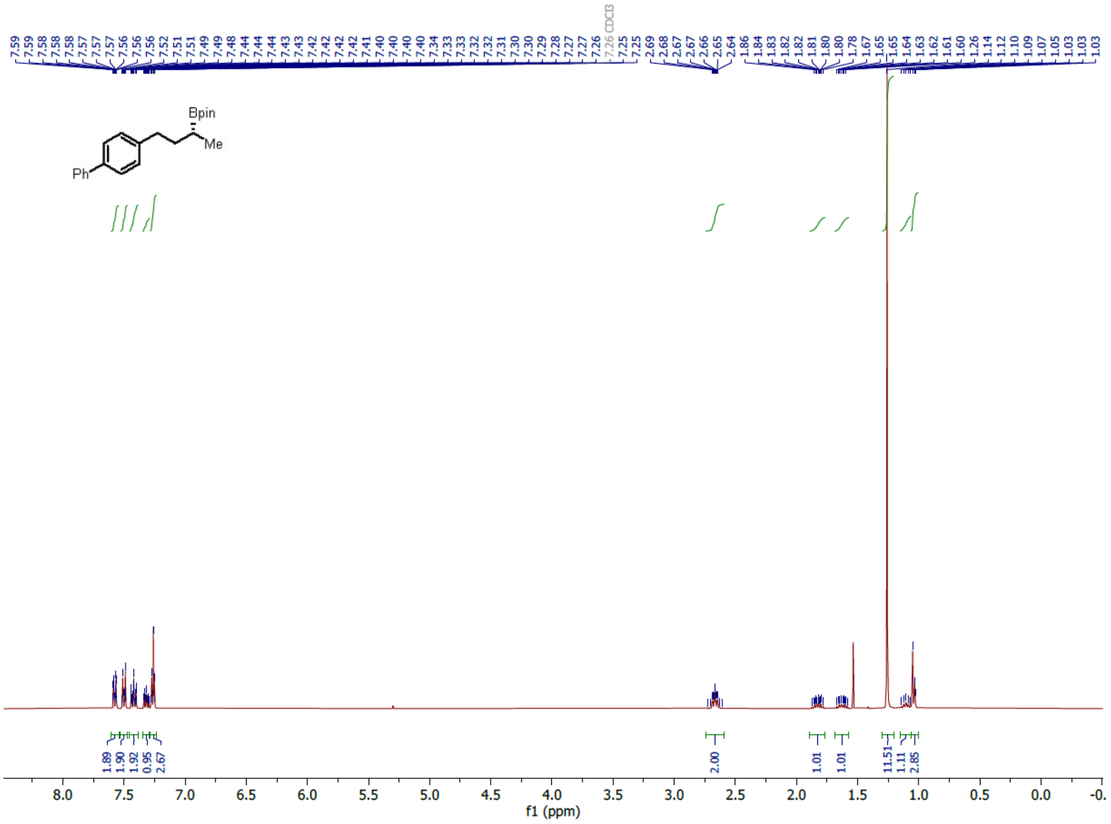

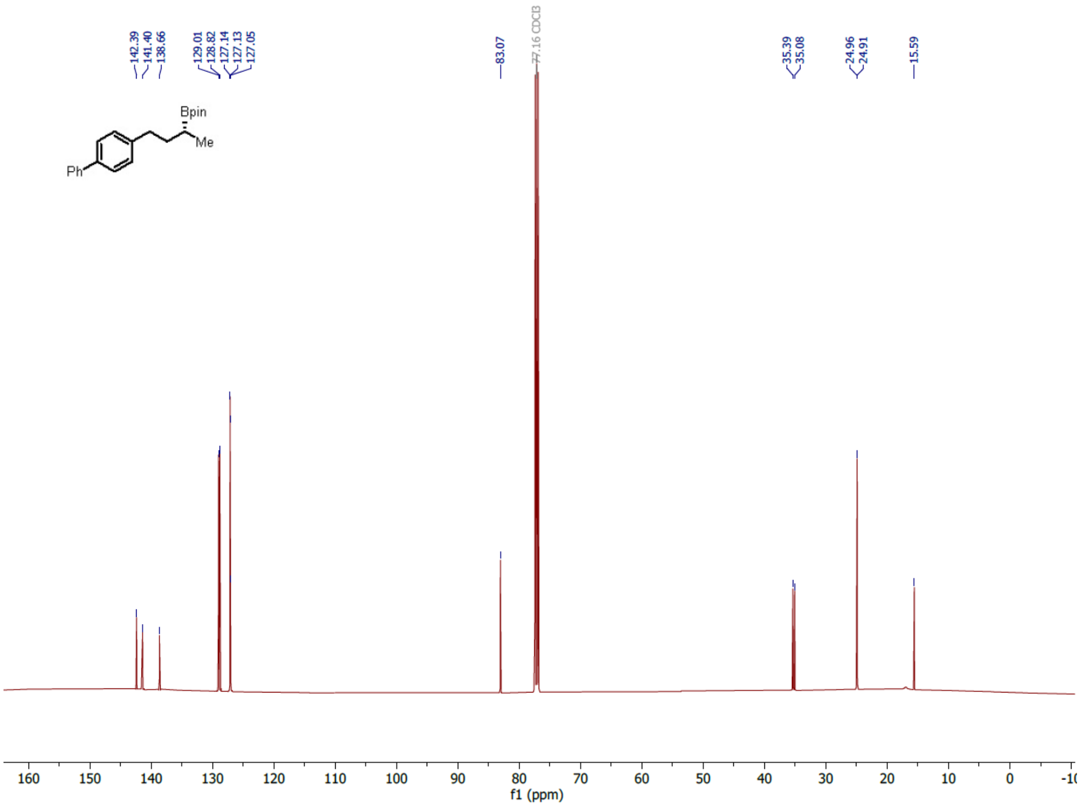

Compound 29

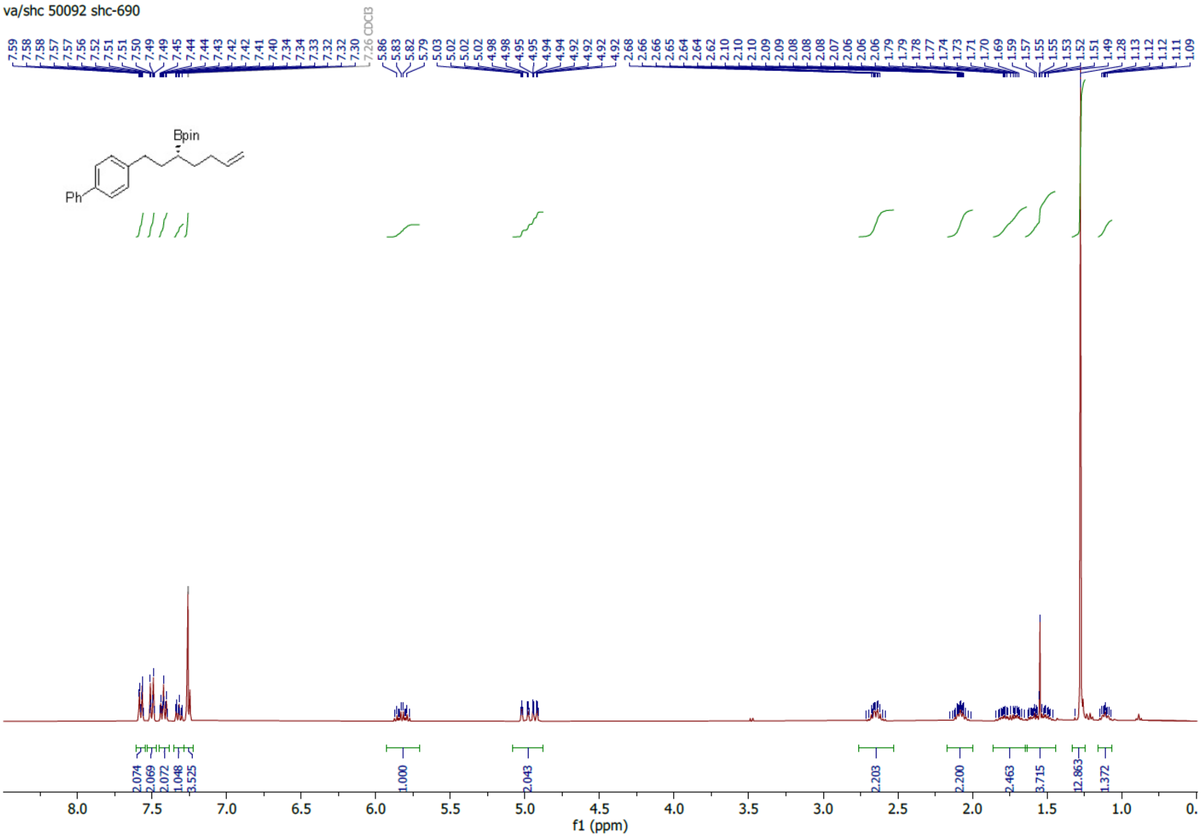
va/shc 50092 shc-690
Bpin
Ph
f1 (ppm)

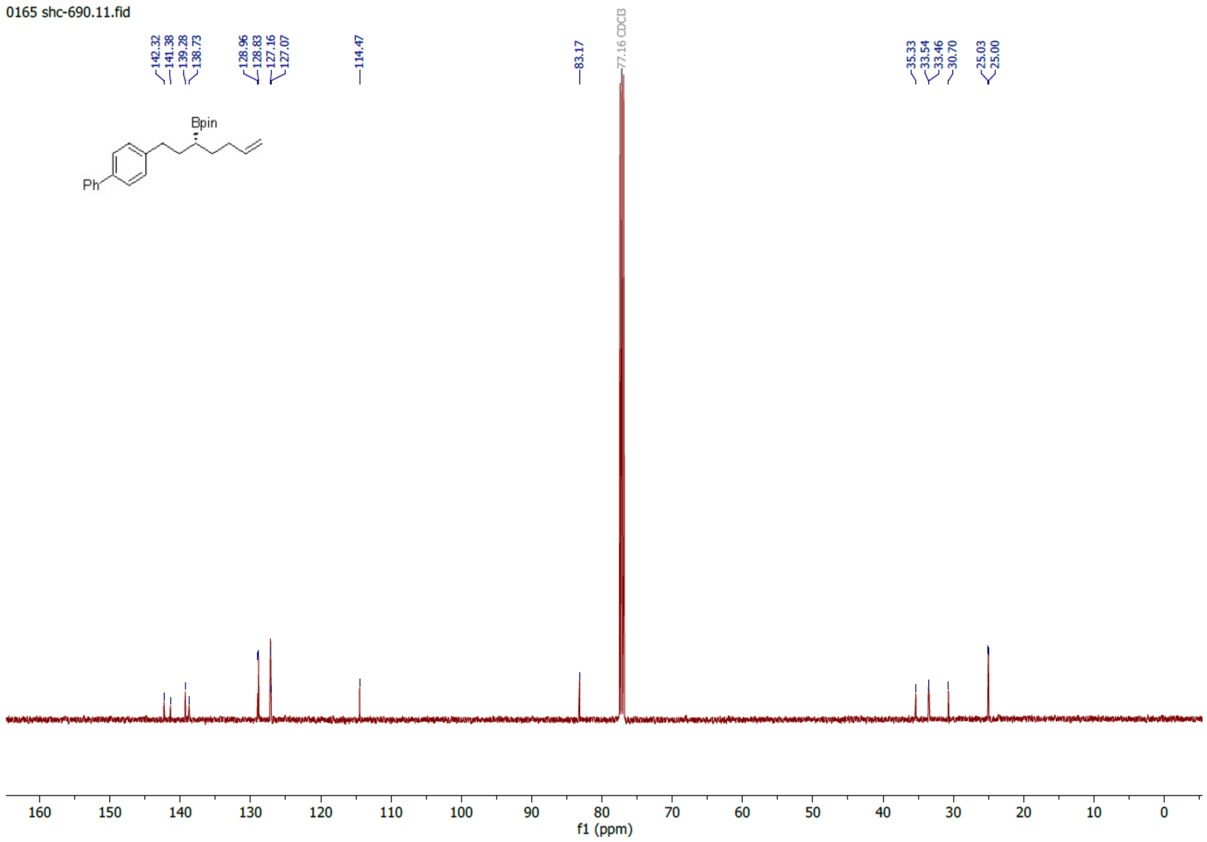
0165 shc-690.11.fid
Bpin
Ph
f1 (ppm)

Compound 32

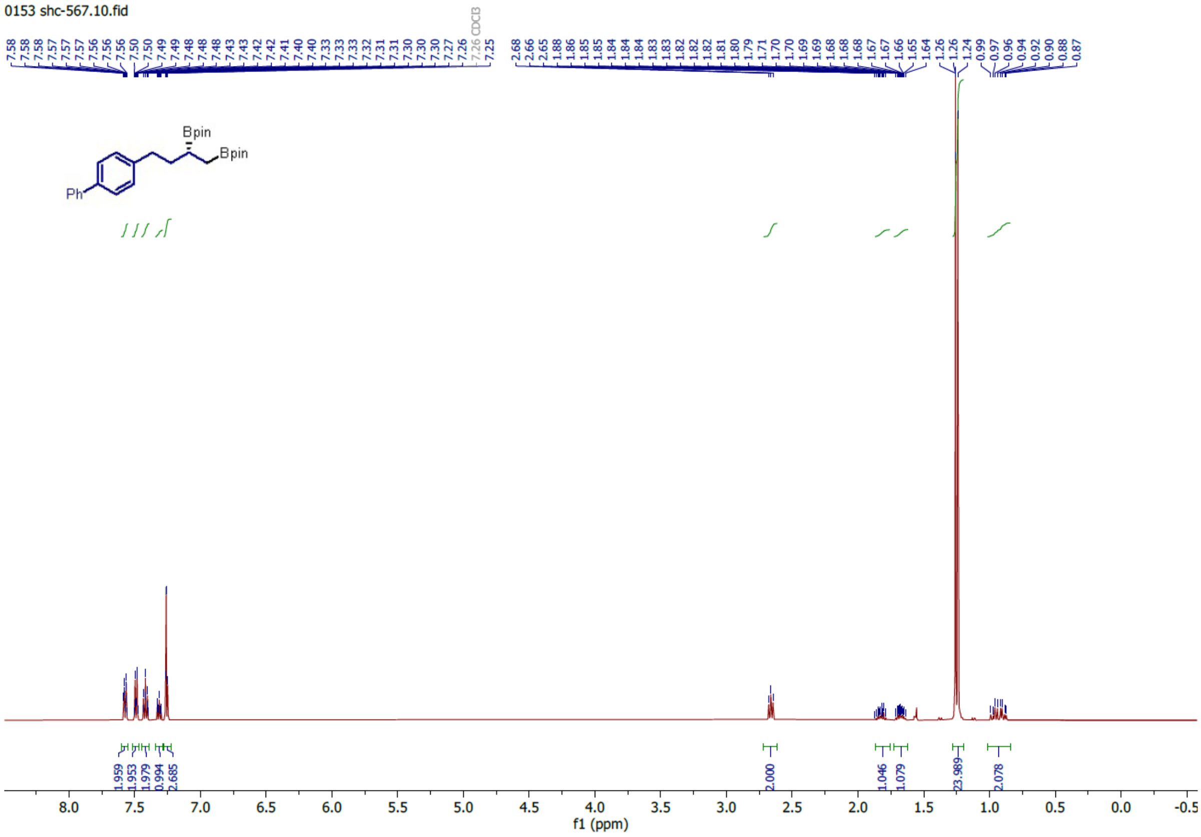
0153 shc-567.10.fid
Bpin
Bpin
Ph
7.26 CDCl3
1.959
1.953
1.979
0.994
2.685
2.000
1.046
1.079
23.989
2.078
f1 (ppm)

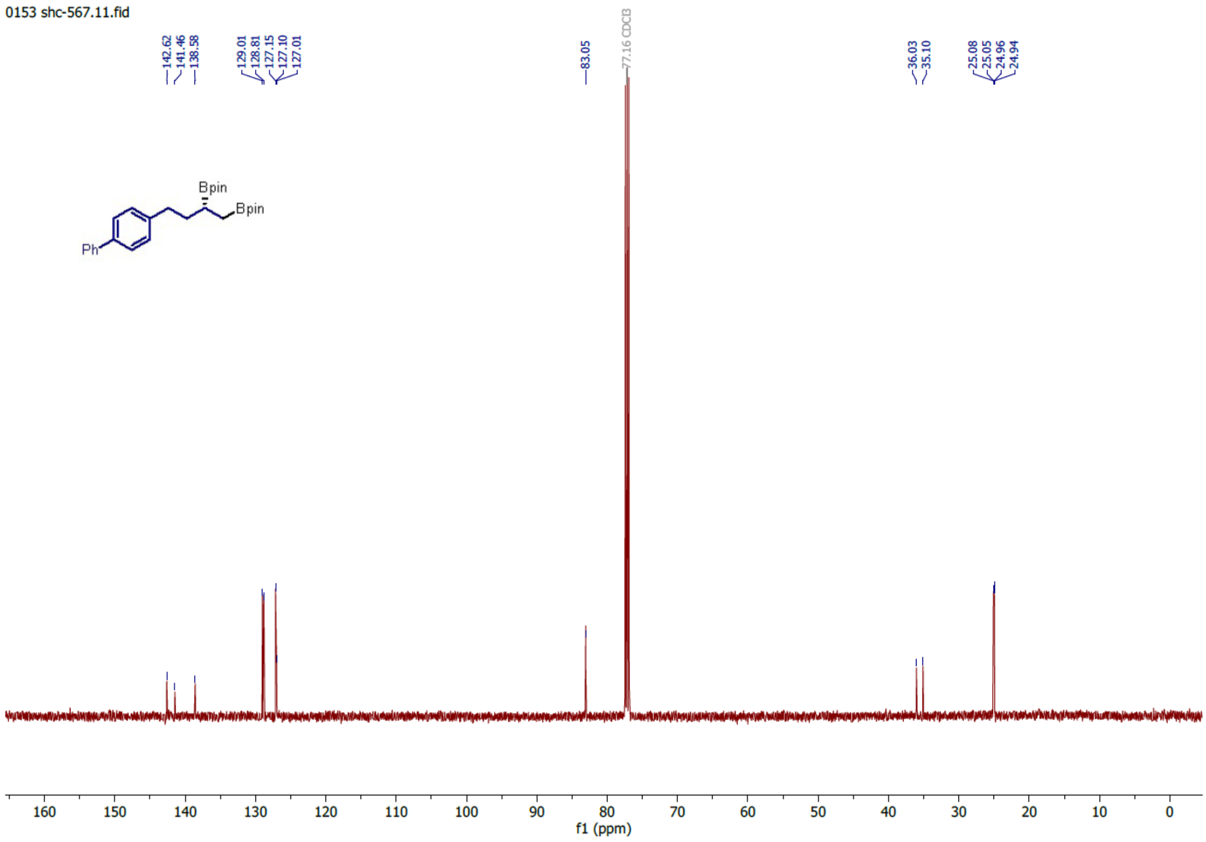
0153 shc-567.11.fid
142.62
141.46
138.58
129.01
128.81
127.15
127.10
127.01
83.05
77.16 CDCl3
36.03
35.10
25.08
25.05
24.96
24.94
Bpin
Bpin
Ph
f1 (ppm)

Compound S1

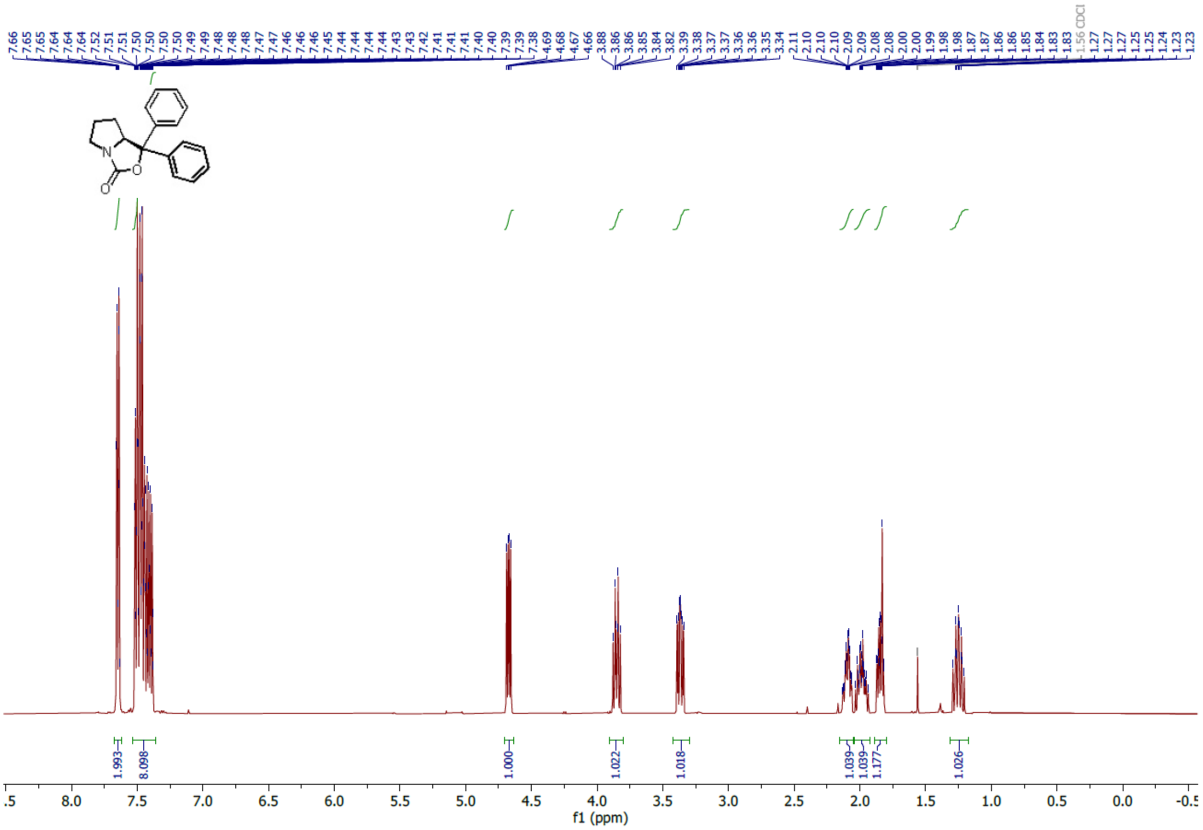

7.66 7.65 7.65 7.64 7.64 7.52 7.51 7.50 7.50 7.50 7.50 7.49 7.49 7.48 7.48 7.48 7.47 7.46 7.46 7.45 7.44 7.44 7.44 7.43 7.42 7.41 7.41 7.40 7.39 7.39 7.38 4.69 4.68 4.67 4.66 3.88 3.86 3.86 3.85 3.84 3.82 3.39 3.38 3.37 3.37 3.36 3.36 3.35 3.34 2.11 2.10 2.10 2.10 2.09 2.09 2.08 2.08 2.00 2.00 1.99 1.98 1.98 1.98 1.87 1.87 1.86 1.86 1.85 1.84 1.83 1.56 CDCl 1.27 1.27 1.27 1.25 1.25 1.24 1.23 1.23
N
O
O
1.993
8.098
1.000
1.022
1.018
1.039
1.039
1.177
1.026
f1 (ppm)

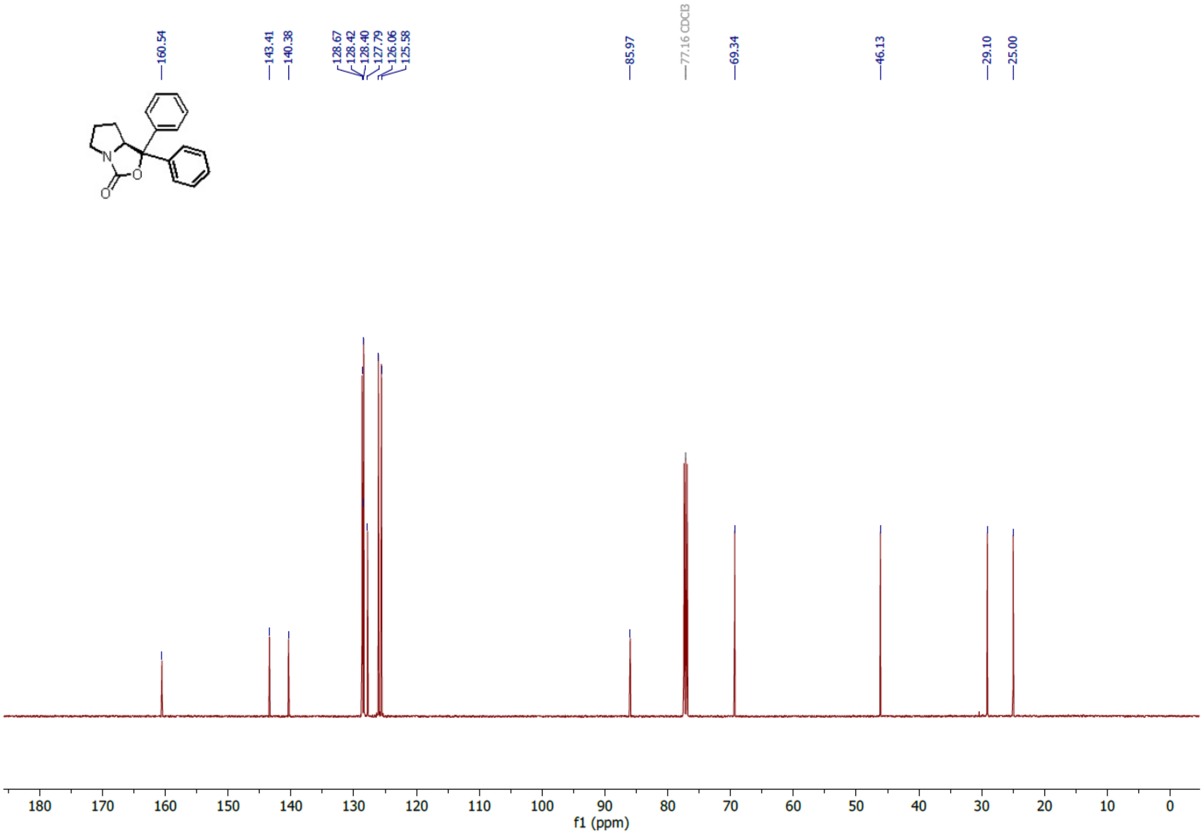

160.54
143.41
140.38
128.67
128.42
128.40
127.79
126.06
125.58
85.97
77.16 CDCl3
69.34
46.13
29.10
25.00
N
O
O
f1 (ppm)

Compound 4

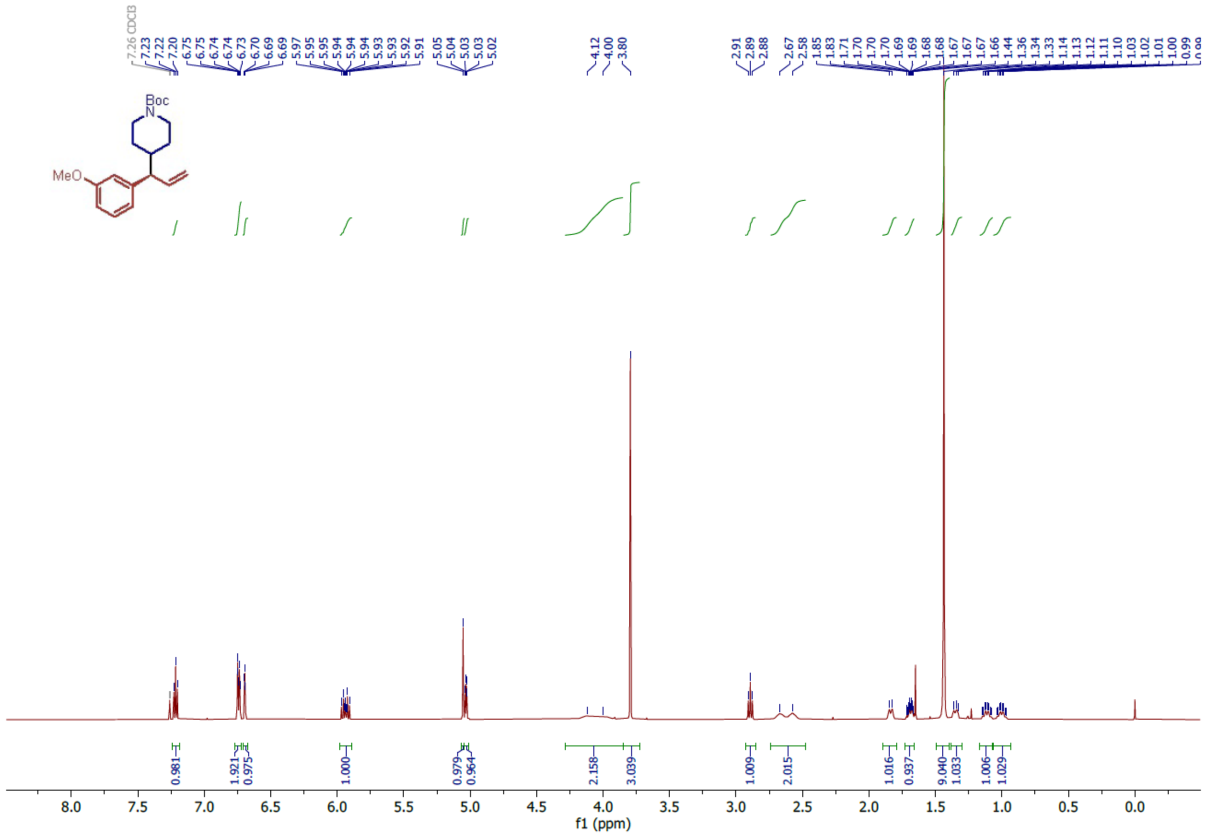

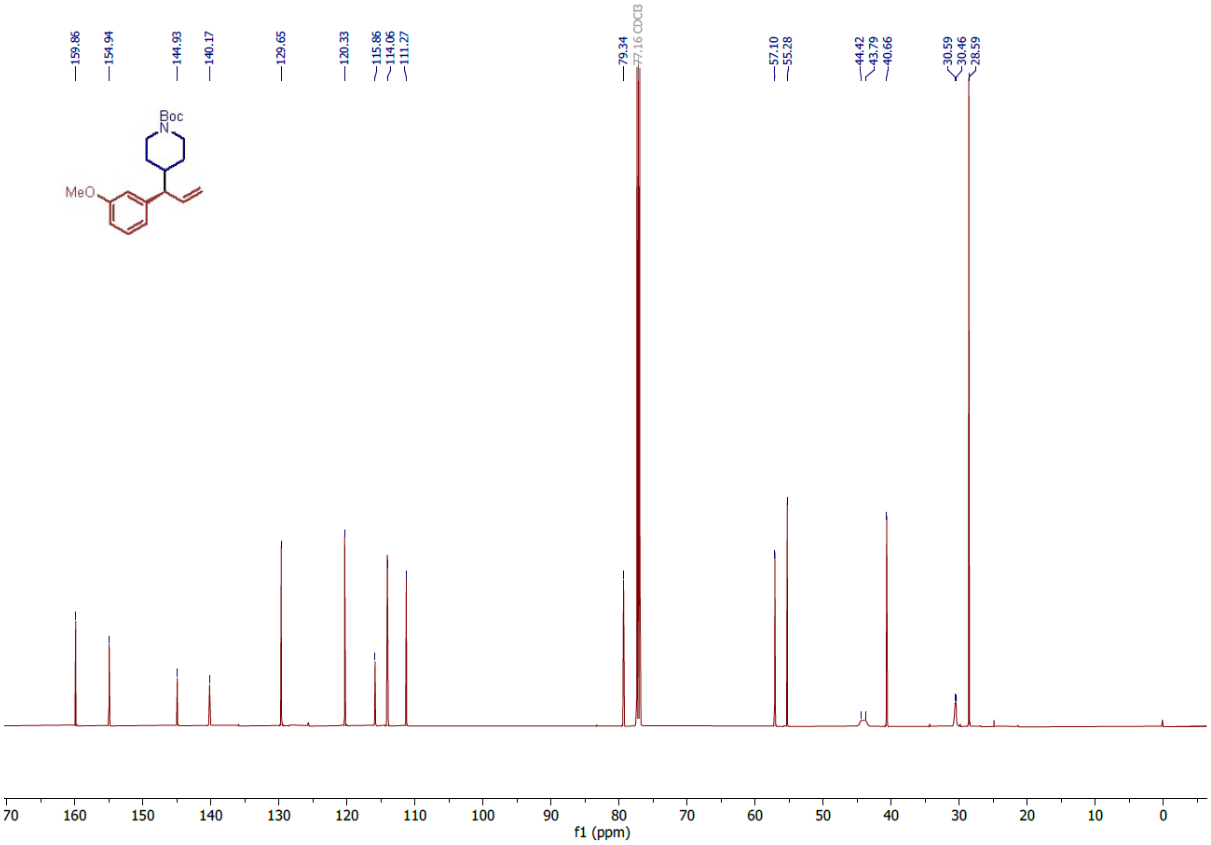

Compound 7

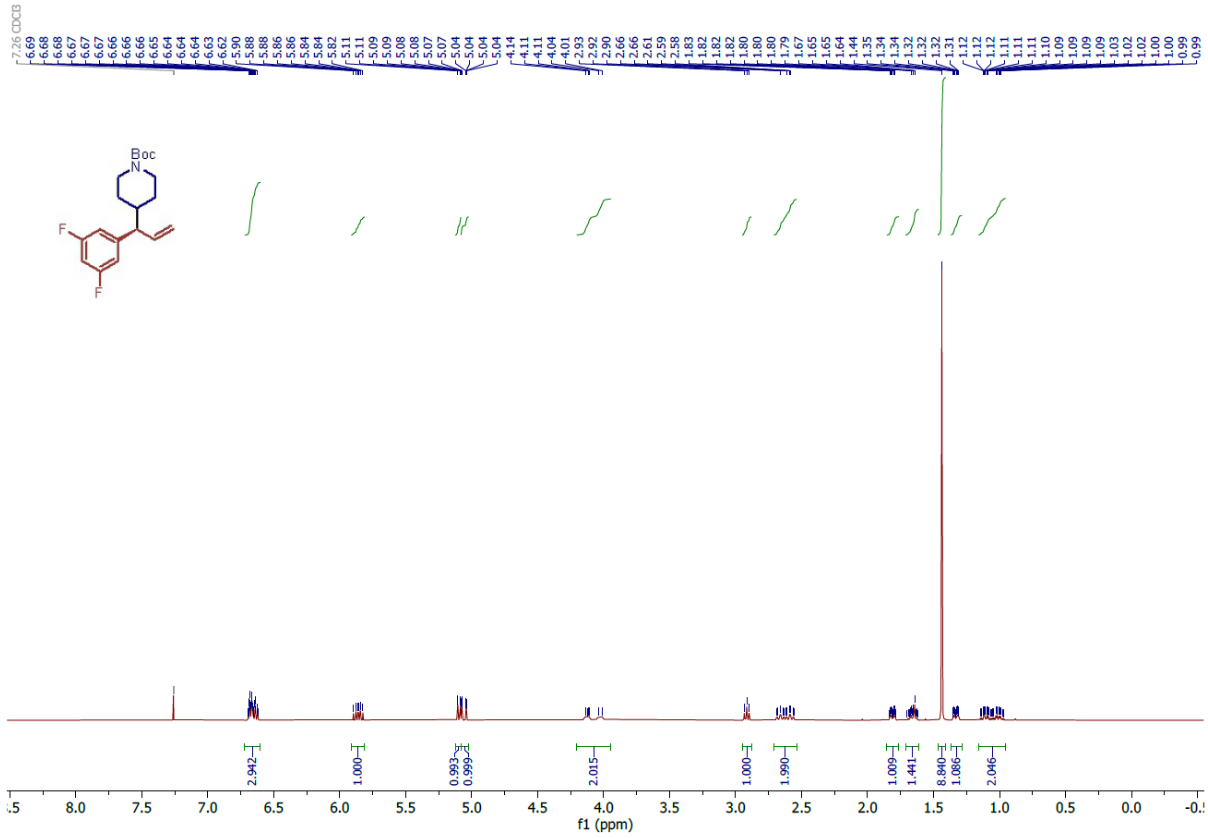

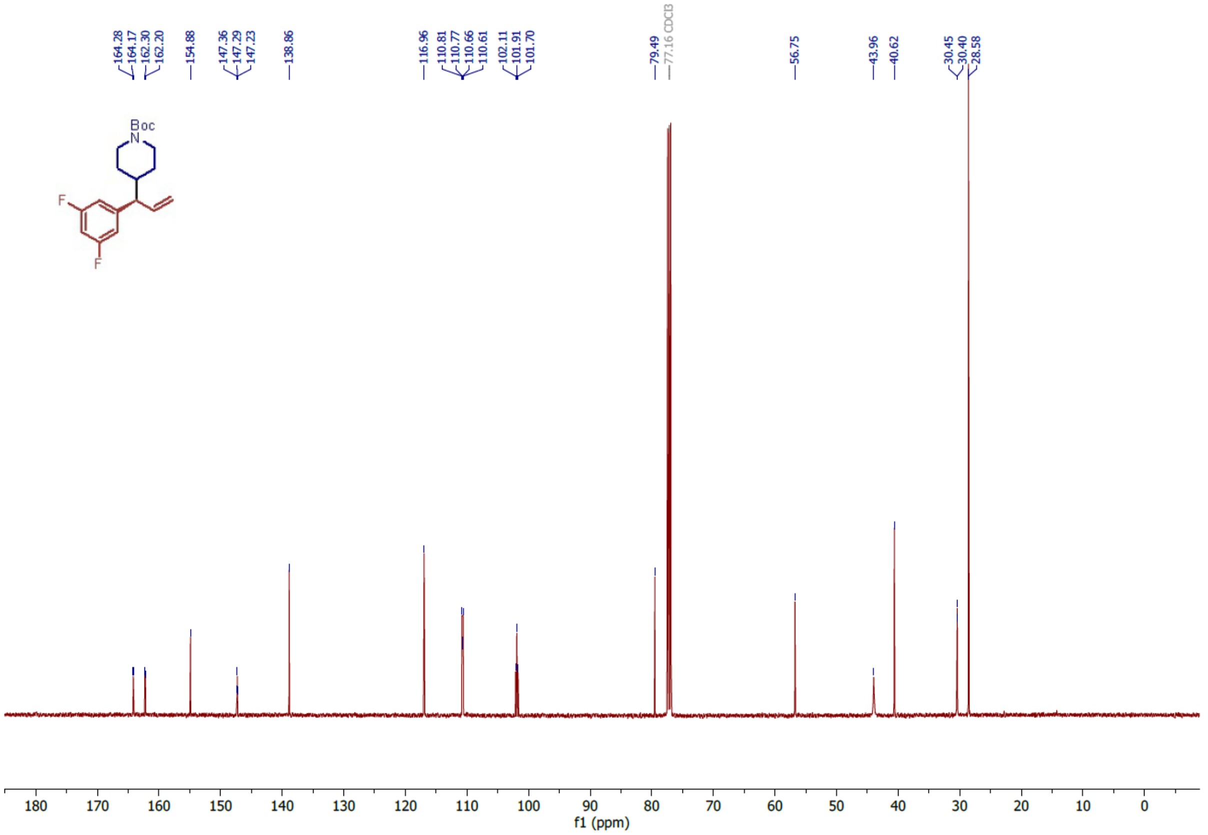

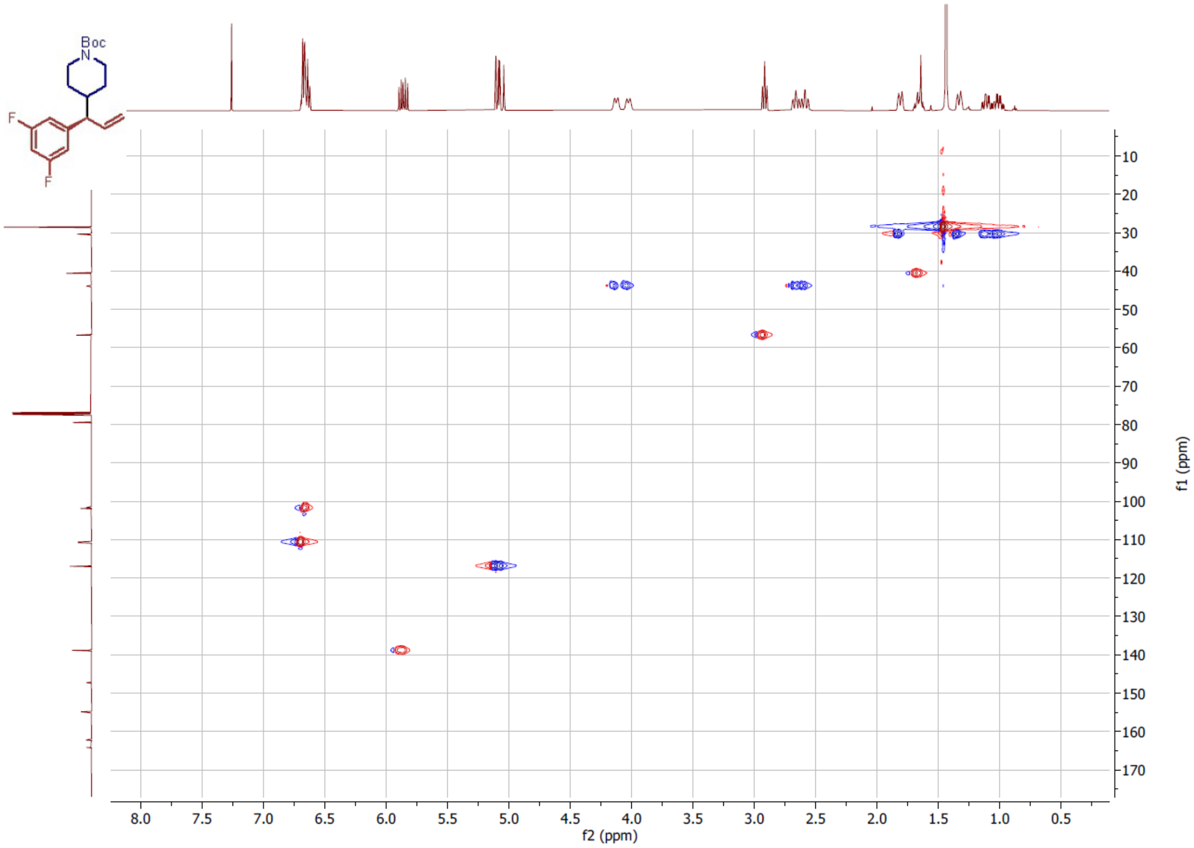

Boc
N
F
F
8.0 7.5 7.0 6.5 6.0 5.5 5.0 4.5 4.0 3.5 3.0 2.5 2.0 1.5 1.0 0.5
f2 (ppm)
10 20 30 40 50 60 70 80 90 100 110 120 130 140 150 160 170
f1 (ppm)

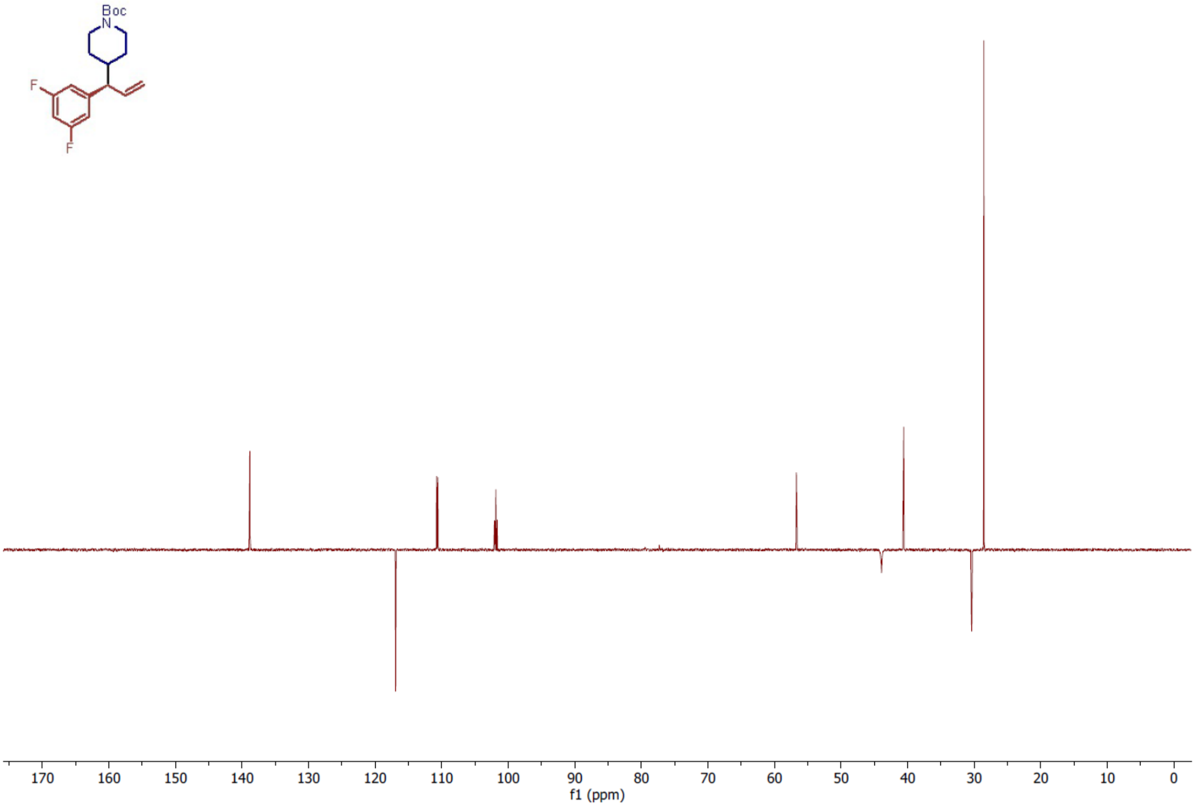

Boc
N
F
F
170 160 150 140 130 120 110 100 90 80 70 60 50 40 30 20 10 0
f1 (ppm)

Compound 8

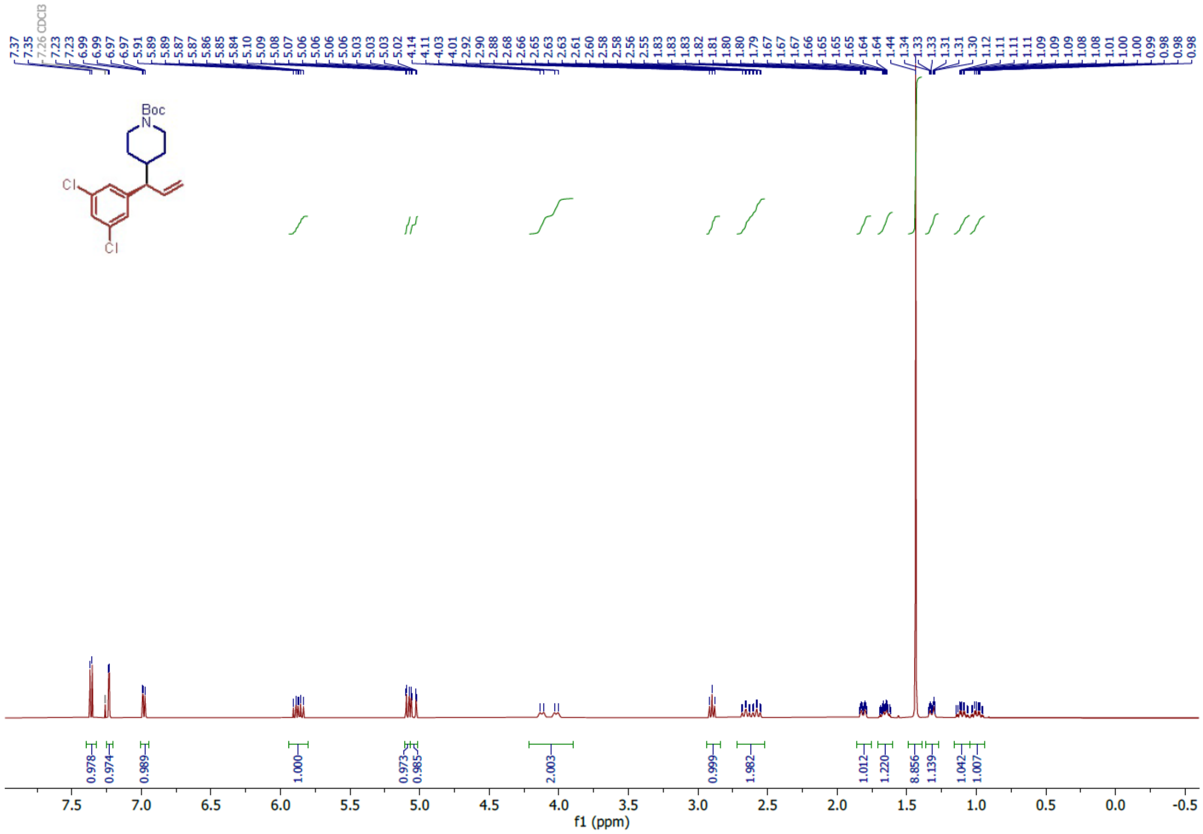
7.37
7.35
7.26 CDCl3
7.23
7.23
6.99
6.99
6.97
6.97
5.91
5.89
5.89
5.87
5.86
5.85
5.84
5.10
5.09
5.08
5.07
5.06
5.06
5.06
5.06
5.03
5.03
5.03
5.02
4.14
4.11
4.03
4.01
2.92
2.90
2.88
2.68
2.66
2.65
2.63
2.63
2.61
2.60
2.58
2.58
2.56
2.55
1.83
1.83
1.83
1.82
1.81
1.80
1.80
1.79
1.67
1.67
1.67
1.66
1.65
1.65
1.65
1.64
1.64
1.44
1.34
1.33
1.33
1.31
1.31
1.30
1.12
1.11
1.11
1.11
1.09
1.09
1.09
1.08
1.08
1.01
1.00
1.00
0.99
0.98
0.98
0.98
Boc
N
Cl
Cl
0.978
0.974
0.989
1.000
0.973
0.985
2.003
0.999
1.982
1.012
1.220
8.856
1.139
1.042
1.007
7.5
7.0
6.5
6.0
5.5
5.0
4.5
4.0
3.5
3.0
2.5
2.0
1.5
1.0
0.5
0.0
-0.5
f1 (ppm)

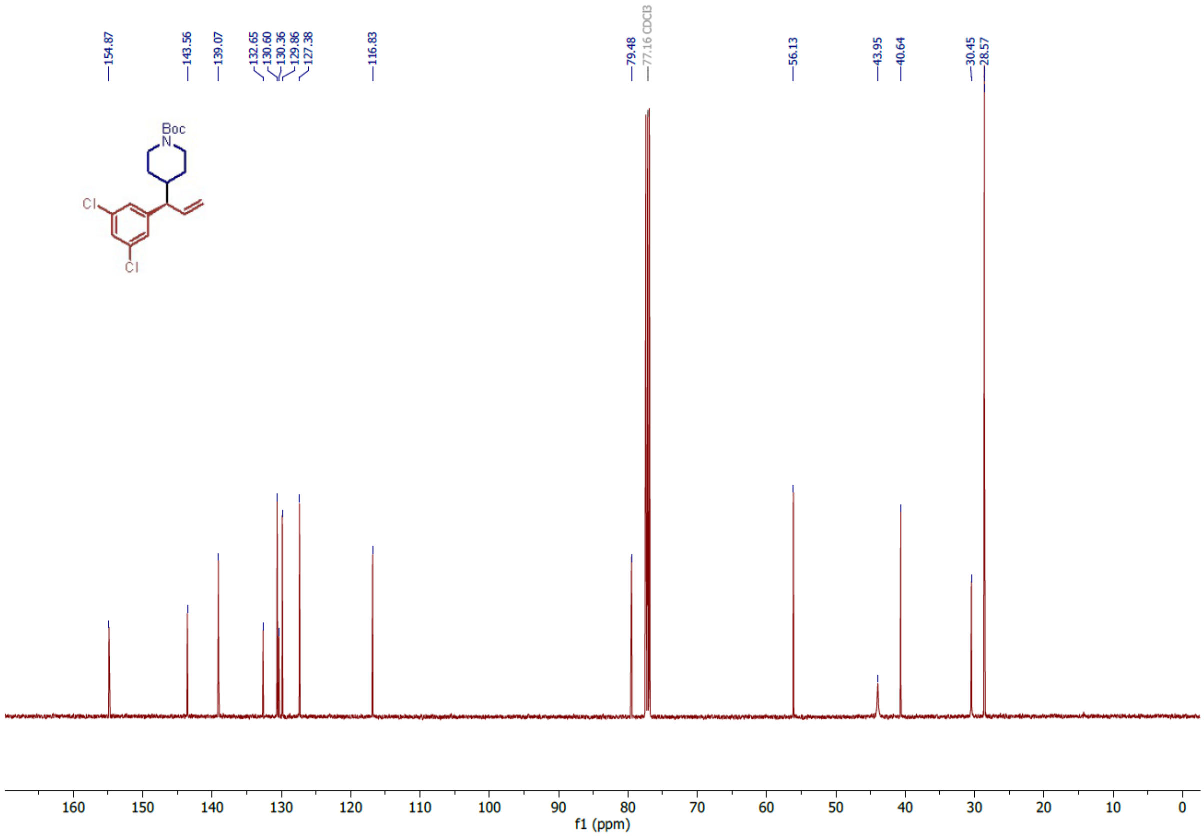
154.87
143.56
139.07
132.65
130.60
130.36
129.86
127.38
116.83
79.48
77.16 CDCl3
56.13
43.95
40.64
30.45
28.57
Boc
N
Cl
Cl
160
150
140
130
120
110
100
90
80
70
60
50
40
30
20
10
0
f1 (ppm)

Compound 9

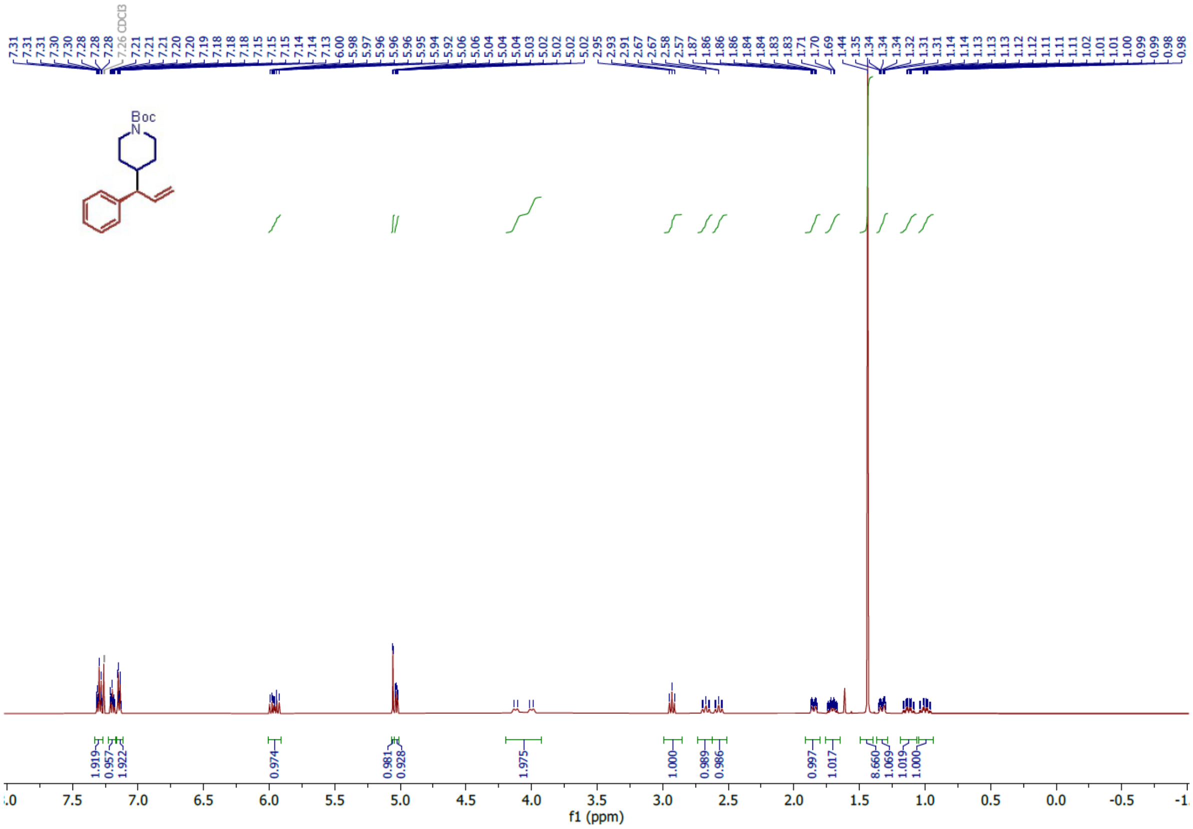

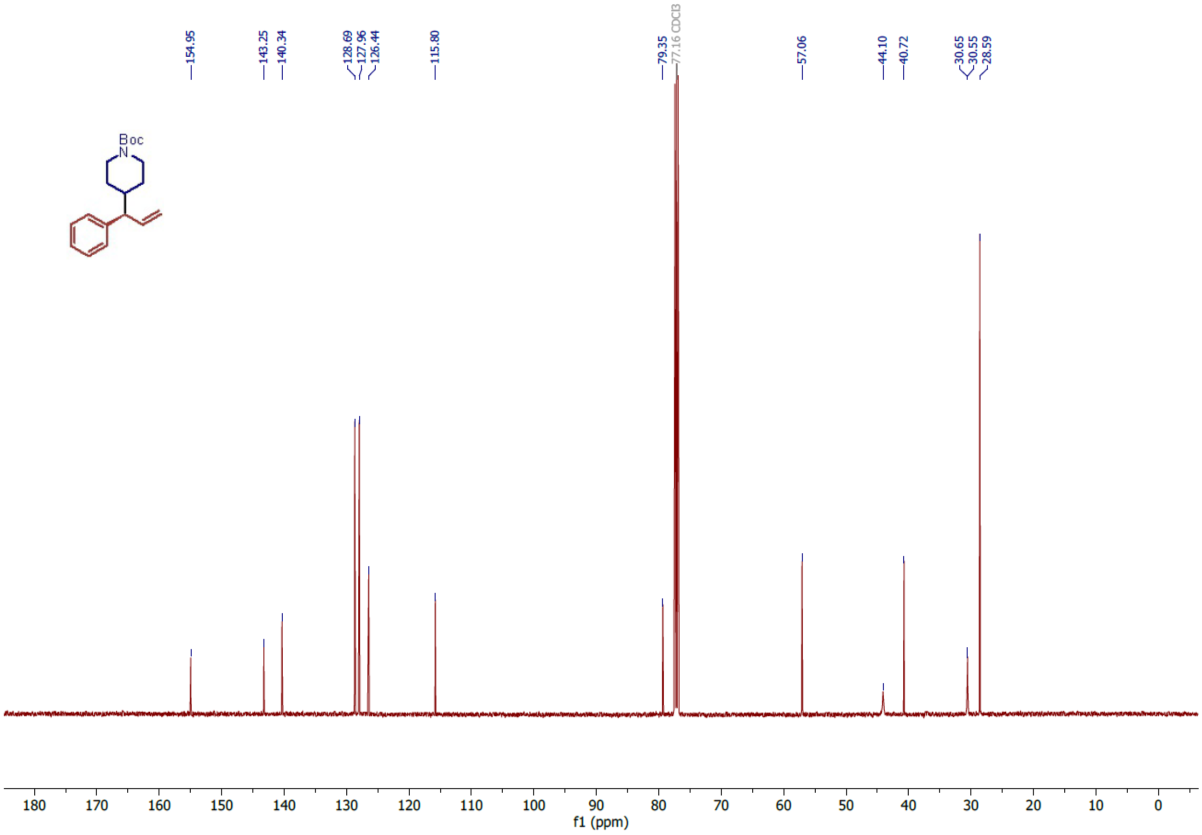

Compound 10

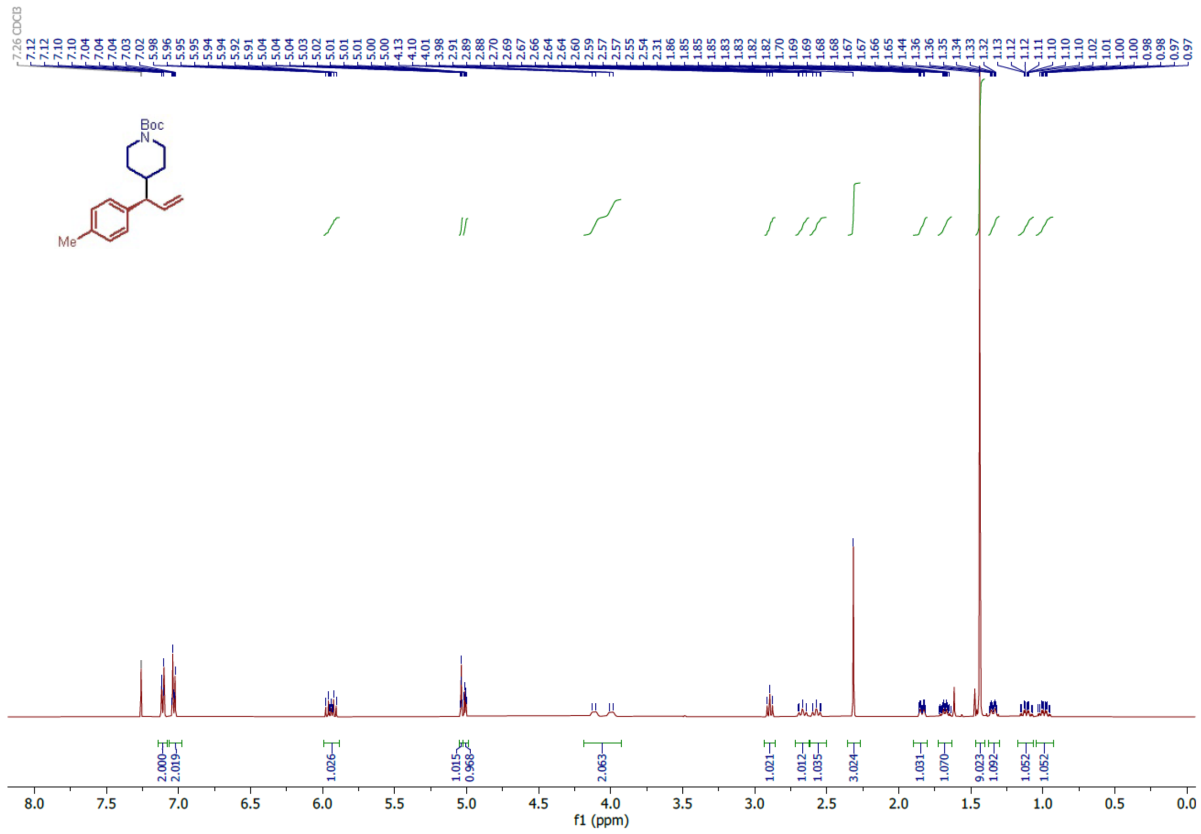
7.26 CDCl3
7.12 7.12 7.10 7.10 7.04 7.04 7.04 7.03 7.02 5.98 5.96 5.95 5.95 5.94 5.94 5.92 5.91 5.04 5.04 5.04 5.03 5.02 5.01 5.01 5.01 5.00 5.00 4.13 4.10 4.01 3.98 2.91 2.89 2.88 2.70 2.69 2.67 2.66 2.64 2.64 2.60 2.59 2.57 2.57 2.55 2.54 2.31 1.86 1.85 1.85 1.85 1.83 1.83 1.82 1.82 1.70 1.69 1.69 1.68 1.68 1.67 1.67 1.66 1.65 1.44 1.36 1.36 1.35 1.34 1.33 1.32 1.13 1.12 1.12 1.11 1.10 1.10 1.10 1.02 1.01 1.00 1.00 0.98 0.98 0.97 0.97
Boc
N
Me
2.000
2.019
1.026
1.015
0.968
2.063
1.021
1.012
1.035
3.024
1.031
1.070
9.023
1.092
1.052
1.052
8.0 7.5 7.0 6.5 6.0 5.5 5.0 4.5 4.0 3.5 3.0 2.5 2.0 1.5 1.0 0.5 0.0
f1 (ppm)

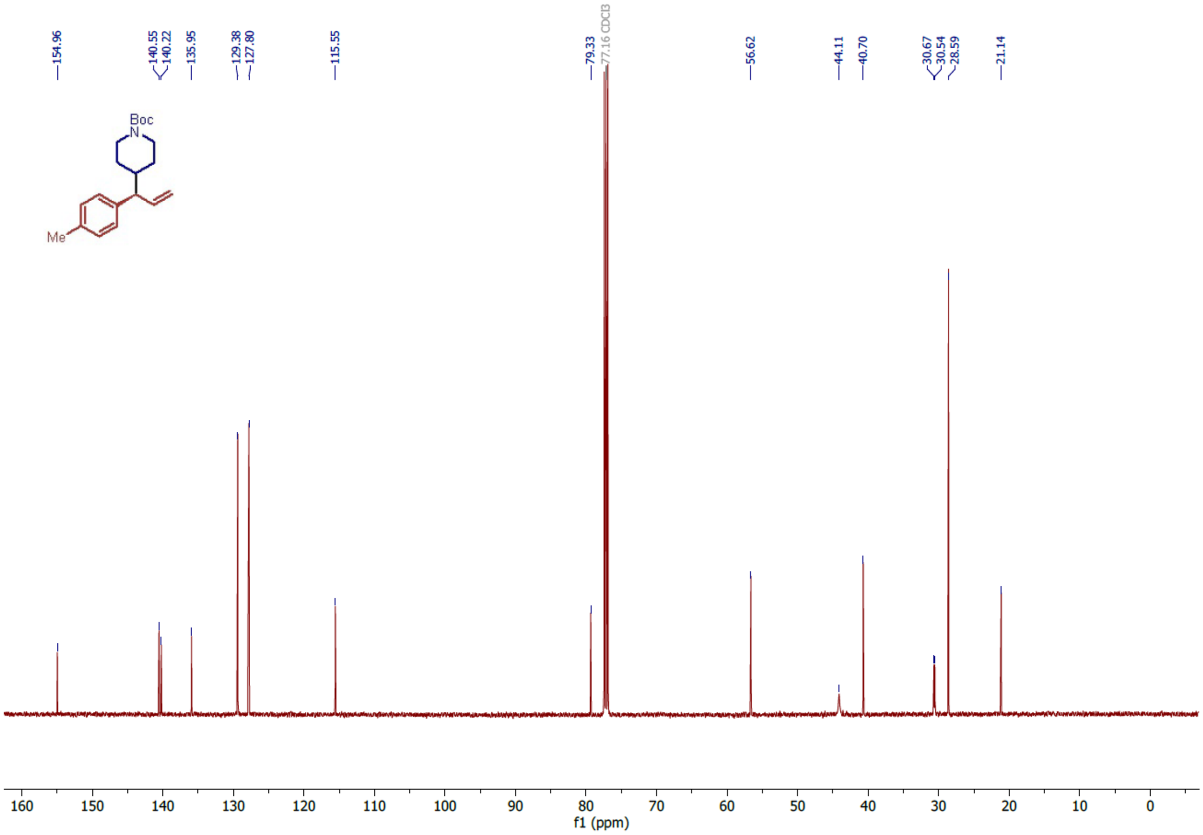
154.96
140.55
140.22
135.95
129.38
127.80
115.55
79.33
77.16 CDCl3
56.62
44.11
40.70
30.67
30.54
28.59
21.14
Boc
N
Me
160 150 140 130 120 110 100 90 80 70 60 50 40 30 20 10 0
f1 (ppm)

Compound 11

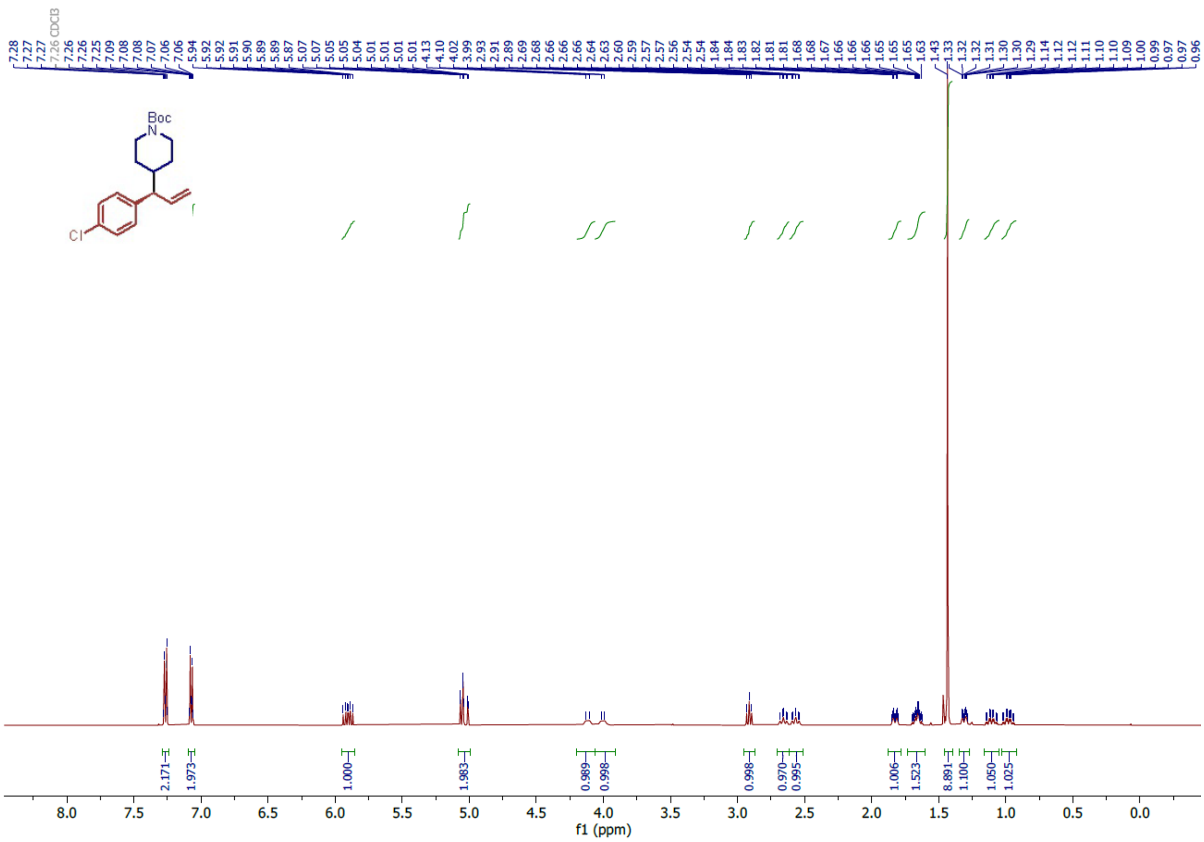

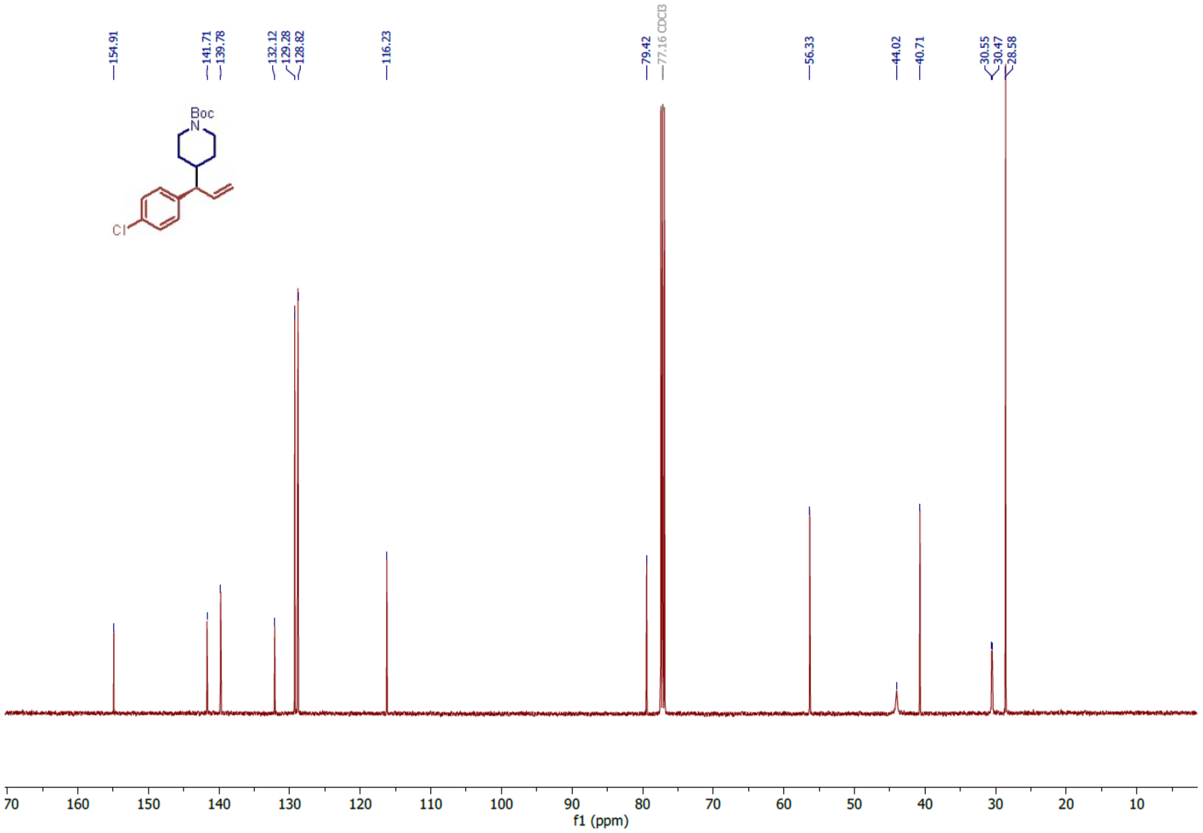

Compound 12

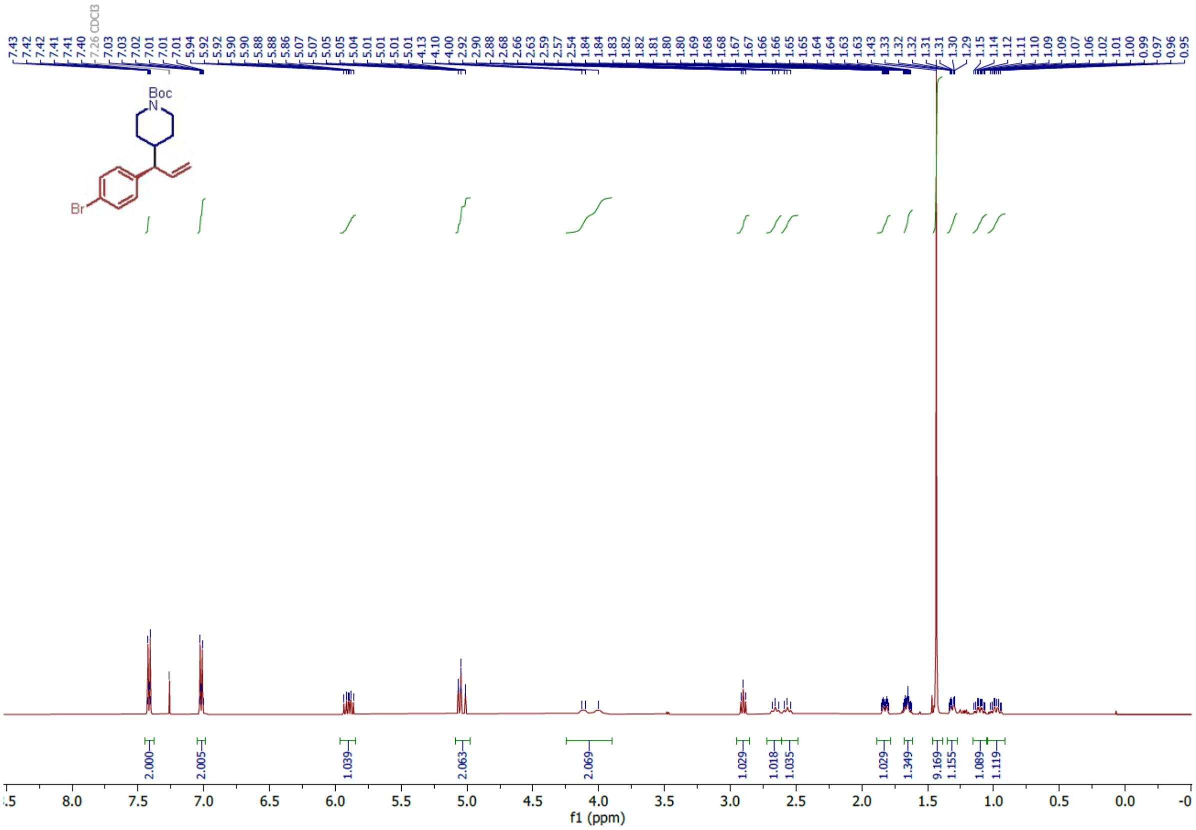

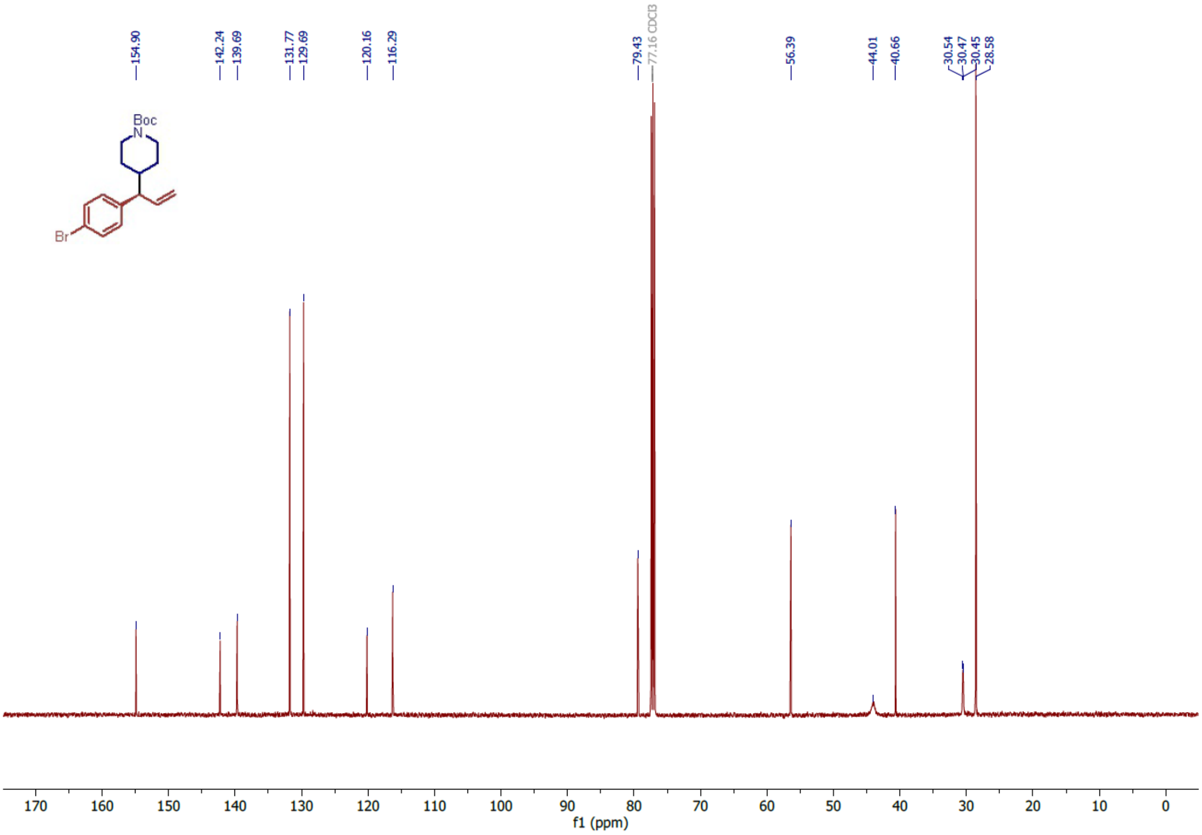

Compound 13

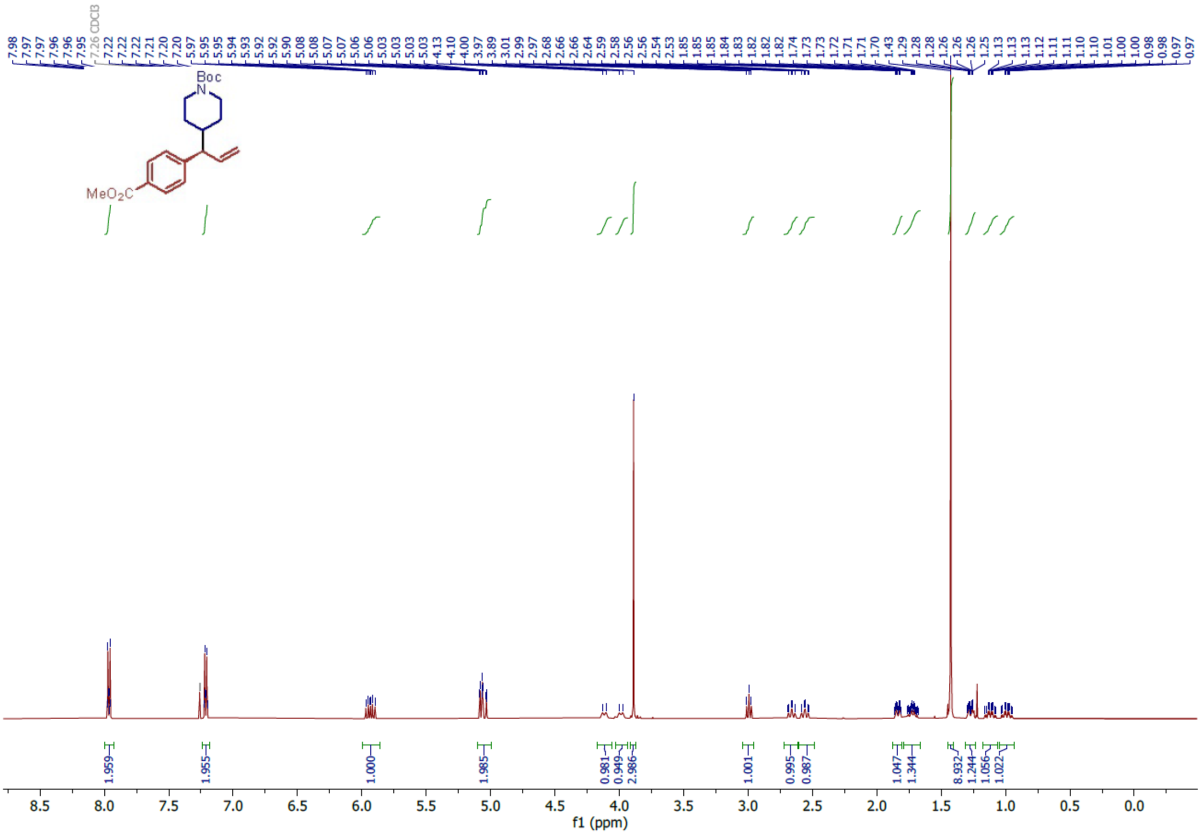

Boc
N
MeO2C
1.959
1.955
1.000
1.985
0.981
0.949
2.986
1.001
0.995
0.987
1.047
1.344
8.932
1.244
1.056
1.022
f1 (ppm)

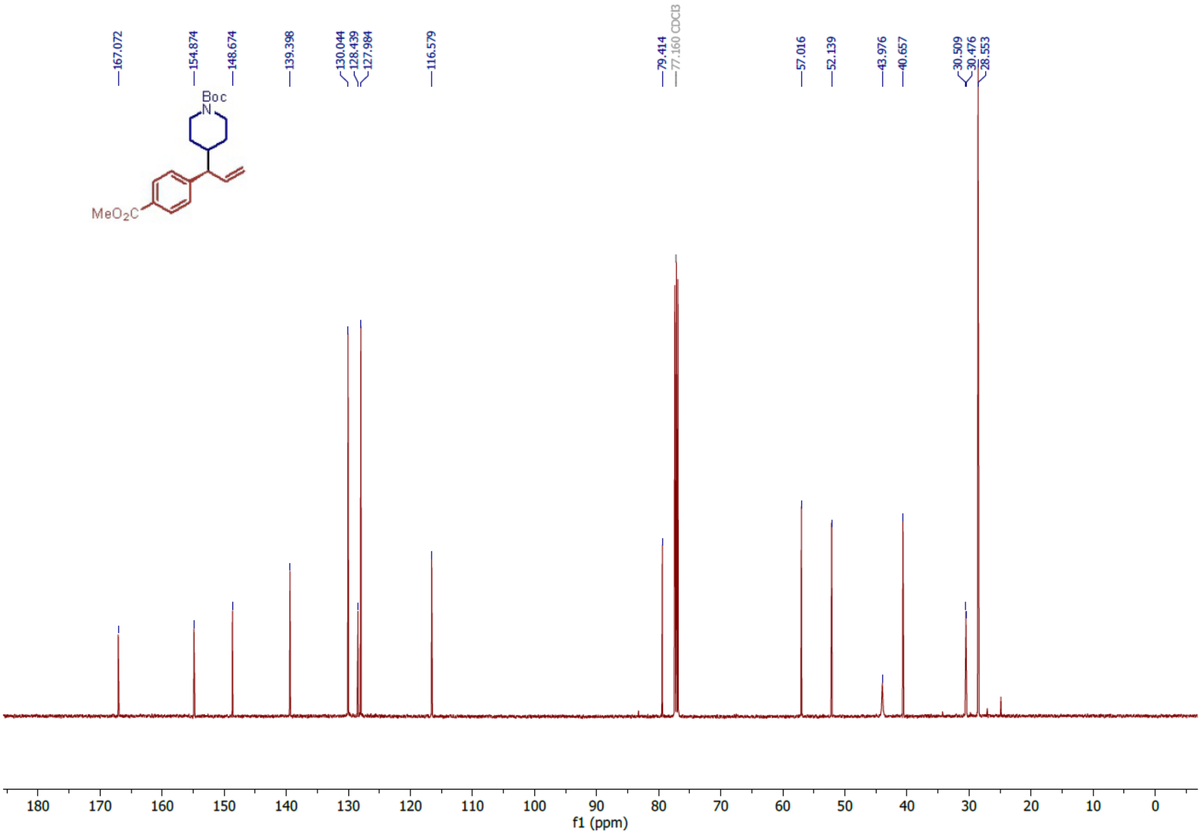

167.072
154.874
148.674
139.398
130.044
128.439
127.984
116.579
79.414
77.160 CDCl3
57.016
52.139
43.976
40.657
30.509
30.476
28.553
Boc
N
MeO2C
f1 (ppm)

Compound 14

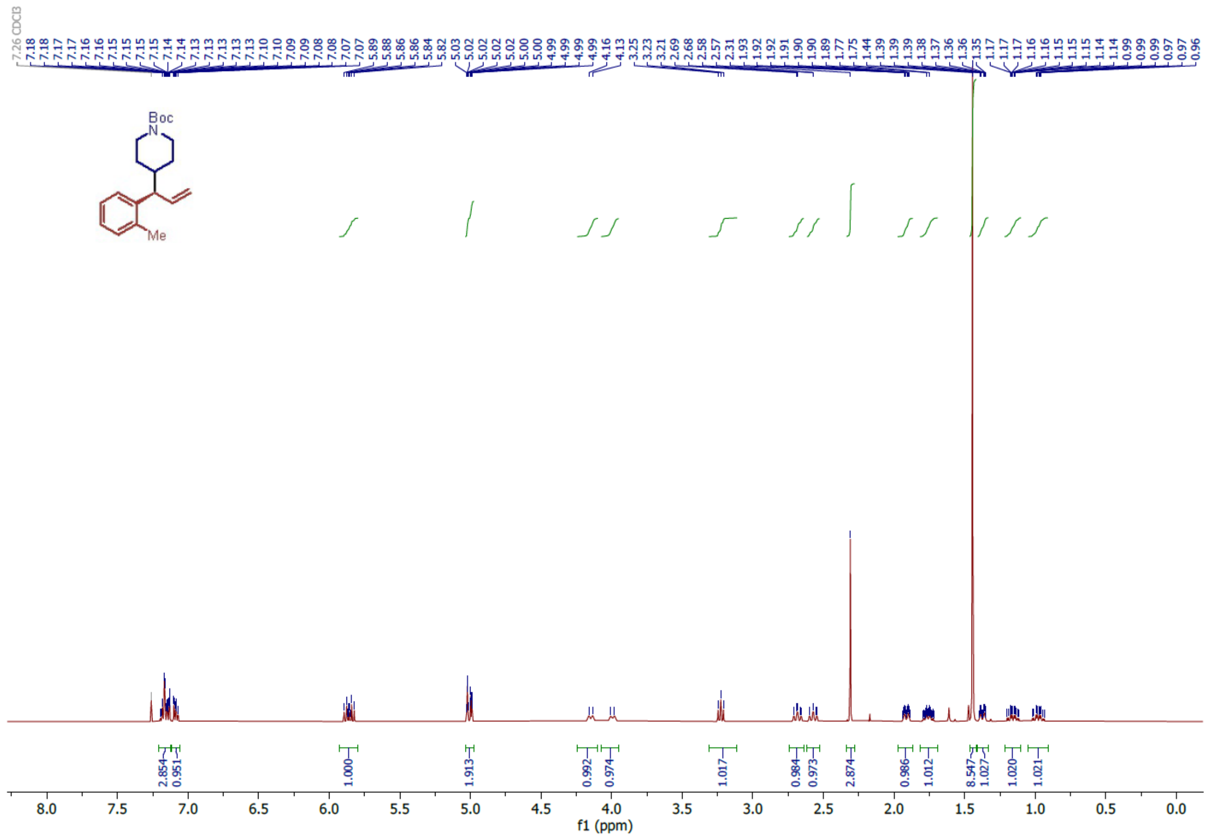
Boc
N
Me
2.854
0.951
1.000
1.913
0.992
0.974
1.017
0.984
0.973
2.874
0.986
1.012
8.547
1.027
1.020
1.021
f1 (ppm)

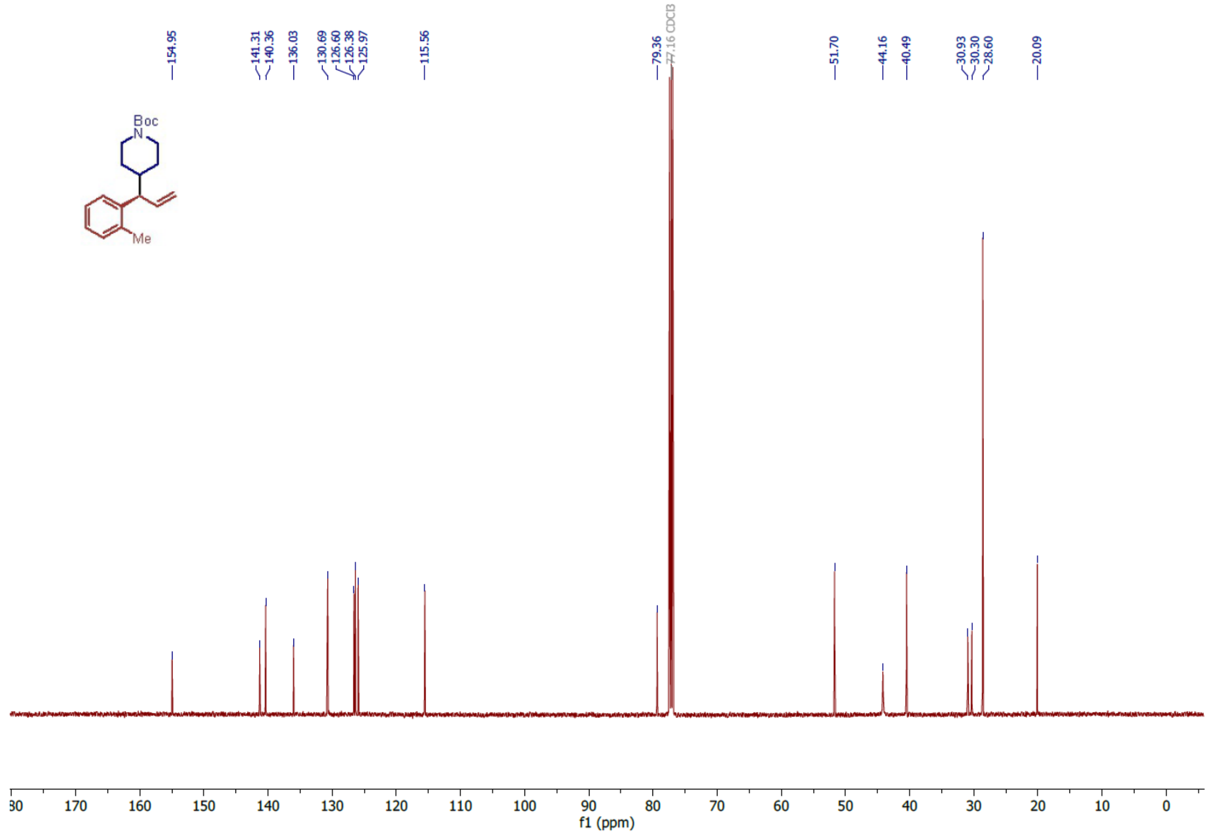
154.95
141.31
140.36
136.03
130.69
126.60
126.38
125.97
115.56
79.36
77.16 CDCl3
51.70
44.16
40.49
30.93
30.30
28.60
20.09
Boc
N
Me
f1 (ppm)

Compound 15

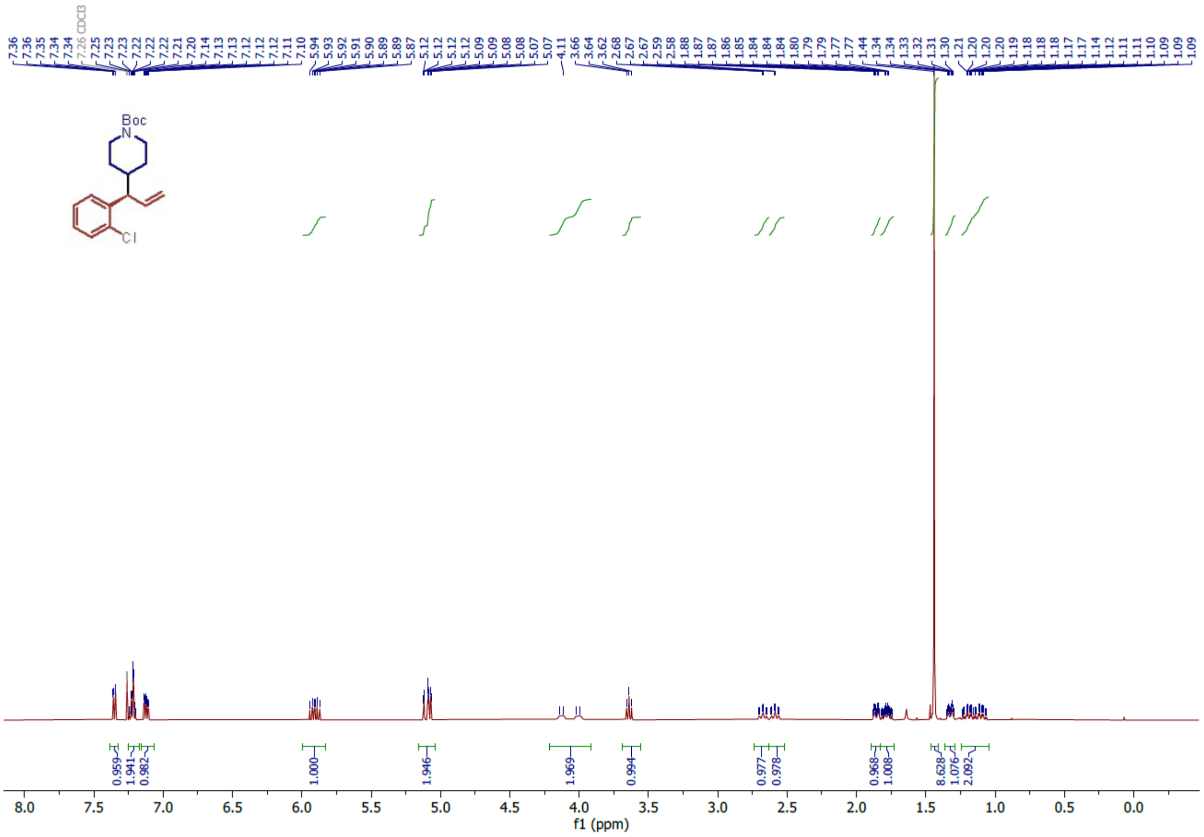

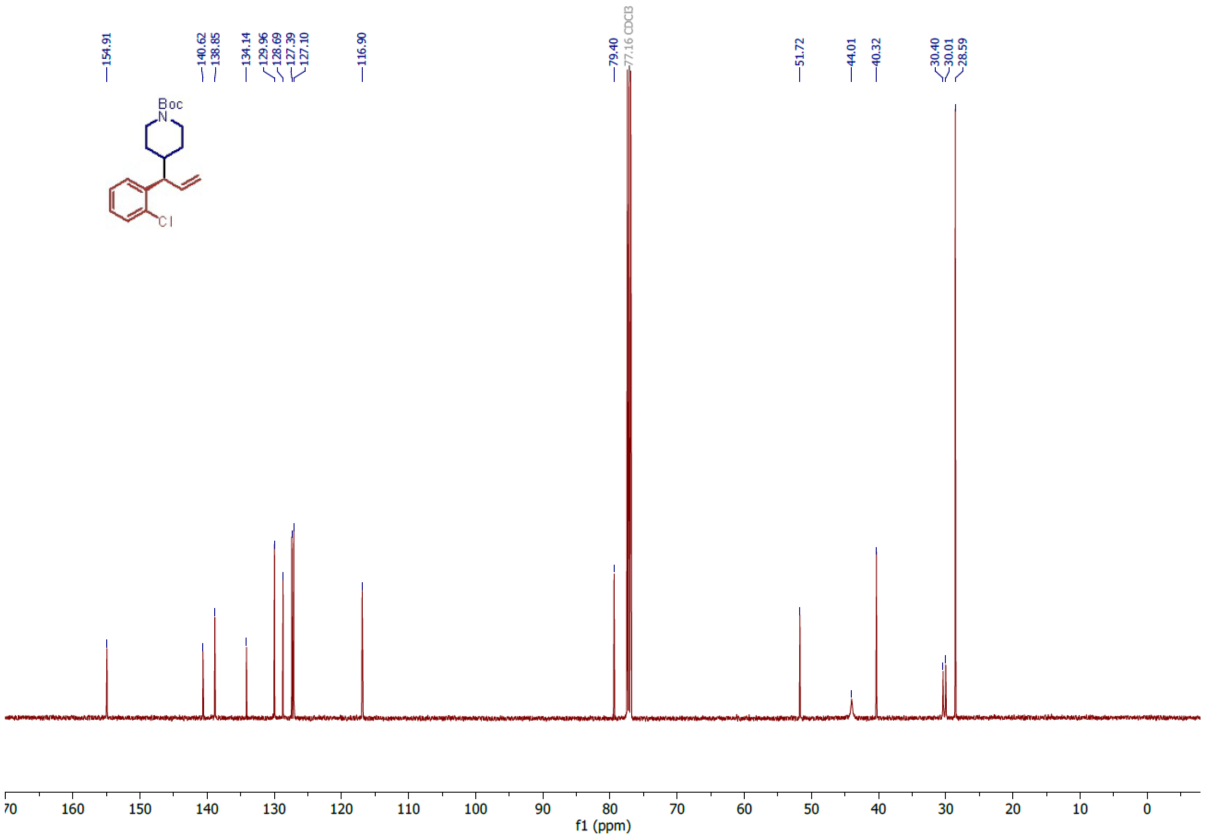

Compound 16

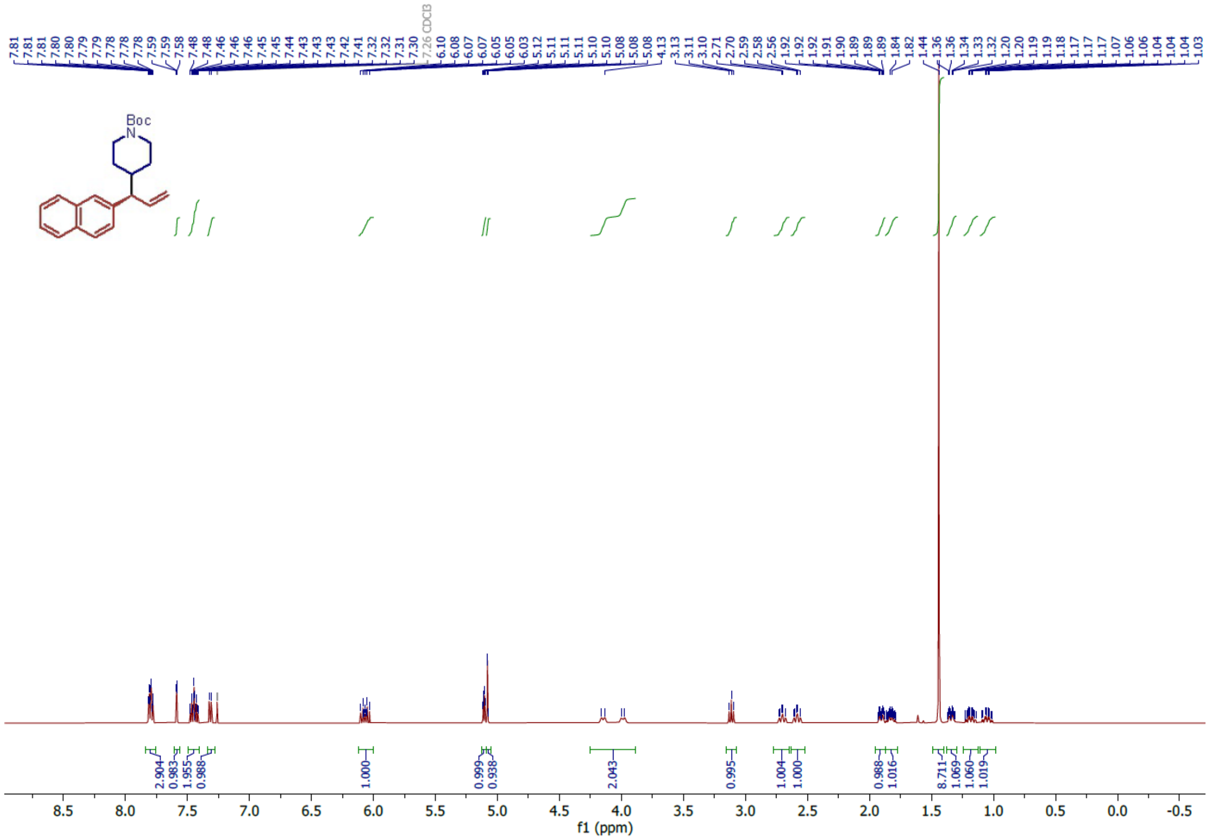

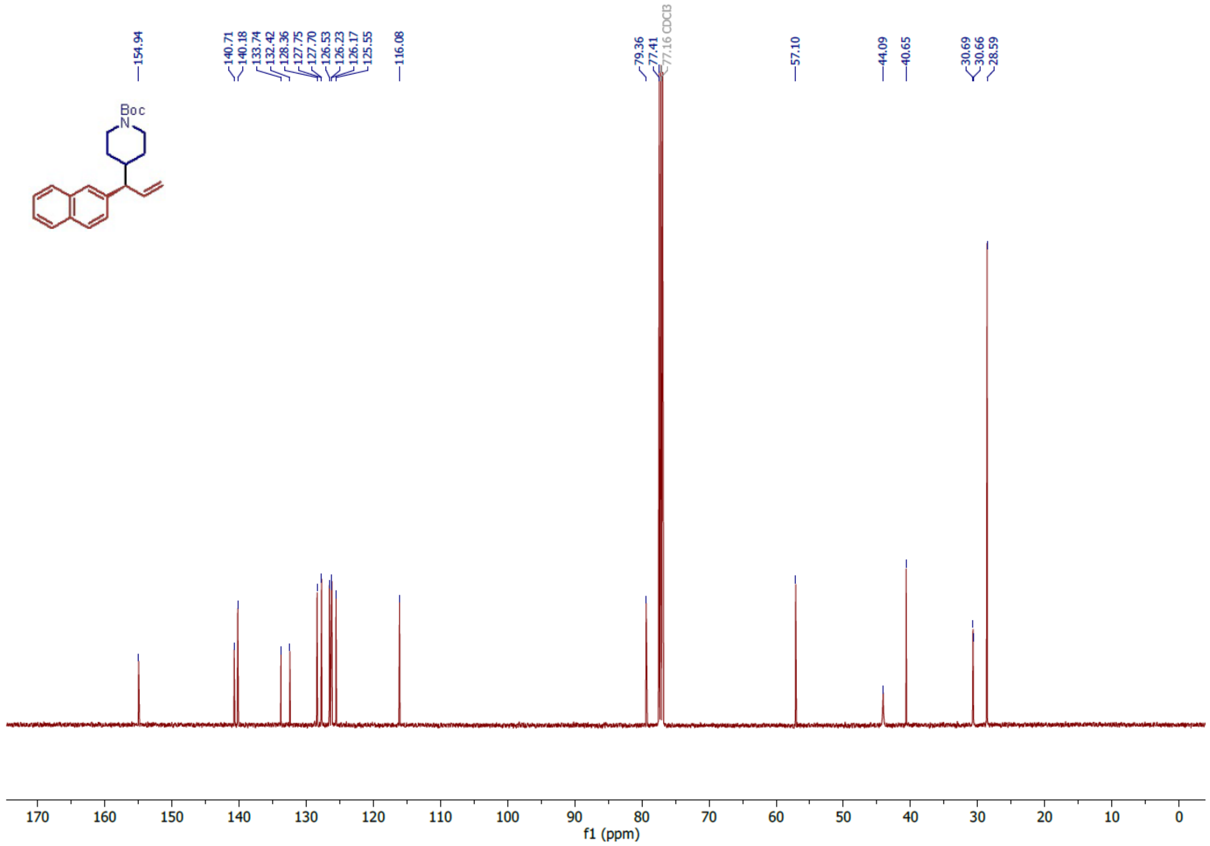

Compound 17

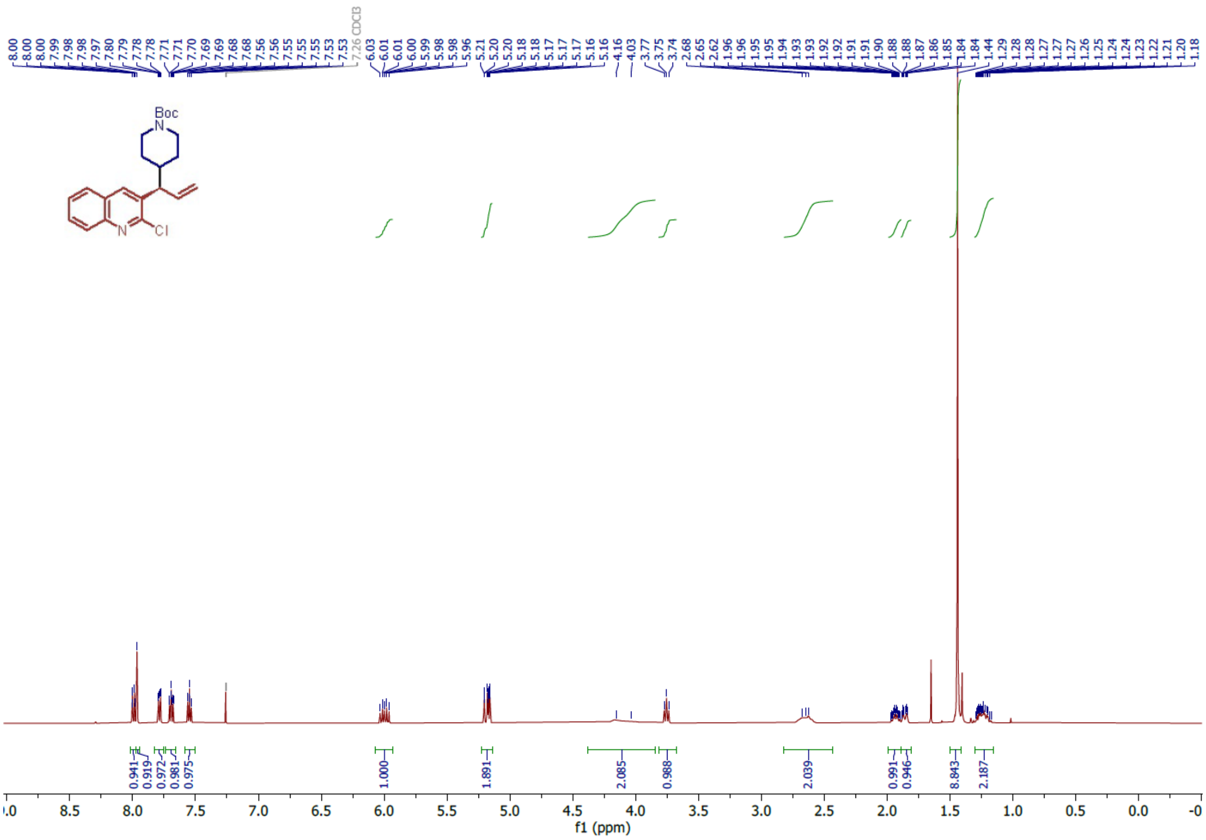

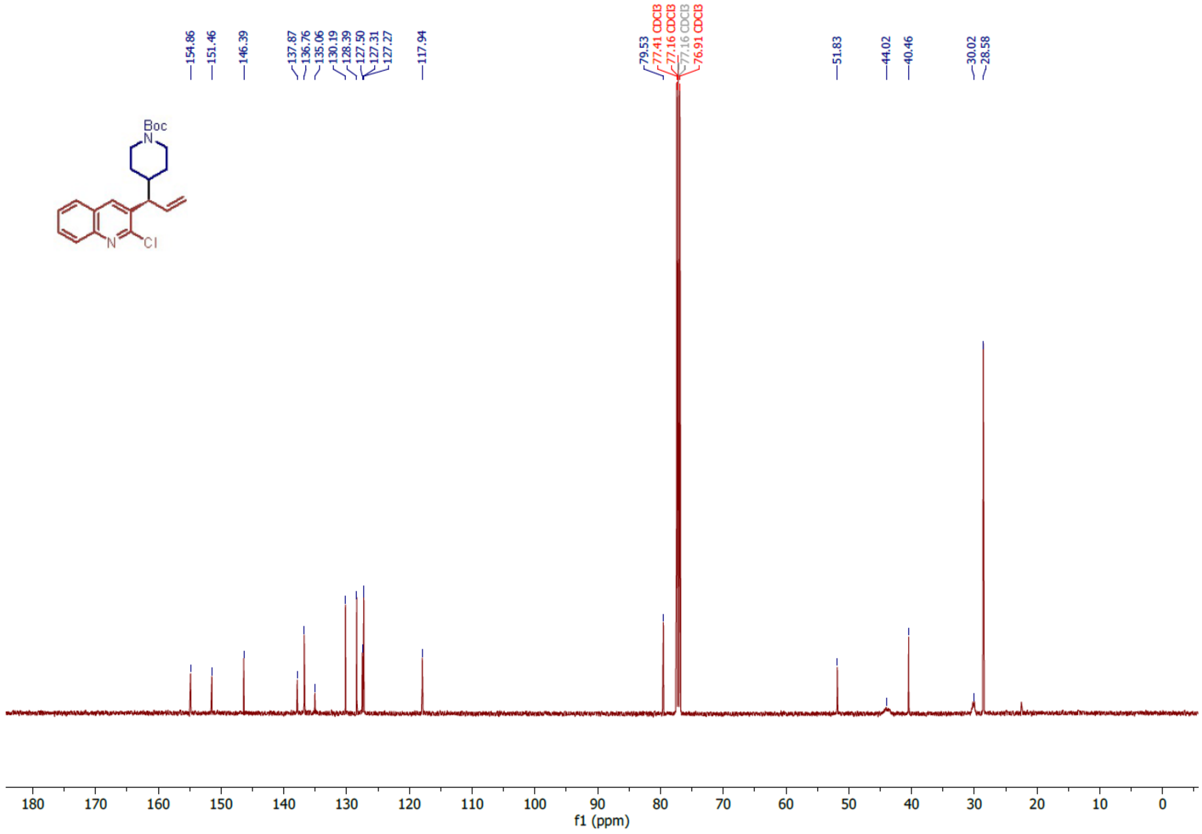

Compound 18

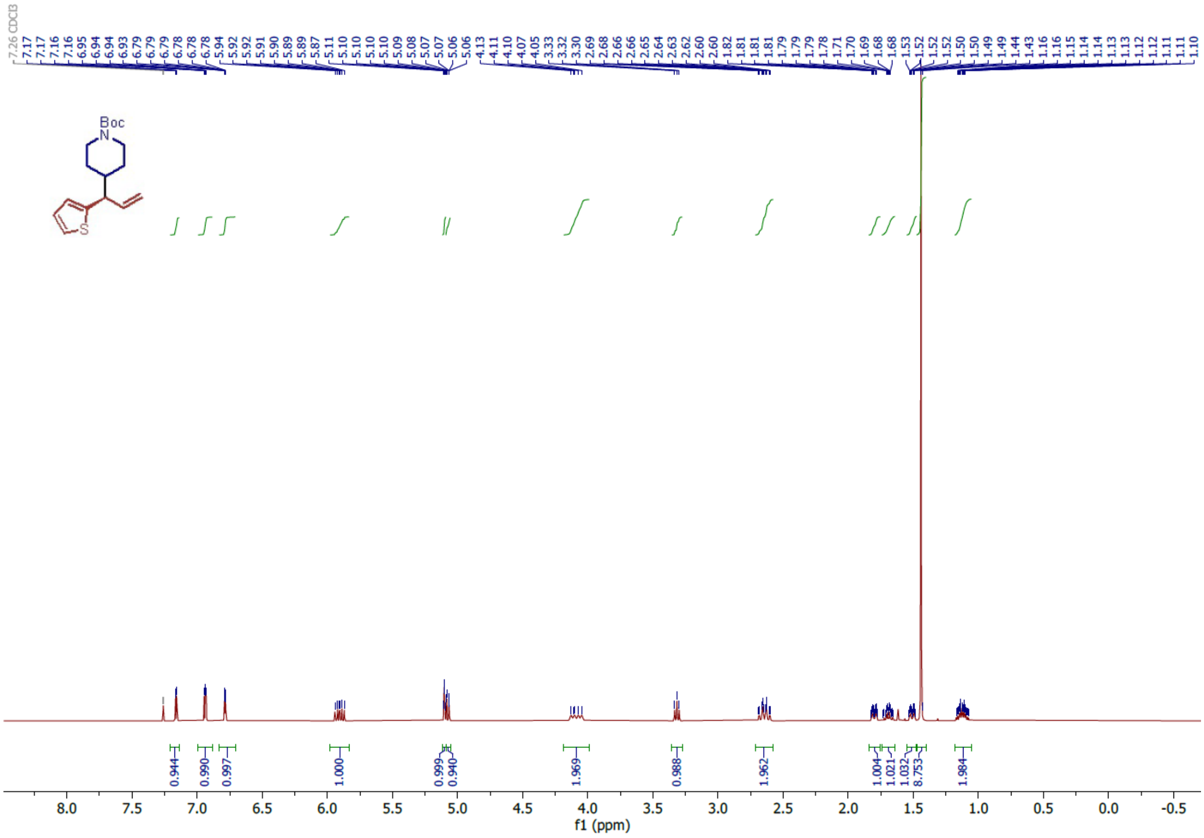

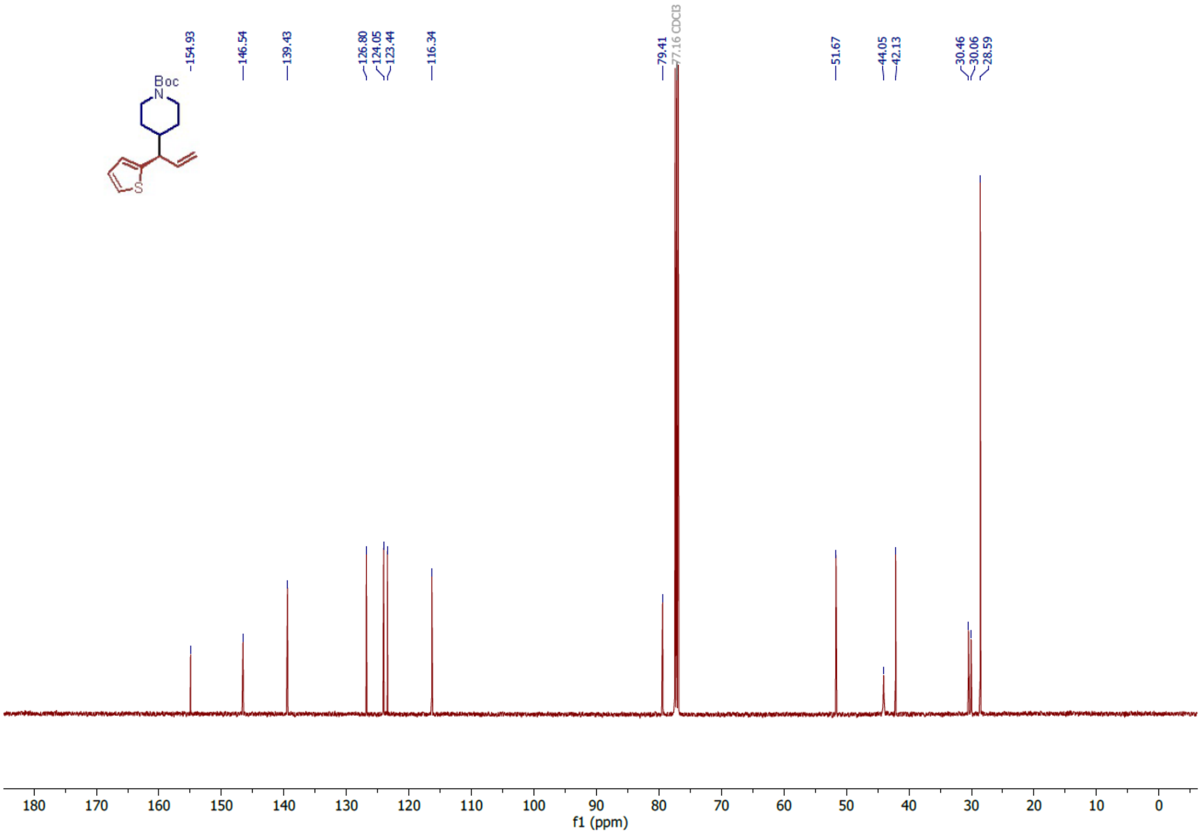

Compound 19

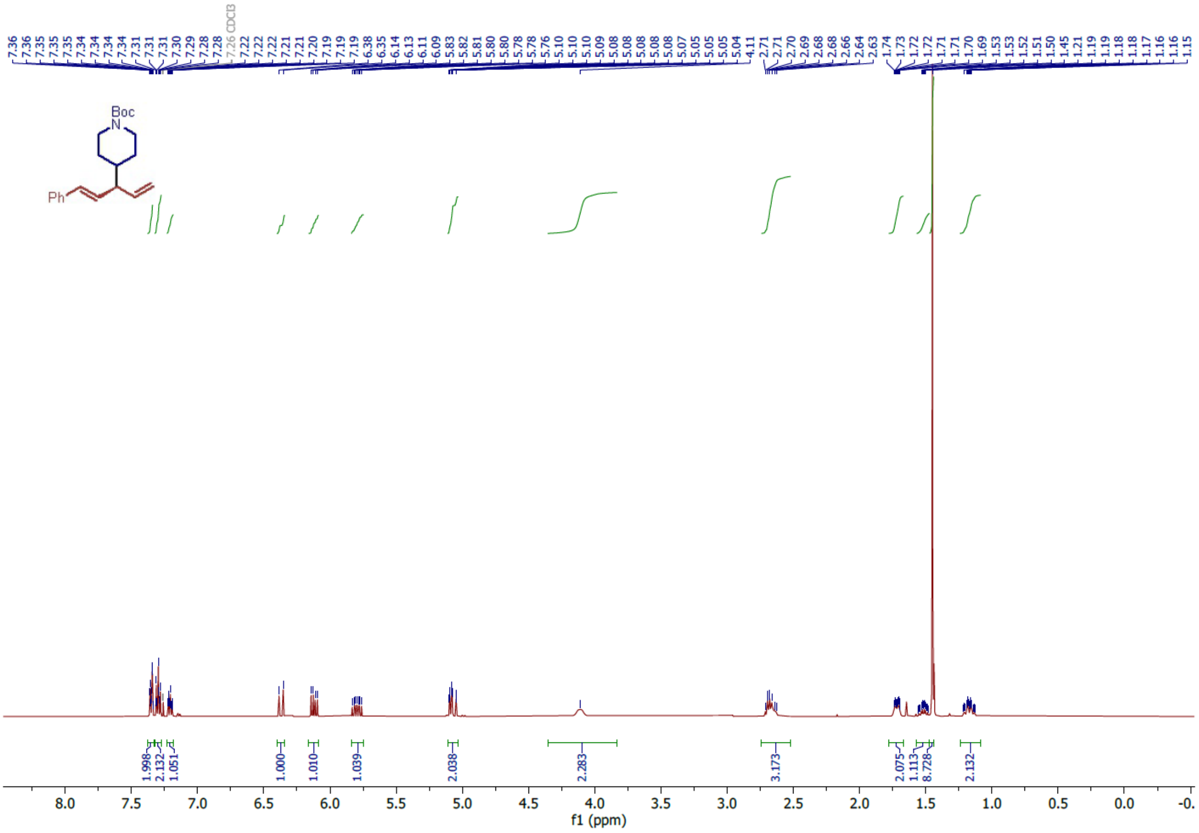

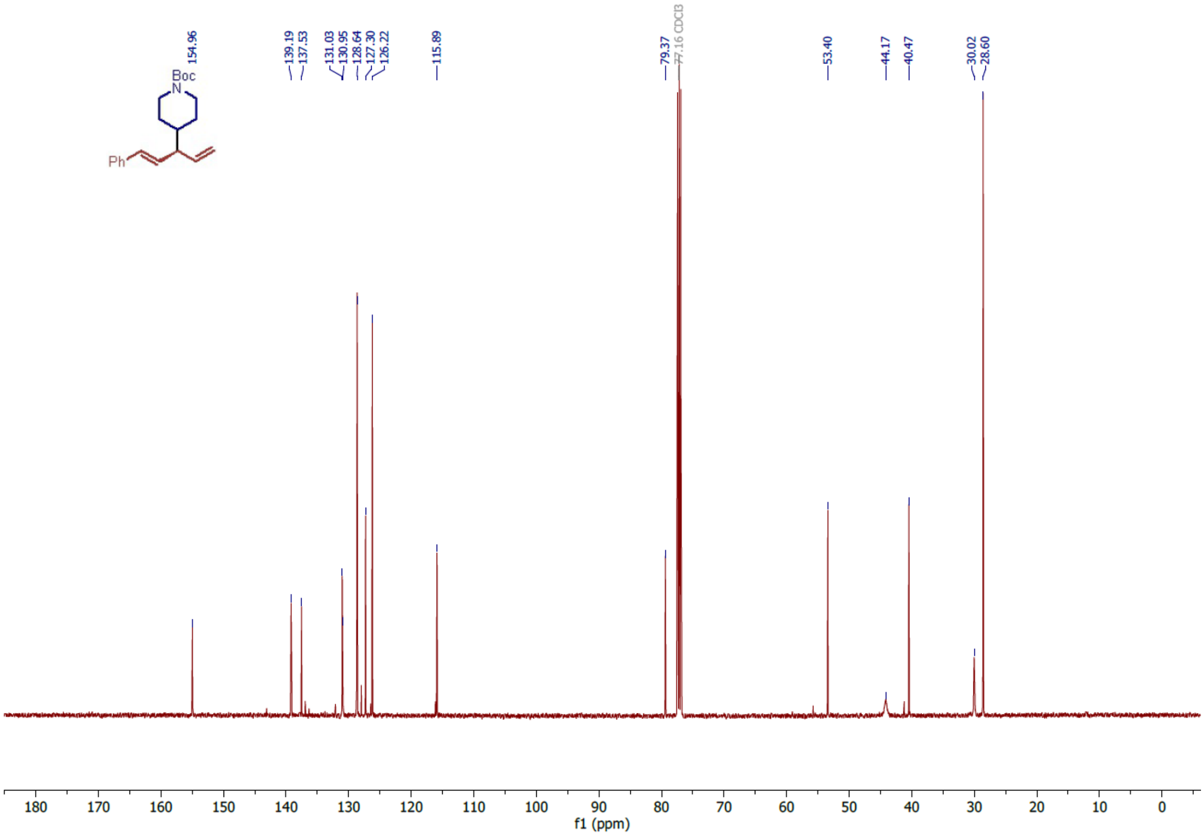

Compound 20

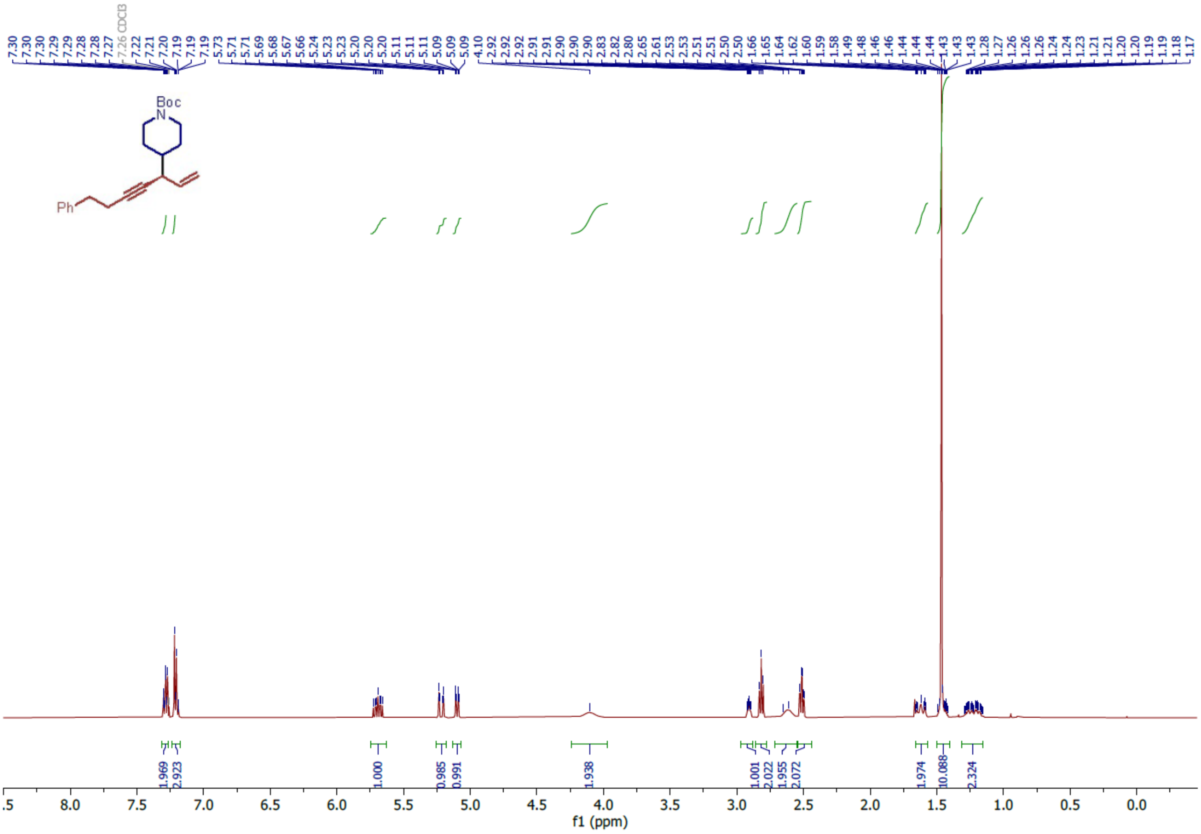
7.30 7.30 7.30 7.29 7.29 7.28 7.28 7.27 7.26 CDCl3 7.22 7.21 7.20 7.19 7.19 7.19 5.73 5.71 5.71 5.69 5.68 5.67 5.66 5.24 5.23 5.23 5.23 5.20 5.20 5.20 5.11 5.11 5.11 5.09 5.09 5.09 4.10 2.92 2.92 2.92 2.91 2.91 2.90 2.90 2.90 2.83 2.82 2.80 2.65 2.61 2.53 2.53 2.53 2.51 2.51 2.50 2.50 1.66 1.65 1.64 1.62 1.60 1.59 1.58 1.49 1.48 1.46 1.46 1.44 1.44 1.44 1.43 1.43 1.43 1.28 1.27 1.26 1.26 1.26 1.24 1.24 1.23 1.21 1.21 1.20 1.19 1.19 1.18 1.17
Boc
N
Ph
1.969
2.923
1.000
0.985
0.991
1.938
1.001
2.022
1.955
2.072
1.974
10.088
2.324
f1 (ppm)

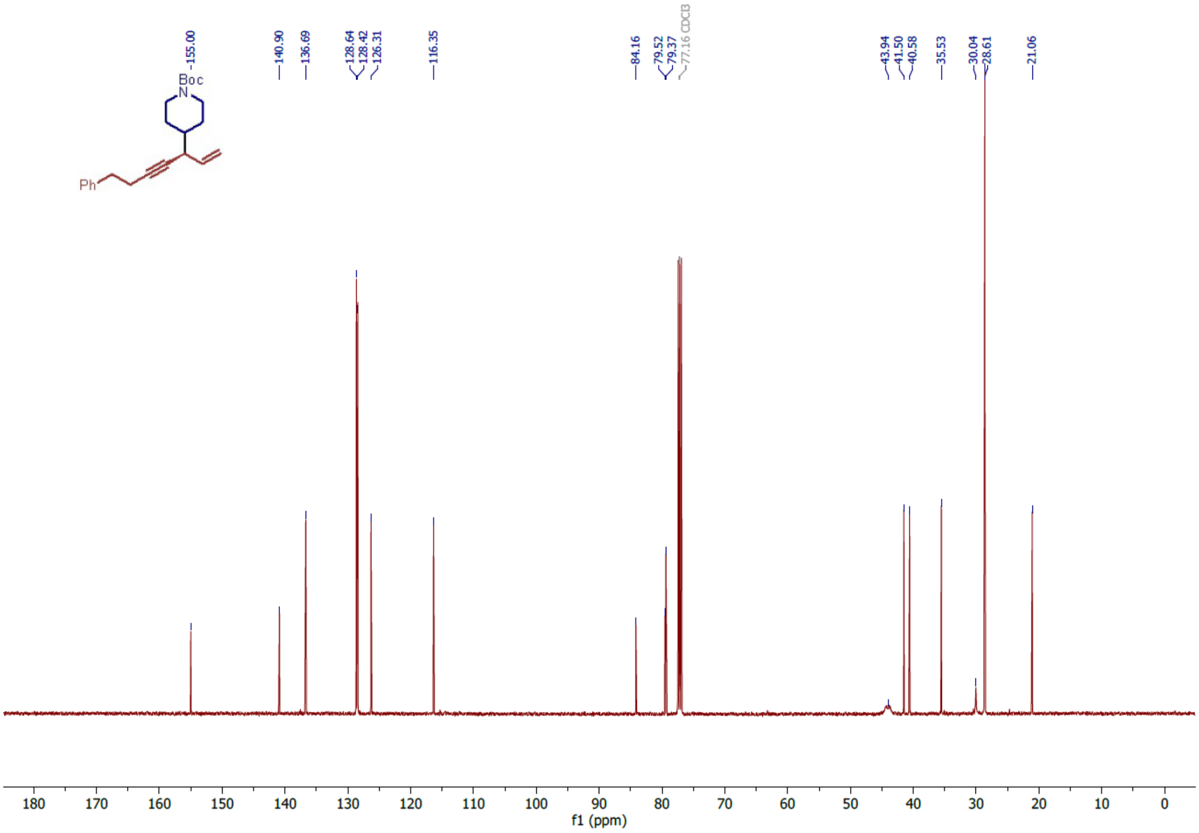
155.00
140.90
136.69
128.64
128.42
126.31
116.35
84.16
79.52
79.37
77.16 CDCl3
43.94
41.50
40.58
35.53
30.04
28.61
21.06
Boc
N
Ph
f1 (ppm)

Compound 21

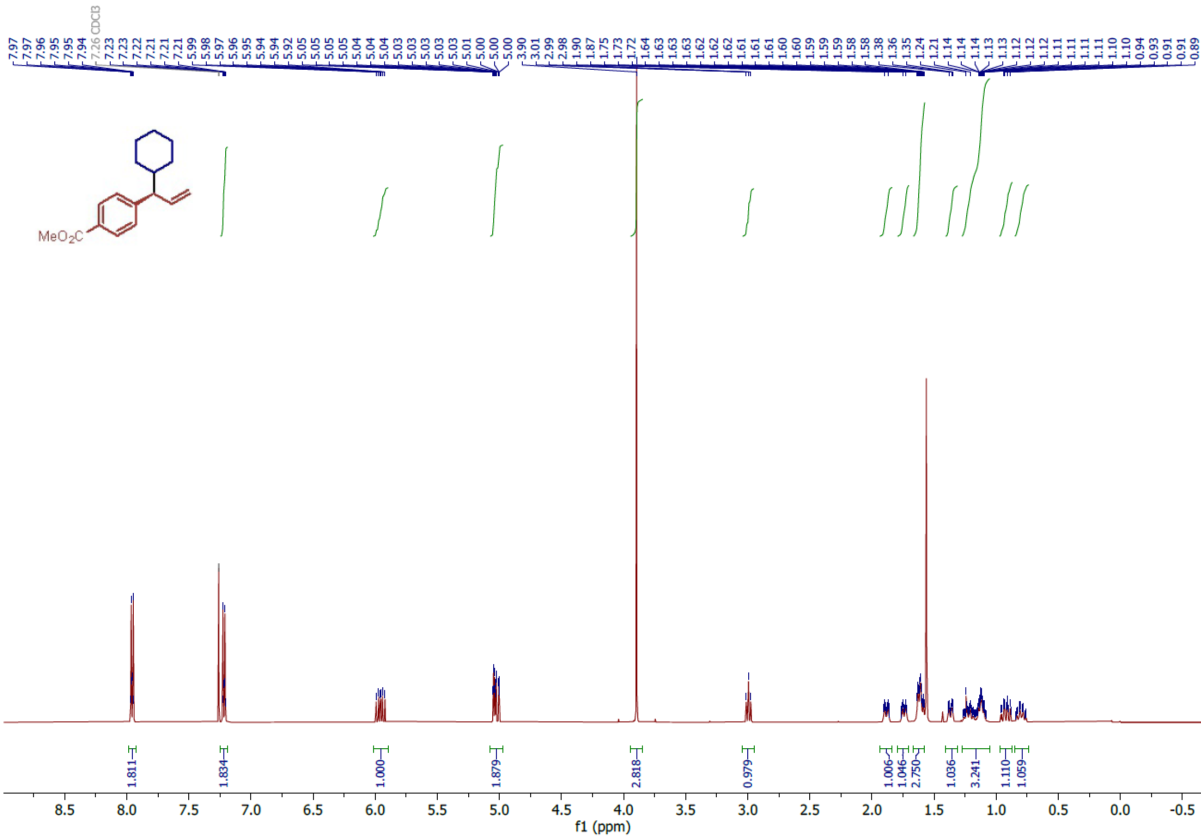
MeO2C
f1 (ppm)

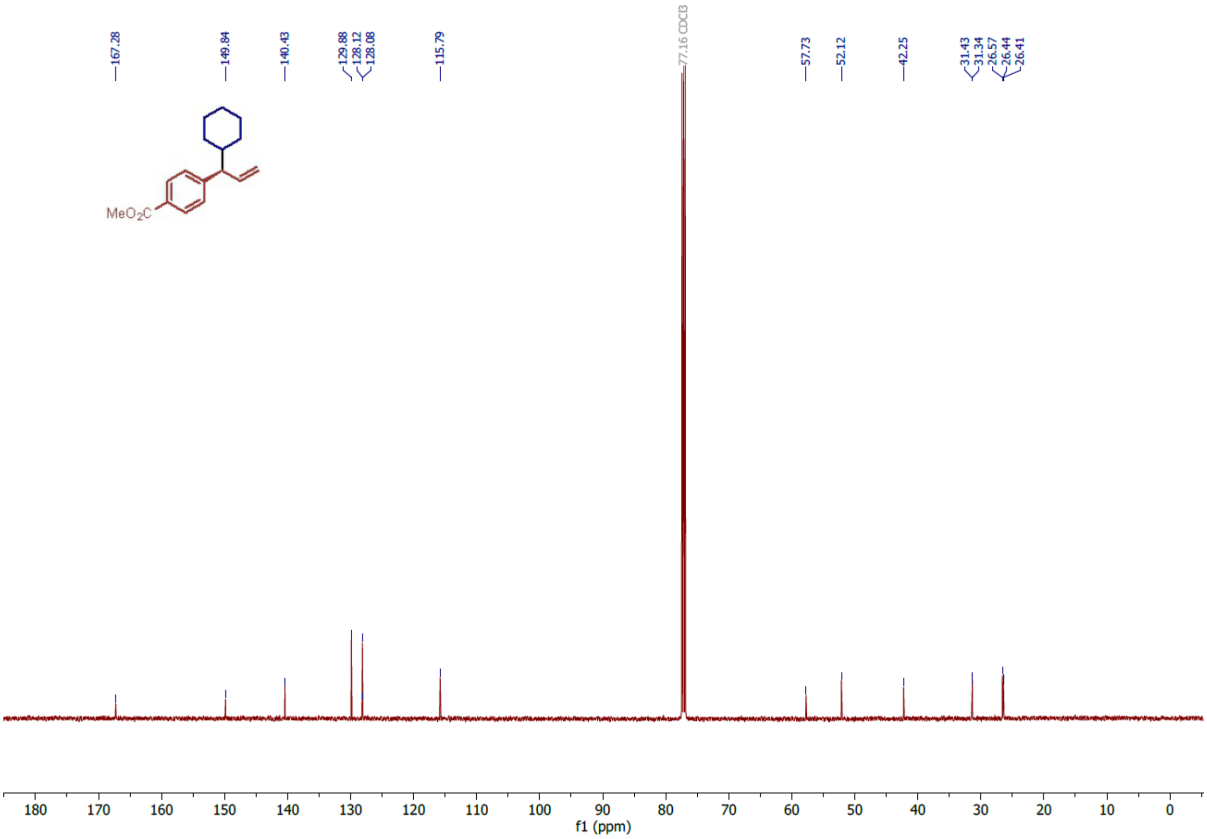
167.28
149.84
140.43
129.88
128.12
128.08
115.79
77.16 CDCl3
57.73
52.12
42.25
31.43
31.34
26.57
26.44
26.41
MeO2C
f1 (ppm)

Compound 22

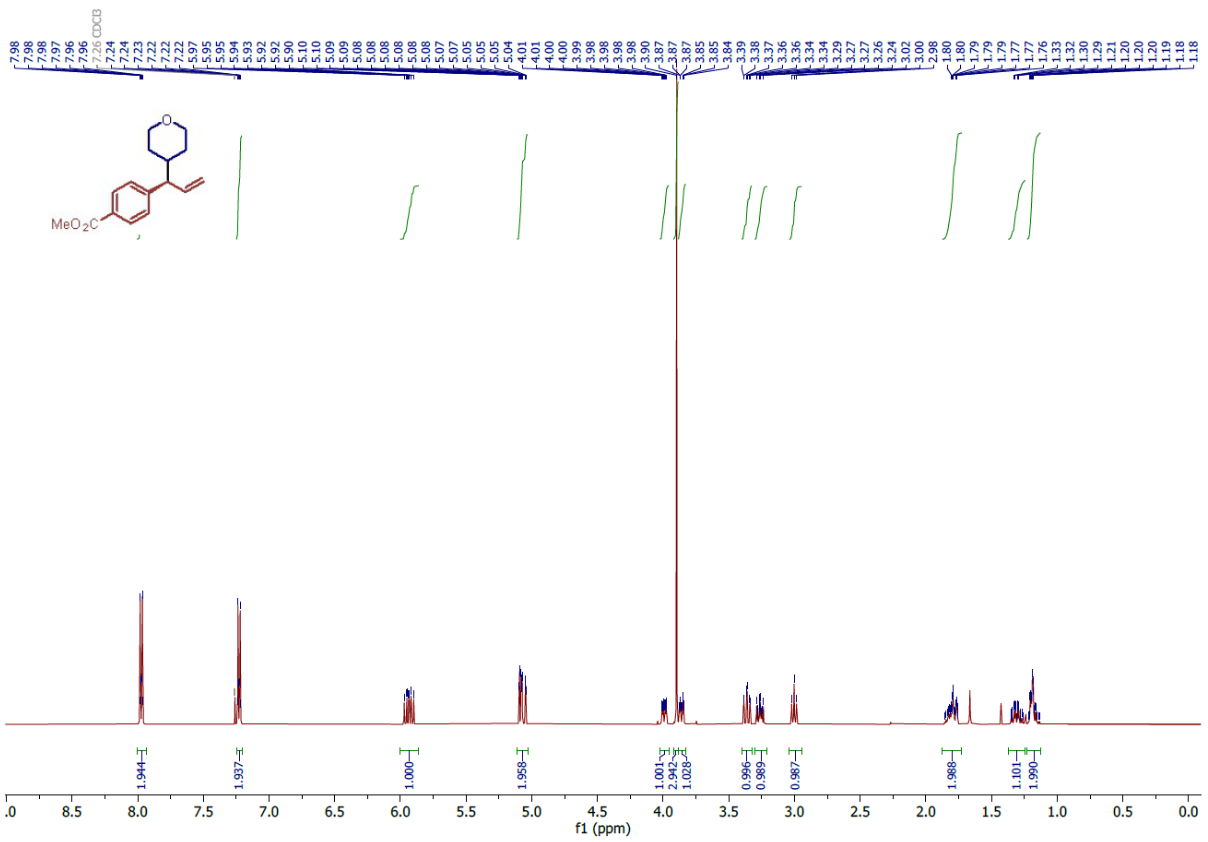

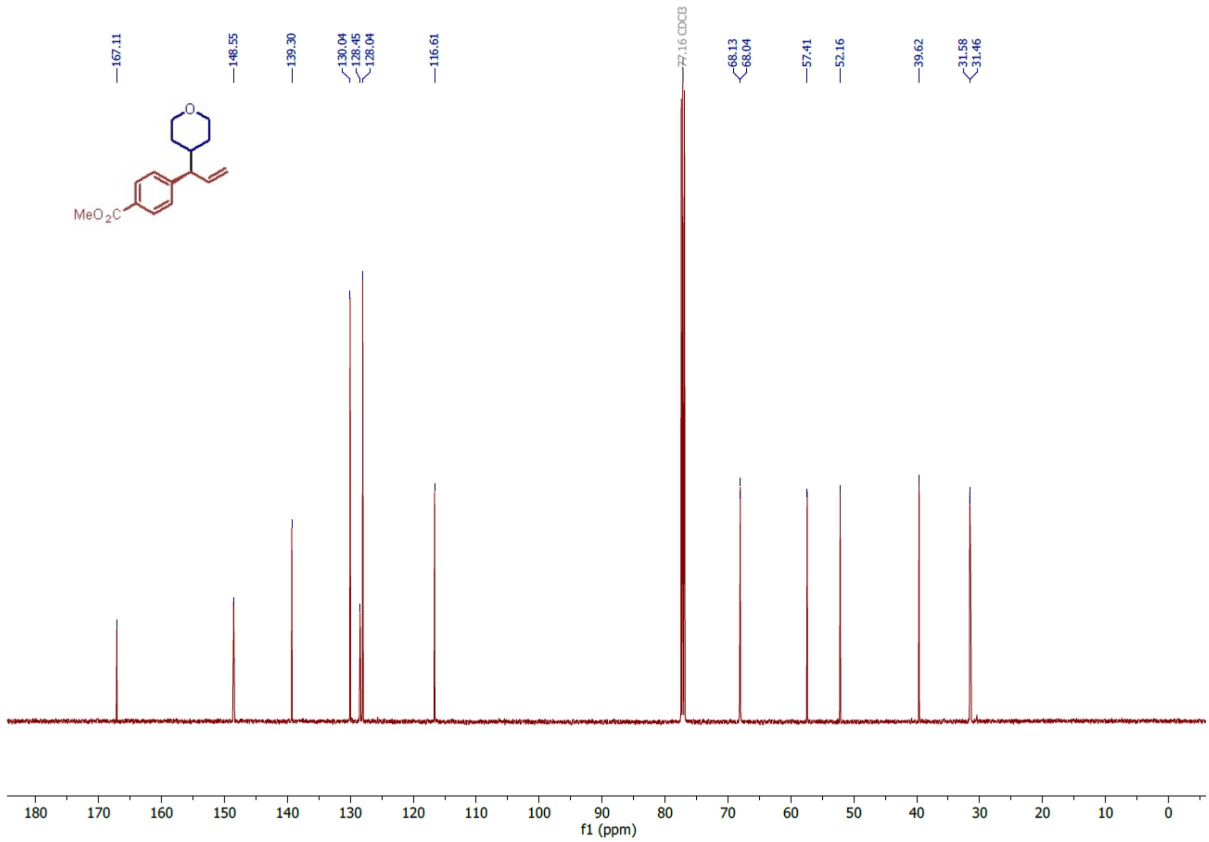

Compound 23

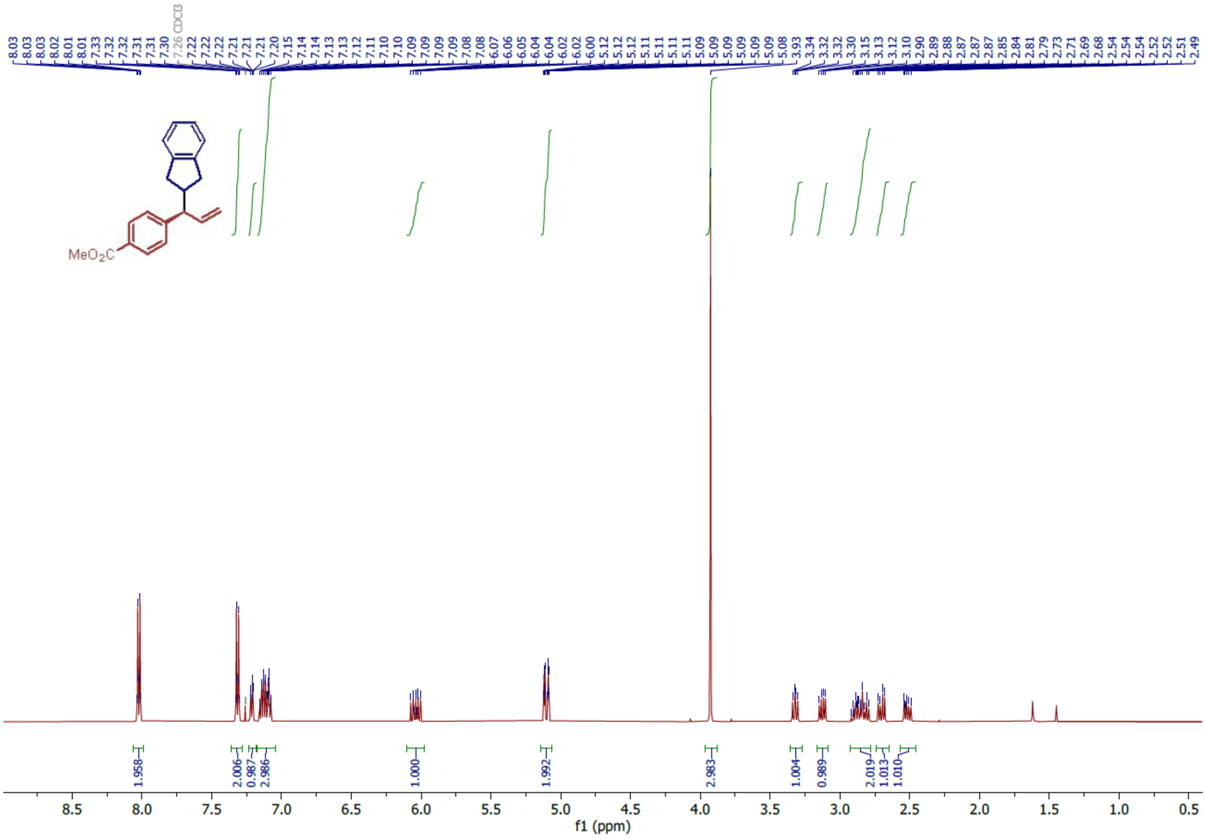

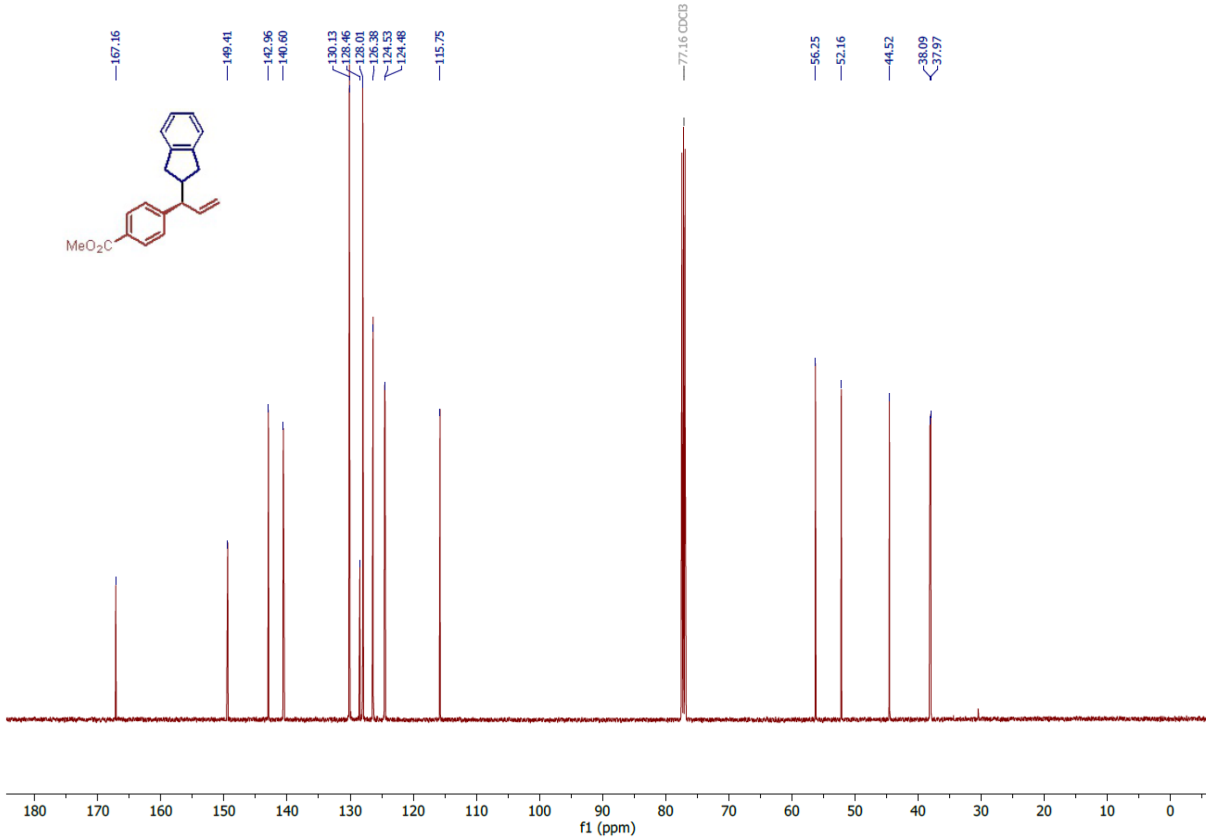

Compound 24

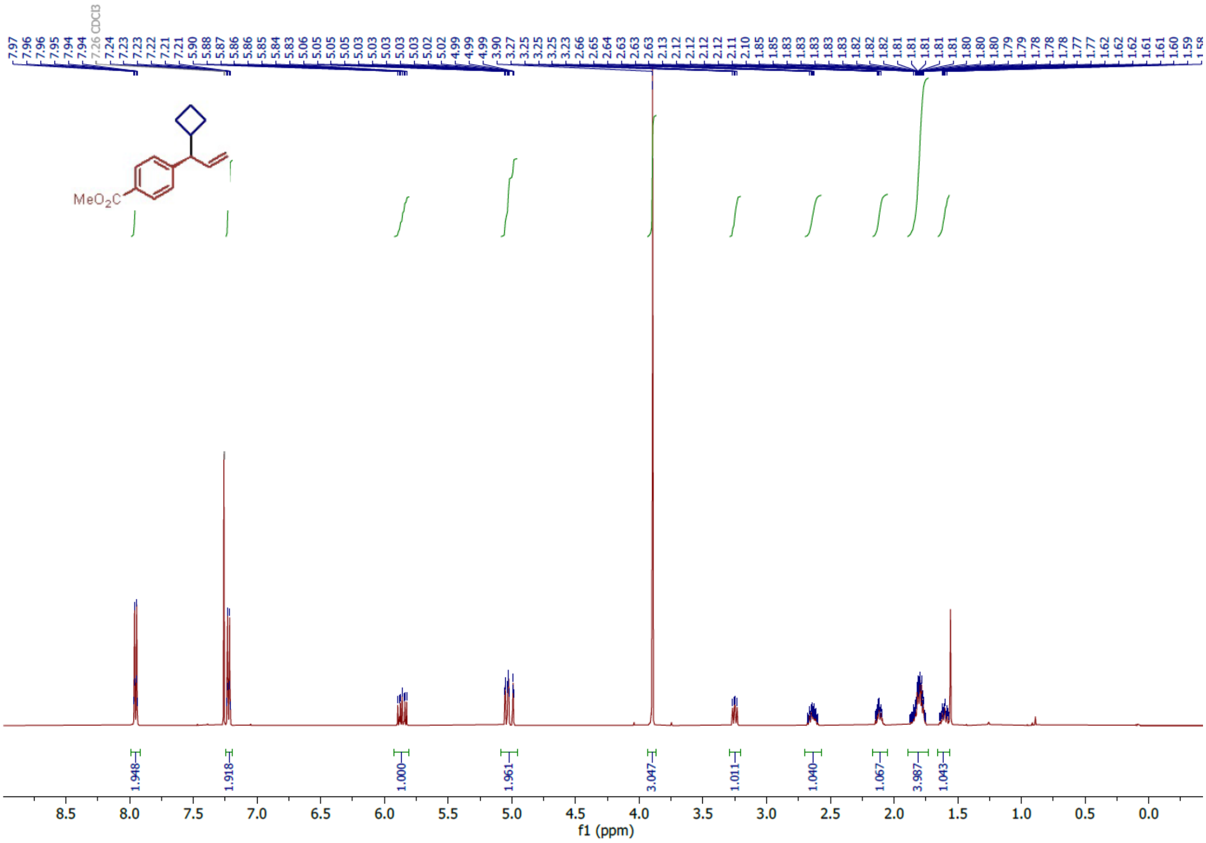

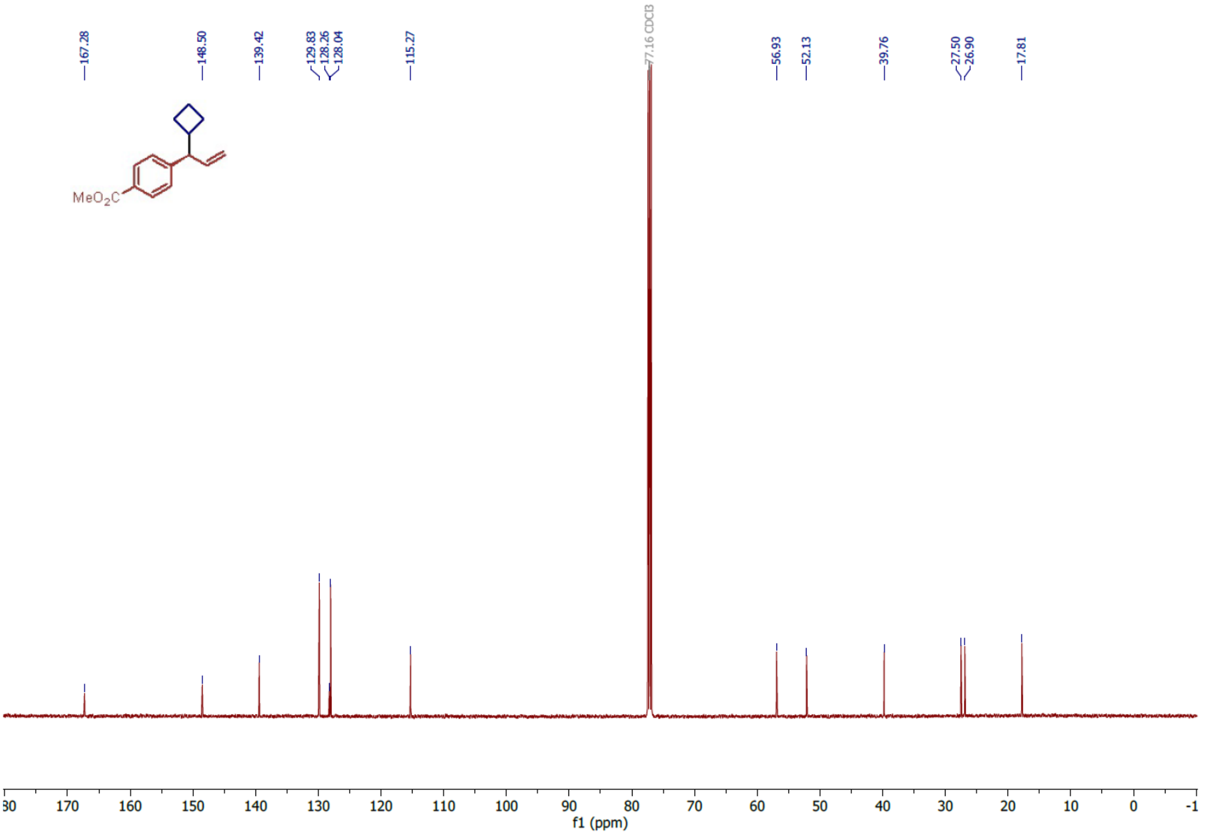

Compound 25

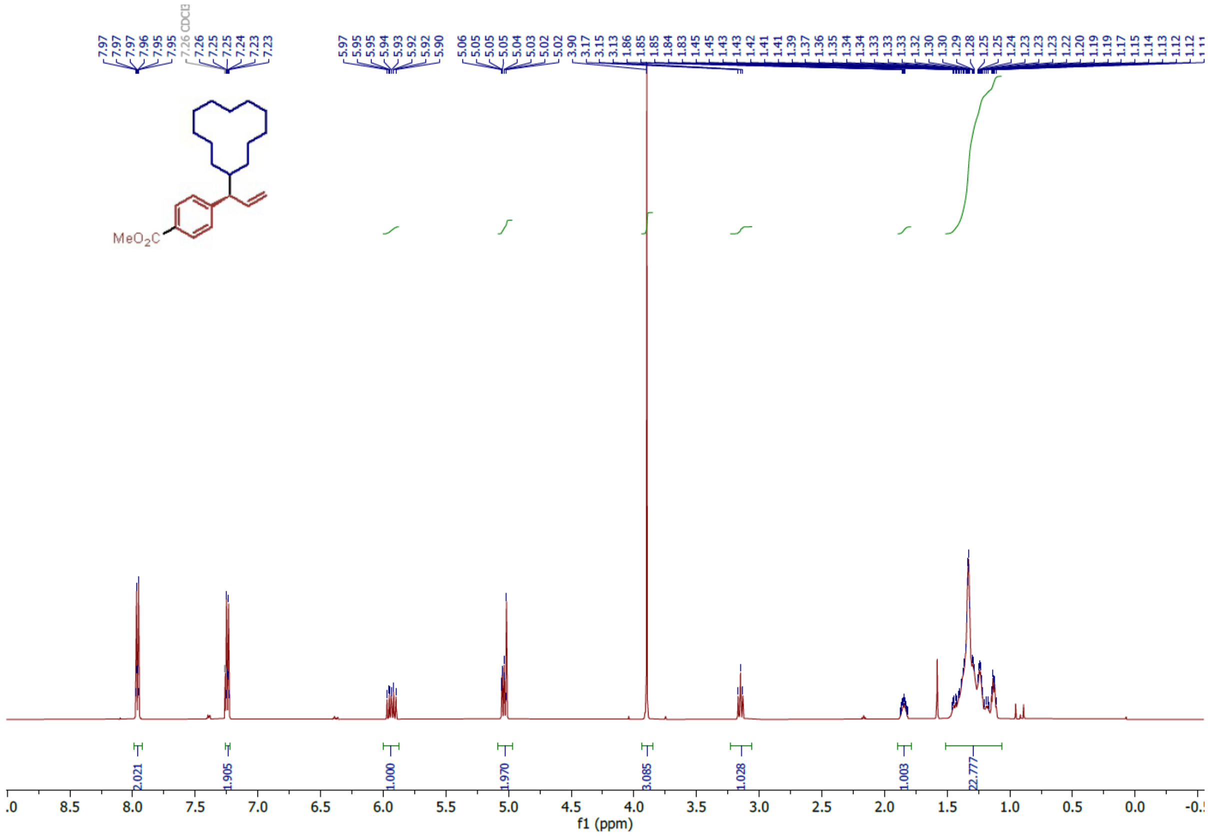
7.97
7.97
7.97
7.96
7.95
7.26 CDCl3
7.26
7.25
7.25
7.24
7.23
7.23
MeO2C
2.021
1.905
1.000
1.970
3.085
1.028
1.003
22.777
f1 (ppm)

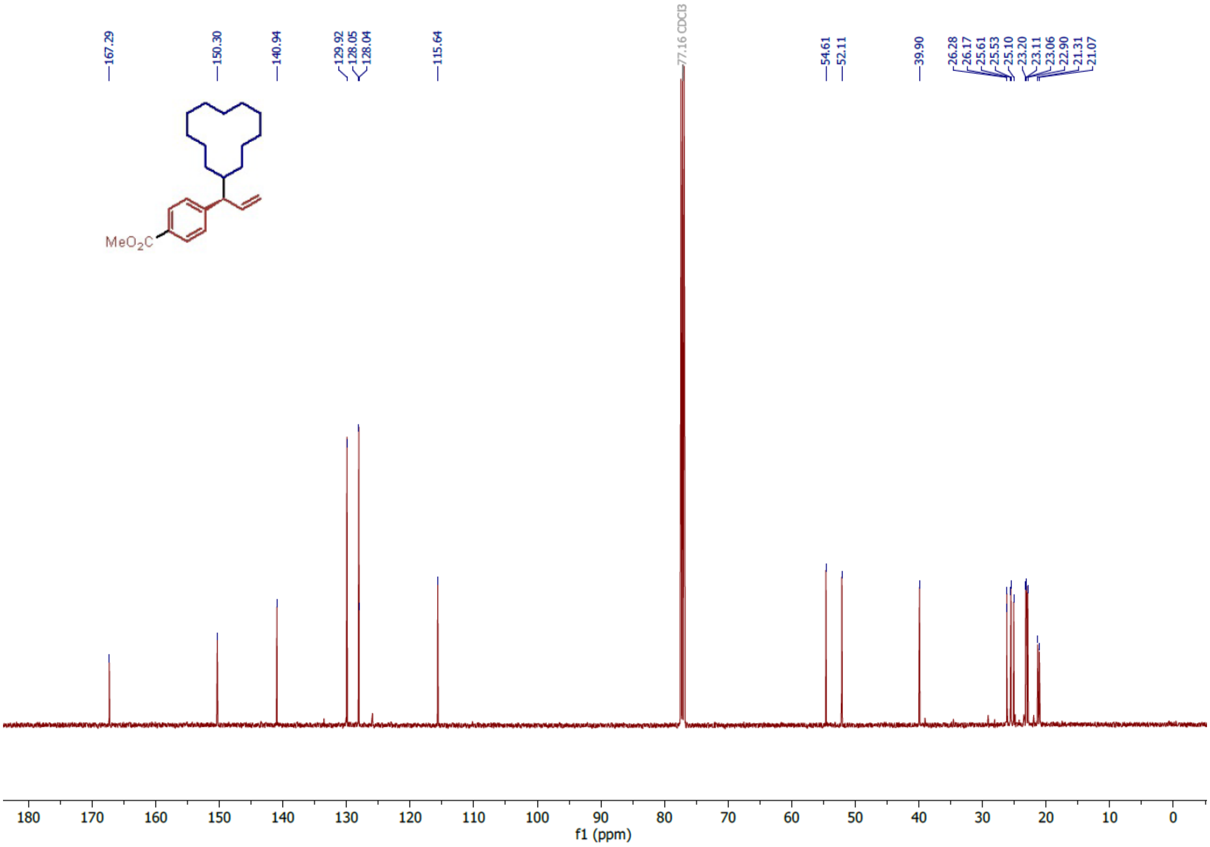
167.29
150.30
140.94
129.92
128.05
128.04
115.64
77.16 CDCl3
54.61
52.11
39.90
26.28
26.17
25.61
25.53
25.10
23.20
23.11
23.06
22.90
21.31
21.07
MeO2C
f1 (ppm)

Compound 27

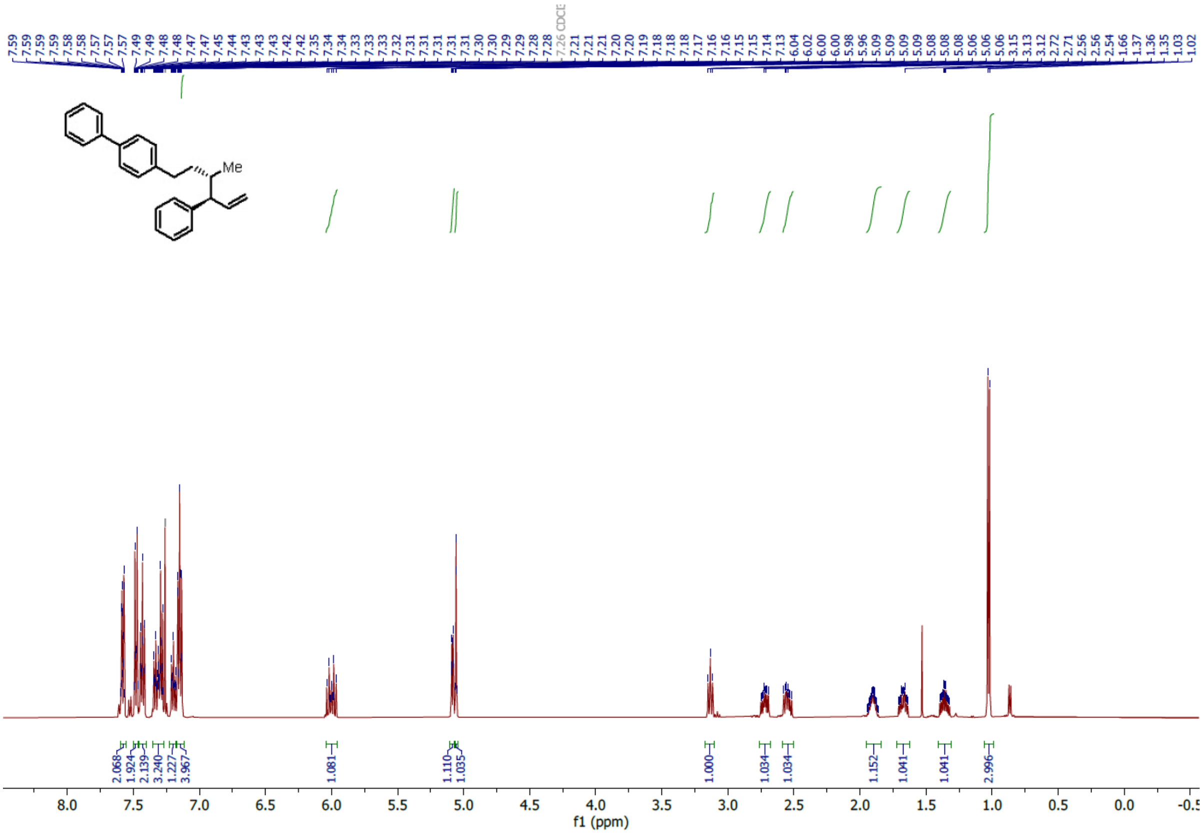

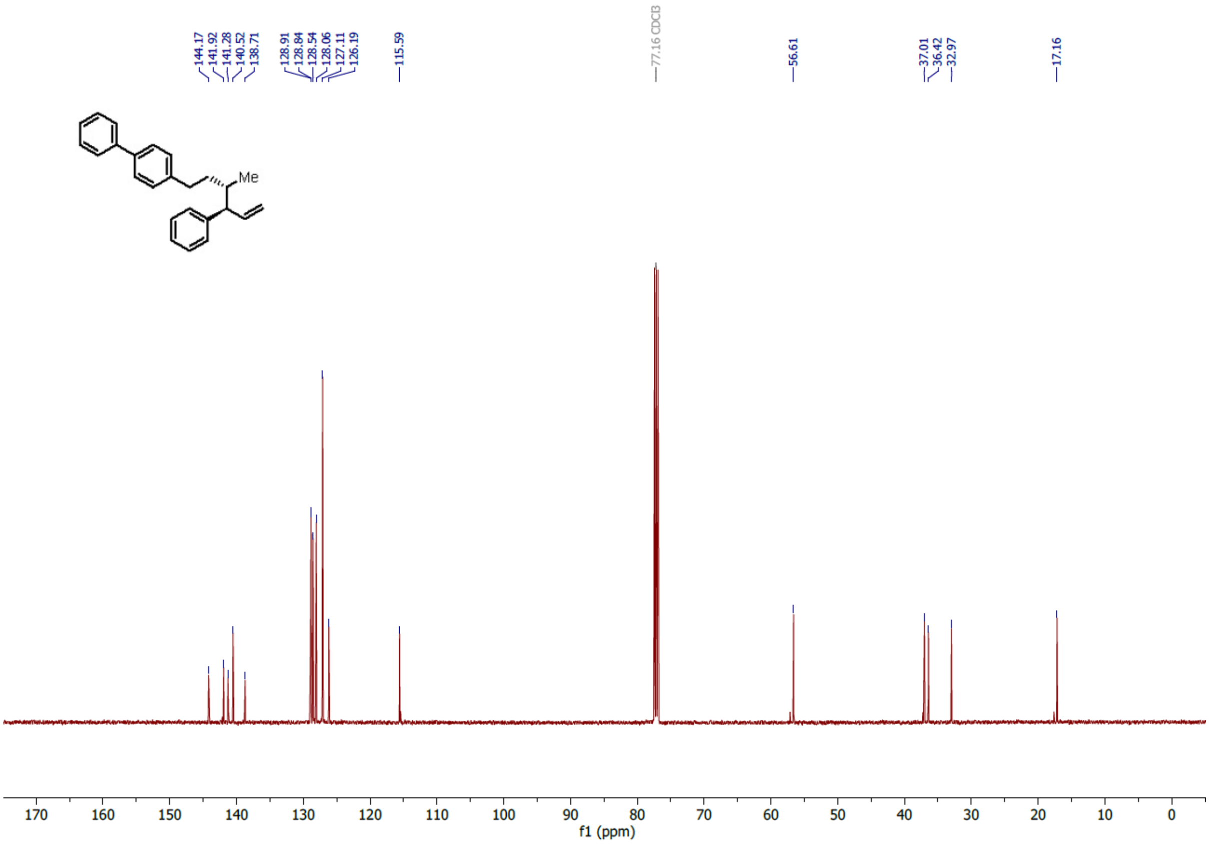

Compound 28

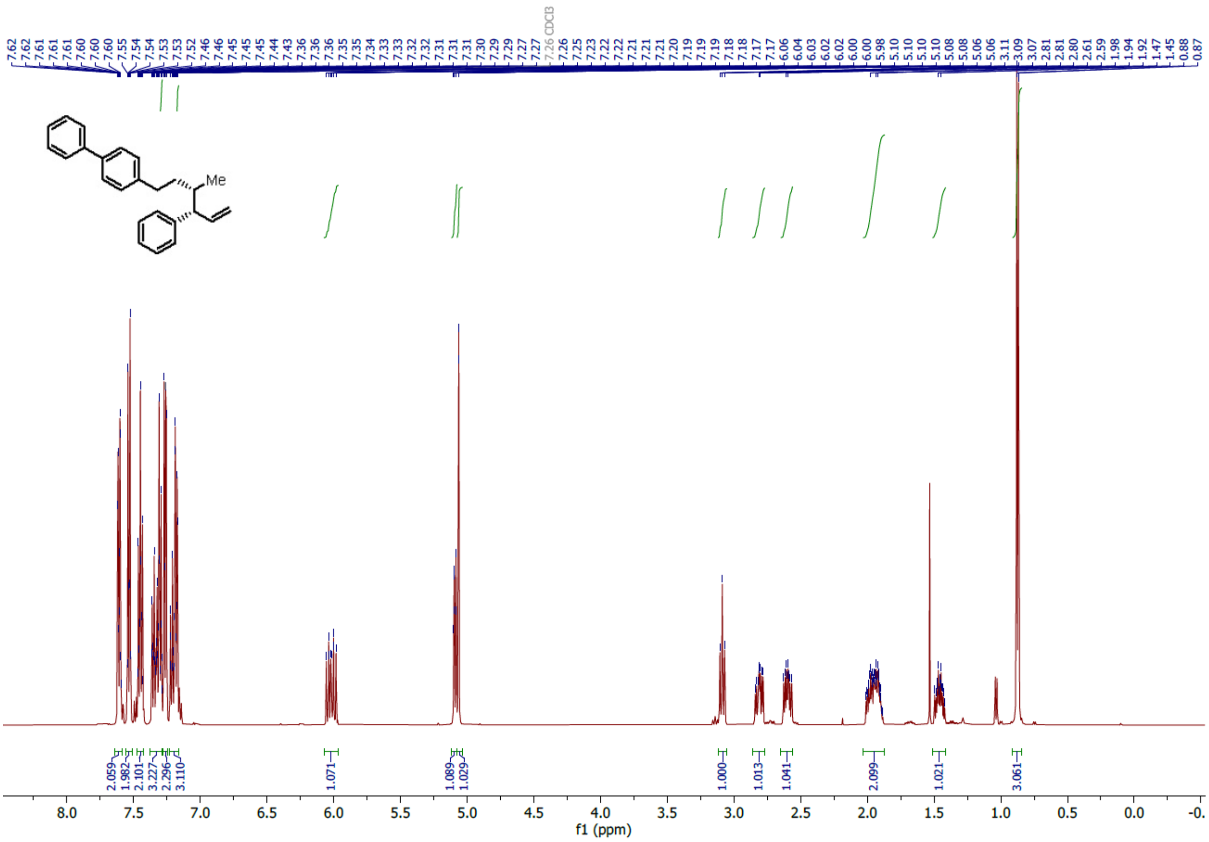

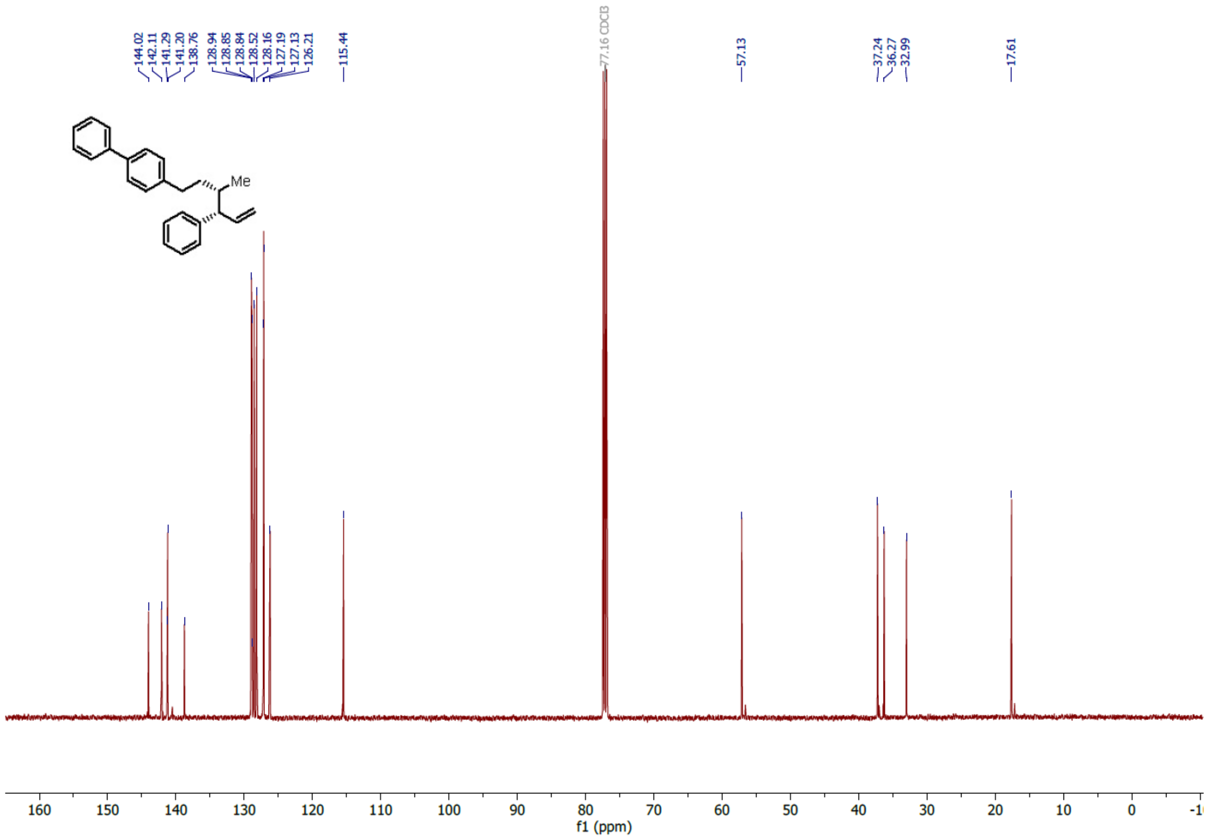

Compound 30

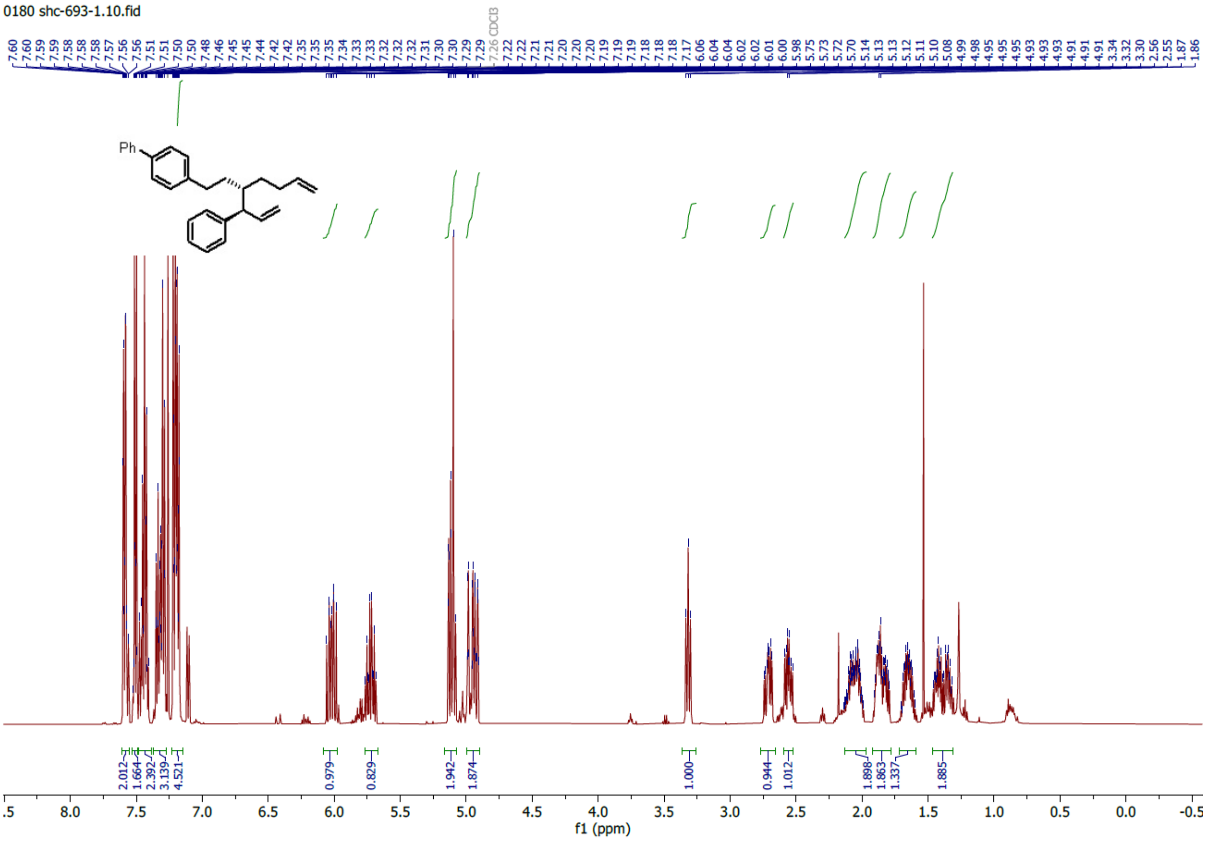
0180 shc-693-1.10.fid
7.60 7.60 7.59 7.59 7.58 7.58 7.58 7.57 7.56 7.56 7.51 7.51 7.50 7.50 7.48 7.46 7.45 7.45 7.44 7.42 7.42 7.35 7.35 7.34 7.33 7.33 7.32 7.32 7.32 7.31 7.30 7.30 7.29 7.29
7.26 CDCl3
7.22 7.22 7.21 7.21 7.20 7.20 7.20 7.19 7.19 7.19 7.18 7.18 7.18 7.17 6.06 6.04 6.04 6.02 6.02 6.01 6.00 5.98 5.75 5.73 5.72 5.70 5.14 5.13 5.13 5.12 5.11 5.10 5.08 4.99 4.98 4.95 4.95 4.95 4.93 4.93 4.93 4.91 4.91 4.91 3.34 3.32 3.30 2.56 2.55 1.87 1.86
Ph
2.012 1.664 2.392 3.139 4.521 0.979 0.829 1.942 1.874 1.000 0.944 1.012 1.898 1.863 1.337 1.885
f1 (ppm)

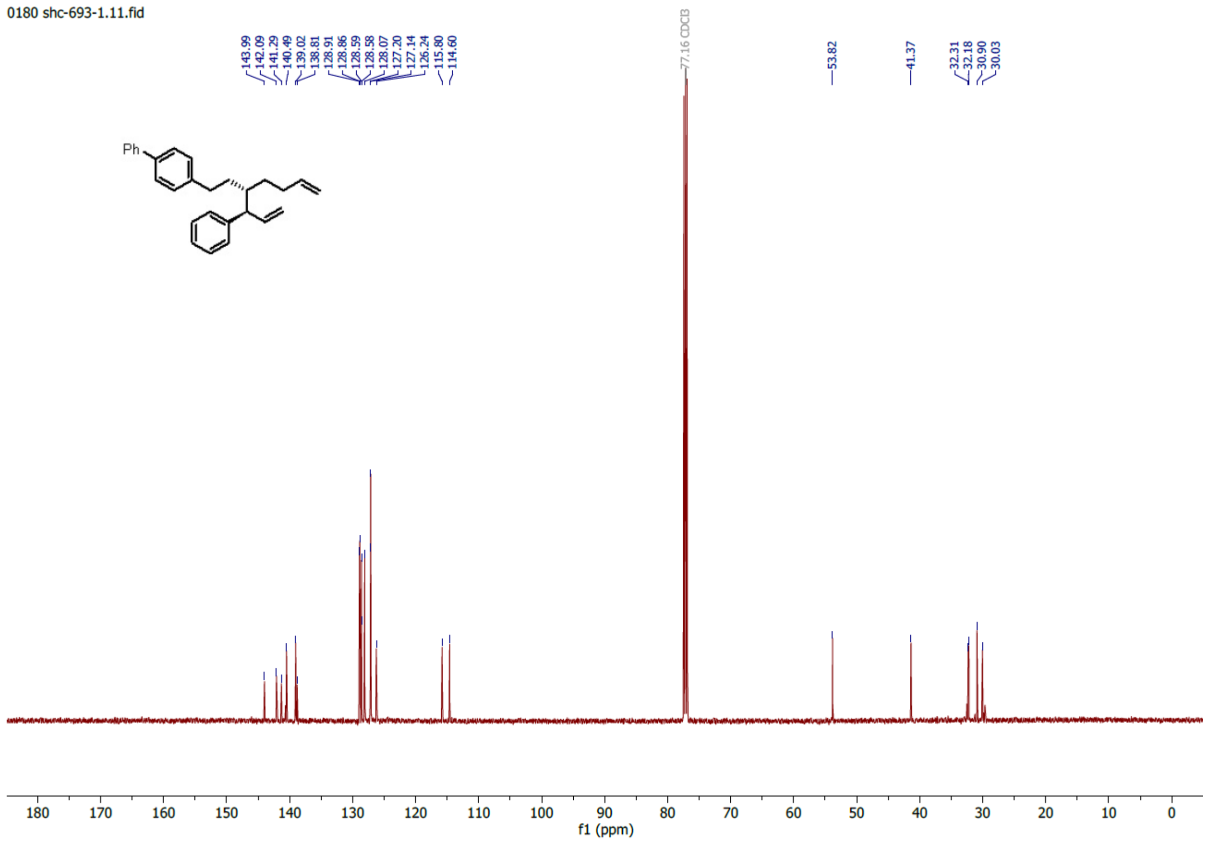
0180 shc-693-1.11.fid
143.99 142.09 141.29 140.49 139.02 138.81 128.91 128.86 128.59 128.58 128.07 127.20 127.14 126.24 115.80 114.60
77.16 CDCl3
53.82
41.37
32.31 32.18 30.90 30.03
Ph
f1 (ppm)

Compound 31

0181 shc-693-2.10.fid

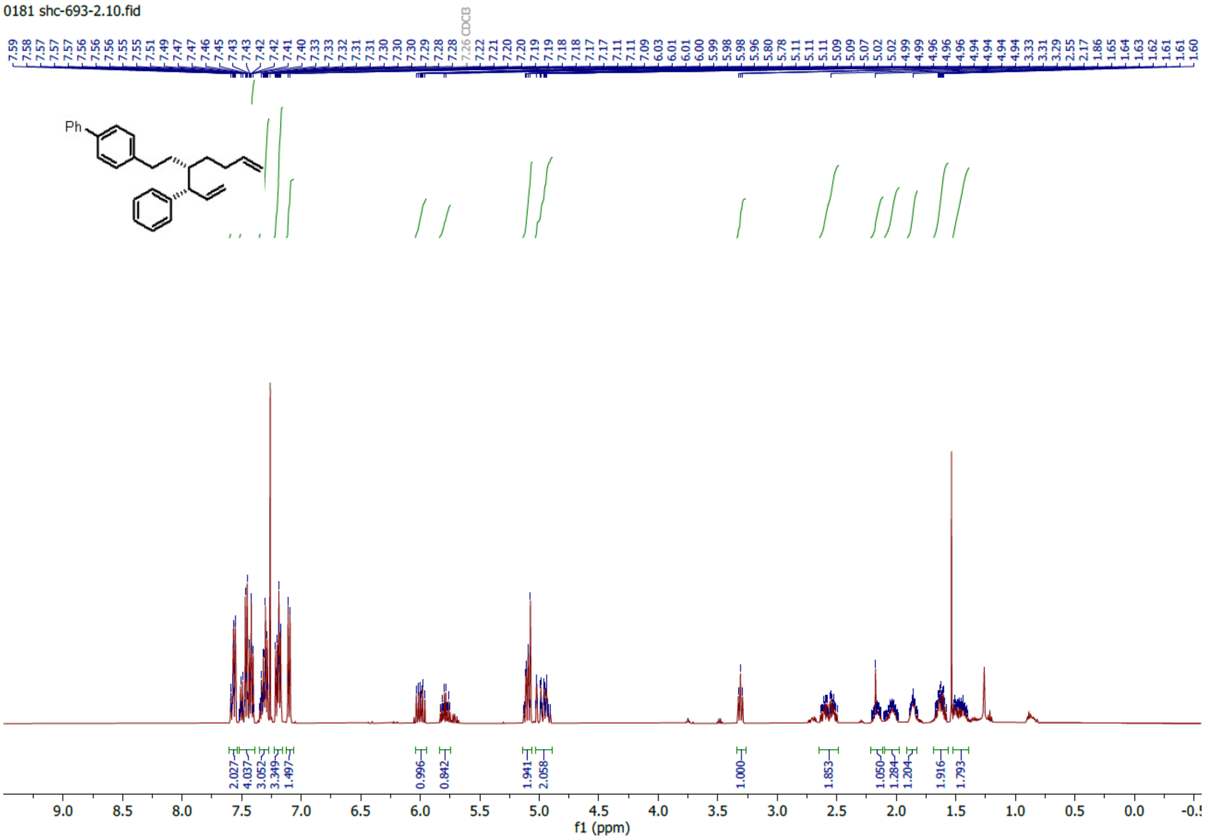

0181 shc-693-2.11.fid

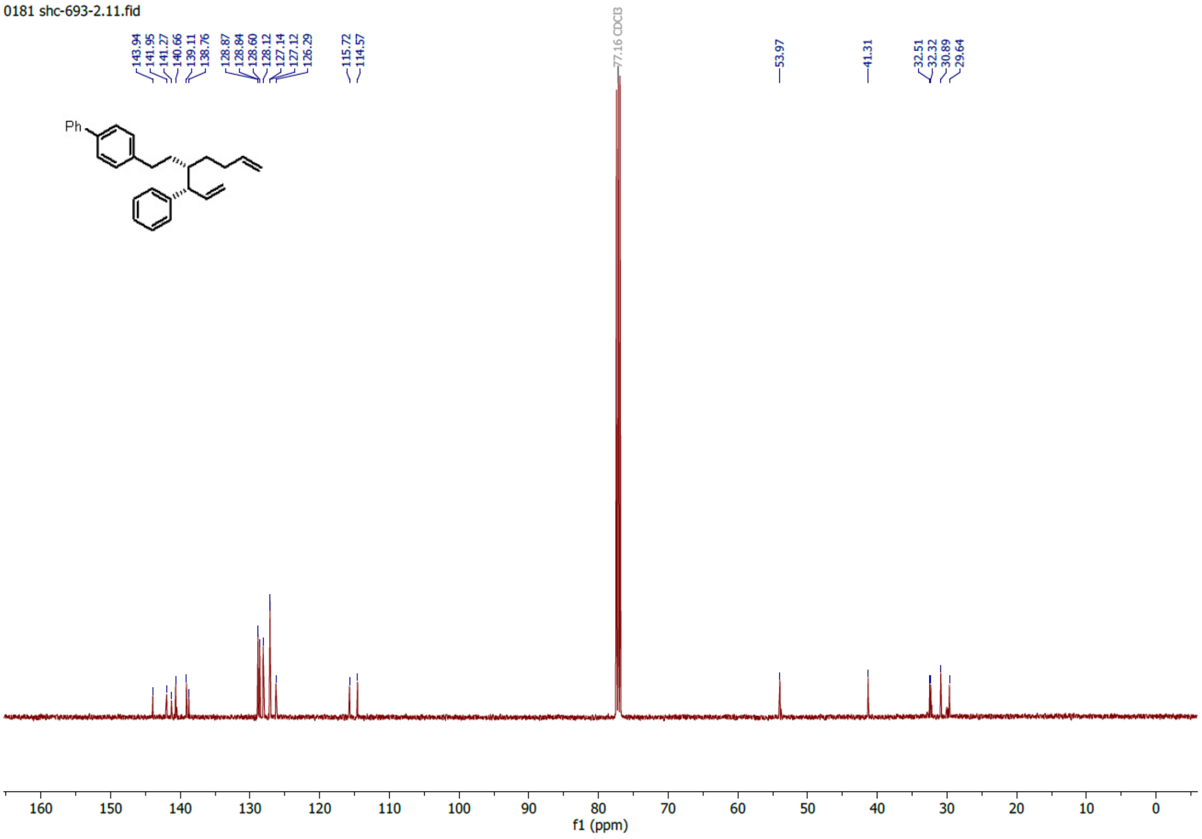

Compound 33

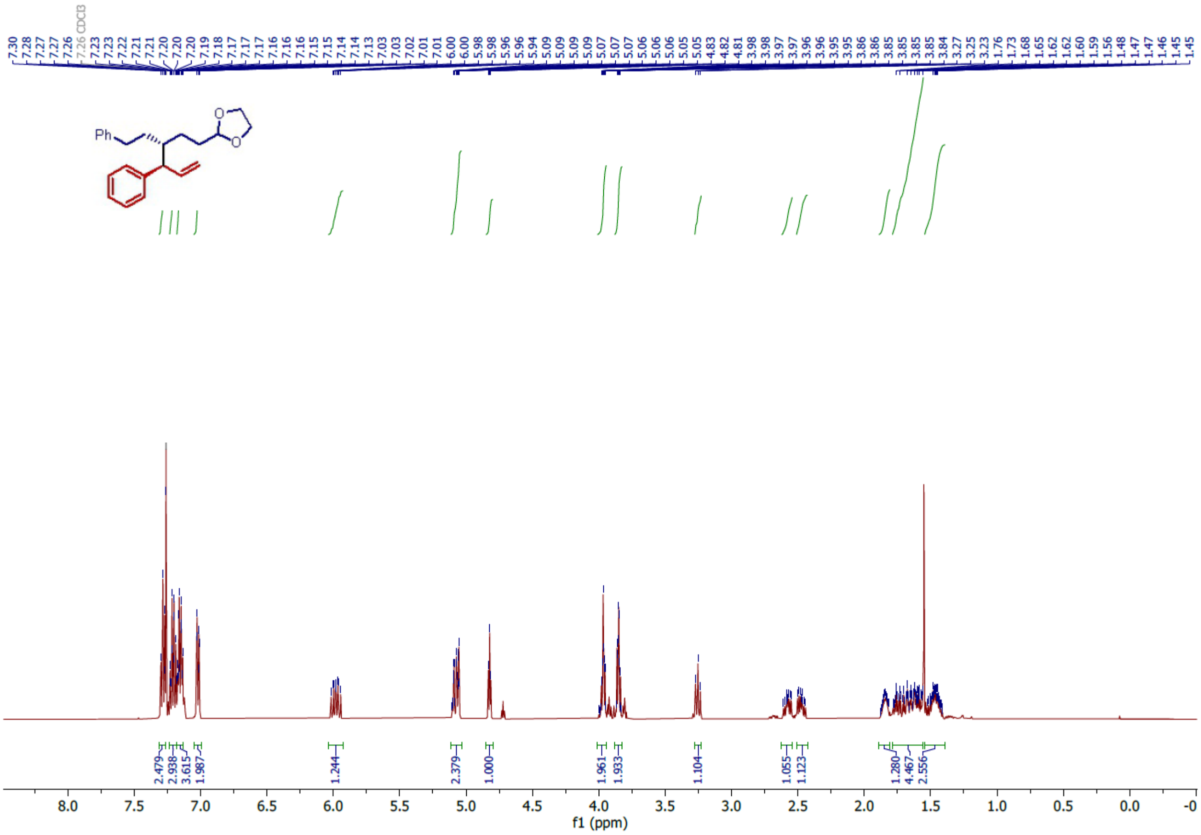

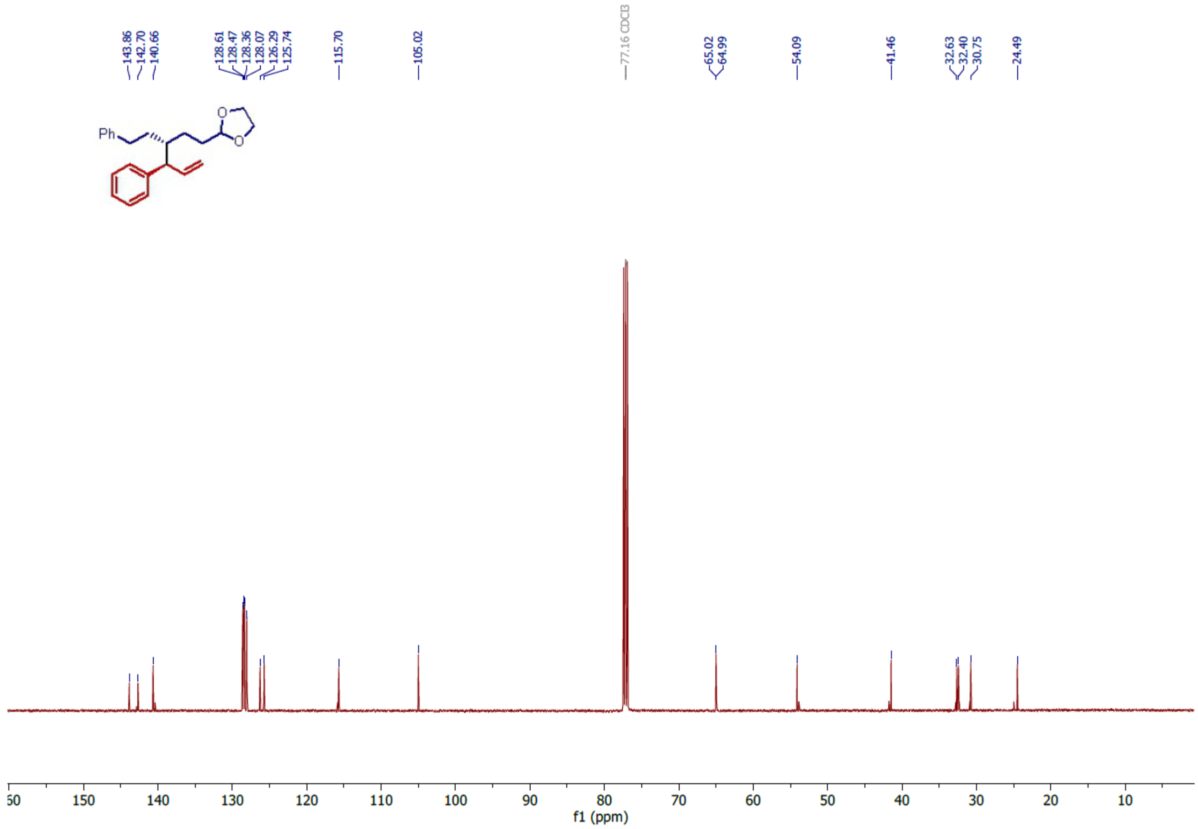

Compound 34

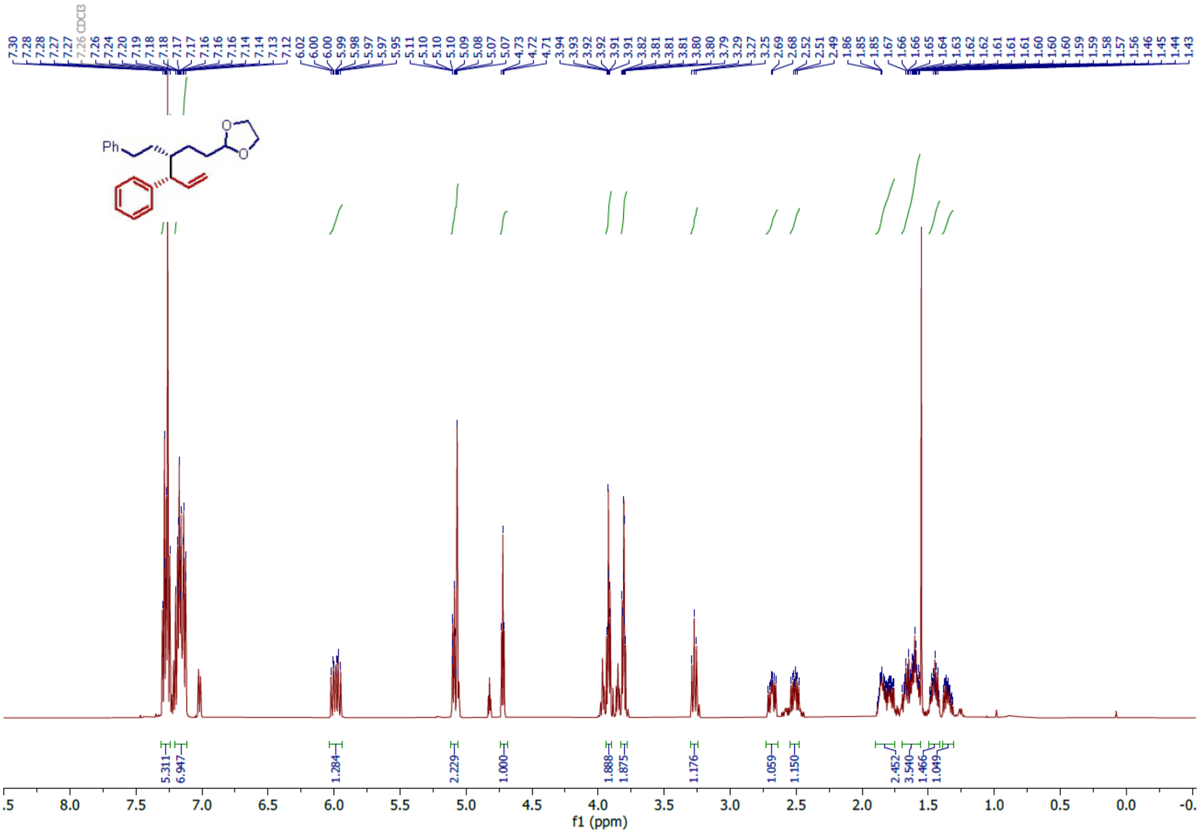

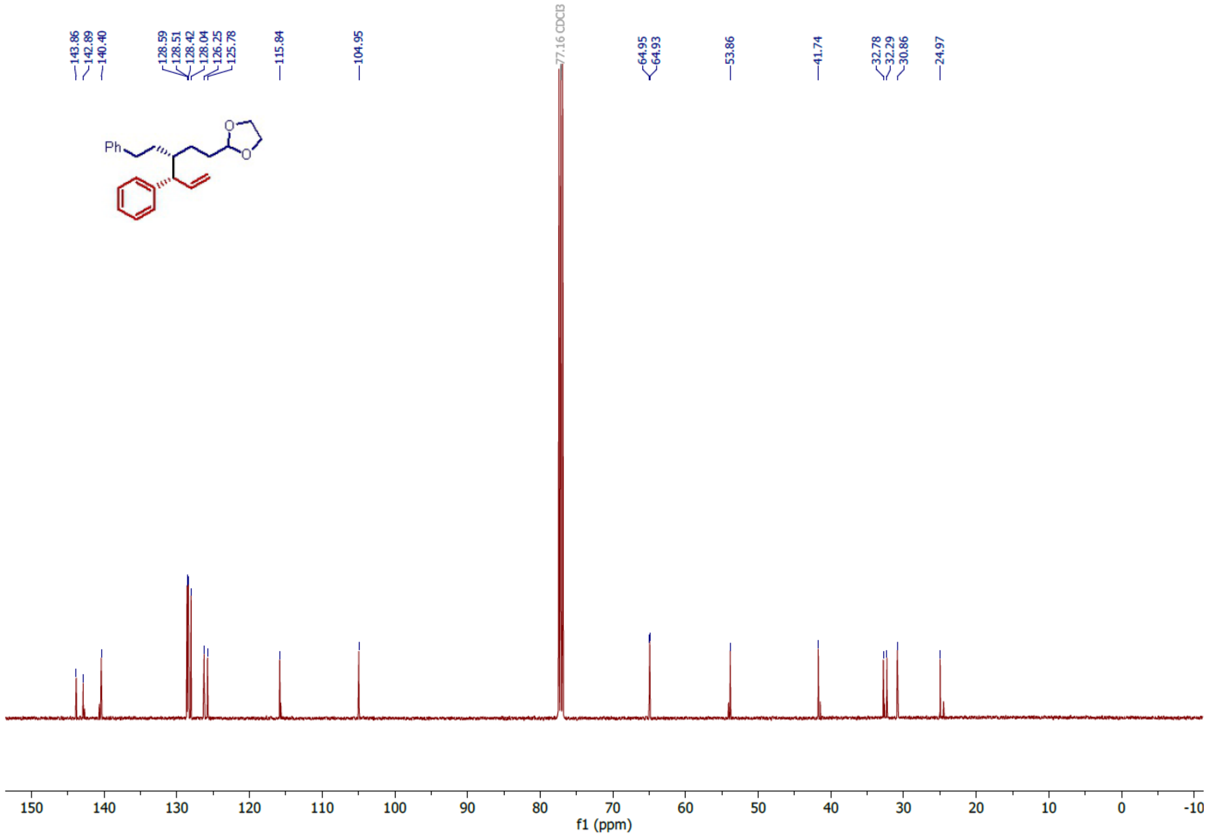

Compound 36

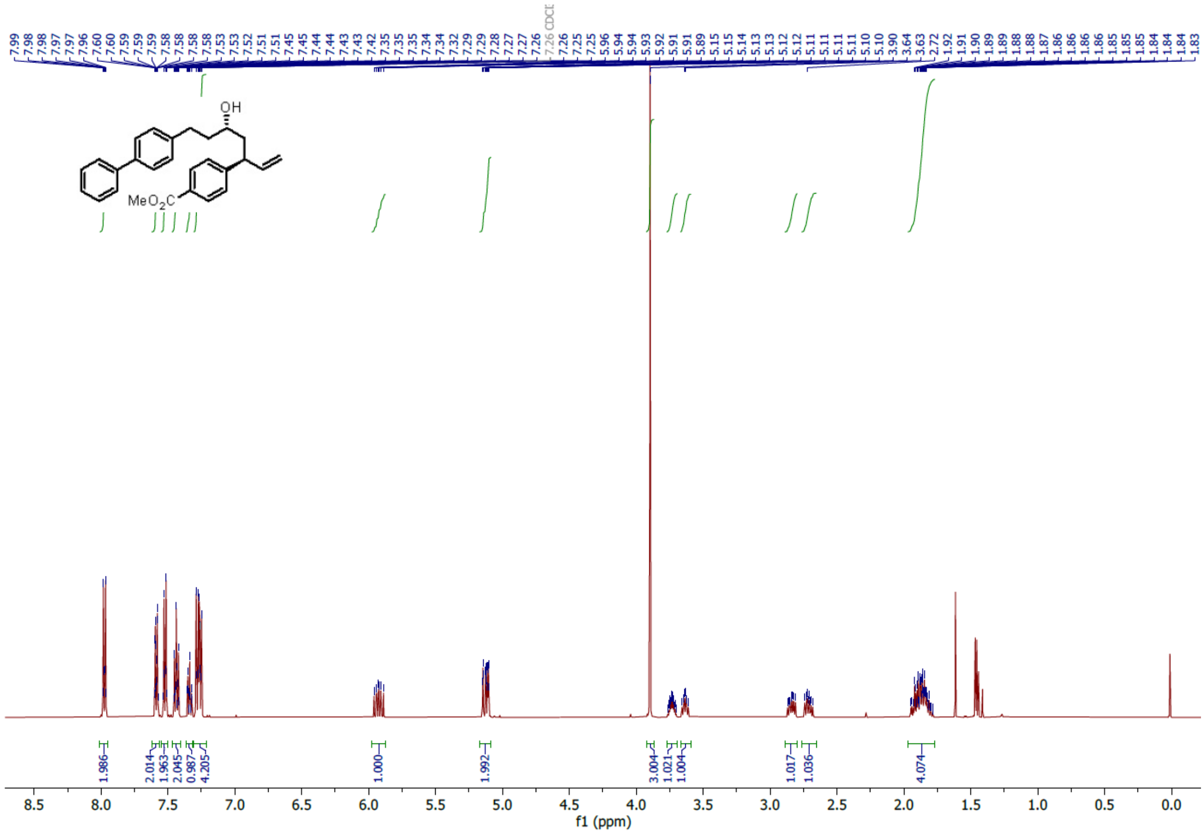

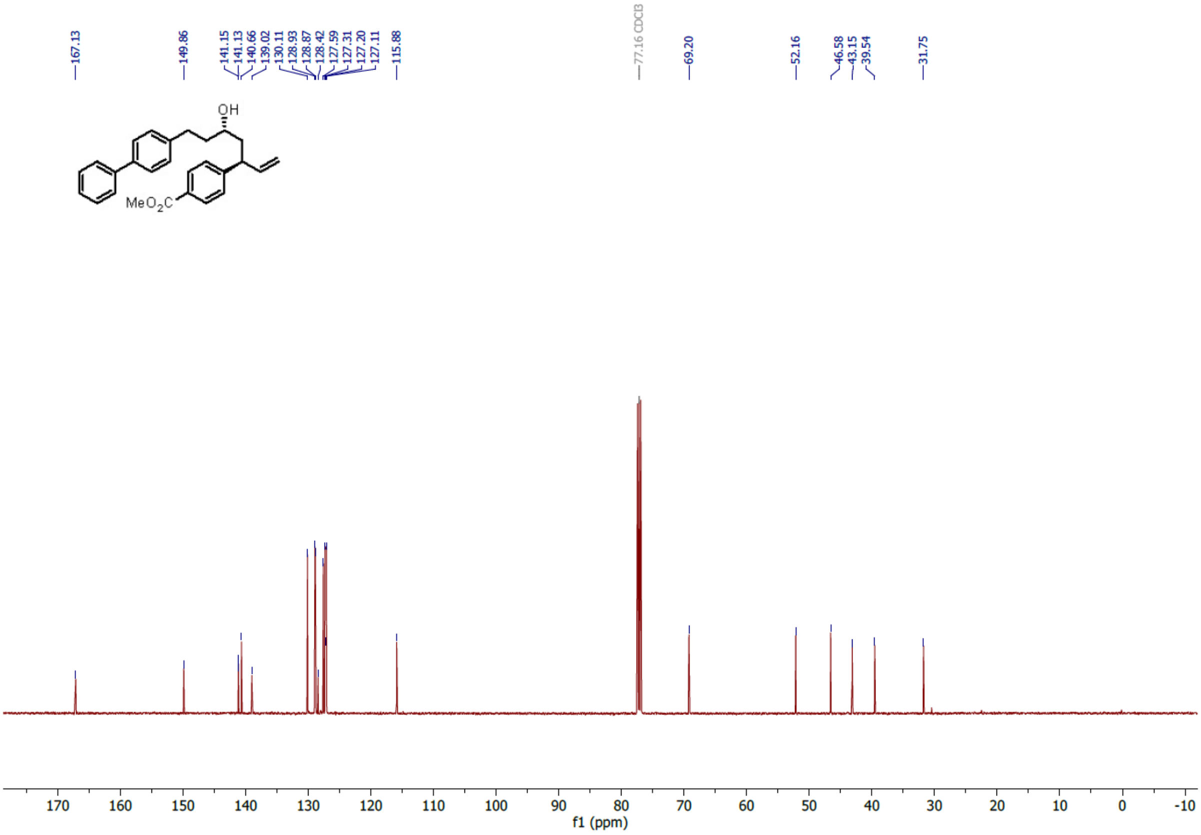

Compound 37

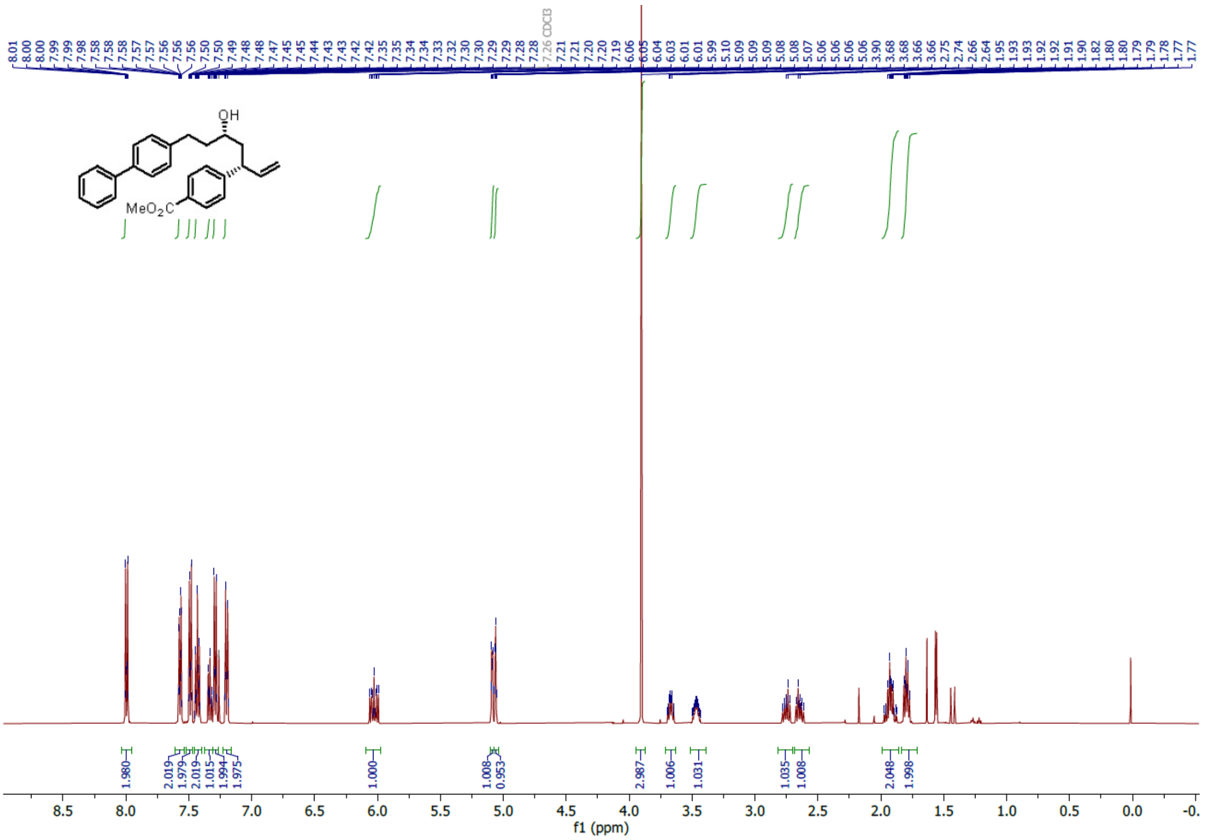
8.01 8.00 8.00 7.99 7.99 7.98 7.58 7.58 7.58 7.57 7.56 7.56 7.50 7.50 7.49 7.48 7.48 7.47 7.45 7.45 7.44 7.43 7.43 7.42 7.42 7.35 7.35 7.34 7.33 7.32 7.30 7.30 7.29 7.29 7.28 7.26 CDCl3 7.21 7.21 7.20 7.20 7.19 6.06 6.05 6.04 6.03 6.01 6.01 5.99 5.10 5.09 5.09 5.09 5.08 5.08 5.07 5.06 5.06 5.06 5.06 3.90 3.68 3.68 3.66 3.66 2.75 2.74 2.66 2.64 1.95 1.93 1.93 1.92 1.92 1.91 1.90 1.82 1.80 1.80 1.79 1.79 1.78 1.77 1.77
OH
MeO2C
1.980
2.019 1.979 2.019 1.015 1.994 1.975
1.000
1.008 0.953
2.987 1.006 1.031
1.035 1.008
2.048 1.998
f1 (ppm)

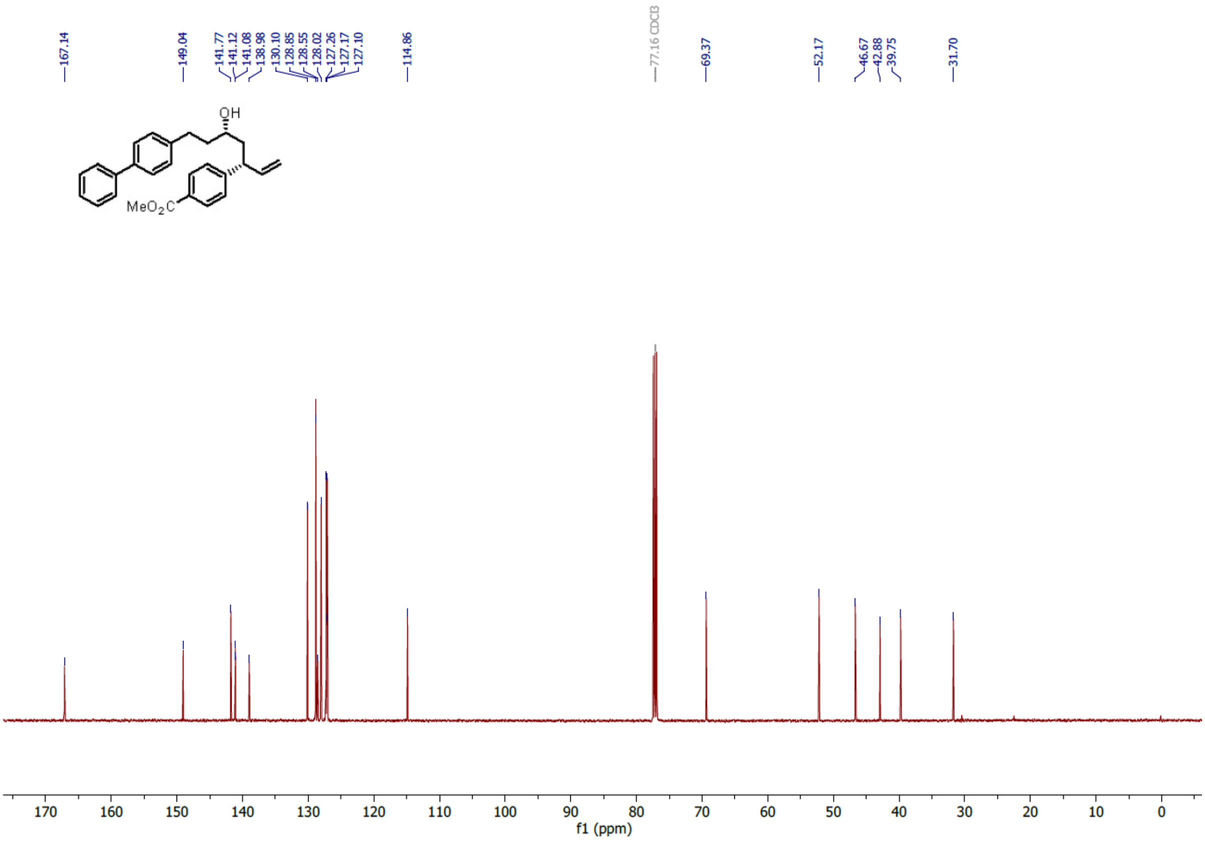
167.14
149.04
141.77 141.12 141.08 138.98 130.10 128.85 128.55 128.02 127.26 127.17 127.10
114.86
77.16 CDCl3
69.37
52.17
46.67 42.88 39.75
31.70
OH
MeO2C
f1 (ppm)

Compound 38

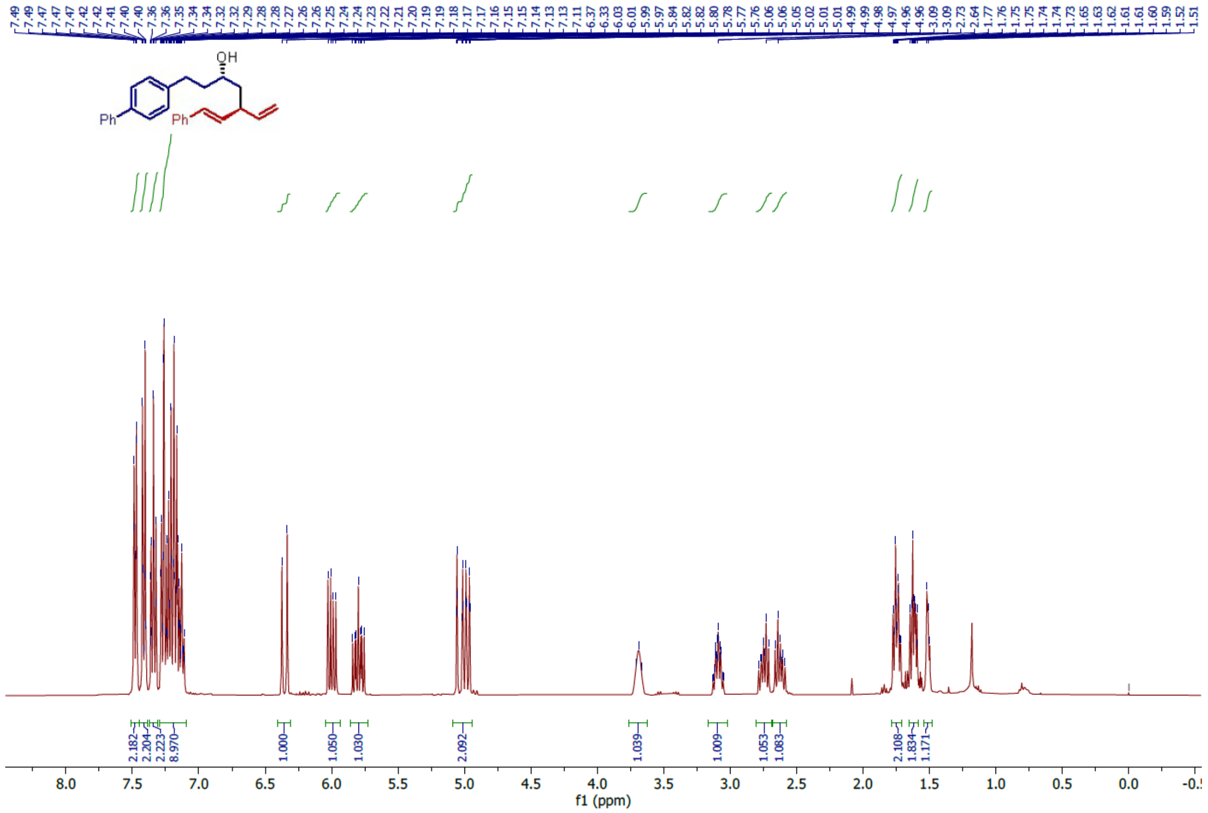

7.49 7.49 7.47 7.47 7.47 7.42 7.42 7.41 7.40 7.40 7.36 7.36 7.35 7.34 7.34 7.32 7.32 7.29 7.28 7.28 7.27 7.26 7.26 7.25 7.24 7.24 7.23 7.22 7.21 7.20 7.19 7.19 7.18 7.17 7.17 7.16 7.15 7.15 7.14 7.13 7.13 7.11 6.37 6.33 6.03 6.01 5.99 5.97 5.84 5.82 5.82 5.80 5.78 5.77 5.76 5.06 5.06 5.05 5.02 5.01 5.01 4.99 4.99 4.98 4.97 4.96 4.96 3.09 3.09 2.73 2.64 1.77 1.76 1.75 1.75 1.74 1.74 1.73 1.65 1.63 1.62 1.61 1.61 1.60 1.59 1.52 1.51
OH
Ph
Ph
2.182 2.204 2.223 8.970 1.000 1.050 1.030 2.092 1.039 1.009 1.053 1.083 2.108 1.834 1.171
8.0 7.5 7.0 6.5 6.0 5.5 5.0 4.5 4.0 3.5 3.0 2.5 2.0 1.5 1.0 0.5 0.0 -0.5
f1 (ppm)

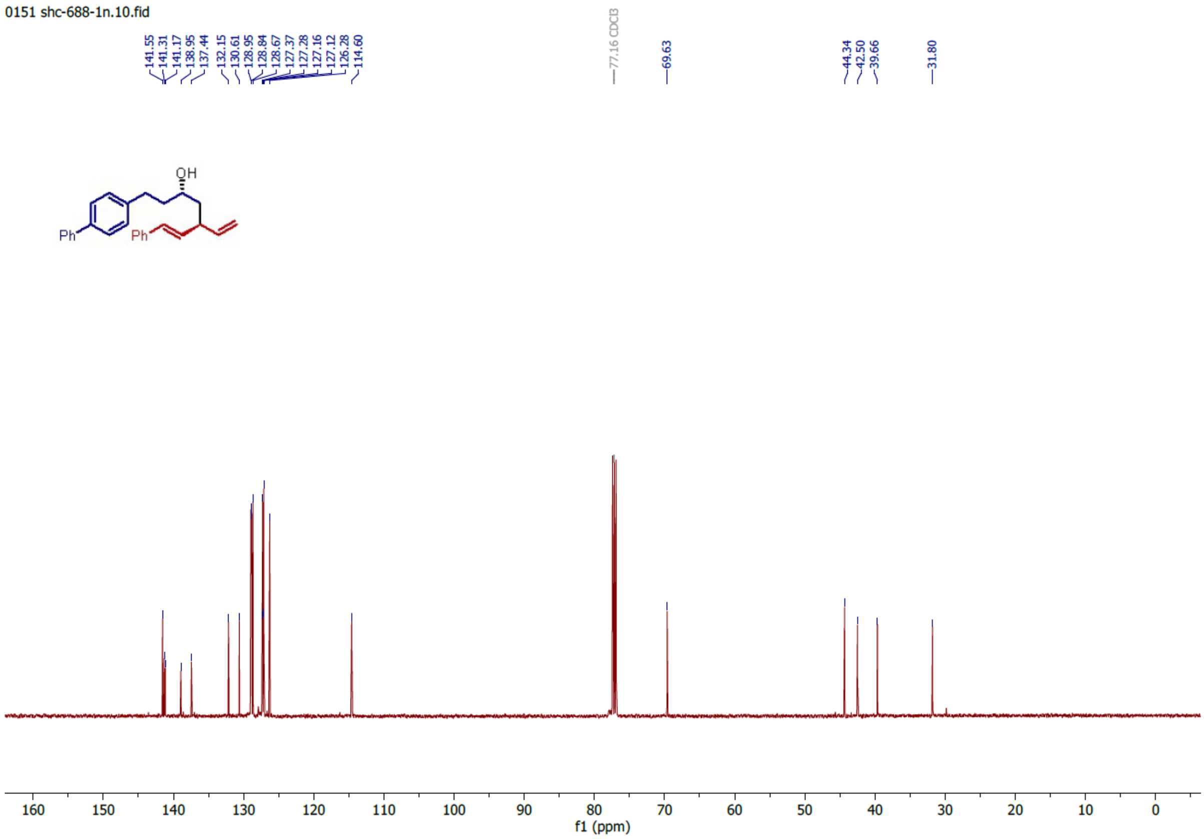

0151 shc-688-1n.10.fid
141.55 141.31 141.17 138.95 137.44 132.15 130.61 128.95 128.84 128.67 127.37 127.28 127.16 127.12 126.28 114.60
77.16 CDCl3
69.63
44.34 42.50 39.66
31.80
OH
Ph
Ph
160 150 140 130 120 110 100 90 80 70 60 50 40 30 20 10 0
f1 (ppm)

Compound 39

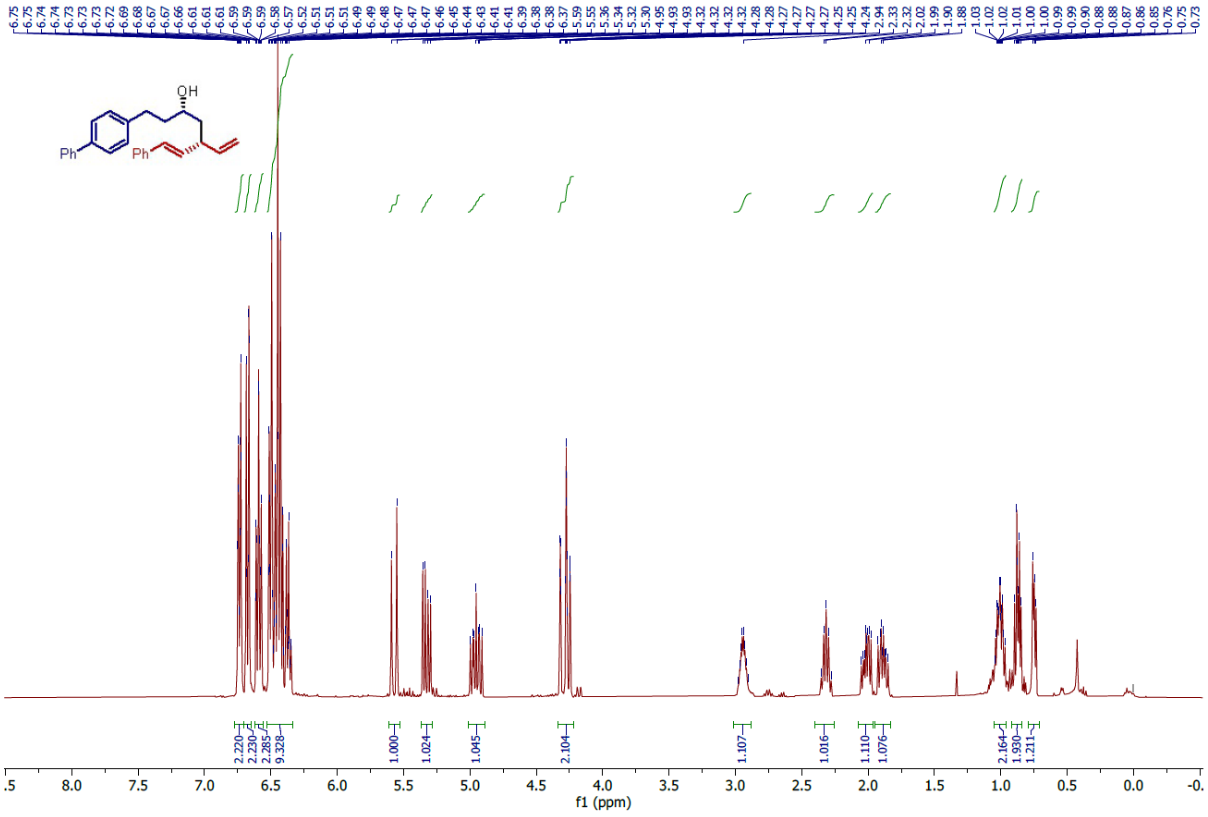

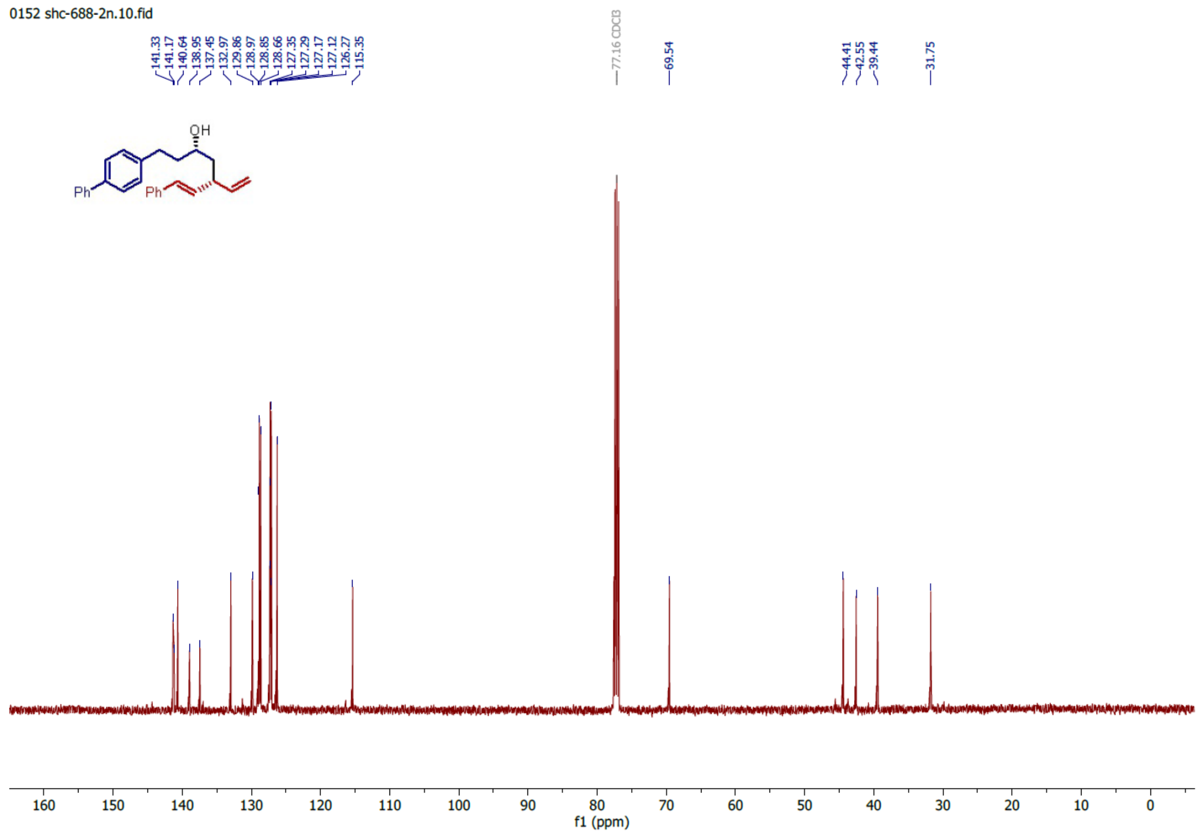

Compound 45

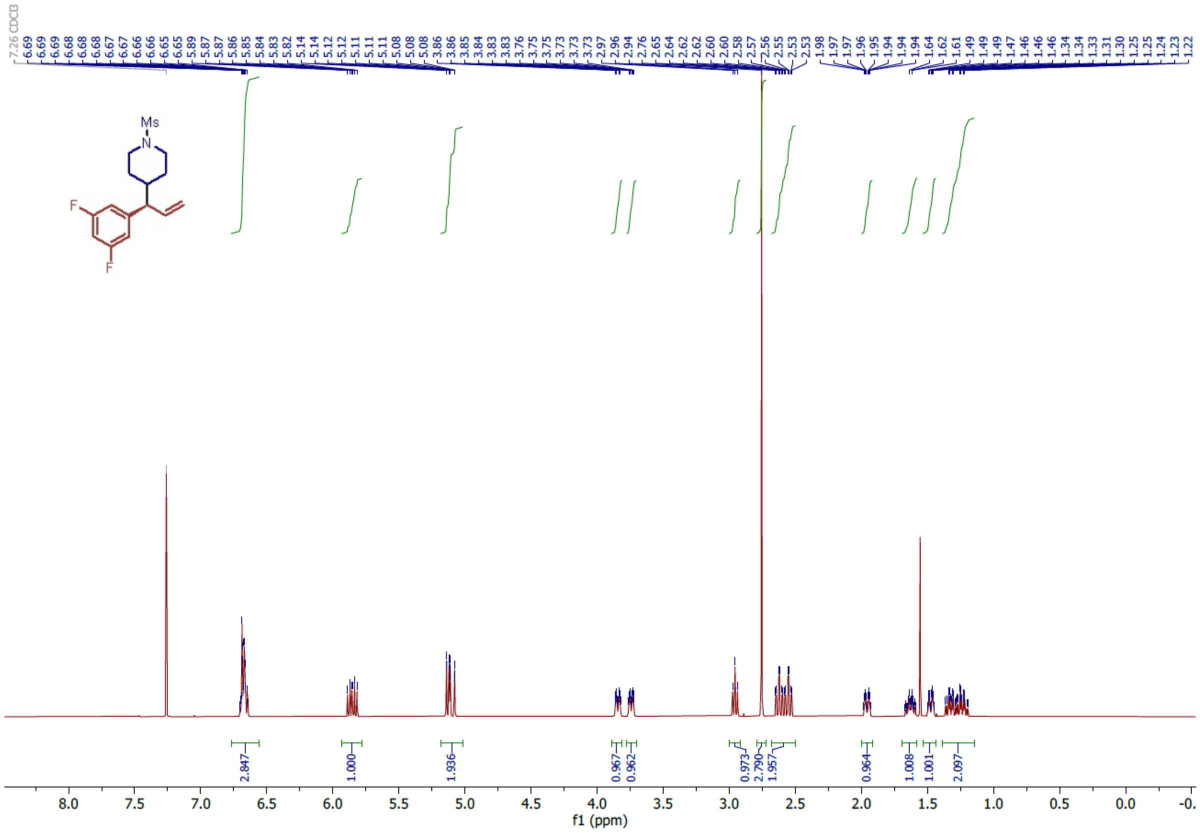

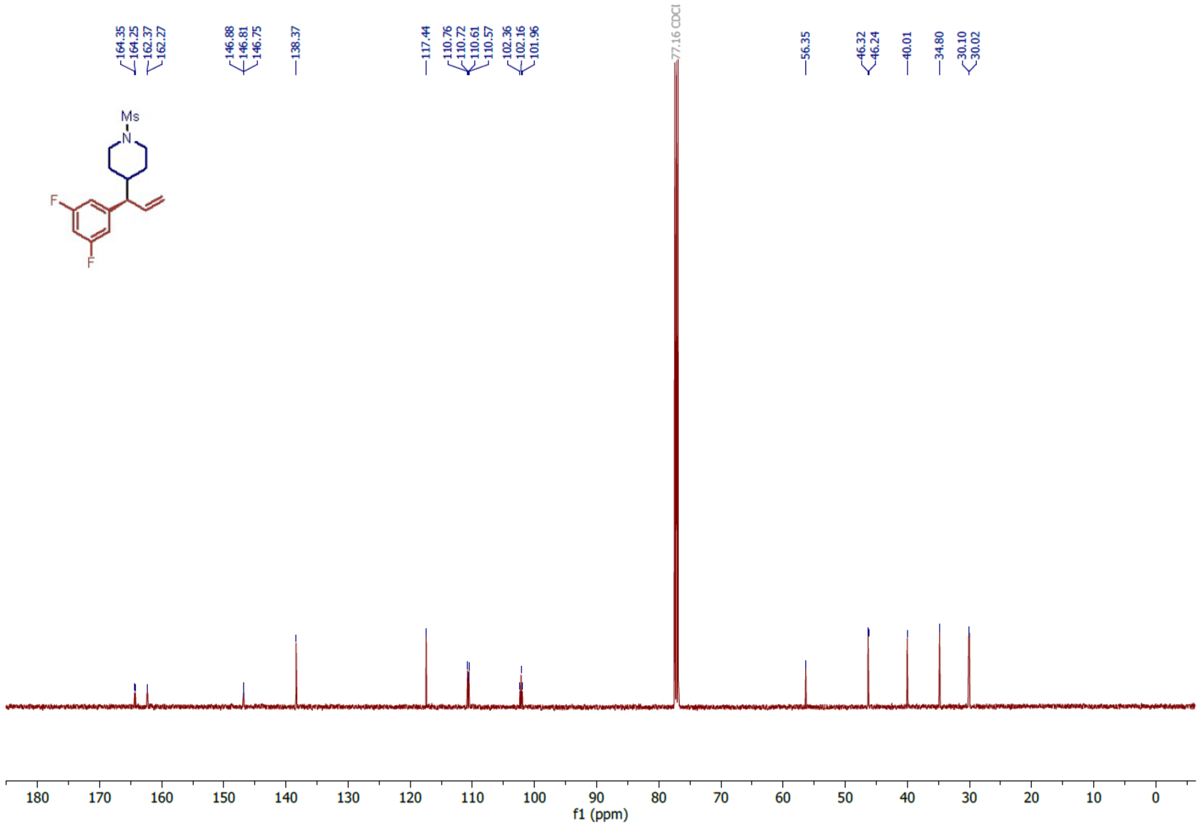

Compound 46

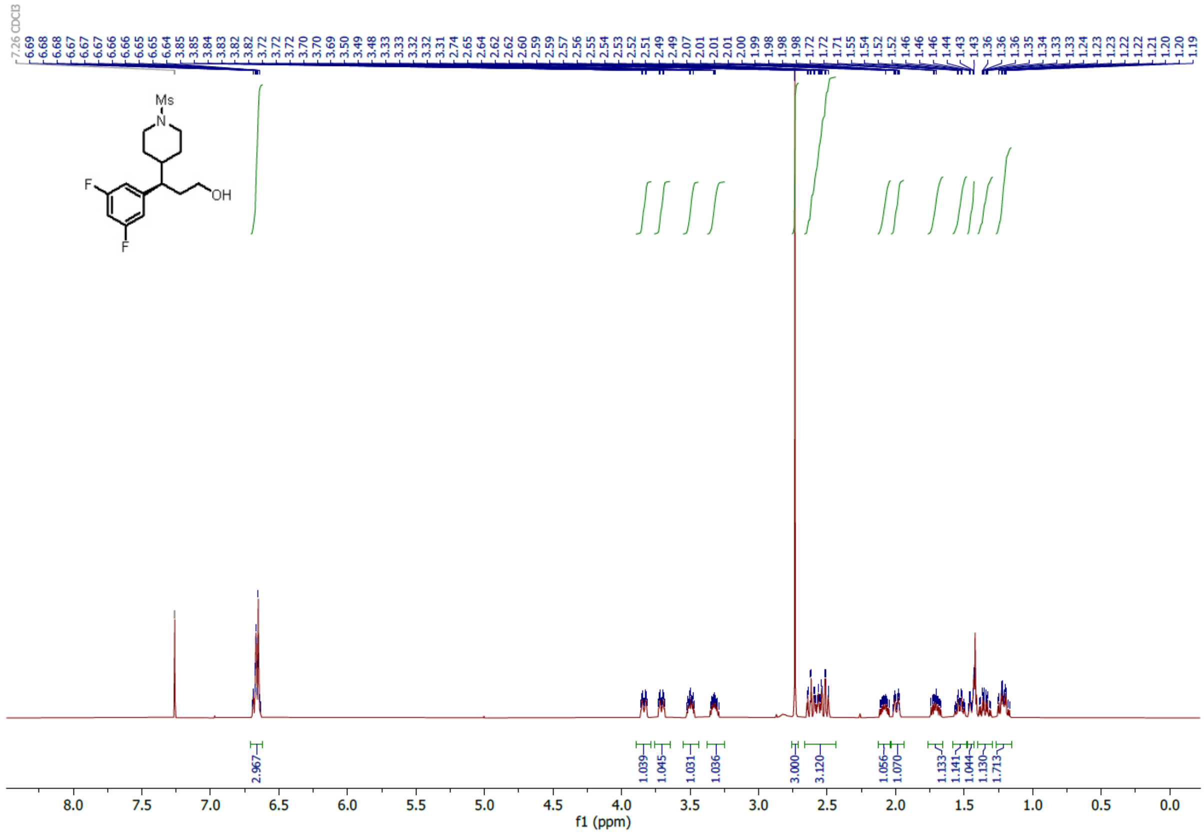

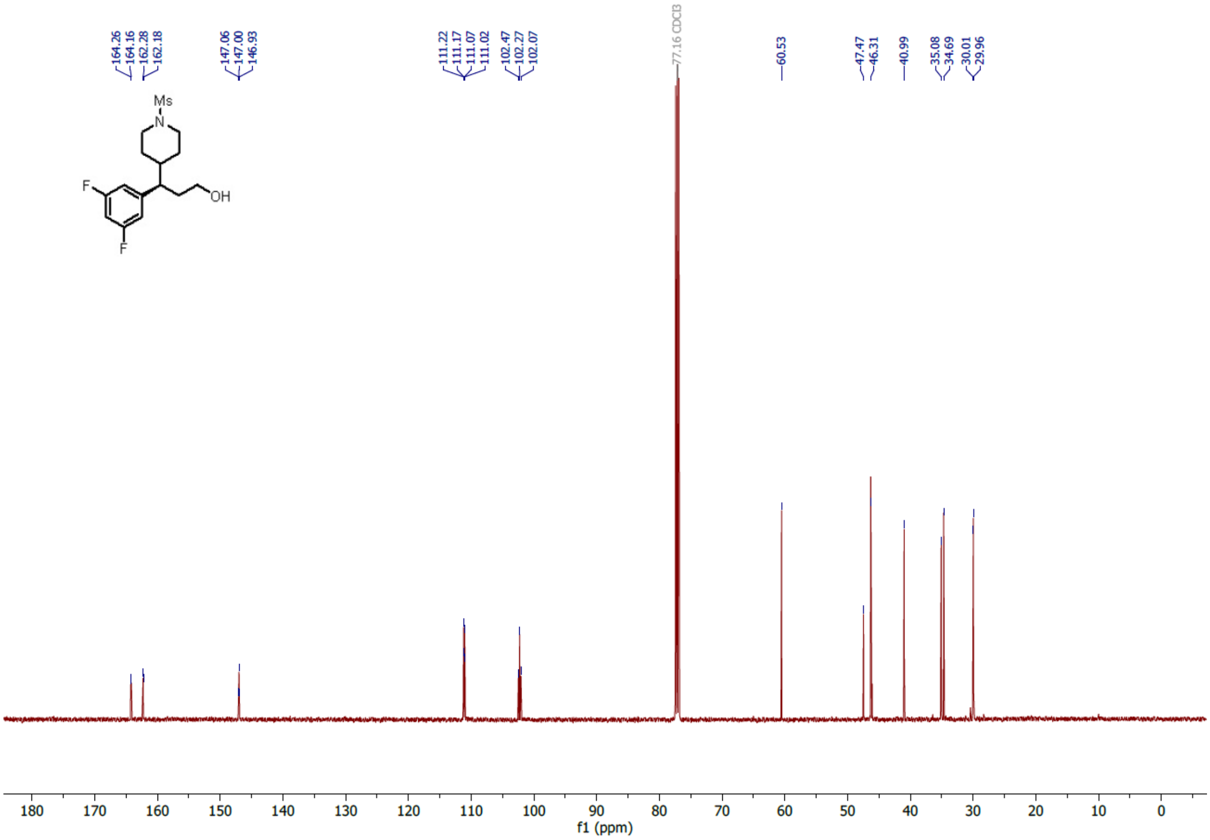

Compound 47

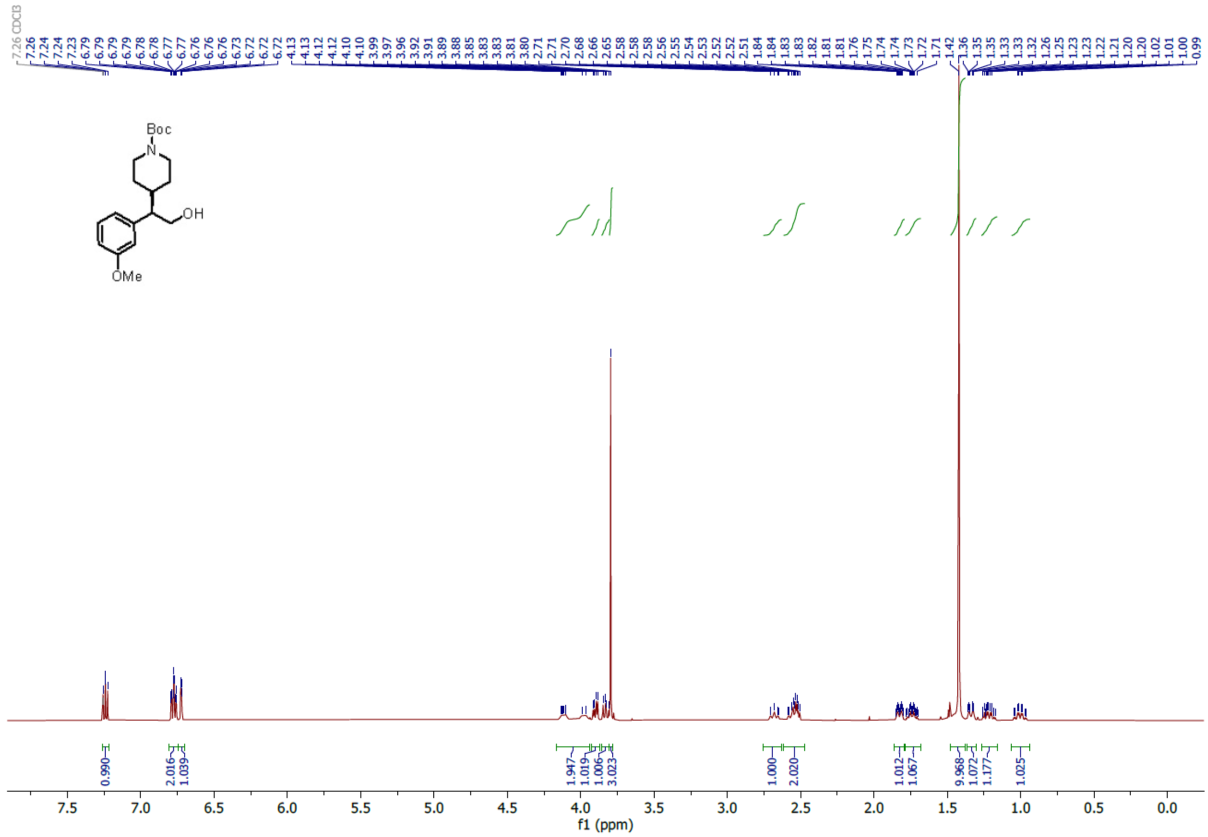

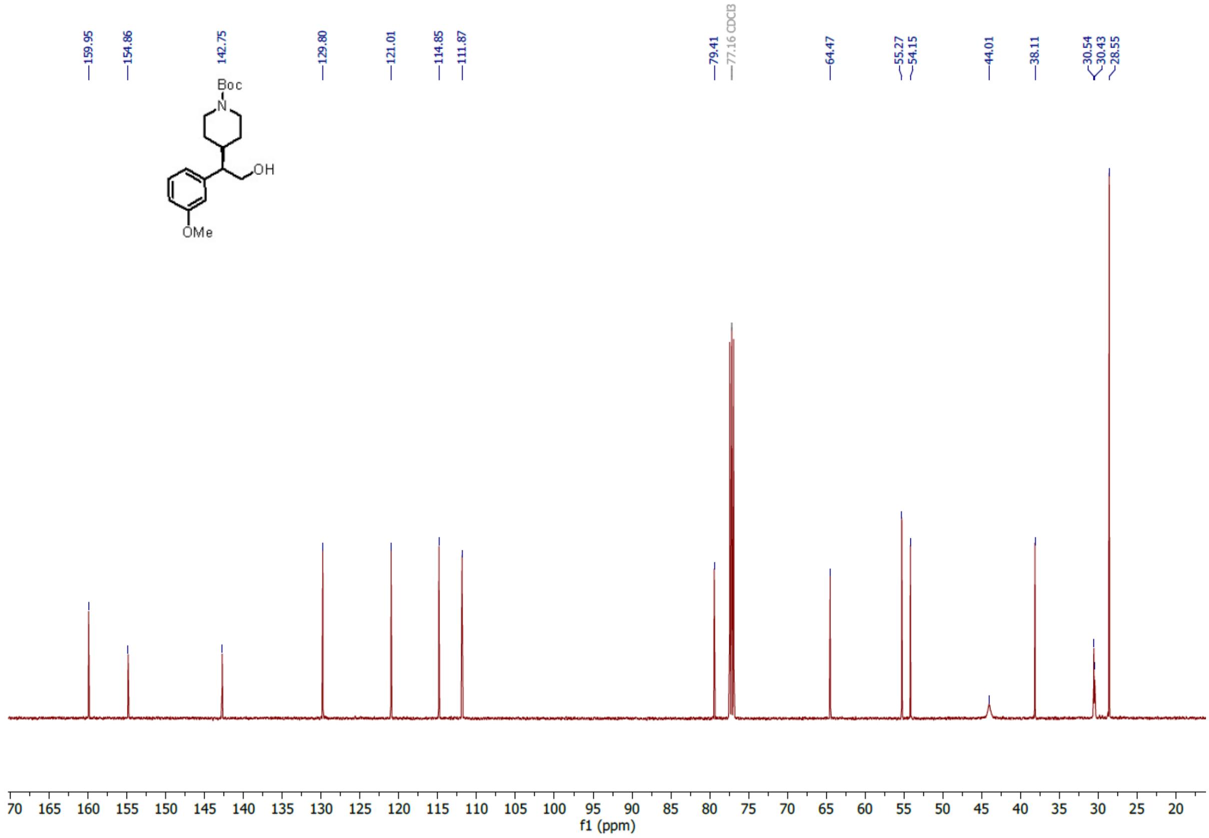

Compound 48

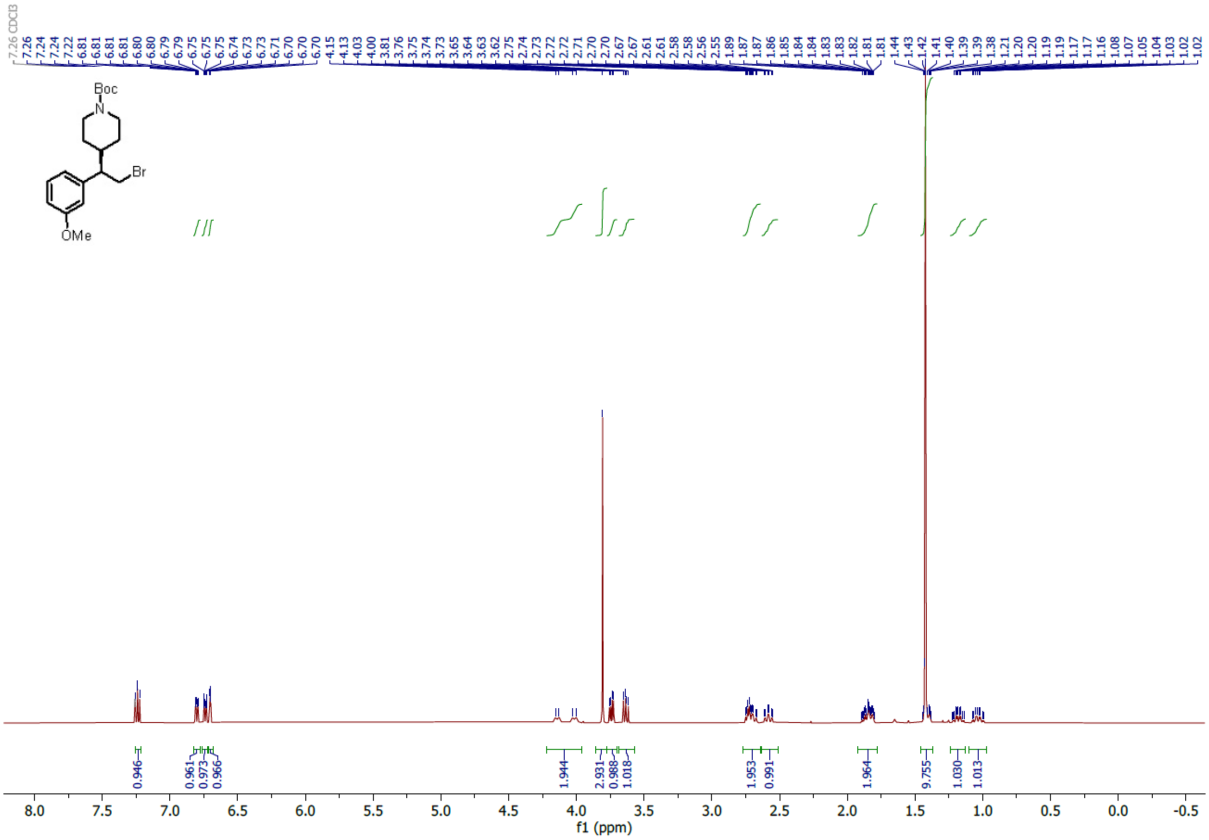

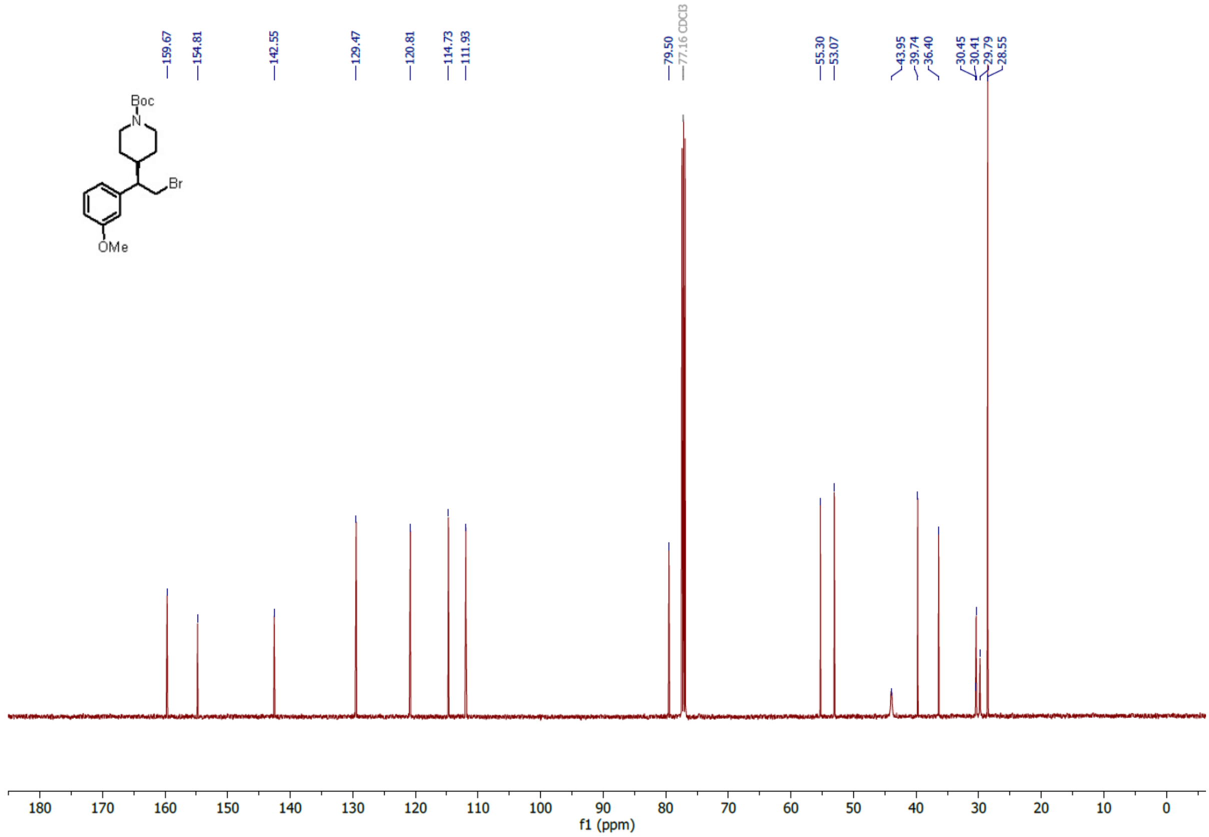

Compound 49

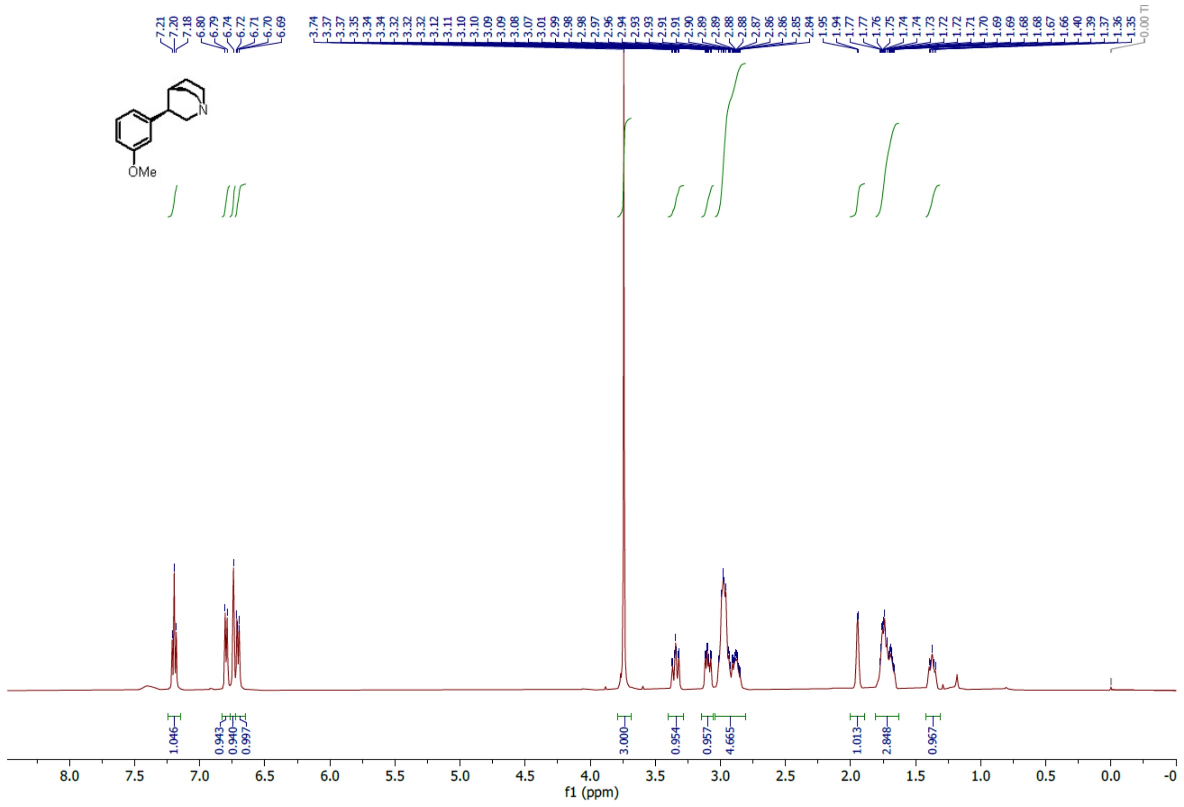

OMe
f1 (ppm)

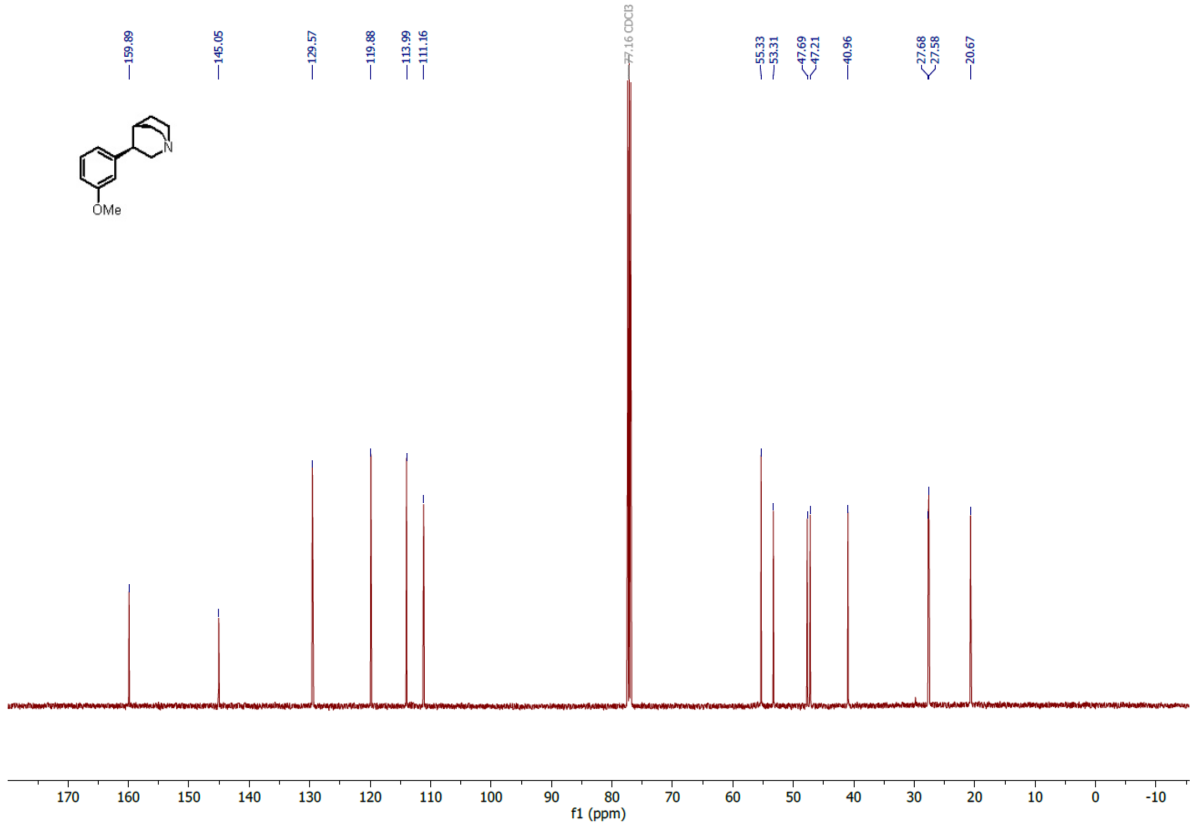

OMe
159.89
145.05
129.57
119.88
113.99
111.16
77.16 CDCl3
55.33
53.31
47.69
47.21
40.96
27.68
27.58
20.67
f1 (ppm)

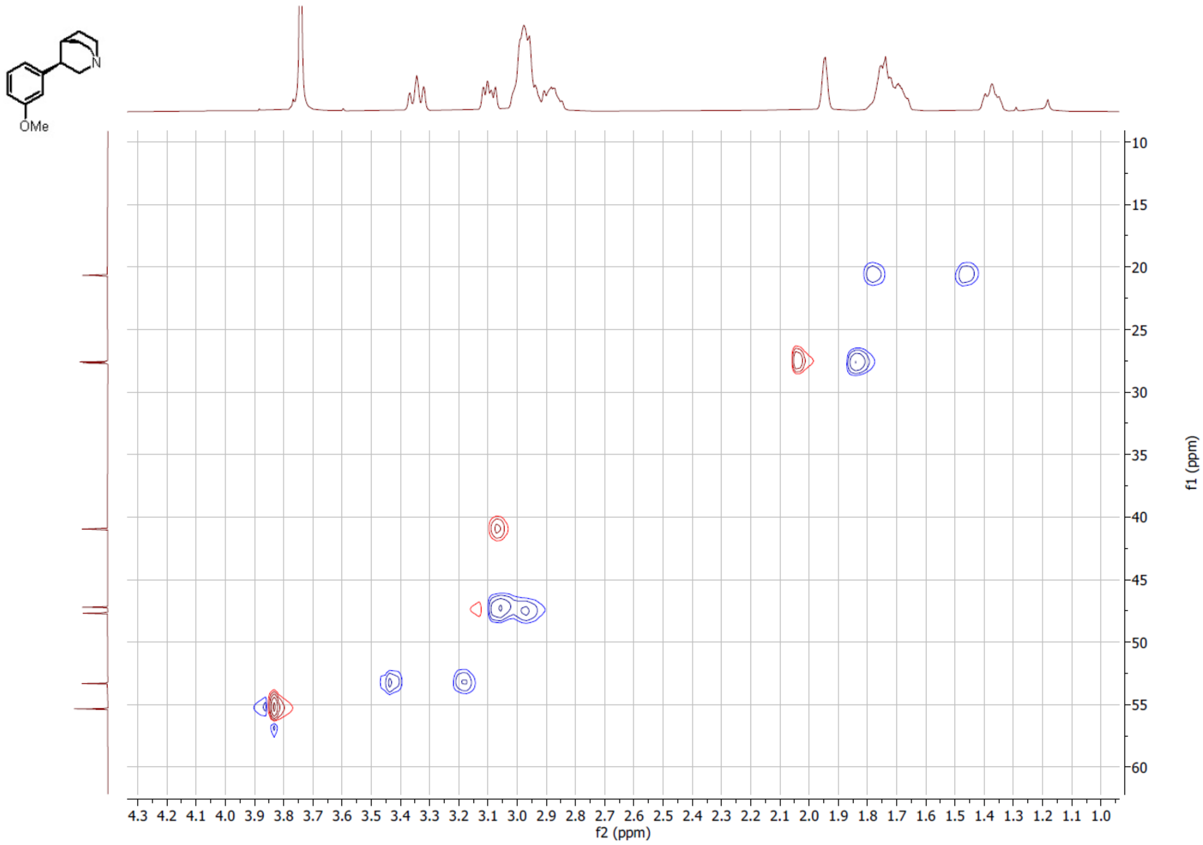
OMe
N
f2 (ppm)
f1 (ppm)

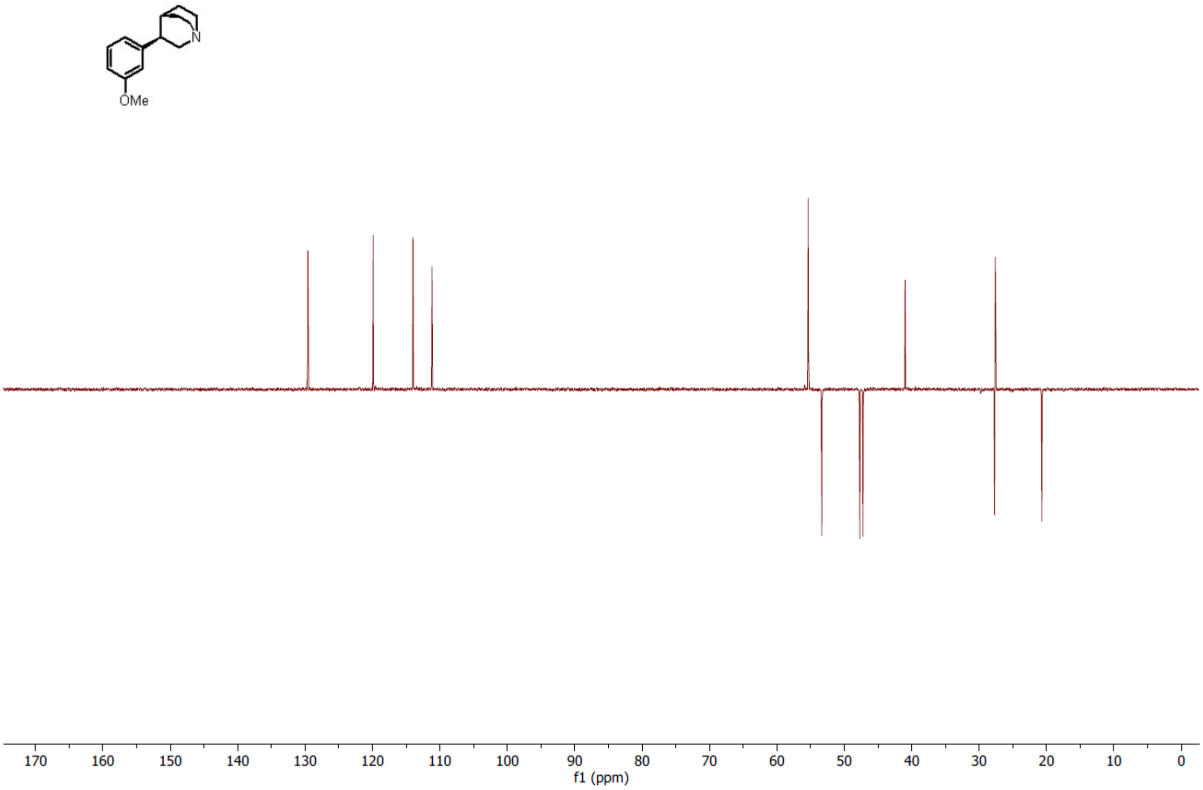
OMe
N
f1 (ppm)
